# GO-Diff: Mining functional differentiation between EST-based transcriptomes

**DOI:** 10.1186/1471-2105-7-72

**Published:** 2006-02-16

**Authors:** Zuozhou Chen, Weilin Wang, Xuefeng Bruce Ling, Jane Jijun Liu, Liangbiao Chen

**Affiliations:** 1 College of Life Science, Zhejiang University, Hangzhou 310029, China; 2 Laboratory of Molecular and Developmental Biology, Institute of Genetics and Developmental Biology, Chinese Academy of Sciences, Beijing 100080, China; 3 Center of Organ Transplantation, First Affiliated Hospital, College of Medicine, Zhejiang University, Hangzhou, 310003, China; 4 Amgen Inc., South San Francisco, CA 94080, USA

## Abstract

**Background:**

Large-scale sequencing efforts produced millions of Expressed Sequence Tags (ESTs) collectively representing differentiated biochemical and functional states. Analysis of these EST libraries reveals differential gene expressions, and therefore EST data sets constitute valuable resources for comparative transcriptomics. To translate differentially expressed genes into a better understanding of the underlying biological phenomena, existing microarray analysis approaches usually involve the integration of gene expression with Gene Ontology (GO) databases to derive comparable functional profiles. However, methods are not available yet to process EST-derived transcription maps to enable GO-based global functional profiling for comparative transcriptomics in a high throughput manner.

**Results:**

Here we present GO-Diff, a GO-based functional profiling approach towards high throughput EST-based gene expression analysis and comparative transcriptomics. Utilizing holistic gene expression information, the software converts EST frequencies into EST Coverage Ratios of GO Terms. The ratios are then tested for statistical significances to uncover differentially represented GO terms between the compared transcriptomes, and functional differences are thus inferred. We demonstrated the validity and the utility of this software by identifying differentially represented GO terms in three application cases: intra-species comparison; meta-analysis to test a specific hypothesis; inter-species comparison. GO-Diff findings were consistent with previous knowledge and provided new clues for further discoveries. A comprehensive test on the GO-Diff results using series of comparisons between EST libraries of human and mouse tissues showed acceptable levels of consistency: 61% for human-human; 69% for mouse-mouse; 47% for human-mouse.

**Conclusion:**

GO-Diff is the first software integrating EST profiles with GO knowledge databases to mine functional differentiation between biological systems, e.g. tissues of the same species or the same tissue cross species. With rapid accumulation of EST resources in the public domain and expanding sequencing effort in individual laboratories, GO-Diff is useful as a screening tool before undertaking serious expression studies.

## Background

Cellular development and its associated biochemical processes within and between various cell types are determined by the relevant cellular proteomes, which are tightly regulated by biochemical synthesis, different stage genetic interactions and various metabolic pathways. The proteome of a cell is largely (but not exclusively) regulated by gene expression [1], and the transcriptome can be regarded as a sensitive read-out of the proteome revealing the biochemical state of the cell. Currently the most popular gene expression analysis platforms include gene microarray [2] and the serial analysis of gene expression (SAGE) [3]. To analyze the molecular and cellular processes and probe the principles, mechanisms, and major developmental events giving rise to diverse tissue types, gene expression analysis has become an indispensable approach to facilitate our understanding of biology. Developmental abnormalities, including tumor, have also been explored through tumor expression profiling analysis to discover the contributing genetic and extrinsic factors.

Many genes participating in the same biological process are co-regulated and these periodically expressed genes drive the dynamics of the underlying biological processes, such as the periodically expressed protein complexes during the yeast cell cycles [4]. However, to discover such functional dynamics and their associated gene members directly from expression data is both biologically important and computationally challenging [5,6]. Nevertheless, from the biological perspective, it is imperative to integrate and associate gene expression with molecular functions, cellular components, and biological processes, thus allowing the comparative transcriptomic analysis to be an effective biological knowledge mining process. Through a taxonomy of biological concepts and their species-independent attributes for annotating gene sequences, the Gene Ontology (GO) [7,8], serves as a shared language, standardizing biological vocabularies, for communicating biological data and knowledge for comparative genomics and comparative transcriptomics.

The GO database schema models a directed acyclic graph (DAG) relationally, and the terms (graph nodes) and term-term relationships provide the conceptualizations of biological domains of knowledge [9]. High throughput annotation methods [10-13] can electronically annotate any uncharacterized protein or transcript through identifying GO annotated domains or aligning with GO annotated model organism sequences. For example, DIAN [10] and InterProScan[14] apply domain-mapping approaches to assign sequences with GO terms, GOtcha [11] predicts uncharacterized sequences' GO associations by assign each association a term-specific probability (P-score) as a measure of confidence and AutoFACT [12] combines multiple BLAST reports from several user-selected databases to predict GO associations. These tools are good for genome annotators, where the goal is for gene annotation and classification purposes. Thanks to the GO consortium, gene sequences of model organisms, either from manual curatorial efforts or from direct experimental evidences, have been well characterized with high quality GO annotations. High-quality manual and computational GO annotations provide invaluable resource and solid groundwork for additional data mining and biological mechanism characterization.

The advances in microarray technology and data mining studies allow the simultaneous analysis of all genes in the entire transcriptome, producing differentially regulated gene lists in the condition under study. To obtain the biological significance, these differentially expressed genetic profiles should be interpreted under the contexts of molecular functions, biological processes and cellular components. The GO databases have been utilized as tools to annotate these differentially expressed genes [15]. By comparing the number of differentially expressed genes with those of background genes at each GO graph node, over represented GO terms can be identified to translate the gene lists into a better understanding of the biological phenomena involved [16-21]. This approach of focusing on the genes with high magnitude of changes and relying on these sparse annotations with specific GO terms ignores the majority of the expression data sets, and may fail the detection of considerably more subtle changes in gene networks [22]. To address this, methods have been developed to evaluate GO terms utilizing Holistic Expression information (GHE) to obtain functional analysis, such as GO-Mapper, GOAL and GOdist [22-24].

The availability of the huge amount of expressed sequence tags (ESTs)[25] have made it possible to construct various tissue specific transcriptomes, thus allowing much more flexibility in the areas of large scale comparative transcriptomics analysis between different biological systems. Specifically, the dbEST, a division of GenBank, has collected 31,307,034 ESTs from 976 species, of which 474 species having at least 1,000 sequence tags (dbEST release 111105, Nov, 11,2005). To support the EST-based gene expression analysis, software tools have been developed to convert the EST frequencies into readily analyzable transcription maps to identify differentially expressed genes, which include Digital Differential Display [26,27], cDNA xProfiler [28], cDNA Digital Gene Expression Displayer (DGED) [29], and DigiNorthern [30]. However, methods are not available yet to analyze EST derived transcription maps to extract GO terms that are either significantly over- or under-represented to enable global functional profiling for comparative transcriptomics.

GO based microarray profiling analysis approach, however, cannot readily be applied to EST based transcription analysis and functional profiling. First, unlike microarry, where gene expression is normally distributed, EST (and also SAGE) data is generated by random sampling, results in "tag counts", governed by Poisson distribution [31,32]. Thus, statistical approaches for EST-analyses are different. It has been shown that Chi-square test performed the best among several statistical methods in the EST and SAGE analyses [32]. EST analysis is based upon the count of the sequence tags where some have sufficient while others have insufficient tag counts. As a consequence, microarray analysis approaches cannot be directly applicable. Third, the gene expression representations are different between the microarray and EST data sets, not easily accommodated by current microarray analytical tools. In contrast to the difficulty to compare microarray data cross array platforms, unbiased EST libraries can be easily combined and compared. This is because EST data sets are in the same data formats, and are generated and processed with similar procedures.

In this study, we present GO-Diff, a GO-based system biology approach for high throughput comparative transcriptomics. The algorithm implementation can comprehensively integrate and efficiently process large EST-based transcription maps, and directly compare different biological systems, e.g. the same-type tissue samples from different developmental stages or from different species, based upon GO term representation analysis. Three comparative transcriptomics analyses were described to demonstrate GO-Diff's validities and data mining utilities. A quantitative evaluation was also conducted to evaluate the consistency of GO-Diff performance.

## Implementation

### GO-Diff knowledge base

The GO-Diff knowledge base comes from three contributing resources. The GO structure information, as described in the standard OBO file, was downloaded from the GO website [33]. The mapping between Unigenes and GO terms was constructed through the integration of the Gene-GO mapping and the Gene-Unigene mapping [34]. The Unigene-GO mapping is also readily available from other resources including the GOA Uniprot-GO and the Uniprot-Gene [35] mappings in human, mouse, rat and zebrafish.

EST frequencies are computed for all Unigenes in each dbEST tissue specific transcription map within the knowledge base. Source files are downloaded from the Unigene FTP site [36]. The GO-Diff Knowledge base can be updated via the GO-Diff update programs to integrate latest EST data sets and GO knowledge data sets. The GO evidence codes are integrated as part of the knowledge base. In order to assist the user to focus and limit the search space, those GO terms corresponding to irrelevant biological knowledge can be excluded from further analysis if relevant GO term evidence codes are selected.

### GO-Diff algorithm

**Figure 1:**
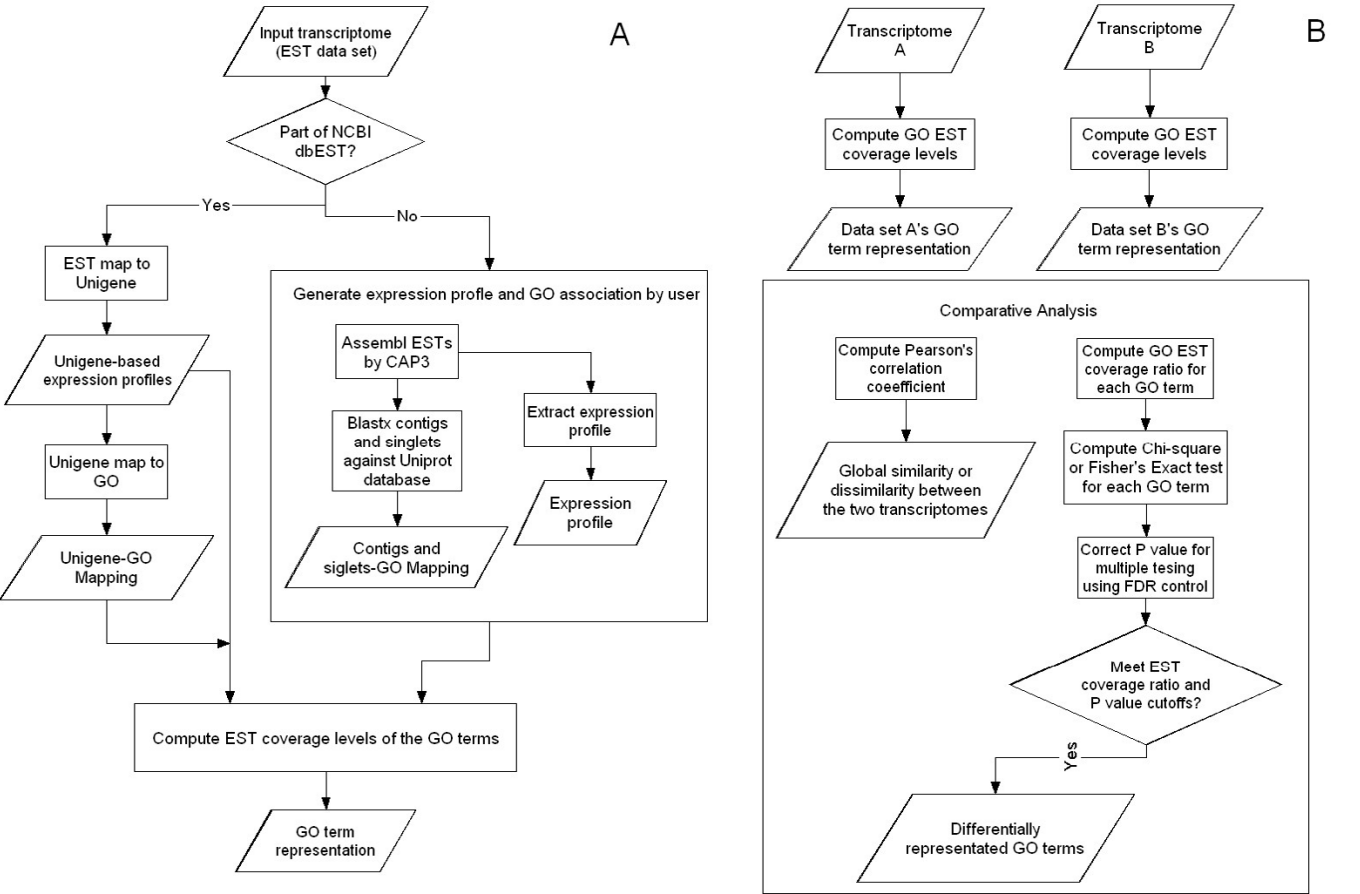
A. Flow diagram of GO term representation calculation B. Overview diagram of GO-Diff algorithm.

The algorithm flow chart is diagramed in Figure 1A and 1B. GO-Diff is designed and implemented to perform comparative transcriptomics with the following three analytical options: comparing dbEST libraries captured in GO-Diff knowledge base; comparing dbEST libraries captured in GO-Diff knowledge base with a user-defined EST transcriptome; comparing EST transcriptomes which are both defined by the user. The EST libraries within the knowledge base can be selected through the descriptive keywords or dbEST library identification numbers for comparative analysis. The expression profile for each sample of interest is based upon the EST frequencies of Unigenes computed from that sample's unbiased EST libraries. The Unigene clusters serve as the bridge between the EST-based gene expression and biological knowledge encapsulated by GO terms, leading to the construction of the "GO profiles" for the biological samples. The EST-based gene expression analysis essentially is the comparative dissection of the two GO profiles.

Independent of Unigene, another approach to link EST to the GO terms and construct EST-based expression profile is through sequence assembly and direct sequence GO annotation. This approach has the advantages to perform GO-Diff analysis *de novo *and does not depend on previous Unigene annotations. This is especially true when these EST sequences are novel and fresh from an ongoing sequence project. In addition, this approach can maximally utilize the EST information in a given transcriptomes. However, the computational cost might be heavy. EST sequences are assembled into contigs using sequence assembly tools such as CAP3 [37], Phrap[38] and TIGR assembler [39] to BLAST against the GO annotated databases. GoPipe [40,41] and other tools are used to post process the BLAST results and extract GO annotations for the assembled contigs. The expression profile for each sample of interest is based upon the EST frequencies of these contigs. Like the Unigene clusters, the assembled contigs link the EST information to the GO terms to construct a GO profile for that particular transcriptome.

We define the "**E**ST **C**overage **L**evel of a **G**O Term" (**ECLG**) as the total of the ESTs of the Unigenes or contigs mapped to a specific GO term, where x_i _is the EST count of Unigene cluster or contig *i *that is associated with a specific GO term.

ECLGO−Id=fGO−Id(x1,x2,…,xn)=∑i=1nxi     (1)
 MathType@MTEF@5@5@+=feaafiart1ev1aaatCvAUfKttLearuWrP9MDH5MBPbIqV92AaeXatLxBI9gBaebbnrfifHhDYfgasaacH8akY=wiFfYdH8Gipec8Eeeu0xXdbba9frFj0=OqFfea0dXdd9vqai=hGuQ8kuc9pgc9s8qqaq=dirpe0xb9q8qiLsFr0=vr0=vr0dc8meaabaqaciaacaGaaeqabaqabeGadaaakeaacqWGfbqrcqWGdbWqcqWGmbatdaWgaaWcbaGaem4raCKaem4ta8KaeyOeI0IaemysaKKaemizaqgabeaakiabg2da9iabdAgaMnaaBaaaleaacqWGhbWrcqWGpbWtcqGHsislcqWGjbqscqWGKbazaeqaaOGaeiikaGIaemiEaG3aaSbaaSqaaiabigdaXaqabaGccqGGSaalcqWG4baEdaWgaaWcbaGaeGOmaidabeaakiabcYcaSiablAciljabcYcaSiabdIha4naaBaaaleaacqWGUbGBaeqaaOGaeiykaKIaeyypa0ZaaabCaeaacqWG4baEdaWgaaWcbaGaemyAaKgabeaaaeaacqWGPbqAcqGH9aqpcqaIXaqmaeaacqWGUbGBa0GaeyyeIuoakiaaxMaacaWLjaWaaeWaaeaacqaIXaqmaiaawIcacaGLPaaaaaa@5A66@

The ECLG not only covers the ESTs directly linked to a GO term, but also includes the ESTs associated with its children GO nodes due to the "true path rule".

We define the "**R**elative **E**ST **C**overage **L**evel of a **G**O Term" (**RECLG**) as the proportion of the ESTs under the specific GO term in total ESTs with GO term annotations. *X_All-Go _*is the number of total ESTs within the Unigene clusters or the contigs that have the GO term annotations.

RECLGGO−Id=∑i=1nxi/XAll−GO=∑i=1nxi/∑i=1mxi     (2)
 MathType@MTEF@5@5@+=feaafiart1ev1aaatCvAUfKttLearuWrP9MDH5MBPbIqV92AaeXatLxBI9gBaebbnrfifHhDYfgasaacH8akY=wiFfYdH8Gipec8Eeeu0xXdbba9frFj0=OqFfea0dXdd9vqai=hGuQ8kuc9pgc9s8qqaq=dirpe0xb9q8qiLsFr0=vr0=vr0dc8meaabaqaciaacaGaaeqabaqabeGadaaakeaacqWGsbGucqWGfbqrcqWGdbWqcqWGmbatcqWGhbWrdaWgaaWcbaGaem4raCKaem4ta8KaeyOeI0IaemysaKKaemizaqgabeaakiabg2da9maaqahabaGaemiEaG3aaSbaaSqaaiabdMgaPbqabaGccqGGVaWlcqWGybawdaWgaaWcbaGaemyqaeKaemiBaWMaemiBaWMaeyOeI0Iaem4raCKaem4ta8eabeaaaeaacqWGPbqAcqGH9aqpcqaIXaqmaeaacqWGUbGBa0GaeyyeIuoakiabg2da9maaqahabaGaemiEaG3aaSbaaSqaaiabdMgaPbqabaGccqGGVaWlaSqaaiabdMgaPjabg2da9iabigdaXaqaaiabd6gaUbqdcqGHris5aOWaaabCaeaacqWG4baEdaWgaaWcbaGaemyAaKgabeaaaeaacqWGPbqAcqGH9aqpcqaIXaqmaeaacqWGTbqBa0GaeyyeIuoakiaaxMaacaWLjaWaaeWaaeaacqaIYaGmaiaawIcacaGLPaaaaaa@65F6@

The "**E**ST **C**overage **R**atio of a **G**O Term" (**ECRG**) is defined as the RECLG ratio of the two transcriptomes in the study.

ECRGGO−Id=(RECLGGO−Id|set2)/(RECLGGO−Id|set1)=(∑i=1n2xi/∑i=1m2xi)/(∑i=1n1xi/∑i=1m1xi)     (3)
 MathType@MTEF@5@5@+=feaafiart1ev1aaatCvAUfKttLearuWrP9MDH5MBPbIqV92AaeXatLxBI9gBaebbnrfifHhDYfgasaacH8akY=wiFfYdH8Gipec8Eeeu0xXdbba9frFj0=OqFfea0dXdd9vqai=hGuQ8kuc9pgc9s8qqaq=dirpe0xb9q8qiLsFr0=vr0=vr0dc8meaabaqaciaacaGaaeqabaqabeGadaaakeaacqWGfbqrcqWGdbWqcqWGsbGucqWGhbWrdaWgaaWcbaGaem4raCKaem4ta8KaeyOeI0IaemysaKKaemizaqgabeaakiabg2da9iabcIcaOiabdkfasjabdweafjabdoeadjabdYeamjabdEeahnaaBaaaleaacqWGhbWrcqWGpbWtcqGHsislcqWGjbqscqWGKbazcqGG8baFcqWGZbWCcqWGLbqzcqWG0baDcqaIYaGmaeqaaOGaeiykaKIaei4la8IaeiikaGIaemOuaiLaemyrauKaem4qamKaemitaWKaem4raC0aaSbaaSqaaiabdEeahjabd+eapjabgkHiTiabdMeajjabdsgaKjabcYha8jabdohaZjabdwgaLjabdsha0jabigdaXaqabaGccqGGPaqkcqGH9aqpcqGGOaakdaaeWbqaaiabdIha4naaBaaaleaacqWGPbqAaeqaaOGaei4la8YaaabCaeaacqWG4baEdaWgaaWcbaGaemyAaKgabeaaaeaacqWGPbqAcqGH9aqpcqaIXaqmaeaacqWGTbqBcqaIYaGma0GaeyyeIuoakiabcMcaPiabc+caViabcIcaOmaaqahabaGaemiEaG3aaSbaaSqaaiabdMgaPbqabaaabaGaemyAaKMaeyypa0JaeGymaedabaGaemOBa4MaeGymaedaniabggHiLdGccqGGVaWldaaeWbqaaiabdIha4naaBaaaleaacqWGPbqAaeqaaaqaaiabdMgaPjabg2da9iabigdaXaqaaiabd2gaTjabigdaXaqdcqGHris5aOGaeiykaKIaaCzcaiaaxMaadaqadaqaaiabiodaZaGaayjkaiaawMcaaaWcbaGaemyAaKMaeyypa0JaeGymaedabaGaemOBa4MaeGOmaidaniabggHiLdaaaa@96A3@

Here we first calculate the ECLG before calculating the ratio, which is an inversion of the calculating steps in other GHE approaches like GO-Mapper. This feature is suitable for mining the possible differentially expressed genes represented by low abundance ESTs, which are prone to be either omitted or over-exploited if GO-Mapper approach is directly adopted. GO-Mapper probably performs well in microarray analysis, but it is not sensitive or accurate enough when apply to EST analysis, in which a great portion of "N vs. 0" (N <= 5) and other low abundance tags exist. Under this condition, the GO-Mapper approach would average the insignificant but "highly" ratio-ed genes (for example, 1/0 is infinitive in mathematical calculation, but is not a significant differentiation for gene expression) with other significantly ratio-ed genes (e.g. "1000 vs. 6") of the same GO term, yielding high false positives. On the opposite, if those insignificant but "highly" ratio-ed genes were pre-filtered by the users, a great information loss would occur.

To analyze whether the GO terms are significantly differentially represented between the two transcriptomes in the study, a 2 by 2 contingency table will be constructed for Chi-square test. If the Chi-square test does not meet the empirical criterion, Fisher's Exact test will be used instead. These tests reveal differentially represented GO terms between two GO Profiles. However, additional measures are necessary in order to calculate the global similarity or dissimilarity between the two transcriptomes of interest. To address this, Pearson correlation coefficient is calculated between the two GO profiles to report the global similarities.

Since all the GO terms are sampled during the analysis, the potential issues with multiple testing should be addressed. Within the GO-Diff algorithm, the linear step-up procedure [42] is adopted to adjust the False Discovery Rate (FDR). The algorithm can be fine tuned through parameters including the FDR cut-off defaulting at 0.1, the EST coverage ratio cut-off defaulting at 3, and unwanted GO associations can be excluded by their evidence codes.

## Results

Exhausting EST sequencing projects provide a vast repository of EST information, which can be an alternative resource for gene expression analysis across different biological systems. GO-Diff is the first software to integrate EST-based expression profiles with the GO knowledge database to achieve functional differentiation analysis between transcriptomes. Three comparative transcriptomics analyses were performed to demonstrate GO-Diff's data mining utilities and software processing capabilities. GO-Diff results were studied and characterized against existing biological knowledge for validation analysis where possible.

### Functional differences between mouse oocyte and preimplantation embryos – intra-species comparative transcritpomics

To study the functional differences among transcriptomes from the same species, we applied GO-Diff to analyze dbEST libraries of mouse-unfertilized eggs and different developmentally staged mouse preimplantation embryos.

Using GO-Diff, four different embryonic staged libraries were pooled and compared to that of unfertilized eggs in order to reveal transcriptome dynamics and extract functional and developmental perspectives between oocytes and early embryos. In this study, 121 differentially represented GO terms were revealed under the criteria of a false discovery rate at 0.1 and at least 1.5-fold of the EST coverage ratio. Results are summarized in Table 1 and details can be found in Additional file 1 and 2.

**Table 1:** GO terms revealed by comparing mouse oocyte and preimplantation embryonic transcriptomes F: molecular function. P: biological process. C: cellular component

GO category	GO name space	EST Coverage Ratio (embryos/oocyte)	Corrected P_value
Protein biosynthesis	P	3.0	1.3E-17
Ribosome	C	4.9	9.4E-15
Ribonucleoprotein complex	C	3.2	3.6E-14
Signal transduction	P	0.61	5.0E-07
Cytosolic ribosome (sensu Eukarya)	C	5.3	7.3E-06
M phase of meiotic cell cycle	P	0.32	4.8E-05
Translational elongation	P	7.0	1.0E-04
DNA replication and chromosome cycle	P	0.072	2.1E-04
DNA topoisomerase type I activity	F	0	3.8E-04
Ubiquitin cycle	P	0.63	5.6E-04
Ubiquitin conjugating enzyme activity	F	0.38	8.3E-04
Fertilization (sensu Metazoa)	P	21	1.9E-03
Homologous chromosome segregation	P	0.14	3.4E-03
Ubiquitin-protein ligase activity	F	0.53	3.4E-03
Ribosome biogenesis and assembly	P	2.5	4.2E-03
DNA topological change	P	0.057	5.2E-03
Lyase activity	F	18	6.3E-03
Response to DNA damage stimulus	P	0.50	1.8E-02
Chromatin remodeling	P	0.35	2.0E-02
Amine biosynthesis	P	15	2.7E-02
Transcriptional repressor complex	C	0.10	4.5E-02
Chromatin remodeling complex	C	0.25	4.6E-02
Induction of apoptosis via death domain Receptors	P	0.32	5.6E-02
Translation elongation factor activity	F	2.8	5.9E-02
Apical junction complex	C	0.27	6.3E-02
Lysosome	C	3.6	6.5E-02
DNA repair	P	0.52	6.8E-02
Sodium ion homeostasis	P	0.079	6.8E-02
Lung development	P	0.13	7.6E-02
Chaperone cofactor dependent protein folding	P	12	8.5E-02

The findings by GO-Diff agreed well with previous studies. The absolute rate of protein synthesis increased during preimplantation development from the oocyte to 8-cell stage [43], and this biological process was quantitated to be up-regulated 3-fold in this study. Cellular components involved with protein synthesis, e.g. the ribosome-related GO categories, were simultaneously enriched, also consistent with previous findings [44]. Profiling during preimplanatation mouse development, our GO-Diff analyses and a recent microarray study [45] using the EASE program [21] were very consistent. Our analyses also confirmed assumptions or observations that had not been fully investigated, therefore providing some clues for new discoveries. As shown in Table 2 and comprehensive details Additional file 2, GO-Diff revealed the enrichment of transcripts encoding Cathepsins locating at lysosome during development, indicating active protein degradation in mouse preimplantation embryos [46-48].

**Table 2:** Cellular component "lysosome" – GO-Diff analysis of dbEST libraries of unfertilized egg and embryos

Unigene	GO Term EST Coverage (Unfertilized egg/embryos)	GO_description	GO_ID	Type
Mm.236553 Cathepsin B	1/11	cathepsin B activity	GO:0004213	F
		peroxidase activity	GO:0004601	F
		extracellular space	GO:0005615	C
		mitochondrion	GO:0005739	C
		lysosome	GO:0005764	C
		proteolysis and peptidolysis	GO:0006508	P
		protein targeting	GO:0006605	P

Mm.322945 Cathepsin C	0/5	cysteine-type endopeptidase activity	GO:0004197	F
		dipeptidyl-peptidase I activity	GO:0004214	F
		extracellular space	GO:0005615	C
		lysosome	GO:0005764	C
		proteolysis and peptidolysis	GO:0006508	P

Mm.231395 Cathepsin D	0/15	cathepsin D activity	GO:0004192	F
		pepsin A activity	GO:0004194	F
		mitochondrion	GO:0005739	C
		lysosome	GO:0005764	C
		proteolysis and peptidolysis	GO:0006508	P

Mm.930 Cathepsin L	0/20	cathepsin L activity	GO:0004217	F
		extracellular space	GO:0005615	C
		lysosome	GO:0005764	C
		proteolysis and peptidolysis	GO:0006508	P
		antinidain activity	GO:0008129	F

### Representation of "DNA damage response" related GO terms in mouse oocyte and preimplantation embryos – meta-analysis to test a specific hypothesis

Supported by our GO-Diff results, the recent microarray study of the preimplantation embryos [45] observed the over-representation of transcripts involved in DNA damage response and DNA repair in oocytes in comparison to that in the preimplantation stages, and suggested this over-representation reflected the oocyte's possible response to selective pressures such that genomic integrity could be ensured. However, the over-representation could very well be data analysis artifacts as both over-representation in oocytes and under-representation of those transcripts in embryonic cells could, on the surface, yield similar over-representation analysis results. Under this circumstance, comparisons with other tissues could provide some additional evidences and even definitive answers. With the GO-Diff knowledge base integrating various types of dbEST libraries, this kind of analysis is straightforward. Pairwise comparative analyses of dbEST libraries of eight other tissues with those of oocyte and preimplantation embryos yielded many differentially represented GO terms related to DNA damage response (Table 3). With these cross-tissue examinations, we observed transcripts associated with such processes were indeed highly represented in both the preimplantation embryos and the oocytes. With a number of transcriptomes as references, oocytes had more pronounced transcriptions under those GO terms compared to all samples analyzed including embryos as well, leading to the conclusion that oocytes have more represented GO terms in the area of DNA damage response and DNA repair.

**Table 3:** "DNA damage response" related GO terms that are differentially represented between oocyte and preimplantation with respect with common reference tissues. Pairwise comparisons of oocyte and preimplantation dbEST libraries to eight reference libraries revealed five GO terms related to the biological process of "DNA damage response": GO:0006974 ("response to DNA damage stimulus"), GO:0042770 ("DNA damage response, signal transduction"), GO:0000077 ("DNA damage checkpoint"), GO:0003684 ("damaged DNA binding") and GO:0006281 ("DNA repair"). GO terms with EST Coverage Ratio >= 1.5 or <= 1/1.5 and with corrected P_value of 0.1 were selected.

	GO ID	Oocyte (dbEST 1389)		2-cell to 16-cell Embryos (dbEST 1381,1382,1524,1532)	
		
		EST Coverage Ratio (Other tissue/Oocyte)	Corrected *P*_value	EST Coverage Ratio (Other tissue/Embryos)	Corrected *P*_value
Liver (dbEST 1299)	GO:0006974	9.0E-02	2.5E-38	0.15	7.9E-29
	GO:0042770	5.6E-02	3.2E-04	6.5E-02	4.3E-05
	GO:0000077	1.4E-02	1.4E-05	3.8E-02	7.5E-05
	GO:0003684	1.2E-04	2.0E-02	5.0E-02	3.4E-07
	GO:0006281	9.7E-02	1.4E-30	0.19	4.0E-20

Brain (dbEST 1469)	GO:0006974	0.29	4.8E-06	0.58	6.4E-02
	GO:0042770	0.12	5.6E-02	-	-
	GO:0000077	0.12	5.6E-02	-	-
	GO:0003684	-	-	-	-
	GO:0006281	0.30	6.5E-05	-	-

Kidney (dbEST 1764)	GO:0006974	0.30	6.4E-09	0.35	1.0E-04
	GO:0042770	0.0	7.0E-03	-	-
	GO:0000077	0.0	7.0E-03	-	-
	GO:0003684	-		0.0	7.0E-02
	GO:0006281	0.21	1.4E-06	0.40	9.1E-03

Placenta (dbEST 1783)	GO:0006974	6.2E-02	1.6E-15	0.12	9.1E-08
	GO:0042770	6.6E-02	1.6E-02	-	-
	GO:0000077	0.0	3.8E-03	0	9.9E-02
	GO:0003684	0.0	8.7E-02	0.0	4.7E-02
	GO:0006281	3.7E-02	1.7E-14	7.1E-02	1.0E-07

Skin (dbEST 498)	GO:0006974	0.33	1.1E-07	0.65	2.7E-02
	GO:0042770	-	-	-	-
	GO:0000077	-	-	-	-
	GO:0003684	-	-	-	-
	GO:0006281	0.28	4.7E-08	0.55	2.5E-03

Testis (dbEST 483)	GO:0006974	0.28	1.4E-05	0.57	9.0E-02
	GO:0042770	6.8E-02	4.1E-02	-	-
	GO:0000077	6.8E-02	4.1E-02	-	-
	GO:0003684		-	-	-
	GO:0006281	0.33	4.6E-04	-	-

Heart (dbEST 509)	GO:0006974	0.33	1.4E-06	0.65	7.6E-02
	GO:0042770				-
	GO:0000077	0.14	3.7E-02		-
	GO:0003684				-
	GO:0006281	0.34	3.1E-05		-

Lung (dbEST 526)	GO:0006974	0.24	2.2E-06	0.48	2.3E-02
	GO:0042770	-	-	-	-
	GO:0000077	-	-	-	-
	GO:0003684	-	-	-	-
	GO:0006281	0.21	5.2E-06	0.40	1.4E-02

### Preliminary characterization of functional differences between human and mouse liver – inter-species comparative transcritpomics

It is interesting to explore how transcriptome variations are related with physiological differences between species. Comparing transcriptomes from a functional perspective may help explore physiological diversities. In this study, we explored the functional differences of liver transcriptomes between human and mouse. Inter-species transcriptome comparison is not as straightforward as intra-species comparison due to the unequal GO annotation coverage between species. To reduce false positives caused by biased GO annotation, we incorporated multiple GO-Diff results into meta-analysis using both GO associations in the background database and from BLAST search. Following this strategy, we compared a series of dbEST libraries of human and mouse liver as shown in Table 4. 261 GO terms were found differentially represented between human and mouse in the liver (Additional file 3).

**Table 4:** The pairwise comparative analysis of the relevant mouse and human dbEST libraries

			Human			
			
			Adult	Fetal		
			
			dbEST 252	dbEST 13052	dbEST 1365	dbEST 13859
Mouse	Adult	dbEST 1299	+	-	-	-
		dbEST 1778	+	-	-	-
			
	Fetal	dbEST 1883	-	+	+	+

Currently, comparative transcriptomic analyses between mouse and human are challenging both experimentally and statistically. Therefore it is difficult to validate GO-Diff's results in this regard. Nevertheless, the current results may provide some evidence relating to the physiological divergence between human and mouse. In liver, the GO categories related with aerobic metabolism are represented in higher levels in the mouse, such as "mitochondrion", "hemoglobin complex", "proton-transporting ATP synthase complex (sensu Eukarya)", "ATP synthesis coupled proton transport", "oxidoreductase activity, acting on peroxide as acceptor" and "oxygen transporter activity" ... These results may simply reflect the faster metabolic rate in mouse due to the body mass effect. Our findings may provide a gene expression perspective to explore relationships between body mass and standard metabolic rate.

### Quantitative and qualitative estimation of GO-Diff performance

Neither quantitative benchmark data sets nor other similar tools are currently available to accurately evaluate GO-Diff performance. We selected 16 unbiased EST libraries from human and mouse brain and liver, and ran GO-Diff to determine the consistency and reproducibility of the GO-Diff algorithm results (for detailed method see the Additional file 4). Table 5 and 6 show the match-up schemes of the pairwise transcriptome comparisons, and Table 7 shows the consistencies of the comparison results within and cross-species. Due to the fact that small EST libraries contain fewer genes, a portion of differentially represented GO terms identified in large-library comparisons may not be detected in smaller-library comparisons. This may reduce the observed consistency between the small library comparisons. To reduce this artifact, results from low-volume library comparisons were paired solely to that of the largest library comparison of that group to perform evaluation as shown in Table 7.

**Table 5:** Human EST libraries and their match-up grid in consistency test of GO-Diff Libraries are listed in their Ids, and each pairwise comparison is numbered. The evaluation criteria are also shown.

Human (ECRG>4 or <0.25 P < = 0.01)		Liver		
		
		Lib.9631	Lib.6533	Lib.5601
Brain	Lib.6812	(1)	(8)	(15)
	Lib.742	(2)	(9)	(16)
	Lib.13053	(3)	(10)	(17)
	Lib.2	(4)	(11)	(18)
	Lib.2297	(5)	(12)	(19)
	Lib.6811	(6)	(13)	(20)
	Lib.6682	(7)	(14)	(21)

**Table 6:** Mouse EST libraries and their match-up grid in consistency test of GO-Diff

Mouse (ECRG>4 or <0.25 P < = 0.01)		Liver	
		
		Lib.7065	Lib.1299
Brain	Lib.11268	(22)	(26)
	Lib.10522	(23)	(27)
	Lib.9978	(24)	(28)
	Lib.12357	(25)	(29)

**Table 7:** Consistency evaluation of GO-Diff results from the above comparisons in pairs listed GO terms identified as differentially represented in both sides are listed as 'Identical', those in the same GO paths are listed as 'Parent-Child', and those appeared in only one side are listed as 'Different'.Consistency rate is calculated by ('Identical'+'Parent-Child')/('Identical'+'Parent-Child'+'Different').

	Pairs	Identical	Parent-Child	Different	Consistency Rate
Human vs. Human	(1)-(7)	129	23	158	49.03%
	(2)-(7)	115	14	82	61.14%
	(3)-(7)	117	21	175	44.09%
	(4)-(7)	151	29	91	66.42%
	(5)-(7)	53	14	44	60.36%
	(6)-(7)	113	11	93	57.14%
	(8)-(14)	146	15	136	54.21%
	(9)-(14)	192	25	128	62.90%
	(10)-(14)	169	36	205	50.00%
	(11)-(14)	194	36	101	69.49%
	(12)-(14)	93	12	55	65.63%
	(13)-(14)	128	10	76	64.49%
	(15)-(21)	182	33	127	62.87%
	(16)-(21)	226	42	119	69.25%
	(17)-(21)	197	61	240	51.81%
	(18)-(21)	223	40	64	80.43%
	(19)-(21)	115	13	101	55.90%
	(20)-(21)	150	17	69	70.76%

Mouse vs. Mouse	(22)-(25)	359	122	347	58.09%
	(23)-(25)	187	25	80	72.60%
	(24)-(25)	280	72	158	69.02%
	(26)-(29)	371	93	262	63.91%
	(27)-(29)	267	29	83	78.10%
	(28)-(29)	347	60	145	73.73%

Human vs. Mouse	(1)-(25)	78	64	168	45.81%
	(2)-(25)	66	27	118	44.08%
	(3)-(25)	60	53	200	36.10%
	(4)-(25)	79	50	142	47.60%
	(5)-(25)	21	11	79	28.83%
	(6)-(25)	52	34	131	39.63%
	(8)-(25)	103	58	136	54.21%
	(9)-(25)	132	48	165	52.17%
	(10)-(25)	116	73	221	46.10%
	(11)-(25)	125	54	152	54.08%
	(12)-(25)	57	17	86	46.25%
	(13)-(25)	76	27	111	48.13%
	(15)-(25)	96	65	181	47.08%
	(16)-(25)	136	57	194	49.87%
	(17)-(25)	112	94	292	41.37%
	(18)-(25)	121	56	150	54.13%
	(19)-(25)	59	25	145	36.68%
	(20)-(25)	88	34	134	47.66%
	(1)-(29)	76	63	171	44.84%
	(2)-(29)	69	39	103	51.18%
	(3)-(29)	64	51	198	36.74%
	(4)-(29)	77	48	146	46.13%
	(5)-(29)	19	16	76	31.53%
	(6)-(29)	63	25	129	40.55%
	(8)-(29)	108	68	121	59.26%
	(9)-(29)	144	53	148	57.10%
	(10)-(29)	118	78	214	47.80%
	(11)-(29)	131	66	134	59.52%
	(12)-(29)	53	31	76	52.50%
	(13)-(29)	79	34	101	52.80%
	(15)-(29)	113	67	162	52.63%
	(16)-(29)	153	54	180	53.49%
	(17)-(29)	120	98	280	43.78%
	(18)-(29)	135	48	144	55.96%
	(19)-(29)	68	29	132	42.36%
	(20)-(29)	94	27	135	47.27%

The average consistencies of human-human, mouse-mouse and human-mouse comparisons were 60.9%, 69.2% and 47.1% respectively. Recent studies showed that 18%-94% of genes could be differentially expressed among individuals of the same species [49-51], which adversely affected the results of consistency test. Detailed discussion of intra- and inter- species expression variations falls out of the scope of the current work. Nevertheless, even in the contexts of the high intra-species variation and even higher variation between species, GO-Diff can generate repeatable and reliable results.

## Discussion

GO-Diff is a knowledge-based data mining method, and its implementation analyzes EST transcription maps from a functional perspective upon biological domain knowledge encapsulated by GO terms. As in our case study analyses of mouse preimplantation development and human/mouse liver comparison, GO-Diff revealed many differentially represented GO categories, some of which were consistent with previous findings, others could be suggestive for future follow-up studies.

When exploring biological mechanisms of non-model organisms or un-profiled tissues, EST analysis is usually the first step to systematically study gene constitutions and gene expression. Given that GO terms are coined to be species independent, GO-Diff can facilitate the comparisons of the transcriptomes of new species according to molecular function, biological process and cellular components. In addition, the GO-Diff framework has the capability to quickly establish the analysis process to allow whole-transcriptome comparative analysis between the transcriptome of interest against a large repository of pre-sorted transcriptomes, which span different species or different tissue origins within the knowledge base. Recently, it has been suggested that many tissue-specific differences in gene expression are unique only to one population and thus are unlikely to contribute to fundamental differences between tissue types [52]. In this regard, the GO-Diff approach does offer the benefit of quickly constructing several transcriptomes of the same type and allow global analysis of different populations of the same tissue. The comparative analysis of these transcriptomes against various reference transcriptomes can weed out those population specific sampling artifacts. This kind of analyses would be difficult to perform across different platforms when conventional microarray or SAGE technologies are utilized if multiple transcriptomes are profiled and analyzed simultaneously and comparatively.

EST sequencing is not as high throughput as array technology. Based on Fisher's Exact test, we listed in Table 8 the tag counts required to achieve 95% of confidence in determining differential expression. In gene-based differential expression analysis, the number in the table is the tag count of a given gene, and in GO-based analysis, it is the ECLG. For libraries containing a few thousand tags, a tag count ratio of at least 0 vs. 6 is required to be a differential expression. The criterion is even more restrictive when multiple testing is taken into account, therefore, only a few highly expressed genes in the libraries can be evaluated, rendering the GO over-representation analysis unrealistic. GO-Diff attempts to solve this problem with the following features: it incorporates the entire body of the expression information; optionally combines multiple libraries of same kind; and lastly, adds up the tag counts of the same GO term before calculating the ratio – the EST Coverage Ratio of a GO term (ECRG), instead of averaging the expression ratios of a GO term, making it more sensitive and accurate in detection of differential GO terms represented by low abundant ESTs.

**Table 8:** The minimum tags in the compared libraries required for a 'significant' evaluation based on the Fisher's Exact test calculation The number of tags on the top of each column indicates the total number of ESTs in each library.

1000 tags			3000 tags			10000 tags			50000 tags		
Lib 1	Lib 2	P_value	Lib 1	Lib 2	P_value	Lib 1	Lib 2	P_value	Lib 1	Lib 2	P_value

0	6	0.031	0	6	0.031	0	6	0.031	0	6	0.031
1	8	0.039	1	8	0.039	1	8	0.039	1	8	0.039
2	10	0.038	2	10	0.038	2	10	0.039	2	10	0.039
3	12	0.034	3	12	0.035	3	12	0.035	3	12	0.035
4	13	0.048	4	13	0.049	4	13	0.049	4	13	0.049
5	15	0.040	5	15	0.041	5	15	0.041	5	15	0.041
6	17	0.033	6	17	0.034	6	17	0.035	6	17	0.035
7	18	0.042	7	18	0.043	7	18	0.043	7	18	0.043
8	20	0.034	8	20	0.035	8	20	0.035	8	20	0.036
9	21	0.041	9	21	0.042	9	21	0.043	9	21	0.043

It is common that genes may play multiple biological roles in different tissues or different species. This may become the source of false positives where some physiologically irrelevant GO terms will make into the final analysis report. For example, the Unigene cluster Mm.5098 is a component of transcriptional repressor complex (GO:0017053) and also plays a role in lung development (GO:0030324). In the case study of oocyte and preimplantation embryo comparative analysis, both of the GO terms were found to be differentially represented. Obviously, "role in lung development" is a false positive result in this context. This phenomenon appears more frequently when a highly expressed gene dominates several GO terms. GO-Diff tries to address these issues by providing detailed information of the significant GO terms for manual verification and following analysis. First, the expression levels of Unigene clusters associated with the significant GO terms are displayed, which allows the researchers to find significant GO terms that may have been dominated by the same Unigene cluster. Once identified, those GO terms, which are dominated by spurious gene expression artifacts and are obviously irrelevant to the particular research focus, can be excluded. Second, GO-Diff produces additional html-formatted outputs with links to AmiGO [53] and the NCBI Unigene database to gather relevant information for additional analysis. Third, the user graphical interface facilitates the interactive usage of the program. In this regard, GO-Diff provides not only a high throughput processing method but also an iterative data analysis platform much involving the researchers.

Inter-species comparisons are essential and increasingly demanding when genomes and transcriptomes of many organisms of various evolutionary lineages are available. However, inter-species transcriptome comparisons lack a common reference set. Unlike transcriptomes of the same species, in which a set of common genes or transcripts are used as references, and the expression level of each reference sequence can be uniformly evaluated among the experimental samples, transcriptomes from different species usually do not share the same set of reference sequences, which make the comparisons methodologically more challenging. One solution is to employ a set of orthologous genes from the compared species to form a reference set as implemented in methods of [54-58] explicitly or implicitly. This approach by its design suffers from some limitations, especially in moderately related species and for EST analysis as well. In moderately related species, many orthologs are no longer in a simple one to one relation, and when alternative splicing and EST assembly errors are taken into account, a common unique-transcript set between two species becomes very difficult to establish. GO-Diff made the first attempt to utilize the GO structure as the common reference set to organize transcripts into functional groups, and perform meaningful comparisons.

The current GO-Diff implementation focuses on the leverage of the EST resources for comparative transcriptomics. However, since the GO-Diff analysis is comparing the GO term representations rather than comparing the expression directly to interpret biology, the algorithm is flexible and can be further applied to SAGE data analysis. With the rapid accumulation of different gene expression resources in the public domain, GO-Diff can have broad applications and can serve as a knowledge driven data mining platform for comparative transcriptomics analysis.

## Conclusion

GO-Diff is the first software to mine functional differentiation between any EST-based transcriptomes by integrating EST profiles with GO knowledge databases. It efficiently and effectively translates EST frequencies in transcriptomes of various tissues or the same tissue across different species into EST Coverage Ratio of GO Terms. The ratio is then tested for statistical significance to uncover differentially represented GO terms between the transcriptomes, and functional differences are thus inferred. With the rapid accumulation of different EST resources in the public domain, GO-Diff can have broad applications and can serve as a knowledge driven data mining platform for comparative transcriptomic analysis.

## Abbreviations

EST: Expressed Sequence Tag; GO: Gene Ontology; SAGE: Serial Analysis of Gene Expression; FDR: False Discovery Rate; ECLG: EST Coverage Level of a GO Term; RECLG: Relative EST Coverage Level of a GO Term; ECRG: EST Coverage Ratio of a GO Term; GHE: Evaluate GO terms utilizing Holistic Expression information

## Authors' contributions

ZC, WW and LC designed and developed the methodology. ZC programmed the software. ZC, WW and XBL carried out the transcriptome comparisons and analysis. ZC, XBL JJL and LC wrote the manuscript. JJL tested the software.

## Availability and requirements

- Project name: GO-Diff

- Project home page: 

- Operating system(s): Linux, Unix (no GUI)

- Programming language: Perl

- Other requirements: X Window System for GUI, Gtk and Perl-Gtk for x_godiff.pl

- License: GPL

- Restrictions to use by non-academics: on request

## Supplementary Material

Additional File 1 Full list of the differentially represented GO terms between transcriptomes of mouse oocyte and preimplantation embryos. Click here for file

Additional File 2 Full list of the Unigene clusters associated with the differentially presented GO terms in transcriptomes of mouse oocyte and preimplantation embryos. Description: The four columns of numbers from left to right are: tag number of the Unigene cluster and the relative abundant of Unigene cluster of the oocyte transcriptome and the preimplantation embryos respectively. Click here for file

Additional File 3 List of Meta-analysis-supported GO terms identified by GO-Diff in the comparison between human and mouse liver Description: The number in the table is the EST coverage ratio (human/mouse) of the GO term. It is represented by "inf" when the EST coverage level in mouse is zero, and by "1" when no significant (ECRG >= 3, FDR <= 0.1) differences are found in the two sets of dbEST libraries. Click here for file

Additional File 4 Procedures to evaluate GO-Diff consistency. Click here for file
