# Supplementary material for: GO-Diff: Mining functional differentiation between EST-based transcriptomes
Source: BMC Bioinformatics. 2006 Feb 16;7:72. doi: 10.1186/1471-2105-7-72 (PMC1388240; doi:10.1186/1471-2105-7-72)
Supplement: Additional File 1 — Full list of the differentially represented GO terms between transcriptomes of mouse oocyte and preimplantation embryos. [file 1471-2105-7-72-S1.pdf]

Table 1. Full list of differentially represented GO terms between transcriptomes of mouse oocyte and preimplantation embryos

GO-ID	Oocyte ECLG	Oocyte RECLG	Embryos ECLG	Embryos RECLG	ECRG(embryos/oocyte)	Raw *P* _value	Corrected *P* _value	GO description	GO name_space
GO:0009058	92	0.06424581	1211	0.167265193	2.603519097	3.13E-23	1.09E-19	biosynthesis	P
GO:0044249	92	0.06424581	1190	0.164364641	2.558371367	2.67E-22	4.65E-19	cellular biosynthesis	P
GO:0006412	61	0.042597765	931	0.12859116	3.018730187	1.45E-20	1.28E-17	protein biosynthesis	P
GO:0009059	65	0.045391061	960	0.132596685	2.92120697	1.47E-20	1.28E-17	macromolecule biosynthesis	P
GO:0005840	23	0.016061453	571	0.078867403	4.910353111	1.34E-17	9.36E-15	ribosome	C
GO:0003735	26	0.018156425	588	0.08121547	4.473098173	3.06E-17	1.78E-14	structural constituent of ribosome	F
GO:0030529	44	0.030726257	721	0.099585635	3.241059769	7.17E-17	3.57E-14	ribonucleoprotein complex	C
GO:0005198	54	0.037709497	754	0.104143646	2.761735216	4.07E-15	1.77E-12	structural molecule activity	F
GO:0000278	58	0.040502793	108	0.014917127	0.368298724	2.14E-10	7.47E-08	mitotic cell cycle	P
GO:0007165	182	0.127094972	563	0.077762431	0.611845061	1.59E-09	5.02E-07	signal transduction	P
GO:0007154	217	0.151536313	708	0.097790055	0.645324236	2.32E-09	5.79E-07	cell communication	P
GO:0043228	141	0.098463687	1161	0.160359116	1.628611732	2.67E-09	5.81E-07	non-membrane-bound organelle	C
GO:0043232	141	0.098463687	1161	0.160359116	1.628611732	2.67E-09	5.81E-07	intracellular non-membrane-bound organelle	C
GO:0005830	9	0.006284916	242	0.033425414	5.318354819	3.56E-08	7.30E-06	cytosolic ribosome (sensu Eukaryota)	C
GO:0000279	55	0.038407821	120	0.016574586	0.431541939	1.40E-07	2.60E-05	M phase	P
GO:0000087	41	0.028631285	79	0.010911602	0.381107667	3.04E-07	4.60E-05	M phase of mitotic cell cycle	P
GO:0051327	30	0.020949721	48	0.006629834	0.316464088	3.56E-07	4.77E-05	M phase of meiotic cell cycle	P
GO:0051321	30	0.020949721	48	0.006629834	0.316464088	3.56E-07	4.77E-05	meiotic cell cycle	P
GO:0007126	30	0.020949721	48	0.006629834	0.316464088	3.56E-07	4.77E-05	meiosis	P
GO:0006414	5	0.00349162	176	0.024309392	6.962209945	8.05E-07	0.000103966	translational elongation	P
GO:0000067	11	0.007681564	4	0.000552486	0.071923656	1.72E-06	0.000213634	DNA replication and chromosome cycle	P
GO:0048513	88	0.061452514	251	0.034668508	0.56415118	2.55E-06	0.000307011	organ development	P
GO:0003917	7	0.004888268	0	0	0	3.31E-06	0.000384168	DNA topoisomerase type I activity	F
GO:0006512	122	0.085195531	389	0.053729282	0.630658455	5.14E-06	0.000560238	ubiquitin cycle	P
GO:0008639	32	0.022346369	62	0.008563536	0.383218232	8.09E-06	0.000828555	small protein conjugating enzyme activity	F
GO:0009887	84	0.058659218	244	0.033701657	0.574533018	8.68E-06	0.000828555	organogenesis	P
GO:0004840	32	0.022346369	62	0.008563536	0.383218232	8.09E-06	0.000828555	ubiquitin conjugating enzyme activity	F
GO:0006259	99	0.069134078	302	0.041712707	0.603359562	8.75E-06	0.000828555	DNA metabolism	P
GO:0005615	96	0.067039106	767	0.105939227	1.580260129	8.80E-06	0.000828555	extracellular space	C
GO:0005576	104	0.072625698	811	0.112016575	1.542382065	1.15E-05	0.00105715	extracellular region	C
GO:0007166	76	0.053072626	216	0.029834254	0.562140157	1.22E-05	0.001088752	cell surface receptor linked signal transduction	P
GO:0009653	101	0.070530726	317	0.04378453	0.620786609	2.14E-05	0.00186178	morphogenesis	P
GO:0009566	1	0.000698324	106	0.014640884	20.96574586	2.27E-05	0.001865272	fertilization	P
GO:0007338	1	0.000698324	106	0.014640884	20.96574586	2.27E-05	0.001865272	fertilization (sensu Metazoa)	P
GO:0007242	99	0.069134078	310	0.04281768	0.619342597	2.40E-05	0.001899697	intracellular signaling cascade	P
GO:0007028	19	0.013268156	251	0.034668508	2.61291073	2.95E-05	0.002286359	cytoplasm organization and biogenesis	P
GO:0045143	11	0.007681564	8	0.001104972	0.143847313	4.98E-05	0.003409477	homologous chromosome segregation	P
GO:0045132	11	0.007681564	8	0.001104972	0.143847313	4.98E-05	0.003409477	meiotic chromosome segregation	P
GO:0009888	18	0.012569832	27	0.003729282	0.296685083	5.05E-05	0.003409477	histogenesis	P
GO:0016881	55	0.038407821	147	0.020303867	0.528638875	5.03E-05	0.003409477	acid-amino acid ligase activity	F
GO:0016879	58	0.040502793	158	0.021823204	0.538807392	5.09E-05	0.003409477	ligase activity, forming carbon-nitrogen bonds	F
GO:0004842	55	0.038407821	147	0.020303867	0.528638875	5.03E-05	0.003409477	ubiquitin-protein ligase activity	F
GO:0009719	41	0.028631285	98	0.013535912	0.472766474	5.32E-05	0.0035004	response to endogenous stimulus	P
GO:0042254	19	0.013268156	242	0.033425414	2.519220704	6.48E-05	0.004180773	ribosome biogenesis and assembly	P
GO:0000080	7	0.004888268	2	0.000276243	0.056511444	8.73E-05	0.005156221	G1 phase of mitotic cell cycle	P
GO:0007089	7	0.004888268	2	0.000276243	0.056511444	8.73E-05	0.005156221	traversing start control point of mitotic cell cycle	P
GO:0006265	7	0.004888268	2	0.000276243	0.056511444	8.73E-05	0.005156221	DNA topological change	P
GO:0051318	7	0.004888268	2	0.000276243	0.056511444	8.73E-05	0.005156221	G1 phase	P
GO:0003916	7	0.004888268	2	0.000276243	0.056511444	8.73E-05	0.005156221	DNA topoisomerase activity	F
GO:0016829	1	0.000698324	91	0.012569061	17.99889503	0.000111032	0.006343368	lyase activity	F
GO:0007046	19	0.013268156	235	0.032458564	2.446350683	0.000118404	0.006655437	ribosome biogenesis	P
GO:0006511	47	0.032821229	123	0.01698895	0.517620783	0.000120831	0.006684079	ubiquitin-dependent protein catabolism	P
GO:0005554	105	0.073324022	349	0.04820442	0.657416469	0.000125811	0.006850823	molecular_function unknown	F
GO:0043037	31	0.021648045	317	0.04378453	2.022562823	0.000130126	0.006976776	translation	P
GO:0016773	77	0.05377095	240	0.033149171	0.616488484	0.000197322	0.010419221	phosphotransferase activity, alcohol group as ac	F
GO:0015935	5	0.00349162	120	0.016574586	4.746961326	0.000238816	0.012239322	small ribosomal subunit	C
GO:0005096	28	0.019553073	61	0.008425414	0.430899763	0.000238678	0.012239322	GTPase activator activity	F
GO:0019941	47	0.032821229	127	0.017541436	0.534453979	0.00024768	0.012509642	modification-dependent protein catabolism	P
GO:0007127	13	0.009078212	16	0.002209945	0.243433914	0.000319887	0.0159258	meiosis I	P
GO:0007067	33	0.023044693	79	0.010911602	0.473497405	0.000333864	0.01638753	mitosis	P
GO:0006974	38	0.026536313	97	0.01339779	0.504885141	0.000381065	0.018444619	response to DNA damage stimulus	P
GO:0006338	18	0.012569832	32	0.00441989	0.351626765	0.000414121	0.019770037	chromatin remodeling	P
GO:0007346	10	0.00698324	10	0.001381215	0.197790055	0.000545093	0.025670946	regulation of mitotic cell cycle	P
GO:0051325	13	0.009078212	19	0.002624309	0.289077773	0.000577702	0.026490697	interphase	P
GO:0051329	13	0.009078212	19	0.002624309	0.289077773	0.000577702	0.026490697	interphase of mitotic cell cycle	P
GO:0030004	9	0.006284916	8	0.001104972	0.175813382	0.000609429	0.027228984	monovalent inorganic cation homeostasis	P
GO:0009309	1	0.000698324	75	0.010359116	14.83425414	0.000606327	0.027228984	amine biosynthesis	P
GO:0000045	4	0.002793296	0	0	0	0.000740925	0.032276554	autophagic vacuole formation	P
GO:0016236	4	0.002793296	0	0	0	0.000740925	0.032276554	macroautophagy	P
GO:0007059	18	0.012569832	34	0.004696133	0.373603438	0.000840709	0.03617126	chromosome segregation	P
GO:0017053	6	0.004189944	3	0.000414365	0.098895028	0.00107164	0.044995974	transcriptional repressor complex	C
GO:0016585	11	0.007681564	14	0.001933702	0.251732798	0.001119413	0.046442297	chromatin remodeling complex	C

GO:0019992	8	0.005586592	7	0.000966851	0.173066298	0.001167422	0.047307743	diacylglycerol binding	F
GO:0003700	66	0.046089385	212	0.029281768	0.635325632	0.001295205	0.051741238	transcription factor activity	F
GO:0008625	13	0.009078212	21	0.002900552	0.319507012	0.001439307	0.056109151	induction of apoptosis via death domain receptors	P
GO:0000910	43	0.030027933	123	0.01698895	0.565771553	0.001449017	0.056109151	cytokinesis	P
GO:0003746	10	0.00698324	140	0.019337017	2.769060773	0.001548776	0.059313032	translation elongation factor activity	F
GO:0043296	11	0.007681564	15	0.002071823	0.269713712	0.001653979	0.062653452	apical junction complex	C
GO:0000323	6	0.004189944	108	0.014917127	3.560220994	0.001750965	0.064916093	lytic vacuole	C
GO:0005764	6	0.004189944	108	0.014917127	3.560220994	0.001750965	0.064916093	lysosome	C
GO:0048519	67	0.046787709	219	0.030248619	0.646507793	0.001800858	0.065374883	negative regulation of biological process	P
GO:0051276	53	0.037011173	163	0.022513812	0.608297717	0.00178602	0.065374883	chromosome organization and biogenesis	P
GO:0006281	32	0.022346369	84	0.01160221	0.519198895	0.001884289	0.067698441	DNA repair	P
GO:0006883	5	0.00349162	2	0.000276243	0.079116022	0.001909519	0.067904837	sodium ion homeostasis	P
GO:0051301	45	0.031424581	133	0.018370166	0.584579497	0.002060176	0.072522369	cell division	P
GO:0030323	6	0.004189944	4	0.000552486	0.131860037	0.002305113	0.075786033	respiratory tube development	P
GO:0004672	66	0.046089385	217	0.029972376	0.650309727	0.002250236	0.075786033	protein kinase activity	F
GO:0008286	6	0.004189944	4	0.000552486	0.131860037	0.002305113	0.075786033	insulin receptor signaling pathway	P
GO:0030324	6	0.004189944	4	0.000552486	0.131860037	0.002305113	0.075786033	lung development	P
GO:0004459	7	0.004888268	6	0.000828729	0.169534333	0.002249226	0.075786033	L-lactate dehydrogenase activity	F
GO:0004457	7	0.004888268	6	0.000828729	0.169534333	0.002249226	0.075786033	lactate dehydrogenase activity	F
GO:0031090	19	0.013268156	198	0.027348066	2.061180576	0.002492481	0.081180336	organelle membrane	C
GO:0007420	9	0.006284916	11	0.001519337	0.241743401	0.002628211	0.084808465	brain development	P
GO:0001707	4	0.002793296	1	0.000138122	0.049447514	0.003216374	0.084917157	mesoderm formation	P
GO:0008135	32	0.022346369	282	0.038950276	1.743024862	0.002737093	0.084917157	translation factor activity, nucleic acid binding	F
GO:0007417	10	0.00698324	14	0.001933702	0.276906077	0.003069791	0.084917157	central nervous system development	P
GO:0004718	4	0.002793296	1	0.000138122	0.049447514	0.003216374	0.084917157	Janus kinase activity	F
GO:0001933	4	0.002793296	1	0.000138122	0.049447514	0.003216374	0.084917157	negative regulation of protein amino acid phosph	P
GO:0051209	4	0.002793296	1	0.000138122	0.049447514	0.003216374	0.084917157	release of sequestered calcium ion into cytoplasn	P
GO:0051282	4	0.002793296	1	0.000138122	0.049447514	0.003216374	0.084917157	regulation of sequestering of calcium ion	P
GO:0051084	1	0.000698324	60	0.008287293	11.86740331	0.003010184	0.084917157	posttranslational protein folding	P
GO:0007186	38	0.026536313	109	0.015055249	0.567345158	0.003044567	0.084917157	G-protein coupled receptor protein signaling path	P
GO:0000178	4	0.002793296	1	0.000138122	0.049447514	0.003216374	0.084917157	exosome (RNase complex)	C
GO:0016765	2	0.001396648	70	0.009668508	6.922651934	0.002765675	0.084917157	transferase activity, transferring alkyl or aryl (othe	F
GO:0001704	4	0.002793296	1	0.000138122	0.049447514	0.003216374	0.084917157	formation of primary germ layer	P
GO:0051208	4	0.002793296	1	0.000138122	0.049447514	0.003216374	0.084917157	sequestering of calcium ion	P
GO:0051238	4	0.002793296	1	0.000138122	0.049447514	0.003216374	0.084917157	sequestering of metal ion	P
GO:0008624	13	0.009078212	23	0.003176796	0.349936252	0.003191425	0.084917157	induction of apoptosis by extracellular signals	P
GO:0004716	4	0.002793296	1	0.000138122	0.049447514	0.003216374	0.084917157	receptor signaling protein tyrosine kinase activity	F
GO:0006468	62	0.043296089	204	0.028176796	0.650793085	0.003199325	0.084917157	protein amino acid phosphorylation	P
GO:0030292	4	0.002793296	1	0.000138122	0.049447514	0.003216374	0.084917157	protein tyrosine kinase inhibitor activity	F
GO:0051085	1	0.000698324	60	0.008287293	11.86740331	0.003010184	0.084917157	chaperone cofactor dependent protein folding	P
GO:0051283	4	0.002793296	1	0.000138122	0.049447514	0.003216374	0.084917157	negative regulation of sequestering of calcium ior	P
GO:0005732	0	0	50	0.006906077	inf	0.003047858	0.084917157	small nucleolar ribonucleoprotein complex	C
GO:0048332	4	0.002793296	1	0.000138122	0.049447514	0.003216374	0.084917157	mesoderm morphogenesis	P
GO:0005730	12	0.008379888	146	0.020165746	2.406445672	0.003294486	0.086325449	nucleolus	C
GO:0004869	11	0.007681564	17	0.002348066	0.30567554	0.003343278	0.086950182	cysteine protease inhibitor activity	F
GO:0042803	19	0.013268156	42	0.005801105	0.437220122	0.003542595	0.091451438	protein homodimerization activity	F
GO:0016481	31	0.021648045	84	0.01160221	0.535947246	0.003613022	0.0925837	negative regulation of transcription	P
GO:0018212	7	0.004888268	7	0.000966851	0.197790055	0.003861875	0.09752633	peptidyl-tyrosine modification	P
GO:0018108	7	0.004888268	7	0.000966851	0.197790055	0.003861875	0.09752633	peptidyl-tyrosine phosphorylation	P
