# Supplementary material for: GO-Diff: Mining functional differentiation between EST-based transcriptomes
Source: BMC Bioinformatics. 2006 Feb 16;7:72. doi: 10.1186/1471-2105-7-72 (PMC1388240; doi:10.1186/1471-2105-7-72)
Supplement: Additional File 2 — Full list of the Unigene clusters associated with the differentially presented GO terms in transcriptomes of mouse oocyte and preimplantation embryos. Description: The four columns of numbers from left to right are: tag number of the Unigene cluster and the relative abundant of Unigene cluster of the oocyte transcriptome and the preimplantation embryos respectively. [file 1471-2105-7-72-S2.pdf]

Table 2. Full list of the Unigene clusters associated with the differentially represented GO terms

The four columns of numbers from left to right are: tag number of the Unigene cluster and the relative abundant of Unigene cluster of the oocyte transcriptome and the pre-implantation embryos respectively.

>GO:0009058	92	0.06424581	1211	0.167265193	2.603519097	3.13E-23	1.09E-19	biosynthesis	P
Mm.290578	0	0	3	0.000318674					
Mm.302724	0	0	1	0.000106225					
Mm.209300	0	0	1	0.000106225					
Mm.371560	0	0	5	0.000531124					
Mm.4505	0	0	1	0.000106225					
Mm.230169	0	0	2	0.00021245					
Mm.26207	0	0	4	0.000424899					
Mm.22218	0	0	1	0.000106225					
Mm.23951	0	0	1	0.000106225					
Mm.216089	0	0	2	0.00021245					
Mm.280895	0	0	2	0.00021245					
Mm.313345	0	0	1	0.000106225					
Mm.1457	0	0	2	0.00021245					
Mm.234247	0	0	1	0.000106225					
Mm.28278	0	0	2	0.00021245					
Mm.276776	0	0	1	0.000106225					
Mm.196220	1	0.000502008	0	0					
Mm.3941	1	0.000502008	9	0.000956023					
Mm.185453	2	0.001004016	14	0.001487147					
Mm.227183	0	0	1	0.000106225					
Mm.351579	1	0.000502008	2	0.00021245					
Mm.126534	0	0	1	0.000106225					
Mm.28753	3	0.001506024	0	0					
Mm.34701	0	0	1	0.000106225					
Mm.341243	0	0	1	0.000106225					
Mm.18845	0	0	1	0.000106225					
Mm.273122	1	0.000502008	0	0					
Mm.251255	0	0	3	0.000318674					
Mm.293628	1	0.000502008	0	0					
Mm.289992	0	0	4	0.000424899					
Mm.271222	2	0.001004016	10	0.001062248					
Mm.261831	1	0.000502008	4	0.000424899					
Mm.288669	0	0	3	0.000318674					
Mm.289800	1	0.000502008	2	0.00021245					
Mm.303071	0	0	4	0.000424899					
Mm.27764	0	0	3	0.000318674					
Mm.5356	0	0	2	0.00021245					
Mm.256765	3	0.001506024	0	0					
Mm.22673	0	0	1	0.000106225					
Mm.22119	0	0	1	0.000106225					
Mm.213003	0	0	1	0.000106225					
Mm.1894	0	0	1	0.000106225					
Mm.105218	0	0	1	0.000106225					
Mm.41417	0	0	1	0.000106225					
Mm.329993	0	0	1	0.000106225					
Mm.257482	0	0	3	0.000318674					
Mm.336104	0	0	2	0.00021245					
Mm.181862	1	0.000502008	0	0					
Mm.6375	0	0	1	0.000106225					
Mm.29201	0	0	2	0.00021245					
Mm.12863	1	0.000502008	1	0.000106225					

Mm.21118	0	0	3	0.000318674
Mm.371950	0	0	1	0.000106225
Mm.145488	0	0	3	0.000318674
Mm.154511	3	0.001506024	7	0.000743573
Mm.100113	0	0	16	0.001699596
Mm.361980	0	0	14	0.001487147
Mm.286061	0	0	3	0.000318674
Mm.298467	0	0	1	0.000106225
Mm.371545	0	0	7	0.000743573
Mm.5286	0	0	23	0.00244317
Mm.217354	0	0	1	0.000106225
Mm.138471	0	0	1	0.000106225
Mm.360075	4	0.002008032	85	0.009029106
Mm.289431	0	0	17	0.001805821
Mm.262037	0	0	1	0.000106225
Mm.29394	0	0	1	0.000106225
Mm.2238	0	0	2	0.00021245
Mm.371557	0	0	10	0.001062248
Mm.260084	0	0	5	0.000531124
Mm.235137	0	0	2	0.00021245
Mm.329631	0	0	1	0.000106225
Mm.271674	0	0	3	0.000318674
Mm.4071	0	0	11	0.001168472
Mm.328846	0	0	1	0.000106225
Mm.197555	1	0.000502008	2	0.00021245
Mm.182628	0	0	3	0.000318674
Mm.336955	0	0	1	0.000106225
Mm.347060	1	0.000502008	4	0.000424899
Mm.349277	0	0	8	0.000849798
Mm.372072	0	0	3	0.000318674
Mm.309019	0	0	1	0.000106225
Mm.29046	0	0	2	0.00021245
Mm.316592	0	0	4	0.000424899
Mm.307846	0	0	4	0.000424899
Mm.12144	1	0.000502008	0	0
Mm.306548	0	0	1	0.000106225
Mm.300263	0	0	3	0.000318674
Mm.325521	0	0	5	0.000531124
Mm.247113	0	0	3	0.000318674
Mm.358632	0	0	1	0.000106225
Mm.104368	0	0	6	0.000637349
Mm.21529	1	0.000502008	1	0.000106225
Mm.371576	0	0	1	0.000106225
Mm.323357	0	0	4	0.000424899
Mm.354330	0	0	10	0.001062248
Mm.4419	0	0	1	0.000106225
Mm.262021	0	0	18	0.001912046
Mm.282053	0	0	14	0.001487147
Mm.290772	0	0	1	0.000106225
Mm.289868	0	0	9	0.000956023
Mm.300271	0	0	1	0.000106225
Mm.353923	0	0	6	0.000637349
Mm.43778	0	0	7	0.000743573
Mm.643	0	0	7	0.000743573
Mm.702	0	0	1	0.000106225
Mm.371577	0	0	2	0.00021245

Mm.371578	0	0	2	0.00021245
Mm.300281	0	0	1	0.000106225
Mm.35583	0	0	1	0.000106225
Mm.16775	3	0.001506024	11	0.001168472
Mm.154915	0	0	2	0.00021245
Mm.331113	0	0	11	0.001168472
Mm.6957	0	0	6	0.000637349
Mm.66	0	0	21	0.00223072
Mm.5291	2	0.001004016	17	0.001805821
Mm.325584	0	0	3	0.000318674
Mm.371579	0	0	5	0.000531124
Mm.260904	0	0	4	0.000424899
Mm.299312	0	0	1	0.000106225
Mm.13886	0	0	1	0.000106225
Mm.28839	3	0.001506024	3	0.000318674
Mm.262067	0	0	1	0.000106225
Mm.29041	0	0	1	0.000106225
Mm.328378	0	0	1	0.000106225
Mm.180458	2	0.001004016	14	0.001487147
Mm.297372	0	0	4	0.000424899
Mm.275195	0	0	2	0.00021245
Mm.312227	0	0	1	0.000106225
Mm.329353	0	0	2	0.00021245
Mm.259294	1	0.000502008	0	0
Mm.260943	0	0	3	0.000318674
Mm.52275	1	0.000502008	0	0
Mm.21105	0	0	1	0.000106225
Mm.274482	0	0	1	0.000106225
Mm.288212	1	0.000502008	2	0.00021245
Mm.22723	1	0.000502008	2	0.00021245
Mm.218851	0	0	5	0.000531124
Mm.250909	0	0	1	0.000106225
Mm.250030	0	0	11	0.001168472
Mm.30066	1	0.000502008	14	0.001487147
Mm.319719	0	0	2	0.00021245
Mm.236868	0	0	1	0.000106225
Mm.330075	1	0.000502008	11	0.001168472
Mm.196538	0	0	6	0.000637349
Mm.316362	0	0	1	0.000106225
Mm.290899	0	0	22	0.002336945
Mm.261679	0	0	6	0.000637349
Mm.29183	1	0.000502008	0	0
Mm.196607	0	0	8	0.000849798
Mm.21671	0	0	4	0.000424899
Mm.276337	0	0	5	0.000531124
Mm.219675	0	0	1	0.000106225
Mm.27816	0	0	3	0.000318674
Mm.193040	0	0	1	0.000106225
Mm.260064	0	0	7	0.000743573
Mm.11376	0	0	2	0.00021245
Mm.277891	0	0	1	0.000106225
Mm.250874	0	0	2	0.00021245
Mm.3955	1	0.000502008	5	0.000531124
Mm.2718	0	0	11	0.001168472
Mm.3158	0	0	5	0.000531124
Mm.133851	0	0	1	0.000106225

Mm.22776	1	0.000502008	4	0.000424899
Mm.19355	0	0	1	0.000106225
Mm.27796	0	0	1	0.000106225
Mm.313236	0	0	1	0.000106225
Mm.140380	0	0	5	0.000531124
Mm.155033	0	0	1	0.000106225
Mm.218533	0	0	4	0.000424899
Mm.294623	0	0	1	0.000106225
Mm.249809	0	0	2	0.00021245
Mm.29900	0	0	2	0.00021245
Mm.195628	0	0	1	0.000106225
Mm.28291	2	0.001004016	1	0.000106225
Mm.23825	0	0	2	0.00021245
Mm.295618	0	0	4	0.000424899
Mm.2050	0	0	5	0.000531124
Mm.350080	0	0	9	0.000956023
Mm.289669	0	0	1	0.000106225
Mm.345333	0	0	1	0.000106225
Mm.292517	0	0	2	0.00021245
Mm.371622	1	0.000502008	1	0.000106225
Mm.289810	0	0	12	0.001274697
Mm.371625	0	0	6	0.000637349
Mm.373568	2	0.001004016	11	0.001168472
Mm.45171	0	0	1	0.000106225
Mm.21938	0	0	4	0.000424899
Mm.238817	0	0	2	0.00021245
Mm.280083	1	0.000502008	15	0.001593372
Mm.290786	0	0	11	0.001168472
Mm.339491	0	0	8	0.000849798
Mm.21724	0	0	2	0.00021245
Mm.107869	0	0	3	0.000318674
Mm.140568	0	0	5	0.000531124
Mm.236795	0	0	1	0.000106225
Mm.287443	0	0	1	0.000106225
Mm.146283	0	0	1	0.000106225
Mm.27871	0	0	2	0.00021245
Mm.70690	0	0	6	0.000637349
Mm.316754	0	0	1	0.000106225
Mm.268369	0	0	2	0.00021245
Mm.29714	0	0	1	0.000106225
Mm.288960	0	0	1	0.000106225
Mm.180873	0	0	6	0.000637349
Mm.371658	0	0	11	0.001168472
Mm.13944	0	0	19	0.002018271
Mm.199698	1	0.000502008	2	0.00021245
Mm.218515	1	0.000502008	0	0
Mm.196544	0	0	1	0.000106225
Mm.34951	0	0	1	0.000106225
Mm.331142	0	0	3	0.000318674
Mm.188544	0	0	1	0.000106225
Mm.319038	0	0	2	0.00021245
Mm.2863	0	0	1	0.000106225
Mm.248334	0	0	1	0.000106225
Mm.4954	0	0	2	0.00021245
Mm.258094	2	0.001004016	1	0.000106225
Mm.11132	0	0	1	0.000106225

Mm.173119	0	0	1	0.000106225
Mm.154783	0	0	1	0.000106225
Mm.21596	0	0	1	0.000106225
Mm.18353	0	0	1	0.000106225
Mm.3196	0	0	2	0.00021245
Mm.214593	0	0	1	0.000106225
Mm.7236	0	0	3	0.000318674
Mm.259191	0	0	2	0.00021245
Mm.271670	0	0	1	0.000106225
Mm.281298	0	0	1	0.000106225
Mm.11827	1	0.000502008	0	0
Mm.210305	0	0	2	0.00021245
Mm.65021	0	0	7	0.000743573
Mm.272427	0	0	2	0.00021245
Mm.32889	0	0	1	0.000106225
Mm.28549	0	0	1	0.000106225
Mm.280768	0	0	1	0.000106225
Mm.22147	0	0	2	0.00021245
Mm.341719	1	0.000502008	7	0.000743573
Mm.274904	0	0	1	0.000106225
Mm.22363	0	0	1	0.000106225
Mm.486	0	0	4	0.000424899
Mm.27435	0	0	1	0.000106225
Mm.26379	2	0.001004016	0	0
Mm.19893	0	0	2	0.00021245
Mm.333916	1	0.000502008	1	0.000106225
Mm.15622	0	0	2	0.00021245
Mm.202561	1	0.000502008	1	0.000106225
Mm.233534	0	0	4	0.000424899
Mm.29357	0	0	4	0.000424899
Mm.641	0	0	1	0.000106225
Mm.289741	0	0	1	0.000106225
Mm.3711	0	0	4	0.000424899
Mm.4222	0	0	3	0.000318674
Mm.18939	0	0	1	0.000106225
Mm.22374	0	0	1	0.000106225
Mm.29703	1	0.000502008	0	0
Mm.7013	0	0	2	0.00021245
Mm.287178	0	0	5	0.000531124
Mm.250332	0	0	1	0.000106225
Mm.268395	0	0	3	0.000318674
Mm.121549	0	0	1	0.000106225
Mm.29584	0	0	1	0.000106225
Mm.38151	0	0	2	0.00021245
Mm.338021	1	0.000502008	0	0
Mm.299381	1	0.000502008	5	0.000531124
Mm.6065	0	0	7	0.000743573
Mm.1260	0	0	6	0.000637349
Mm.275430	0	0	2	0.00021245
Mm.27082	5	0.00251004	0	0
Mm.276137	1	0.000502008	9	0.000956023
Mm.238973	2	0.001004016	21	0.00223072
Mm.12677	0	0	2	0.00021245
Mm.217787	1	0.000502008	2	0.00021245
Mm.249096	0	0	1	0.000106225
Mm.17708	0	0	2	0.00021245

Mm.29045	0	0	1	0.000106225					
Mm.30155	1	0.000502008	3	0.000318674					
Mm.41	0	0	7	0.000743573					
Mm.133551	0	0	1	0.000106225					
Mm.278560	0	0	1	0.000106225					
Mm.311549	0	0	4	0.000424899					
Mm.2966	0	0	1	0.000106225					
Mm.10314	0	0	1	0.000106225					
Mm.371641	0	0	3	0.000318674					
Mm.1815	0	0	1	0.000106225					
Mm.305535	0	0	4	0.000424899					
Mm.310036	0	0	2	0.00021245					
Mm.24738	1	0.000502008	2	0.00021245					
Mm.289936	0	0	14	0.001487147					
Mm.28301	0	0	2	0.00021245					
Mm.23869	0	0	1	0.000106225					
Mm.3217	0	0	3	0.000318674					
Mm.4606	0	0	2	0.00021245					
Mm.206417	0	0	3	0.000318674					
Mm.16898	0	0	3	0.000318674					
Mm.258142	0	0	1	0.000106225					
Mm.196067	0	0	2	0.00021245					
Mm.274180	0	0	1	0.000106225					
Mm.29815	0	0	12	0.001274697					
Mm.24887	0	0	2	0.00021245					
Mm.7286	0	0	1	0.000106225					
Mm.246240	1	0.000502008	2	0.00021245					
Mm.196574	0	0	1	0.000106225					
Mm.34102	0	0	19	0.002018271					
Mm.250214	0	0	2	0.00021245					
Mm.31597	0	0	3	0.000318674					
Mm.29648	2	0.001004016	7	0.000743573					
Mm.200912	0	0	2	0.00021245					
Mm.2339	0	0	1	0.000106225					
Mm.193096	1	0.000502008	7	0.000743573					
Mm.282096	0	0	1	0.000106225					
Mm.306903	0	0	2	0.00021245					
Mm.206919	0	0	1	0.000106225					
Mm.281738	1	0.000502008	1	0.000106225					
Mm.314113	0	0	2	0.00021245					
Mm.12286	0	0	1	0.000106225					
Mm.271775	1	0.000502008	1	0.000106225					
Mm.17991	0	0	1	0.000106225					
Mm.30204	2	0.001004016	0	0					
Mm.341742	0	0	1	0.000106225					
Mm.302865	2	0.001004016	1	0.000106225					
Mm.140811	2	0.001004016	0	0					
Mm.43358	0	0	4	0.000424899					
Mm.61526	0	0	5	0.000531124					
Mm.6988	0	0	1	0.000106225					
Mm.29114	0	0	3	0.000318674					
Mm.180189	0	0	1	0.000106225					
Mm.443	1	0.000502008	0	0					
Mm.255044	0	0	1	0.000106225					
>GO:0044249	92	0.06424581	1190	0.164364641	2.558371367	2.67E-22	4.65E-19	cellular biosynthesis	P
Mm.21118	0	0	3	0.000318674					

Mm.371950	0	0	1	0.000106225
Mm.145488	0	0	3	0.000318674
Mm.154511	3	0.001506024	7	0.000743573
Mm.100113	0	0	16	0.001699596
Mm.361980	0	0	14	0.001487147
Mm.286061	0	0	3	0.000318674
Mm.298467	0	0	1	0.000106225
Mm.371545	0	0	7	0.000743573
Mm.5286	0	0	23	0.00244317
Mm.217354	0	0	1	0.000106225
Mm.138471	0	0	1	0.000106225
Mm.360075	4	0.002008032	85	0.009029106
Mm.289431	0	0	17	0.001805821
Mm.262037	0	0	1	0.000106225
Mm.196220	1	0.000502008	0	0
Mm.29394	0	0	1	0.000106225
Mm.2238	0	0	2	0.00021245
Mm.371557	0	0	10	0.001062248
Mm.260084	0	0	5	0.000531124
Mm.3941	1	0.000502008	9	0.000956023
Mm.185453	2	0.001004016	14	0.001487147
Mm.235137	0	0	2	0.00021245
Mm.329631	0	0	1	0.000106225
Mm.289992	0	0	4	0.000424899
Mm.271674	0	0	3	0.000318674
Mm.4071	0	0	11	0.001168472
Mm.328846	0	0	1	0.000106225
Mm.197555	1	0.000502008	2	0.00021245
Mm.182628	0	0	3	0.000318674
Mm.336955	0	0	1	0.000106225
Mm.347060	1	0.000502008	4	0.000424899
Mm.349277	0	0	8	0.000849798
Mm.372072	0	0	3	0.000318674
Mm.309019	0	0	1	0.000106225
Mm.29046	0	0	2	0.00021245
Mm.316592	0	0	4	0.000424899
Mm.307846	0	0	4	0.000424899
Mm.12144	1	0.000502008	0	0
Mm.306548	0	0	1	0.000106225
Mm.300263	0	0	3	0.000318674
Mm.325521	0	0	5	0.000531124
Mm.247113	0	0	3	0.000318674
Mm.358632	0	0	1	0.000106225
Mm.104368	0	0	6	0.000637349
Mm.21529	1	0.000502008	1	0.000106225
Mm.371576	0	0	1	0.000106225
Mm.323357	0	0	4	0.000424899
Mm.354330	0	0	10	0.001062248
Mm.4419	0	0	1	0.000106225
Mm.262021	0	0	18	0.001912046
Mm.282053	0	0	14	0.001487147
Mm.290772	0	0	1	0.000106225
Mm.289868	0	0	9	0.000956023
Mm.300271	0	0	1	0.000106225
Mm.353923	0	0	6	0.000637349
Mm.43778	0	0	7	0.000743573

Mm.643	0	0	7	0.000743573
Mm.702	0	0	1	0.000106225
Mm.371577	0	0	2	0.00021245
Mm.371578	0	0	2	0.00021245
Mm.300281	0	0	1	0.000106225
Mm.35583	0	0	1	0.000106225
Mm.16775	3	0.001506024	11	0.001168472
Mm.154915	0	0	2	0.00021245
Mm.331113	0	0	11	0.001168472
Mm.6957	0	0	6	0.000637349
Mm.66	0	0	21	0.00223072
Mm.5291	2	0.001004016	17	0.001805821
Mm.325584	0	0	3	0.000318674
Mm.371579	0	0	5	0.000531124
Mm.260904	0	0	4	0.000424899
Mm.299312	0	0	1	0.000106225
Mm.13886	0	0	1	0.000106225
Mm.28839	3	0.001506024	3	0.000318674
Mm.262067	0	0	1	0.000106225
Mm.29041	0	0	1	0.000106225
Mm.271222	2	0.001004016	10	0.001062248
Mm.328378	0	0	1	0.000106225
Mm.180458	2	0.001004016	14	0.001487147
Mm.297372	0	0	4	0.000424899
Mm.275195	0	0	2	0.00021245
Mm.312227	0	0	1	0.000106225
Mm.329353	0	0	2	0.00021245
Mm.259294	1	0.000502008	0	0
Mm.260943	0	0	3	0.000318674
Mm.52275	1	0.000502008	0	0
Mm.21105	0	0	1	0.000106225
Mm.274482	0	0	1	0.000106225
Mm.288212	1	0.000502008	2	0.00021245
Mm.22723	1	0.000502008	2	0.00021245
Mm.218851	0	0	5	0.000531124
Mm.250909	0	0	1	0.000106225
Mm.250030	0	0	11	0.001168472
Mm.30066	1	0.000502008	14	0.001487147
Mm.227183	0	0	1	0.000106225
Mm.319719	0	0	2	0.00021245
Mm.236868	0	0	1	0.000106225
Mm.330075	1	0.000502008	11	0.001168472
Mm.196538	0	0	6	0.000637349
Mm.316362	0	0	1	0.000106225
Mm.290899	0	0	22	0.002336945
Mm.261679	0	0	6	0.000637349
Mm.29183	1	0.000502008	0	0
Mm.196607	0	0	8	0.000849798
Mm.21671	0	0	4	0.000424899
Mm.276337	0	0	5	0.000531124
Mm.219675	0	0	1	0.000106225
Mm.27816	0	0	3	0.000318674
Mm.193040	0	0	1	0.000106225
Mm.260064	0	0	7	0.000743573
Mm.11376	0	0	2	0.00021245
Mm.277891	0	0	1	0.000106225

Mm.250874	0	0	2	0.00021245
Mm.3955	1	0.000502008	5	0.000531124
Mm.2718	0	0	11	0.001168472
Mm.3158	0	0	5	0.000531124
Mm.133851	0	0	1	0.000106225
Mm.22776	1	0.000502008	4	0.000424899
Mm.19355	0	0	1	0.000106225
Mm.27796	0	0	1	0.000106225
Mm.313236	0	0	1	0.000106225
Mm.140380	0	0	5	0.000531124
Mm.155033	0	0	1	0.000106225
Mm.218533	0	0	4	0.000424899
Mm.294623	0	0	1	0.000106225
Mm.249809	0	0	2	0.00021245
Mm.29900	0	0	2	0.00021245
Mm.195628	0	0	1	0.000106225
Mm.28291	2	0.001004016	1	0.000106225
Mm.23825	0	0	2	0.00021245
Mm.295618	0	0	4	0.000424899
Mm.2050	0	0	5	0.000531124
Mm.350080	0	0	9	0.000956023
Mm.289669	0	0	1	0.000106225
Mm.345333	0	0	1	0.000106225
Mm.292517	0	0	2	0.00021245
Mm.371622	1	0.000502008	1	0.000106225
Mm.289810	0	0	12	0.001274697
Mm.371625	0	0	6	0.000637349
Mm.373568	2	0.001004016	11	0.001168472
Mm.45171	0	0	1	0.000106225
Mm.21938	0	0	4	0.000424899
Mm.238817	0	0	2	0.00021245
Mm.280083	1	0.000502008	15	0.001593372
Mm.290786	0	0	11	0.001168472
Mm.339491	0	0	8	0.000849798
Mm.21724	0	0	2	0.00021245
Mm.289800	1	0.000502008	2	0.00021245
Mm.107869	0	0	3	0.000318674
Mm.140568	0	0	5	0.000531124
Mm.236795	0	0	1	0.000106225
Mm.287443	0	0	1	0.000106225
Mm.28753	3	0.001506024	0	0
Mm.146283	0	0	1	0.000106225
Mm.27871	0	0	2	0.00021245
Mm.70690	0	0	6	0.000637349
Mm.316754	0	0	1	0.000106225
Mm.268369	0	0	2	0.00021245
Mm.29714	0	0	1	0.000106225
Mm.288960	0	0	1	0.000106225
Mm.180873	0	0	6	0.000637349
Mm.371658	0	0	11	0.001168472
Mm.13944	0	0	19	0.002018271
Mm.199698	1	0.000502008	2	0.00021245
Mm.218515	1	0.000502008	0	0
Mm.196544	0	0	1	0.000106225
Mm.34951	0	0	1	0.000106225
Mm.331142	0	0	3	0.000318674

Mm.351579	1	0.000502008	2	0.00021245
Mm.126534	0	0	1	0.000106225
Mm.34701	0	0	1	0.000106225
Mm.341243	0	0	1	0.000106225
Mm.18845	0	0	1	0.000106225
Mm.273122	1	0.000502008	0	0
Mm.251255	0	0	3	0.000318674
Mm.293628	1	0.000502008	0	0
Mm.261831	1	0.000502008	4	0.000424899
Mm.288669	0	0	3	0.000318674
Mm.303071	0	0	4	0.000424899
Mm.27764	0	0	3	0.000318674
Mm.5356	0	0	2	0.00021245
Mm.256765	3	0.001506024	0	0
Mm.22673	0	0	1	0.000106225
Mm.22119	0	0	1	0.000106225
Mm.213003	0	0	1	0.000106225
Mm.1894	0	0	1	0.000106225
Mm.105218	0	0	1	0.000106225
Mm.41417	0	0	1	0.000106225
Mm.329993	0	0	1	0.000106225
Mm.257482	0	0	3	0.000318674
Mm.336104	0	0	2	0.00021245
Mm.188544	0	0	1	0.000106225
Mm.319038	0	0	2	0.00021245
Mm.2863	0	0	1	0.000106225
Mm.248334	0	0	1	0.000106225
Mm.4954	0	0	2	0.00021245
Mm.258094	2	0.001004016	1	0.000106225
Mm.11132	0	0	1	0.000106225
Mm.173119	0	0	1	0.000106225
Mm.154783	0	0	1	0.000106225
Mm.21596	0	0	1	0.000106225
Mm.18353	0	0	1	0.000106225
Mm.3196	0	0	2	0.00021245
Mm.214593	0	0	1	0.000106225
Mm.7236	0	0	3	0.000318674
Mm.259191	0	0	2	0.00021245
Mm.271670	0	0	1	0.000106225
Mm.281298	0	0	1	0.000106225
Mm.11827	1	0.000502008	0	0
Mm.210305	0	0	2	0.00021245
Mm.65021	0	0	7	0.000743573
Mm.272427	0	0	2	0.00021245
Mm.32889	0	0	1	0.000106225
Mm.28549	0	0	1	0.000106225
Mm.280768	0	0	1	0.000106225
Mm.22147	0	0	2	0.00021245
Mm.341719	1	0.000502008	7	0.000743573
Mm.274904	0	0	1	0.000106225
Mm.22363	0	0	1	0.000106225
Mm.486	0	0	4	0.000424899
Mm.27435	0	0	1	0.000106225
Mm.26379	2	0.001004016	0	0
Mm.19893	0	0	2	0.00021245
Mm.6375	0	0	1	0.000106225

Mm.181862	1	0.000502008	0	0
Mm.29201	0	0	2	0.00021245
Mm.12863	1	0.000502008	1	0.000106225
Mm.333916	1	0.000502008	1	0.000106225
Mm.15622	0	0	2	0.00021245
Mm.202561	1	0.000502008	1	0.000106225
Mm.233534	0	0	4	0.000424899
Mm.29357	0	0	4	0.000424899
Mm.641	0	0	1	0.000106225
Mm.289741	0	0	1	0.000106225
Mm.3711	0	0	4	0.000424899
Mm.4222	0	0	3	0.000318674
Mm.18939	0	0	1	0.000106225
Mm.22374	0	0	1	0.000106225
Mm.29703	1	0.000502008	0	0
Mm.7013	0	0	2	0.00021245
Mm.26207	0	0	4	0.000424899
Mm.287178	0	0	5	0.000531124
Mm.250332	0	0	1	0.000106225
Mm.268395	0	0	3	0.000318674
Mm.121549	0	0	1	0.000106225
Mm.29584	0	0	1	0.000106225
Mm.38151	0	0	2	0.00021245
Mm.338021	1	0.000502008	0	0
Mm.4505	0	0	1	0.000106225
Mm.299381	1	0.000502008	5	0.000531124
Mm.6065	0	0	7	0.000743573
Mm.1260	0	0	6	0.000637349
Mm.275430	0	0	2	0.00021245
Mm.27082	5	0.00251004	0	0
Mm.276137	1	0.000502008	9	0.000956023
Mm.238973	2	0.001004016	21	0.00223072
Mm.12677	0	0	2	0.00021245
Mm.217787	1	0.000502008	2	0.00021245
Mm.249096	0	0	1	0.000106225
Mm.17708	0	0	2	0.00021245
Mm.29045	0	0	1	0.000106225
Mm.30155	1	0.000502008	3	0.000318674
Mm.41	0	0	7	0.000743573
Mm.133551	0	0	1	0.000106225
Mm.278560	0	0	1	0.000106225
Mm.311549	0	0	4	0.000424899
Mm.2966	0	0	1	0.000106225
Mm.10314	0	0	1	0.000106225
Mm.371641	0	0	3	0.000318674
Mm.1815	0	0	1	0.000106225
Mm.305535	0	0	4	0.000424899
Mm.310036	0	0	2	0.00021245
Mm.24738	1	0.000502008	2	0.00021245
Mm.289936	0	0	14	0.001487147
Mm.28301	0	0	2	0.00021245
Mm.23869	0	0	1	0.000106225
Mm.3217	0	0	3	0.000318674
Mm.4606	0	0	2	0.00021245
Mm.206417	0	0	3	0.000318674
Mm.16898	0	0	3	0.000318674

Mm.258142	0	0	1	0.000106225					
Mm.196067	0	0	2	0.00021245					
Mm.274180	0	0	1	0.000106225					
Mm.29815	0	0	12	0.001274697					
Mm.24887	0	0	2	0.00021245					
Mm.7286	0	0	1	0.000106225					
Mm.246240	1	0.000502008	2	0.00021245					
Mm.196574	0	0	1	0.000106225					
Mm.34102	0	0	19	0.002018271					
Mm.250214	0	0	2	0.00021245					
Mm.31597	0	0	3	0.000318674					
Mm.29648	2	0.001004016	7	0.000743573					
Mm.200912	0	0	2	0.00021245					
Mm.2339	0	0	1	0.000106225					
Mm.193096	1	0.000502008	7	0.000743573					
Mm.282096	0	0	1	0.000106225					
Mm.306903	0	0	2	0.00021245					
Mm.206919	0	0	1	0.000106225					
Mm.281738	1	0.000502008	1	0.000106225					
Mm.314113	0	0	2	0.00021245					
Mm.12286	0	0	1	0.000106225					
Mm.271775	1	0.000502008	1	0.000106225					
Mm.17991	0	0	1	0.000106225					
Mm.30204	2	0.001004016	0	0					
Mm.341742	0	0	1	0.000106225					
Mm.302865	2	0.001004016	1	0.000106225					
Mm.140811	2	0.001004016	0	0					
Mm.43358	0	0	4	0.000424899					
Mm.61526	0	0	5	0.000531124					
Mm.290578	0	0	3	0.000318674					
Mm.302724	0	0	1	0.000106225					
Mm.6988	0	0	1	0.000106225					
Mm.29114	0	0	3	0.000318674					
Mm.180189	0	0	1	0.000106225					
Mm.443	1	0.000502008	0	0					
Mm.255044	0	0	1	0.000106225					
>GO:0006412	61	0.042597765	931	0.12859116	3.018730187	1.45E-20	1.28E-17	protein biosynthesis	P
Mm.21118	0	0	3	0.000318674					
Mm.371950	0	0	1	0.000106225					
Mm.145488	0	0	3	0.000318674					
Mm.154511	3	0.001506024	7	0.000743573					
Mm.100113	0	0	16	0.001699596					
Mm.361980	0	0	14	0.001487147					
Mm.286061	0	0	3	0.000318674					
Mm.298467	0	0	1	0.000106225					
Mm.371545	0	0	7	0.000743573					
Mm.5286	0	0	23	0.00244317					
Mm.217354	0	0	1	0.000106225					
Mm.138471	0	0	1	0.000106225					
Mm.360075	4	0.002008032	85	0.009029106					
Mm.289431	0	0	17	0.001805821					
Mm.262037	0	0	1	0.000106225					
Mm.196220	1	0.000502008	0	0					
Mm.29394	0	0	1	0.000106225					
Mm.2238	0	0	2	0.00021245					
Mm.371557	0	0	10	0.001062248					

Mm.260084	0	0	5	0.000531124
Mm.3941	1	0.000502008	9	0.000956023
Mm.185453	2	0.001004016	14	0.001487147
Mm.235137	0	0	2	0.00021245
Mm.329631	0	0	1	0.000106225
Mm.289992	0	0	4	0.000424899
Mm.271674	0	0	3	0.000318674
Mm.4071	0	0	11	0.001168472
Mm.328846	0	0	1	0.000106225
Mm.197555	1	0.000502008	2	0.00021245
Mm.182628	0	0	3	0.000318674
Mm.336955	0	0	1	0.000106225
Mm.347060	1	0.000502008	4	0.000424899
Mm.349277	0	0	8	0.000849798
Mm.372072	0	0	3	0.000318674
Mm.309019	0	0	1	0.000106225
Mm.29046	0	0	2	0.00021245
Mm.316592	0	0	4	0.000424899
Mm.307846	0	0	4	0.000424899
Mm.12144	1	0.000502008	0	0
Mm.306548	0	0	1	0.000106225
Mm.300263	0	0	3	0.000318674
Mm.325521	0	0	5	0.000531124
Mm.247113	0	0	3	0.000318674
Mm.358632	0	0	1	0.000106225
Mm.104368	0	0	6	0.000637349
Mm.21529	1	0.000502008	1	0.000106225
Mm.371576	0	0	1	0.000106225
Mm.323357	0	0	4	0.000424899
Mm.354330	0	0	10	0.001062248
Mm.4419	0	0	1	0.000106225
Mm.262021	0	0	18	0.001912046
Mm.282053	0	0	14	0.001487147
Mm.290772	0	0	1	0.000106225
Mm.289868	0	0	9	0.000956023
Mm.300271	0	0	1	0.000106225
Mm.353923	0	0	6	0.000637349
Mm.43778	0	0	7	0.000743573
Mm.643	0	0	7	0.000743573
Mm.702	0	0	1	0.000106225
Mm.371577	0	0	2	0.00021245
Mm.371578	0	0	2	0.00021245
Mm.300281	0	0	1	0.000106225
Mm.35583	0	0	1	0.000106225
Mm.16775	3	0.001506024	11	0.001168472
Mm.154915	0	0	2	0.00021245
Mm.331113	0	0	11	0.001168472
Mm.6957	0	0	6	0.000637349
Mm.66	0	0	21	0.00223072
Mm.5291	2	0.001004016	17	0.001805821
Mm.325584	0	0	3	0.000318674
Mm.371579	0	0	5	0.000531124
Mm.260904	0	0	4	0.000424899
Mm.299312	0	0	1	0.000106225
Mm.13886	0	0	1	0.000106225
Mm.28839	3	0.001506024	3	0.000318674

Mm.262067	0	0	1	0.000106225
Mm.29041	0	0	1	0.000106225
Mm.271222	2	0.001004016	10	0.001062248
Mm.328378	0	0	1	0.000106225
Mm.180458	2	0.001004016	14	0.001487147
Mm.297372	0	0	4	0.000424899
Mm.275195	0	0	2	0.00021245
Mm.312227	0	0	1	0.000106225
Mm.329353	0	0	2	0.00021245
Mm.259294	1	0.000502008	0	0
Mm.260943	0	0	3	0.000318674
Mm.52275	1	0.000502008	0	0
Mm.21105	0	0	1	0.000106225
Mm.274482	0	0	1	0.000106225
Mm.288212	1	0.000502008	2	0.00021245
Mm.22723	1	0.000502008	2	0.00021245
Mm.218851	0	0	5	0.000531124
Mm.250909	0	0	1	0.000106225
Mm.250030	0	0	11	0.001168472
Mm.30066	1	0.000502008	14	0.001487147
Mm.227183	0	0	1	0.000106225
Mm.319719	0	0	2	0.00021245
Mm.236868	0	0	1	0.000106225
Mm.330075	1	0.000502008	11	0.001168472
Mm.196538	0	0	6	0.000637349
Mm.316362	0	0	1	0.000106225
Mm.290899	0	0	22	0.002336945
Mm.261679	0	0	6	0.000637349
Mm.29183	1	0.000502008	0	0
Mm.196607	0	0	8	0.000849798
Mm.21671	0	0	4	0.000424899
Mm.276337	0	0	5	0.000531124
Mm.219675	0	0	1	0.000106225
Mm.27816	0	0	3	0.000318674
Mm.193040	0	0	1	0.000106225
Mm.260064	0	0	7	0.000743573
Mm.11376	0	0	2	0.00021245
Mm.277891	0	0	1	0.000106225
Mm.250874	0	0	2	0.00021245
Mm.3955	1	0.000502008	5	0.000531124
Mm.2718	0	0	11	0.001168472
Mm.3158	0	0	5	0.000531124
Mm.133851	0	0	1	0.000106225
Mm.22776	1	0.000502008	4	0.000424899
Mm.19355	0	0	1	0.000106225
Mm.27796	0	0	1	0.000106225
Mm.313236	0	0	1	0.000106225
Mm.140380	0	0	5	0.000531124
Mm.155033	0	0	1	0.000106225
Mm.218533	0	0	4	0.000424899
Mm.294623	0	0	1	0.000106225
Mm.249809	0	0	2	0.00021245
Mm.29900	0	0	2	0.00021245
Mm.195628	0	0	1	0.000106225
Mm.28291	2	0.001004016	1	0.000106225
Mm.23825	0	0	2	0.00021245

Mm.295618	0	0	4	0.000424899
Mm.2050	0	0	5	0.000531124
Mm.350080	0	0	9	0.000956023
Mm.289669	0	0	1	0.000106225
Mm.345333	0	0	1	0.000106225
Mm.292517	0	0	2	0.00021245
Mm.371622	1	0.000502008	1	0.000106225
Mm.289810	0	0	12	0.001274697
Mm.371625	0	0	6	0.000637349
Mm.373568	2	0.001004016	11	0.001168472
Mm.45171	0	0	1	0.000106225
Mm.21938	0	0	4	0.000424899
Mm.238817	0	0	2	0.00021245
Mm.280083	1	0.000502008	15	0.001593372
Mm.290786	0	0	11	0.001168472
Mm.339491	0	0	8	0.000849798
Mm.21724	0	0	2	0.00021245
Mm.289800	1	0.000502008	2	0.00021245
Mm.107869	0	0	3	0.000318674
Mm.140568	0	0	5	0.000531124
Mm.236795	0	0	1	0.000106225
Mm.287443	0	0	1	0.000106225
Mm.28753	3	0.001506024	0	0
Mm.146283	0	0	1	0.000106225
Mm.27871	0	0	2	0.00021245
Mm.70690	0	0	6	0.000637349
Mm.316754	0	0	1	0.000106225
Mm.268369	0	0	2	0.00021245
Mm.29714	0	0	1	0.000106225
Mm.288960	0	0	1	0.000106225
Mm.180873	0	0	6	0.000637349
Mm.371658	0	0	11	0.001168472
Mm.13944	0	0	19	0.002018271
Mm.199698	1	0.000502008	2	0.00021245
Mm.218515	1	0.000502008	0	0
Mm.196544	0	0	1	0.000106225
Mm.34951	0	0	1	0.000106225
Mm.331142	0	0	3	0.000318674
Mm.351579	1	0.000502008	2	0.00021245
Mm.126534	0	0	1	0.000106225
Mm.34701	0	0	1	0.000106225
Mm.341243	0	0	1	0.000106225
Mm.18845	0	0	1	0.000106225
Mm.273122	1	0.000502008	0	0
Mm.251255	0	0	3	0.000318674
Mm.293628	1	0.000502008	0	0
Mm.261831	1	0.000502008	4	0.000424899
Mm.288669	0	0	3	0.000318674
Mm.303071	0	0	4	0.000424899
Mm.27764	0	0	3	0.000318674
Mm.5356	0	0	2	0.00021245
Mm.256765	3	0.001506024	0	0
Mm.22673	0	0	1	0.000106225
Mm.22119	0	0	1	0.000106225
Mm.213003	0	0	1	0.000106225
Mm.1894	0	0	1	0.000106225

Mm.105218	0	0	1	0.000106225				
Mm.41417	0	0	1	0.000106225				
Mm.329993	0	0	1	0.000106225				
Mm.257482	0	0	3	0.000318674				
Mm.336104	0	0	2	0.00021245				
Mm.188544	0	0	1	0.000106225				
Mm.319038	0	0	2	0.00021245				
Mm.2863	0	0	1	0.000106225				
Mm.248334	0	0	1	0.000106225				
Mm.4954	0	0	2	0.00021245				
Mm.258094	2	0.001004016	1	0.000106225				
Mm.11132	0	0	1	0.000106225				
Mm.173119	0	0	1	0.000106225				
Mm.154783	0	0	1	0.000106225				
Mm.21596	0	0	1	0.000106225				
Mm.18353	0	0	1	0.000106225				
Mm.3196	0	0	2	0.00021245				
Mm.214593	0	0	1	0.000106225				
Mm.7236	0	0	3	0.000318674				
Mm.259191	0	0	2	0.00021245				
Mm.271670	0	0	1	0.000106225				
Mm.281298	0	0	1	0.000106225				
Mm.11827	1	0.000502008	0	0				
Mm.210305	0	0	2	0.00021245				
Mm.65021	0	0	7	0.000743573				
Mm.272427	0	0	2	0.00021245				
Mm.32889	0	0	1	0.000106225				
Mm.28549	0	0	1	0.000106225				
Mm.280768	0	0	1	0.000106225				
Mm.22147	0	0	2	0.00021245				
Mm.341719	1	0.000502008	7	0.000743573				
Mm.274904	0	0	1	0.000106225				
Mm.22363	0	0	1	0.000106225				
Mm.486	0	0	4	0.000424899				
Mm.27435	0	0	1	0.000106225				
Mm.26379	2	0.001004016	0	0				
>GO:0009059	65	0.045391061	960	0.132596685	2.92120697	1.47E-20	1.28E-17	macromolecule biosynthesis P
Mm.21118	0	0	3	0.000318674				
Mm.371950	0	0	1	0.000106225				
Mm.145488	0	0	3	0.000318674				
Mm.154511	3	0.001506024	7	0.000743573				
Mm.100113	0	0	16	0.001699596				
Mm.361980	0	0	14	0.001487147				
Mm.286061	0	0	3	0.000318674				
Mm.298467	0	0	1	0.000106225				
Mm.371545	0	0	7	0.000743573				
Mm.5286	0	0	23	0.00244317				
Mm.217354	0	0	1	0.000106225				
Mm.138471	0	0	1	0.000106225				
Mm.360075	4	0.002008032	85	0.009029106				
Mm.289431	0	0	17	0.001805821				
Mm.262037	0	0	1	0.000106225				
Mm.196220	1	0.000502008	0	0				
Mm.29394	0	0	1	0.000106225				
Mm.2238	0	0	2	0.00021245				
Mm.371557	0	0	10	0.001062248				

Mm.260084	0	0	5	0.000531124
Mm.3941	1	0.000502008	9	0.000956023
Mm.185453	2	0.001004016	14	0.001487147
Mm.235137	0	0	2	0.00021245
Mm.329631	0	0	1	0.000106225
Mm.289992	0	0	4	0.000424899
Mm.271674	0	0	3	0.000318674
Mm.4071	0	0	11	0.001168472
Mm.328846	0	0	1	0.000106225
Mm.197555	1	0.000502008	2	0.00021245
Mm.182628	0	0	3	0.000318674
Mm.336955	0	0	1	0.000106225
Mm.347060	1	0.000502008	4	0.000424899
Mm.349277	0	0	8	0.000849798
Mm.372072	0	0	3	0.000318674
Mm.309019	0	0	1	0.000106225
Mm.29046	0	0	2	0.00021245
Mm.316592	0	0	4	0.000424899
Mm.307846	0	0	4	0.000424899
Mm.12144	1	0.000502008	0	0
Mm.306548	0	0	1	0.000106225
Mm.300263	0	0	3	0.000318674
Mm.325521	0	0	5	0.000531124
Mm.247113	0	0	3	0.000318674
Mm.358632	0	0	1	0.000106225
Mm.104368	0	0	6	0.000637349
Mm.21529	1	0.000502008	1	0.000106225
Mm.371576	0	0	1	0.000106225
Mm.323357	0	0	4	0.000424899
Mm.354330	0	0	10	0.001062248
Mm.4419	0	0	1	0.000106225
Mm.262021	0	0	18	0.001912046
Mm.282053	0	0	14	0.001487147
Mm.290772	0	0	1	0.000106225
Mm.289868	0	0	9	0.000956023
Mm.300271	0	0	1	0.000106225
Mm.353923	0	0	6	0.000637349
Mm.43778	0	0	7	0.000743573
Mm.643	0	0	7	0.000743573
Mm.702	0	0	1	0.000106225
Mm.371577	0	0	2	0.00021245
Mm.371578	0	0	2	0.00021245
Mm.300281	0	0	1	0.000106225
Mm.35583	0	0	1	0.000106225
Mm.16775	3	0.001506024	11	0.001168472
Mm.154915	0	0	2	0.00021245
Mm.331113	0	0	11	0.001168472
Mm.6957	0	0	6	0.000637349
Mm.66	0	0	21	0.00223072
Mm.5291	2	0.001004016	17	0.001805821
Mm.325584	0	0	3	0.000318674
Mm.371579	0	0	5	0.000531124
Mm.260904	0	0	4	0.000424899
Mm.299312	0	0	1	0.000106225
Mm.13886	0	0	1	0.000106225
Mm.28839	3	0.001506024	3	0.000318674

Mm.262067	0	0	1	0.000106225
Mm.29041	0	0	1	0.000106225
Mm.271222	2	0.001004016	10	0.001062248
Mm.328378	0	0	1	0.000106225
Mm.180458	2	0.001004016	14	0.001487147
Mm.297372	0	0	4	0.000424899
Mm.275195	0	0	2	0.00021245
Mm.312227	0	0	1	0.000106225
Mm.329353	0	0	2	0.00021245
Mm.259294	1	0.000502008	0	0
Mm.260943	0	0	3	0.000318674
Mm.52275	1	0.000502008	0	0
Mm.21105	0	0	1	0.000106225
Mm.274482	0	0	1	0.000106225
Mm.288212	1	0.000502008	2	0.00021245
Mm.22723	1	0.000502008	2	0.00021245
Mm.218851	0	0	5	0.000531124
Mm.250909	0	0	1	0.000106225
Mm.250030	0	0	11	0.001168472
Mm.30066	1	0.000502008	14	0.001487147
Mm.227183	0	0	1	0.000106225
Mm.319719	0	0	2	0.00021245
Mm.236868	0	0	1	0.000106225
Mm.330075	1	0.000502008	11	0.001168472
Mm.196538	0	0	6	0.000637349
Mm.316362	0	0	1	0.000106225
Mm.290899	0	0	22	0.002336945
Mm.261679	0	0	6	0.000637349
Mm.29183	1	0.000502008	0	0
Mm.196607	0	0	8	0.000849798
Mm.21671	0	0	4	0.000424899
Mm.276337	0	0	5	0.000531124
Mm.219675	0	0	1	0.000106225
Mm.27816	0	0	3	0.000318674
Mm.193040	0	0	1	0.000106225
Mm.260064	0	0	7	0.000743573
Mm.11376	0	0	2	0.00021245
Mm.277891	0	0	1	0.000106225
Mm.250874	0	0	2	0.00021245
Mm.3955	1	0.000502008	5	0.000531124
Mm.2718	0	0	11	0.001168472
Mm.3158	0	0	5	0.000531124
Mm.133851	0	0	1	0.000106225
Mm.22776	1	0.000502008	4	0.000424899
Mm.19355	0	0	1	0.000106225
Mm.27796	0	0	1	0.000106225
Mm.313236	0	0	1	0.000106225
Mm.140380	0	0	5	0.000531124
Mm.155033	0	0	1	0.000106225
Mm.218533	0	0	4	0.000424899
Mm.294623	0	0	1	0.000106225
Mm.249809	0	0	2	0.00021245
Mm.29900	0	0	2	0.00021245
Mm.195628	0	0	1	0.000106225
Mm.28291	2	0.001004016	1	0.000106225
Mm.23825	0	0	2	0.00021245

Mm.295618	0	0	4	0.000424899
Mm.2050	0	0	5	0.000531124
Mm.350080	0	0	9	0.000956023
Mm.289669	0	0	1	0.000106225
Mm.345333	0	0	1	0.000106225
Mm.292517	0	0	2	0.00021245
Mm.371622	1	0.000502008	1	0.000106225
Mm.289810	0	0	12	0.001274697
Mm.371625	0	0	6	0.000637349
Mm.373568	2	0.001004016	11	0.001168472
Mm.45171	0	0	1	0.000106225
Mm.21938	0	0	4	0.000424899
Mm.238817	0	0	2	0.00021245
Mm.280083	1	0.000502008	15	0.001593372
Mm.290786	0	0	11	0.001168472
Mm.339491	0	0	8	0.000849798
Mm.21724	0	0	2	0.00021245
Mm.289800	1	0.000502008	2	0.00021245
Mm.107869	0	0	3	0.000318674
Mm.140568	0	0	5	0.000531124
Mm.236795	0	0	1	0.000106225
Mm.287443	0	0	1	0.000106225
Mm.28753	3	0.001506024	0	0
Mm.146283	0	0	1	0.000106225
Mm.27871	0	0	2	0.00021245
Mm.70690	0	0	6	0.000637349
Mm.316754	0	0	1	0.000106225
Mm.268369	0	0	2	0.00021245
Mm.29714	0	0	1	0.000106225
Mm.288960	0	0	1	0.000106225
Mm.180873	0	0	6	0.000637349
Mm.371658	0	0	11	0.001168472
Mm.13944	0	0	19	0.002018271
Mm.199698	1	0.000502008	2	0.00021245
Mm.218515	1	0.000502008	0	0
Mm.196544	0	0	1	0.000106225
Mm.34951	0	0	1	0.000106225
Mm.331142	0	0	3	0.000318674
Mm.351579	1	0.000502008	2	0.00021245
Mm.126534	0	0	1	0.000106225
Mm.34701	0	0	1	0.000106225
Mm.341243	0	0	1	0.000106225
Mm.18845	0	0	1	0.000106225
Mm.273122	1	0.000502008	0	0
Mm.251255	0	0	3	0.000318674
Mm.293628	1	0.000502008	0	0
Mm.261831	1	0.000502008	4	0.000424899
Mm.288669	0	0	3	0.000318674
Mm.303071	0	0	4	0.000424899
Mm.27764	0	0	3	0.000318674
Mm.5356	0	0	2	0.00021245
Mm.256765	3	0.001506024	0	0
Mm.22673	0	0	1	0.000106225
Mm.22119	0	0	1	0.000106225
Mm.213003	0	0	1	0.000106225
Mm.1894	0	0	1	0.000106225

Mm.105218	0	0	1	0.000106225					
Mm.41417	0	0	1	0.000106225					
Mm.329993	0	0	1	0.000106225					
Mm.257482	0	0	3	0.000318674					
Mm.336104	0	0	2	0.00021245					
Mm.188544	0	0	1	0.000106225					
Mm.319038	0	0	2	0.00021245					
Mm.2863	0	0	1	0.000106225					
Mm.248334	0	0	1	0.000106225					
Mm.4954	0	0	2	0.00021245					
Mm.258094	2	0.001004016	1	0.000106225					
Mm.11132	0	0	1	0.000106225					
Mm.173119	0	0	1	0.000106225					
Mm.154783	0	0	1	0.000106225					
Mm.21596	0	0	1	0.000106225					
Mm.18353	0	0	1	0.000106225					
Mm.3196	0	0	2	0.00021245					
Mm.214593	0	0	1	0.000106225					
Mm.7236	0	0	3	0.000318674					
Mm.259191	0	0	2	0.00021245					
Mm.271670	0	0	1	0.000106225					
Mm.281298	0	0	1	0.000106225					
Mm.11827	1	0.000502008	0	0					
Mm.210305	0	0	2	0.00021245					
Mm.65021	0	0	7	0.000743573					
Mm.272427	0	0	2	0.00021245					
Mm.32889	0	0	1	0.000106225					
Mm.28549	0	0	1	0.000106225					
Mm.280768	0	0	1	0.000106225					
Mm.22147	0	0	2	0.00021245					
Mm.341719	1	0.000502008	7	0.000743573					
Mm.274904	0	0	1	0.000106225					
Mm.22363	0	0	1	0.000106225					
Mm.486	0	0	4	0.000424899					
Mm.27435	0	0	1	0.000106225					
Mm.26379	2	0.001004016	0	0					
Mm.19893	0	0	2	0.00021245					
Mm.6375	0	0	1	0.000106225					
Mm.181862	1	0.000502008	0	0					
Mm.29201	0	0	2	0.00021245					
Mm.12863	1	0.000502008	1	0.000106225					
Mm.333916	1	0.000502008	1	0.000106225					
Mm.15622	0	0	2	0.00021245					
Mm.202561	1	0.000502008	1	0.000106225					
Mm.233534	0	0	4	0.000424899					
Mm.29357	0	0	4	0.000424899					
Mm.641	0	0	1	0.000106225					
Mm.289741	0	0	1	0.000106225					
Mm.3711	0	0	4	0.000424899					
Mm.4222	0	0	3	0.000318674					
Mm.18939	0	0	1	0.000106225					
Mm.22374	0	0	1	0.000106225					
>GO:0005840	23	0.016061453	571	0.078867403	4.910353111	1.34E-17	9.36E-15	ribosome	C
Mm.100113	0	0	16	0.001699596					
Mm.361980	0	0	14	0.001487147					
Mm.298467	0	0	1	0.000106225					

Mm.371545	0	0	7	0.000743573
Mm.5286	0	0	23	0.00244317
Mm.217354	0	0	1	0.000106225
Mm.329631	0	0	1	0.000106225
Mm.4071	0	0	11	0.001168472
Mm.328846	0	0	1	0.000106225
Mm.182628	0	0	3	0.000318674
Mm.336955	0	0	1	0.000106225
Mm.347060	1	0.000502008	4	0.000424899
Mm.349277	0	0	8	0.000849798
Mm.372072	0	0	3	0.000318674
Mm.309019	0	0	1	0.000106225
Mm.29046	0	0	2	0.00021245
Mm.316592	0	0	4	0.000424899
Mm.307846	0	0	4	0.000424899
Mm.306548	0	0	1	0.000106225
Mm.300263	0	0	3	0.000318674
Mm.325521	0	0	5	0.000531124
Mm.247113	0	0	3	0.000318674
Mm.358632	0	0	1	0.000106225
Mm.104368	0	0	6	0.000637349
Mm.21529	1	0.000502008	1	0.000106225
Mm.371576	0	0	1	0.000106225
Mm.323357	0	0	4	0.000424899
Mm.354330	0	0	10	0.001062248
Mm.4419	0	0	1	0.000106225
Mm.262021	0	0	18	0.001912046
Mm.282053	0	0	14	0.001487147
Mm.290772	0	0	1	0.000106225
Mm.289868	0	0	9	0.000956023
Mm.300271	0	0	1	0.000106225
Mm.353923	0	0	6	0.000637349
Mm.43778	0	0	7	0.000743573
Mm.643	0	0	7	0.000743573
Mm.702	0	0	1	0.000106225
Mm.371577	0	0	2	0.00021245
Mm.371578	0	0	2	0.00021245
Mm.300281	0	0	1	0.000106225
Mm.35583	0	0	1	0.000106225
Mm.16775	3	0.001506024	11	0.001168472
Mm.154915	0	0	2	0.00021245
Mm.331113	0	0	11	0.001168472
Mm.6957	0	0	6	0.000637349
Mm.66	0	0	21	0.00223072
Mm.5291	2	0.001004016	17	0.001805821
Mm.325584	0	0	3	0.000318674
Mm.371579	0	0	5	0.000531124
Mm.260904	0	0	4	0.000424899
Mm.299312	0	0	1	0.000106225
Mm.262067	0	0	1	0.000106225
Mm.328378	0	0	1	0.000106225
Mm.180458	2	0.001004016	14	0.001487147
Mm.297372	0	0	4	0.000424899
Mm.275195	0	0	2	0.00021245
Mm.312227	0	0	1	0.000106225
Mm.288212	1	0.000502008	2	0.00021245

Mm.22723	1	0.000502008	2	0.00021245
Mm.250030	0	0	11	0.001168472
Mm.30066	1	0.000502008	14	0.001487147
Mm.319719	0	0	2	0.00021245
Mm.236868	0	0	1	0.000106225
Mm.330075	1	0.000502008	11	0.001168472
Mm.196538	0	0	6	0.000637349
Mm.316362	0	0	1	0.000106225
Mm.290899	0	0	22	0.002336945
Mm.261679	0	0	6	0.000637349
Mm.29183	1	0.000502008	0	0
Mm.276337	0	0	5	0.000531124
Mm.11376	0	0	2	0.00021245
Mm.277891	0	0	1	0.000106225
Mm.3158	0	0	5	0.000531124
Mm.133851	0	0	1	0.000106225
Mm.19355	0	0	1	0.000106225
Mm.27796	0	0	1	0.000106225
Mm.313236	0	0	1	0.000106225
Mm.140380	0	0	5	0.000531124
Mm.155033	0	0	1	0.000106225
Mm.218533	0	0	4	0.000424899
Mm.249809	0	0	2	0.00021245
Mm.195628	0	0	1	0.000106225
Mm.28291	2	0.001004016	1	0.000106225
Mm.295618	0	0	4	0.000424899
Mm.2050	0	0	5	0.000531124
Mm.350080	0	0	9	0.000956023
Mm.289669	0	0	1	0.000106225
Mm.345333	0	0	1	0.000106225
Mm.371622	1	0.000502008	1	0.000106225
Mm.289810	0	0	12	0.001274697
Mm.341719	1	0.000502008	7	0.000743573
Mm.21938	0	0	4	0.000424899
Mm.238817	0	0	2	0.00021245
Mm.280083	1	0.000502008	15	0.001593372
Mm.290786	0	0	11	0.001168472
Mm.339491	0	0	8	0.000849798
Mm.21724	0	0	2	0.00021245
Mm.107869	0	0	3	0.000318674
Mm.140568	0	0	5	0.000531124
Mm.236795	0	0	1	0.000106225
Mm.287443	0	0	1	0.000106225
Mm.27871	0	0	2	0.00021245
Mm.288960	0	0	1	0.000106225
Mm.180873	0	0	6	0.000637349
Mm.371658	0	0	11	0.001168472
Mm.13944	0	0	19	0.002018271
Mm.199698	1	0.000502008	2	0.00021245
Mm.218515	1	0.000502008	0	0
Mm.34951	0	0	1	0.000106225
Mm.193040	0	0	1	0.000106225
Mm.168680	0	0	4	0.000424899
Mm.12144	1	0.000502008	0	0
Mm.23825	0	0	2	0.00021245
Mm.260737	0	0	5	0.000531124

Mm.262707	0	0	1	0.000106225				
Mm.297196	1	0.000502008	0	0				
>GO:0003735	26	0.018156425	588	0.08121547	4.473098173	3.06E-17	1.78E-14	structural constituent of ribo F
Mm.100113	0	0	16	0.001699596				
Mm.361980	0	0	14	0.001487147				
Mm.298467	0	0	1	0.000106225				
Mm.371545	0	0	7	0.000743573				
Mm.5286	0	0	23	0.00244317				
Mm.217354	0	0	1	0.000106225				
Mm.329631	0	0	1	0.000106225				
Mm.35498	0	0	1	0.000106225				
Mm.4071	0	0	11	0.001168472				
Mm.328846	0	0	1	0.000106225				
Mm.182628	0	0	3	0.000318674				
Mm.336955	0	0	1	0.000106225				
Mm.347060	1	0.000502008	4	0.000424899				
Mm.349277	0	0	8	0.000849798				
Mm.372072	0	0	3	0.000318674				
Mm.309019	0	0	1	0.000106225				
Mm.29046	0	0	2	0.00021245				
Mm.316592	0	0	4	0.000424899				
Mm.307846	0	0	4	0.000424899				
Mm.12144	1	0.000502008	0	0				
Mm.306548	0	0	1	0.000106225				
Mm.260737	0	0	5	0.000531124				
Mm.300263	0	0	3	0.000318674				
Mm.325521	0	0	5	0.000531124				
Mm.247113	0	0	3	0.000318674				
Mm.358632	0	0	1	0.000106225				
Mm.104368	0	0	6	0.000637349				
Mm.21529	1	0.000502008	1	0.000106225				
Mm.371576	0	0	1	0.000106225				
Mm.323357	0	0	4	0.000424899				
Mm.354330	0	0	10	0.001062248				
Mm.4419	0	0	1	0.000106225				
Mm.262021	0	0	18	0.001912046				
Mm.282053	0	0	14	0.001487147				
Mm.290772	0	0	1	0.000106225				
Mm.289868	0	0	9	0.000956023				
Mm.300271	0	0	1	0.000106225				
Mm.353923	0	0	6	0.000637349				
Mm.43778	0	0	7	0.000743573				
Mm.643	0	0	7	0.000743573				
Mm.702	0	0	1	0.000106225				
Mm.371577	0	0	2	0.00021245				
Mm.371578	0	0	2	0.00021245				
Mm.300281	0	0	1	0.000106225				
Mm.35583	0	0	1	0.000106225				
Mm.16775	3	0.001506024	11	0.001168472				
Mm.154915	0	0	2	0.00021245				
Mm.331113	0	0	11	0.001168472				
Mm.6957	0	0	6	0.000637349				
Mm.66	0	0	21	0.00223072				
Mm.5291	2	0.001004016	17	0.001805821				
Mm.325584	0	0	3	0.000318674				
Mm.371579	0	0	5	0.000531124				

Mm.260904	0	0	4	0.000424899
Mm.299312	0	0	1	0.000106225
Mm.262067	0	0	1	0.000106225
Mm.328378	0	0	1	0.000106225
Mm.180458	2	0.001004016	14	0.001487147
Mm.297372	0	0	4	0.000424899
Mm.275195	0	0	2	0.00021245
Mm.312227	0	0	1	0.000106225
Mm.288212	1	0.000502008	2	0.00021245
Mm.22723	1	0.000502008	2	0.00021245
Mm.250030	0	0	11	0.001168472
Mm.30066	1	0.000502008	14	0.001487147
Mm.319719	0	0	2	0.00021245
Mm.236868	0	0	1	0.000106225
Mm.330075	1	0.000502008	11	0.001168472
Mm.196538	0	0	6	0.000637349
Mm.316362	0	0	1	0.000106225
Mm.290899	0	0	22	0.002336945
Mm.261679	0	0	6	0.000637349
Mm.29183	1	0.000502008	0	0
Mm.276337	0	0	5	0.000531124
Mm.193040	0	0	1	0.000106225
Mm.10996	0	0	1	0.000106225
Mm.371602	0	0	2	0.00021245
Mm.11376	0	0	2	0.00021245
Mm.277891	0	0	1	0.000106225
Mm.3158	0	0	5	0.000531124
Mm.133851	0	0	1	0.000106225
Mm.19355	0	0	1	0.000106225
Mm.27796	0	0	1	0.000106225
Mm.313236	0	0	1	0.000106225
Mm.168680	0	0	4	0.000424899
Mm.140380	0	0	5	0.000531124
Mm.155033	0	0	1	0.000106225
Mm.218533	0	0	4	0.000424899
Mm.38460	0	0	1	0.000106225
Mm.249809	0	0	2	0.00021245
Mm.195628	0	0	1	0.000106225
Mm.28291	2	0.001004016	1	0.000106225
Mm.23825	0	0	2	0.00021245
Mm.295618	0	0	4	0.000424899
Mm.2050	0	0	5	0.000531124
Mm.350080	0	0	9	0.000956023
Mm.289669	0	0	1	0.000106225
Mm.345333	0	0	1	0.000106225
Mm.371622	1	0.000502008	1	0.000106225
Mm.5381	0	0	2	0.00021245
Mm.37617	1	0.000502008	0	0
Mm.275810	0	0	4	0.000424899
Mm.371624	1	0.000502008	2	0.00021245
Mm.289810	0	0	12	0.001274697
Mm.341719	1	0.000502008	7	0.000743573
Mm.21938	0	0	4	0.000424899
Mm.238817	0	0	2	0.00021245
Mm.290166	1	0.000502008	4	0.000424899
Mm.196635	1	0.000502008	0	0

Mm.280083	1	0.000502008	15	0.001593372					
Mm.290786	0	0	11	0.001168472					
Mm.339491	0	0	8	0.000849798					
Mm.21724	0	0	2	0.00021245					
Mm.107869	0	0	3	0.000318674					
Mm.140568	0	0	5	0.000531124					
Mm.236795	0	0	1	0.000106225					
Mm.287443	0	0	1	0.000106225					
Mm.27871	0	0	2	0.00021245					
Mm.262707	0	0	1	0.000106225					
Mm.288960	0	0	1	0.000106225					
Mm.180873	0	0	6	0.000637349					
Mm.371658	0	0	11	0.001168472					
Mm.13944	0	0	19	0.002018271					
Mm.199698	1	0.000502008	2	0.00021245					
Mm.218515	1	0.000502008	0	0					
Mm.34951	0	0	1	0.000106225					
>GO:0030529	44	0.030726257	721	0.099585635	3.241059769	7.17E-17	3.57E-14	ribonucleoprotein complex	C
Mm.12864	0	0	1	0.000106225					
Mm.371669	0	0	2	0.00021245					
Mm.100113	0	0	16	0.001699596					
Mm.361980	0	0	14	0.001487147					
Mm.298467	0	0	1	0.000106225					
Mm.371545	0	0	7	0.000743573					
Mm.5286	0	0	23	0.00244317					
Mm.150231	0	0	1	0.000106225					
Mm.217354	0	0	1	0.000106225					
Mm.329631	0	0	1	0.000106225					
Mm.274690	2	0.001004016	12	0.001274697					
Mm.299367	0	0	1	0.000106225					
Mm.280842	0	0	10	0.001062248					
Mm.288451	1	0.000502008	8	0.000849798					
Mm.35498	0	0	1	0.000106225					
Mm.4071	0	0	11	0.001168472					
Mm.328846	0	0	1	0.000106225					
Mm.236513	1	0.000502008	0	0					
Mm.40120	0	0	1	0.000106225					
Mm.221440	1	0.000502008	1	0.000106225					
Mm.336955	0	0	1	0.000106225					
Mm.347060	1	0.000502008	4	0.000424899					
Mm.349277	0	0	8	0.000849798					
Mm.372072	0	0	3	0.000318674					
Mm.309019	0	0	1	0.000106225					
Mm.29046	0	0	2	0.00021245					
Mm.316592	0	0	4	0.000424899					
Mm.307846	0	0	4	0.000424899					
Mm.12144	1	0.000502008	0	0					
Mm.306548	0	0	1	0.000106225					
Mm.260737	0	0	5	0.000531124					
Mm.300263	0	0	3	0.000318674					
Mm.325521	0	0	5	0.000531124					
Mm.247113	0	0	3	0.000318674					
Mm.358632	0	0	1	0.000106225					
Mm.104368	0	0	6	0.000637349					
Mm.21529	1	0.000502008	1	0.000106225					
Mm.323357	0	0	4	0.000424899					

Mm.354330	0	0	10	0.001062248
Mm.4419	0	0	1	0.000106225
Mm.262021	0	0	18	0.001912046
Mm.282053	0	0	14	0.001487147
Mm.290772	0	0	1	0.000106225
Mm.289868	0	0	9	0.000956023
Mm.300271	0	0	1	0.000106225
Mm.353923	0	0	6	0.000637349
Mm.43778	0	0	7	0.000743573
Mm.643	0	0	7	0.000743573
Mm.702	0	0	1	0.000106225
Mm.371577	0	0	2	0.00021245
Mm.371578	0	0	2	0.00021245
Mm.300281	0	0	1	0.000106225
Mm.35583	0	0	1	0.000106225
Mm.16775	3	0.001506024	11	0.001168472
Mm.154915	0	0	2	0.00021245
Mm.331113	0	0	11	0.001168472
Mm.6957	0	0	6	0.000637349
Mm.66	0	0	21	0.00223072
Mm.5291	2	0.001004016	17	0.001805821
Mm.325584	0	0	3	0.000318674
Mm.371579	0	0	5	0.000531124
Mm.260904	0	0	4	0.000424899
Mm.308514	0	0	1	0.000106225
Mm.88216	0	0	2	0.00021245
Mm.1323	1	0.000502008	1	0.000106225
Mm.274995	0	0	3	0.000318674
Mm.321227	0	0	1	0.000106225
Mm.355161	0	0	1	0.000106225
Mm.40370	0	0	1	0.000106225
Mm.299312	0	0	1	0.000106225
Mm.73276	1	0.000502008	0	0
Mm.29655	0	0	1	0.000106225
Mm.180458	2	0.001004016	14	0.001487147
Mm.297372	0	0	4	0.000424899
Mm.316306	0	0	1	0.000106225
Mm.331491	0	0	3	0.000318674
Mm.274146	0	0	1	0.000106225
Mm.288212	1	0.000502008	2	0.00021245
Mm.22723	1	0.000502008	2	0.00021245
Mm.250030	0	0	11	0.001168472
Mm.30066	1	0.000502008	14	0.001487147
Mm.319719	0	0	2	0.00021245
Mm.236868	0	0	1	0.000106225
Mm.330075	1	0.000502008	11	0.001168472
Mm.196538	0	0	6	0.000637349
Mm.316362	0	0	1	0.000106225
Mm.290899	0	0	22	0.002336945
Mm.261679	0	0	6	0.000637349
Mm.239997	1	0.000502008	0	0
Mm.276337	0	0	5	0.000531124
Mm.193040	0	0	1	0.000106225
Mm.195310	1	0.000502008	3	0.000318674
Mm.2115	1	0.000502008	2	0.00021245
Mm.155896	0	0	3	0.000318674

Mm.11376	0	0	2	0.00021245
Mm.3158	0	0	5	0.000531124
Mm.133851	0	0	1	0.000106225
Mm.260545	0	0	1	0.000106225
Mm.19355	0	0	1	0.000106225
Mm.21740	0	0	2	0.00021245
Mm.168680	0	0	4	0.000424899
Mm.140380	0	0	5	0.000531124
Mm.306162	0	0	1	0.000106225
Mm.155033	0	0	1	0.000106225
Mm.218533	0	0	4	0.000424899
Mm.38460	0	0	1	0.000106225
Mm.249809	0	0	2	0.00021245
Mm.333087	0	0	1	0.000106225
Mm.195628	0	0	1	0.000106225
Mm.28291	2	0.001004016	1	0.000106225
Mm.295618	0	0	4	0.000424899
Mm.2050	0	0	5	0.000531124
Mm.350080	0	0	9	0.000956023
Mm.289669	0	0	1	0.000106225
Mm.345333	0	0	1	0.000106225
Mm.371622	1	0.000502008	1	0.000106225
Mm.5381	0	0	2	0.00021245
Mm.275810	0	0	4	0.000424899
Mm.371624	1	0.000502008	2	0.00021245
Mm.289810	0	0	12	0.001274697
Mm.341719	1	0.000502008	7	0.000743573
Mm.259121	0	0	2	0.00021245
Mm.21938	0	0	4	0.000424899
Mm.238817	0	0	2	0.00021245
Mm.246693	0	0	3	0.000318674
Mm.290166	1	0.000502008	4	0.000424899
Mm.280083	1	0.000502008	15	0.001593372
Mm.290786	0	0	11	0.001168472
Mm.339491	0	0	8	0.000849798
Mm.107869	0	0	3	0.000318674
Mm.140568	0	0	5	0.000531124
Mm.236795	0	0	1	0.000106225
Mm.287443	0	0	1	0.000106225
Mm.288960	0	0	1	0.000106225
Mm.275158	0	0	1	0.000106225
Mm.180873	0	0	6	0.000637349
Mm.371658	0	0	11	0.001168472
Mm.13944	0	0	19	0.002018271
Mm.148973	0	0	1	0.000106225
Mm.199698	1	0.000502008	2	0.00021245
Mm.45683	0	0	1	0.000106225
Mm.228797	1	0.000502008	0	0
Mm.218515	1	0.000502008	0	0
Mm.34951	0	0	1	0.000106225
Mm.122366	1	0.000502008	4	0.000424899
Mm.352859	0	0	1	0.000106225
Mm.371670	0	0	1	0.000106225
Mm.45645	0	0	7	0.000743573
Mm.181824	0	0	2	0.00021245
Mm.132392	0	0	3	0.000318674

Mm.370205	0	0	3	0.000318674					
Mm.358634	1	0.000502008	6	0.000637349					
Mm.43331	0	0	3	0.000318674					
Mm.272685	0	0	2	0.00021245					
Mm.172411	0	0	1	0.000106225					
Mm.1963	0	0	4	0.000424899					
Mm.272826	0	0	1	0.000106225					
Mm.35353	0	0	1	0.000106225					
Mm.248755	4	0.002008032	1	0.000106225					
Mm.279985	0	0	2	0.00021245					
Mm.104919	0	0	1	0.000106225					
Mm.271715	0	0	1	0.000106225					
Mm.279872	0	0	2	0.00021245					
Mm.46063	0	0	1	0.000106225					
Mm.313687	0	0	1	0.000106225					
Mm.182769	0	0	2	0.00021245					
Mm.279736	2	0.001004016	3	0.000318674					
Mm.3757	0	0	1	0.000106225					
Mm.182628	0	0	3	0.000318674					
Mm.371576	0	0	1	0.000106225					
Mm.262067	0	0	1	0.000106225					
Mm.328378	0	0	1	0.000106225					
Mm.275195	0	0	2	0.00021245					
Mm.312227	0	0	1	0.000106225					
Mm.29183	1	0.000502008	0	0					
Mm.277891	0	0	1	0.000106225					
Mm.27796	0	0	1	0.000106225					
Mm.313236	0	0	1	0.000106225					
Mm.21724	0	0	2	0.00021245					
Mm.27871	0	0	2	0.00021245					
Mm.23825	0	0	2	0.00021245					
Mm.262707	0	0	1	0.000106225					
Mm.297196	1	0.000502008	0	0					
Mm.167586	0	0	1	0.000106225					
Mm.78861	0	0	4	0.000424899					
Mm.246863	0	0	1	0.000106225					
Mm.38344	0	0	2	0.00021245					
>GO:0005198	54	0.037709497	754	0.104143646	2.761735216	4.07E-15	1.77E-12	structural molecule activity	F
Mm.355327	3	0.001506024	3	0.000318674					
Mm.297	0	0	2	0.00021245					
Mm.300639	3	0.001506024	13	0.001380922					
Mm.213025	0	0	6	0.000637349					
Mm.289106	2	0.001004016	1	0.000106225					
Mm.119936	0	0	1	0.000106225					
Mm.18962	0	0	1	0.000106225					
Mm.291928	1	0.000502008	0	0					
Mm.35738	0	0	2	0.00021245					
Mm.22680	0	0	1	0.000106225					
Mm.158662	0	0	3	0.000318674					
Mm.3819	0	0	1	0.000106225					
Mm.4352	0	0	1	0.000106225					
Mm.249555	0	0	4	0.000424899					
Mm.181021	0	0	4	0.000424899					
Mm.2509	1	0.000502008	2	0.00021245					
Mm.277792	0	0	1	0.000106225					
Mm.275608	0	0	1	0.000106225					

Mm.336625	0	0	2	0.00021245
Mm.293683	0	0	1	0.000106225
Mm.3982	0	0	1	0.000106225
Mm.329287	1	0.000502008	0	0
Mm.12508	0	0	7	0.000743573
Mm.312059	0	0	6	0.000637349
Mm.22479	0	0	9	0.000956023
Mm.273177	0	0	1	0.000106225
Mm.358618	0	0	6	0.000637349
Mm.42012	0	0	1	0.000106225
Mm.172674	1	0.000502008	4	0.000424899
Mm.243014	0	0	2	0.00021245
Mm.138876	1	0.000502008	0	0
Mm.297109	0	0	2	0.00021245
Mm.1956	0	0	2	0.00021245
Mm.234912	0	0	2	0.00021245
Mm.220821	0	0	1	0.000106225
Mm.245746	2	0.001004016	3	0.000318674
Mm.324696	1	0.000502008	4	0.000424899
Mm.287784	0	0	3	0.000318674
Mm.270295	0	0	1	0.000106225
Mm.273538	0	0	5	0.000531124
Mm.277812	1	0.000502008	1	0.000106225
Mm.268000	0	0	1	0.000106225
Mm.219648	0	0	3	0.000318674
Mm.329277	0	0	3	0.000318674
Mm.1249	0	0	1	0.000106225
Mm.227260	0	0	10	0.001062248
Mm.21109	0	0	3	0.000318674
Mm.251188	1	0.000502008	0	0
Mm.4420	2	0.001004016	0	0
Mm.23895	0	0	1	0.000106225
Mm.44106	0	0	1	0.000106225
Mm.266767	0	0	4	0.000424899
Mm.281896	0	0	1	0.000106225
Mm.3118	0	0	1	0.000106225
Mm.218891	1	0.000502008	0	0
Mm.86421	0	0	1	0.000106225
Mm.25836	1	0.000502008	0	0
Mm.215110	0	0	1	0.000106225
Mm.370228	0	0	1	0.000106225
Mm.276367	0	0	1	0.000106225
Mm.259045	0	0	2	0.00021245
Mm.335292	0	0	2	0.00021245
Mm.183102	0	0	5	0.000531124
Mm.234823	1	0.000502008	0	0
Mm.235074	3	0.001506024	0	0
Mm.292567	0	0	1	0.000106225
Mm.317073	1	0.000502008	0	0
Mm.100113	0	0	16	0.001699596
Mm.361980	0	0	14	0.001487147
Mm.298467	0	0	1	0.000106225
Mm.371545	0	0	7	0.000743573
Mm.5286	0	0	23	0.00244317
Mm.217354	0	0	1	0.000106225
Mm.329631	0	0	1	0.000106225

Mm.35498	0	0	1	0.000106225
Mm.4071	0	0	11	0.001168472
Mm.328846	0	0	1	0.000106225
Mm.182628	0	0	3	0.000318674
Mm.336955	0	0	1	0.000106225
Mm.347060	1	0.000502008	4	0.000424899
Mm.349277	0	0	8	0.000849798
Mm.372072	0	0	3	0.000318674
Mm.309019	0	0	1	0.000106225
Mm.29046	0	0	2	0.00021245
Mm.316592	0	0	4	0.000424899
Mm.307846	0	0	4	0.000424899
Mm.12144	1	0.000502008	0	0
Mm.306548	0	0	1	0.000106225
Mm.260737	0	0	5	0.000531124
Mm.300263	0	0	3	0.000318674
Mm.325521	0	0	5	0.000531124
Mm.247113	0	0	3	0.000318674
Mm.358632	0	0	1	0.000106225
Mm.104368	0	0	6	0.000637349
Mm.21529	1	0.000502008	1	0.000106225
Mm.371576	0	0	1	0.000106225
Mm.323357	0	0	4	0.000424899
Mm.354330	0	0	10	0.001062248
Mm.4419	0	0	1	0.000106225
Mm.262021	0	0	18	0.001912046
Mm.282053	0	0	14	0.001487147
Mm.290772	0	0	1	0.000106225
Mm.289868	0	0	9	0.000956023
Mm.300271	0	0	1	0.000106225
Mm.353923	0	0	6	0.000637349
Mm.43778	0	0	7	0.000743573
Mm.643	0	0	7	0.000743573
Mm.702	0	0	1	0.000106225
Mm.371577	0	0	2	0.00021245
Mm.371578	0	0	2	0.00021245
Mm.300281	0	0	1	0.000106225
Mm.35583	0	0	1	0.000106225
Mm.16775	3	0.001506024	11	0.001168472
Mm.154915	0	0	2	0.00021245
Mm.331113	0	0	11	0.001168472
Mm.6957	0	0	6	0.000637349
Mm.66	0	0	21	0.00223072
Mm.5291	2	0.001004016	17	0.001805821
Mm.325584	0	0	3	0.000318674
Mm.371579	0	0	5	0.000531124
Mm.260904	0	0	4	0.000424899
Mm.299312	0	0	1	0.000106225
Mm.262067	0	0	1	0.000106225
Mm.328378	0	0	1	0.000106225
Mm.180458	2	0.001004016	14	0.001487147
Mm.297372	0	0	4	0.000424899
Mm.275195	0	0	2	0.00021245
Mm.312227	0	0	1	0.000106225
Mm.288212	1	0.000502008	2	0.00021245
Mm.22723	1	0.000502008	2	0.00021245

Mm.250030	0	0	11	0.001168472
Mm.30066	1	0.000502008	14	0.001487147
Mm.319719	0	0	2	0.00021245
Mm.236868	0	0	1	0.000106225
Mm.330075	1	0.000502008	11	0.001168472
Mm.196538	0	0	6	0.000637349
Mm.316362	0	0	1	0.000106225
Mm.290899	0	0	22	0.002336945
Mm.261679	0	0	6	0.000637349
Mm.29183	1	0.000502008	0	0
Mm.276337	0	0	5	0.000531124
Mm.193040	0	0	1	0.000106225
Mm.10996	0	0	1	0.000106225
Mm.371602	0	0	2	0.00021245
Mm.11376	0	0	2	0.00021245
Mm.277891	0	0	1	0.000106225
Mm.3158	0	0	5	0.000531124
Mm.133851	0	0	1	0.000106225
Mm.19355	0	0	1	0.000106225
Mm.27796	0	0	1	0.000106225
Mm.313236	0	0	1	0.000106225
Mm.168680	0	0	4	0.000424899
Mm.140380	0	0	5	0.000531124
Mm.155033	0	0	1	0.000106225
Mm.218533	0	0	4	0.000424899
Mm.38460	0	0	1	0.000106225
Mm.249809	0	0	2	0.00021245
Mm.195628	0	0	1	0.000106225
Mm.28291	2	0.001004016	1	0.000106225
Mm.23825	0	0	2	0.00021245
Mm.295618	0	0	4	0.000424899
Mm.2050	0	0	5	0.000531124
Mm.350080	0	0	9	0.000956023
Mm.289669	0	0	1	0.000106225
Mm.345333	0	0	1	0.000106225
Mm.371622	1	0.000502008	1	0.000106225
Mm.5381	0	0	2	0.00021245
Mm.37617	1	0.000502008	0	0
Mm.275810	0	0	4	0.000424899
Mm.371624	1	0.000502008	2	0.00021245
Mm.289810	0	0	12	0.001274697
Mm.341719	1	0.000502008	7	0.000743573
Mm.21938	0	0	4	0.000424899
Mm.238817	0	0	2	0.00021245
Mm.290166	1	0.000502008	4	0.000424899
Mm.196635	1	0.000502008	0	0
Mm.280083	1	0.000502008	15	0.001593372
Mm.290786	0	0	11	0.001168472
Mm.339491	0	0	8	0.000849798
Mm.21724	0	0	2	0.00021245
Mm.107869	0	0	3	0.000318674
Mm.140568	0	0	5	0.000531124
Mm.236795	0	0	1	0.000106225
Mm.287443	0	0	1	0.000106225
Mm.27871	0	0	2	0.00021245
Mm.262707	0	0	1	0.000106225

Mm.288960	0	0	1	0.000106225					
Mm.180873	0	0	6	0.000637349					
Mm.371658	0	0	11	0.001168472					
Mm.13944	0	0	19	0.002018271					
Mm.199698	1	0.000502008	2	0.00021245					
Mm.218515	1	0.000502008	0	0					
Mm.34951	0	0	1	0.000106225					
Mm.121878	0	0	1	0.000106225					
Mm.41077	1	0.000502008	1	0.000106225					
Mm.288974	0	0	1	0.000106225					
Mm.214514	0	0	1	0.000106225					
Mm.270393	0	0	1	0.000106225					
Mm.29795	0	0	2	0.00021245					
Mm.271770	0	0	1	0.000106225					
Mm.2565	0	0	4	0.000424899					
>GO:0000278	58	0.040502793	108	0.014917127	0.368298724	2.14E-10	7.47E-08	mitotic cell cycle	P
Mm.31512	0	0	2	0.00021245					
Mm.307103	2	0.001004016	0	0					
Mm.286602	0	0	1	0.000106225					
Mm.181836	8	0.004016064	0	0					
Mm.290015	0	0	1	0.000106225					
Mm.289747	3	0.001506024	1	0.000106225					
Mm.29133	2	0.001004016	3	0.000318674					
Mm.168523	1	0.000502008	0	0					
Mm.4189	0	0	2	0.00021245					
Mm.260114	0	0	3	0.000318674					
Mm.22592	0	0	1	0.000106225					
Mm.2103	0	0	1	0.000106225					
Mm.281367	0	0	2	0.00021245					
Mm.12481	0	0	1	0.000106225					
Mm.143877	4	0.002008032	1	0.000106225					
Mm.29755	0	0	2	0.00021245					
Mm.116649	0	0	1	0.000106225					
Mm.56337	2	0.001004016	1	0.000106225					
Mm.182628	0	0	3	0.000318674					
Mm.42135	0	0	3	0.000318674					
Mm.290422	0	0	1	0.000106225					
Mm.332684	0	0	2	0.00021245					
Mm.315959	1	0.000502008	0	0					
Mm.37825	0	0	2	0.00021245					
Mm.29071	1	0.000502008	1	0.000106225					
Mm.26412	5	0.00251004	2	0.00021245					
Mm.24643	0	0	1	0.000106225					
Mm.318364	0	0	1	0.000106225					
Mm.273502	0	0	1	0.000106225					
Mm.172411	0	0	1	0.000106225					
Mm.6856	11	0.005522088	4	0.000424899					
Mm.272568	0	0	3	0.000318674					
Mm.24250	0	0	1	0.000106225					
Mm.24202	0	0	2	0.00021245					
Mm.257590	0	0	1	0.000106225					
Mm.89830	0	0	3	0.000318674					
Mm.257445	0	0	3	0.000318674					
Mm.206841	1	0.000502008	3	0.000318674					
Mm.291624	0	0	2	0.00021245					
Mm.328945	0	0	5	0.000531124					

Mm.28659	0	0	1	0.000106225					
Mm.271947	0	0	2	0.00021245					
Mm.273538	0	0	5	0.000531124					
Mm.24105	0	0	1	0.000106225					
Mm.193924	0	0	1	0.000106225					
Mm.273570	0	0	1	0.000106225					
Mm.272024	0	0	1	0.000106225					
Mm.89845	0	0	1	0.000106225					
Mm.257482	0	0	3	0.000318674					
Mm.292470	6	0.003012048	2	0.00021245					
Mm.21873	0	0	3	0.000318674					
Mm.104932	4	0.002008032	2	0.00021245					
Mm.22670	1	0.000502008	0	0					
Mm.235182	0	0	1	0.000106225					
Mm.153415	1	0.000502008	1	0.000106225					
Mm.331389	0	0	2	0.00021245					
Mm.285771	0	0	2	0.00021245					
Mm.44482	0	0	1	0.000106225					
Mm.150686	0	0	1	0.000106225					
Mm.34405	0	0	1	0.000106225					
Mm.925	1	0.000502008	0	0					
Mm.263913	0	0	3	0.000318674					
Mm.233734	0	0	1	0.000106225					
Mm.341972	0	0	3	0.000318674					
Mm.16753	4	0.002008032	1	0.000106225					
>GO:0007165	182	0.127094972	563	0.077762431	0.611845061	1.59E-09	5.02E-07	signal transduction	P
Mm.211287	0	0	1	0.000106225					
Mm.257404	0	0	1	0.000106225					
Mm.216227	0	0	1	0.000106225					
Mm.244236	1	0.000502008	0	0					
Mm.245513	0	0	2	0.00021245					
Mm.131150	2	0.001004016	2	0.00021245					
Mm.184163	1	0.000502008	2	0.00021245					
Mm.254144	0	0	3	0.000318674					
Mm.103522	0	0	1	0.000106225					
Mm.250265	0	0	1	0.000106225					
Mm.12177	1	0.000502008	2	0.00021245					
Mm.36241	0	0	3	0.000318674					
Mm.119717	1	0.000502008	1	0.000106225					
Mm.6839	0	0	3	0.000318674					
Mm.22680	0	0	1	0.000106225					
Mm.342315	0	0	3	0.000318674					
Mm.3596	1	0.000502008	1	0.000106225					
Mm.7405	1	0.000502008	0	0					
Mm.327835	0	0	1	0.000106225					
Mm.229532	1	0.000502008	0	0					
Mm.5126	0	0	1	0.000106225					
Mm.22673	0	0	1	0.000106225					
Mm.241282	0	0	2	0.00021245					
Mm.7996	2	0.001004016	0	0					
Mm.16340	0	0	2	0.00021245					
Mm.243722	0	0	4	0.000424899					
Mm.273292	1	0.000502008	0	0					
Mm.193925	0	0	4	0.000424899					
Mm.313181	2	0.001004016	0	0					
Mm.196464	1	0.000502008	1	0.000106225					

Mm.195898	0	0	2	0.00021245
Mm.125770	4	0.002008032	4	0.000424899
Mm.2344	0	0	1	0.000106225
Mm.5305	3	0.001506024	17	0.001805821
Mm.17604	0	0	1	0.000106225
Mm.234342	0	0	1	0.000106225
Mm.329700	0	0	1	0.000106225
Mm.140804	0	0	1	0.000106225
Mm.290834	0	0	1	0.000106225
Mm.279400	0	0	2	0.00021245
Mm.4364	0	0	3	0.000318674
Mm.4489	1	0.000502008	0	0
Mm.374793	0	0	1	0.000106225
Mm.248360	0	0	6	0.000637349
Mm.234003	0	0	1	0.000106225
Mm.213003	0	0	1	0.000106225
Mm.1403	0	0	1	0.000106225
Mm.33779	0	0	1	0.000106225
Mm.2537	1	0.000502008	0	0
Mm.256765	3	0.001506024	0	0
Mm.2380	0	0	2	0.00021245
Mm.254898	0	0	1	0.000106225
Mm.296814	0	0	1	0.000106225
Mm.10504	0	0	1	0.000106225
Mm.121361	0	0	2	0.00021245
Mm.260521	0	0	1	0.000106225
Mm.22724	0	0	2	0.00021245
Mm.38009	0	0	1	0.000106225
Mm.23963	0	0	4	0.000424899
Mm.45372	0	0	1	0.000106225
Mm.30039	1	0.000502008	0	0
Mm.25594	0	0	1	0.000106225
Mm.18509	0	0	1	0.000106225
Mm.8681	1	0.000502008	2	0.00021245
Mm.5236	0	0	1	0.000106225
Mm.28262	4	0.002008032	1	0.000106225
Mm.252210	0	0	1	0.000106225
Mm.6710	4	0.002008032	1	0.000106225
Mm.247564	0	0	1	0.000106225
Mm.293120	0	0	1	0.000106225
Mm.249934	0	0	5	0.000531124
Mm.8055	1	0.000502008	0	0
Mm.203747	1	0.000502008	0	0
Mm.24430	0	0	2	0.00021245
Mm.217362	1	0.000502008	0	0
Mm.219648	0	0	3	0.000318674
Mm.329277	0	0	3	0.000318674
Mm.328846	0	0	1	0.000106225
Mm.233813	0	0	3	0.000318674
Mm.289630	1	0.000502008	1	0.000106225
Mm.350516	0	0	20	0.002124495
Mm.277217	1	0.000502008	0	0
Mm.222810	0	0	1	0.000106225
Mm.373622	1	0.000502008	0	0
Mm.23978	0	0	2	0.00021245
Mm.137134	2	0.001004016	0	0

Mm.334648	0	0	2	0.00021245
Mm.196581	0	0	2	0.00021245
Mm.8385	0	0	3	0.000318674
Mm.21495	3	0.001506024	0	0
Mm.240396	0	0	1	0.000106225
Mm.259626	0	0	1	0.000106225
Mm.286753	0	0	2	0.00021245
Mm.332231	1	0.000502008	0	0
Mm.286600	0	0	2	0.00021245
Mm.258739	0	0	1	0.000106225
Mm.19169	0	0	3	0.000318674
Mm.91920	1	0.000502008	0	0
Mm.103728	0	0	2	0.00021245
Mm.248478	1	0.000502008	0	0
Mm.44606	0	0	1	0.000106225
Mm.334321	0	0	1	0.000106225
Mm.234472	0	0	1	0.000106225
Mm.103439	2	0.001004016	0	0
Mm.209349	0	0	1	0.000106225
Mm.23957	0	0	1	0.000106225
Mm.354761	0	0	2	0.00021245
Mm.273251	0	0	1	0.000106225
Mm.18635	0	0	1	0.000106225
Mm.242413	0	0	1	0.000106225
Mm.21876	1	0.000502008	0	0
Mm.272974	0	0	1	0.000106225
Mm.196208	0	0	1	0.000106225
Mm.264743	0	0	1	0.000106225
Mm.87046	0	0	1	0.000106225
Mm.370272	0	0	2	0.00021245
Mm.249586	1	0.000502008	0	0
Mm.162811	1	0.000502008	1	0.000106225
Mm.271944	0	0	5	0.000531124
Mm.132802	0	0	1	0.000106225
Mm.260376	1	0.000502008	0	0
Mm.1387	0	0	2	0.00021245
Mm.28158	0	0	1	0.000106225
Mm.24997	2	0.001004016	4	0.000424899
Mm.35691	0	0	1	0.000106225
Mm.266871	1	0.000502008	0	0
Mm.150	0	0	1	0.000106225
Mm.22119	0	0	1	0.000106225
Mm.233802	0	0	1	0.000106225
Mm.41417	0	0	1	0.000106225
Mm.309954	0	0	1	0.000106225
Mm.271976	0	0	2	0.00021245
Mm.88367	0	0	1	0.000106225
Mm.275742	2	0.001004016	0	0
Mm.247073	0	0	2	0.00021245
Mm.3401	1	0.000502008	0	0
Mm.151940	0	0	1	0.000106225
Mm.5090	0	0	1	0.000106225
Mm.224246	0	0	2	0.00021245
Mm.29891	4	0.002008032	2	0.00021245
Mm.221688	2	0.001004016	0	0
Mm.4509	1	0.000502008	1	0.000106225

Mm.154660	0	0	1	0.000106225
Mm.268521	0	0	1	0.000106225
Mm.288726	0	0	2	0.00021245
Mm.348266	0	0	1	0.000106225
Mm.39089	0	0	2	0.00021245
Mm.172346	1	0.000502008	1	0.000106225
Mm.29790	2	0.001004016	0	0
Mm.118034	1	0.000502008	0	0
Mm.237825	0	0	2	0.00021245
Mm.13806	0	0	2	0.00021245
Mm.7320	1	0.000502008	1	0.000106225
Mm.28733	0	0	4	0.000424899
Mm.223717	0	0	1	0.000106225
Mm.100399	1	0.000502008	1	0.000106225
Mm.22584	0	0	2	0.00021245
Mm.4913	1	0.000502008	0	0
Mm.945	0	0	1	0.000106225
Mm.29855	0	0	1	0.000106225
Mm.287425	0	0	1	0.000106225
Mm.332303	0	0	2	0.00021245
Mm.332936	0	0	1	0.000106225
Mm.277351	2	0.001004016	0	0
Mm.235018	0	0	1	0.000106225
Mm.192026	0	0	1	0.000106225
Mm.285993	3	0.001506024	9	0.000956023
Mm.329243	0	0	3	0.000318674
Mm.298775	0	0	3	0.000318674
Mm.28265	0	0	1	0.000106225
Mm.254629	0	0	1	0.000106225
Mm.93335	0	0	4	0.000424899
Mm.103354	2	0.001004016	0	0
Mm.320183	0	0	5	0.000531124
Mm.349120	0	0	1	0.000106225
Mm.83689	1	0.000502008	0	0
Mm.264860	0	0	1	0.000106225
Mm.29279	0	0	2	0.00021245
Mm.373635	1	0.000502008	5	0.000531124
Mm.70979	1	0.000502008	0	0
Mm.39863	0	0	1	0.000106225
Mm.298256	0	0	2	0.00021245
Mm.273142	0	0	2	0.00021245
Mm.159019	1	0.000502008	0	0
Mm.310036	0	0	2	0.00021245
Mm.325086	1	0.000502008	0	0
Mm.222685	1	0.000502008	0	0
Mm.44241	0	0	1	0.000106225
Mm.8004	1	0.000502008	0	0
Mm.228	0	0	1	0.000106225
Mm.201322	1	0.000502008	0	0
Mm.26150	2	0.001004016	2	0.00021245
Mm.248291	1	0.000502008	1	0.000106225
Mm.12091	1	0.000502008	2	0.00021245
Mm.21739	0	0	2	0.00021245
Mm.159724	0	0	1	0.000106225
Mm.293761	0	0	2	0.00021245
Mm.275574	1	0.000502008	0	0

Mm.228798	0	0	1	0.000106225
Mm.335520	1	0.000502008	0	0
Mm.275895	0	0	1	0.000106225
Mm.15969	0	0	2	0.00021245
Mm.757	2	0.001004016	2	0.00021245
Mm.254494	0	0	1	0.000106225
Mm.271674	0	0	3	0.000318674
Mm.6424	0	0	2	0.00021245
Mm.224825	1	0.000502008	0	0
Mm.208855	0	0	2	0.00021245
Mm.28854	1	0.000502008	0	0
Mm.272616	0	0	1	0.000106225
Mm.206764	1	0.000502008	4	0.000424899
Mm.7883	0	0	2	0.00021245
Mm.23684	0	0	1	0.000106225
Mm.291928	1	0.000502008	0	0
Mm.298893	1	0.000502008	0	0
Mm.51136	0	0	5	0.000531124
Mm.327442	0	0	1	0.000106225
Mm.321990	0	0	1	0.000106225
Mm.222723	1	0.000502008	0	0
Mm.103638	0	0	1	0.000106225
Mm.20593	2	0.001004016	2	0.00021245
Mm.55143	1	0.000502008	0	0
Mm.200770	0	0	1	0.000106225
Mm.87600	1	0.000502008	0	0
Mm.209989	0	0	1	0.000106225
Mm.26908	0	0	8	0.000849798
Mm.348326	0	0	1	0.000106225
Mm.275839	2	0.001004016	0	0
Mm.27804	0	0	2	0.00021245
Mm.336104	0	0	2	0.00021245
Mm.374904	0	0	1	0.000106225
Mm.257073	1	0.000502008	0	0
Mm.252718	0	0	1	0.000106225
Mm.124502	9	0.004518072	0	0
Mm.309296	1	0.000502008	0	0
Mm.5159	0	0	5	0.000531124
Mm.90218	0	0	2	0.00021245
Mm.280125	0	0	1	0.000106225
Mm.21974	0	0	1	0.000106225
Mm.272203	0	0	2	0.00021245
Mm.6900	0	0	1	0.000106225
Mm.289657	2	0.001004016	1	0.000106225
Mm.271947	0	0	2	0.00021245
Mm.265350	2	0.001004016	0	0
Mm.29872	0	0	1	0.000106225
Mm.3810	0	0	2	0.00021245
Mm.333471	0	0	1	0.000106225
Mm.259333	3	0.001506024	0	0
Mm.38370	0	0	1	0.000106225
Mm.291554	1	0.000502008	0	0
Mm.329993	0	0	1	0.000106225
Mm.212039	1	0.000502008	1	0.000106225
Mm.292510	1	0.000502008	1	0.000106225
Mm.86595	0	0	1	0.000106225

Mm.20755	1	0.000502008	0	0
Mm.229287	0	0	1	0.000106225
Mm.262067	0	0	1	0.000106225
Mm.227642	3	0.001506024	1	0.000106225
Mm.29515	0	0	2	0.00021245
Mm.234700	0	0	6	0.000637349
Mm.275127	0	0	1	0.000106225
Mm.274346	0	0	1	0.000106225
Mm.279603	0	0	1	0.000106225
Mm.316592	0	0	4	0.000424899
Mm.273804	0	0	2	0.00021245
Mm.46014	0	0	2	0.00021245
Mm.202606	0	0	2	0.00021245
Mm.244068	0	0	1	0.000106225
Mm.28015	1	0.000502008	0	0
Mm.28489	0	0	1	0.000106225
Mm.277354	0	0	1	0.000106225
Mm.89515	1	0.000502008	2	0.00021245
Mm.252171	1	0.000502008	2	0.00021245
Mm.28251	0	0	4	0.000424899
Mm.28196	0	0	1	0.000106225
Mm.273379	0	0	5	0.000531124
Mm.275266	0	0	3	0.000318674
Mm.44490	0	0	3	0.000318674
Mm.272078	0	0	2	0.00021245
Mm.196692	1	0.000502008	0	0
Mm.28376	0	0	2	0.00021245
Mm.307022	0	0	5	0.000531124
Mm.227583	2	0.001004016	1	0.000106225
Mm.293628	1	0.000502008	0	0
Mm.330160	0	0	13	0.001380922
Mm.214593	0	0	1	0.000106225
Mm.281298	0	0	1	0.000106225
Mm.330501	1	0.000502008	0	0
Mm.28278	0	0	2	0.00021245
Mm.292040	0	0	3	0.000318674
Mm.286006	1	0.000502008	0	0
Mm.308180	0	0	3	0.000318674
Mm.173337	0	0	1	0.000106225
Mm.203952	0	0	1	0.000106225
Mm.244393	0	0	1	0.000106225
Mm.22548	0	0	1	0.000106225
Mm.3996	0	0	3	0.000318674
Mm.290924	1	0.000502008	0	0
Mm.318430	0	0	1	0.000106225
Mm.212525	1	0.000502008	0	0
Mm.147226	0	0	1	0.000106225
Mm.1457	0	0	2	0.00021245
Mm.826	3	0.001506024	0	0
Mm.291247	0	0	2	0.00021245
Mm.333868	3	0.001506024	0	0
Mm.371546	1	0.000502008	8	0.000849798
Mm.27701	0	0	1	0.000106225
Mm.35059	0	0	1	0.000106225
Mm.12723	0	0	1	0.000106225
Mm.34867	0	0	1	0.000106225

Mm.1022	0	0	10	0.001062248					
Mm.268397	0	0	1	0.000106225					
Mm.153226	0	0	7	0.000743573					
Mm.334313	0	0	1	0.000106225					
Mm.256975	0	0	2	0.00021245					
Mm.35727	0	0	1	0.000106225					
Mm.28650	2	0.001004016	0	0					
Mm.333233	0	0	1	0.000106225					
Mm.3903	0	0	2	0.00021245					
Mm.322186	0	0	1	0.000106225					
Mm.233009	1	0.000502008	1	0.000106225					
Mm.21936	0	0	1	0.000106225					
Mm.22413	0	0	1	0.000106225					
Mm.290530	9	0.004518072	11	0.001168472					
Mm.329123	0	0	1	0.000106225					
Mm.207619	0	0	1	0.000106225					
Mm.27968	0	0	1	0.000106225					
Mm.22085	0	0	1	0.000106225					
Mm.25306	0	0	3	0.000318674					
Mm.27832	0	0	1	0.000106225					
Mm.276572	1	0.000502008	0	0					
Mm.31486	3	0.001506024	0	0					
Mm.198264	2	0.001004016	2	0.00021245					
Mm.276669	0	0	2	0.00021245					
Mm.179267	0	0	3	0.000318674					
Mm.245675	0	0	1	0.000106225					
Mm.41637	0	0	1	0.000106225					
Mm.325524	0	0	1	0.000106225					
Mm.271898	0	0	5	0.000531124					
Mm.247473	1	0.000502008	3	0.000318674					
Mm.197534	0	0	1	0.000106225					
Mm.343951	0	0	1	0.000106225					
Mm.218891	1	0.000502008	0	0					
Mm.3057	1	0.000502008	0	0					
Mm.221440	1	0.000502008	1	0.000106225					
Mm.315593	2	0.001004016	0	0					
Mm.331970	2	0.001004016	1	0.000106225					
Mm.187079	0	0	2	0.00021245					
Mm.236009	1	0.000502008	0	0					
Mm.7454	0	0	5	0.000531124					
Mm.42038	0	0	2	0.00021245					
>GO:0007154	217	0.151536313	708	0.097790055	0.645324236	2.32E-09	5.79E-07	cell communication	P
Mm.338720	0	0	2	0.00021245					
Mm.24615	0	0	4	0.000424899					
Mm.26859	0	0	3	0.000318674					
Mm.22398	2	0.001004016	0	0					
Mm.291120	0	0	2	0.00021245					
Mm.151819	0	0	1	0.000106225					
Mm.335520	1	0.000502008	0	0					
Mm.265985	0	0	3	0.000318674					
Mm.757	2	0.001004016	2	0.00021245					
Mm.235204	1	0.000502008	0	0					
Mm.18962	0	0	1	0.000106225					
Mm.291928	1	0.000502008	0	0					
Mm.35738	0	0	2	0.00021245					
Mm.29798	0	0	1	0.000106225					

Mm.297964	0	0	1	0.000106225
Mm.330428	2	0.001004016	0	0
Mm.210676	0	0	5	0.000531124
Mm.35605	1	0.000502008	2	0.00021245
Mm.257437	1	0.000502008	1	0.000106225
Mm.22680	0	0	1	0.000106225
Mm.3819	0	0	1	0.000106225
Mm.4352	0	0	1	0.000106225
Mm.249555	0	0	4	0.000424899
Mm.181021	0	0	4	0.000424899
Mm.2509	1	0.000502008	2	0.00021245
Mm.277792	0	0	1	0.000106225
Mm.3758	0	0	1	0.000106225
Mm.336625	0	0	2	0.00021245
Mm.125580	0	0	1	0.000106225
Mm.193099	0	0	1	0.000106225
Mm.3982	0	0	1	0.000106225
Mm.291831	1	0.000502008	0	0
Mm.6424	0	0	2	0.00021245
Mm.294882	0	0	1	0.000106225
Mm.12508	0	0	7	0.000743573
Mm.312059	0	0	6	0.000637349
Mm.42012	0	0	1	0.000106225
Mm.172674	1	0.000502008	4	0.000424899
Mm.1451	0	0	4	0.000424899
Mm.60590	0	0	2	0.00021245
Mm.4974	0	0	1	0.000106225
Mm.258759	1	0.000502008	0	0
Mm.18503	1	0.000502008	0	0
Mm.93335	0	0	4	0.000424899
Mm.343951	0	0	1	0.000106225
Mm.234912	0	0	2	0.00021245
Mm.316080	0	0	1	0.000106225
Mm.220821	0	0	1	0.000106225
Mm.29855	0	0	1	0.000106225
Mm.292510	1	0.000502008	1	0.000106225
Mm.3057	1	0.000502008	0	0
Mm.3337	1	0.000502008	0	0
Mm.332590	0	0	2	0.00021245
Mm.288474	3	0.001506024	1	0.000106225
Mm.151293	0	0	3	0.000318674
Mm.14455	0	0	1	0.000106225
Mm.210018	0	0	2	0.00021245
Mm.3509	0	0	1	0.000106225
Mm.1249	0	0	1	0.000106225
Mm.302274	0	0	1	0.000106225
Mm.279437	0	0	3	0.000318674
Mm.334648	0	0	2	0.00021245
Mm.103668	1	0.000502008	0	0
Mm.151931	2	0.001004016	0	0
Mm.27291	0	0	3	0.000318674
Mm.269815	4	0.002008032	3	0.000318674
Mm.357762	1	0.000502008	0	0
Mm.218891	1	0.000502008	0	0
Mm.31927	1	0.000502008	1	0.000106225
Mm.257482	0	0	3	0.000318674

Mm.143763	0	0	1	0.000106225
Mm.328072	1	0.000502008	1	0.000106225
Mm.38993	0	0	1	0.000106225
Mm.292107	0	0	1	0.000106225
Mm.29080	0	0	1	0.000106225
Mm.119714	0	0	1	0.000106225
Mm.313558	0	0	1	0.000106225
Mm.234823	1	0.000502008	0	0
Mm.272278	0	0	3	0.000318674
Mm.28770	0	0	4	0.000424899
Mm.247203	0	0	1	0.000106225
Mm.200783	0	0	3	0.000318674
Mm.275054	0	0	1	0.000106225
Mm.57734	1	0.000502008	0	0
Mm.298798	0	0	1	0.000106225
Mm.374780	0	0	1	0.000106225
Mm.15969	0	0	2	0.00021245
Mm.27365	2	0.001004016	0	0
Mm.299254	1	0.000502008	1	0.000106225
Mm.371723	0	0	2	0.00021245
Mm.10516	0	0	1	0.000106225
Mm.272130	0	0	2	0.00021245
Mm.273270	1	0.000502008	0	0
Mm.281003	0	0	1	0.000106225
Mm.211287	0	0	1	0.000106225
Mm.257404	0	0	1	0.000106225
Mm.216227	0	0	1	0.000106225
Mm.244236	1	0.000502008	0	0
Mm.245513	0	0	2	0.00021245
Mm.131150	2	0.001004016	2	0.00021245
Mm.184163	1	0.000502008	2	0.00021245
Mm.254144	0	0	3	0.000318674
Mm.103522	0	0	1	0.000106225
Mm.250265	0	0	1	0.000106225
Mm.12177	1	0.000502008	2	0.00021245
Mm.36241	0	0	3	0.000318674
Mm.119717	1	0.000502008	1	0.000106225
Mm.6839	0	0	3	0.000318674
Mm.342315	0	0	3	0.000318674
Mm.3596	1	0.000502008	1	0.000106225
Mm.7405	1	0.000502008	0	0
Mm.327835	0	0	1	0.000106225
Mm.229532	1	0.000502008	0	0
Mm.5126	0	0	1	0.000106225
Mm.22673	0	0	1	0.000106225
Mm.241282	0	0	2	0.00021245
Mm.7996	2	0.001004016	0	0
Mm.16340	0	0	2	0.00021245
Mm.243722	0	0	4	0.000424899
Mm.273292	1	0.000502008	0	0
Mm.193925	0	0	4	0.000424899
Mm.313181	2	0.001004016	0	0
Mm.196464	1	0.000502008	1	0.000106225
Mm.195898	0	0	2	0.00021245
Mm.125770	4	0.002008032	4	0.000424899
Mm.2344	0	0	1	0.000106225

Mm.5305	3	0.001506024	17	0.001805821
Mm.17604	0	0	1	0.000106225
Mm.234342	0	0	1	0.000106225
Mm.329700	0	0	1	0.000106225
Mm.140804	0	0	1	0.000106225
Mm.290834	0	0	1	0.000106225
Mm.279400	0	0	2	0.00021245
Mm.4364	0	0	3	0.000318674
Mm.4489	1	0.000502008	0	0
Mm.374793	0	0	1	0.000106225
Mm.248360	0	0	6	0.000637349
Mm.234003	0	0	1	0.000106225
Mm.213003	0	0	1	0.000106225
Mm.1403	0	0	1	0.000106225
Mm.33779	0	0	1	0.000106225
Mm.2537	1	0.000502008	0	0
Mm.256765	3	0.001506024	0	0
Mm.2380	0	0	2	0.00021245
Mm.254898	0	0	1	0.000106225
Mm.296814	0	0	1	0.000106225
Mm.10504	0	0	1	0.000106225
Mm.121361	0	0	2	0.00021245
Mm.260521	0	0	1	0.000106225
Mm.22724	0	0	2	0.00021245
Mm.38009	0	0	1	0.000106225
Mm.23963	0	0	4	0.000424899
Mm.45372	0	0	1	0.000106225
Mm.30039	1	0.000502008	0	0
Mm.25594	0	0	1	0.000106225
Mm.18509	0	0	1	0.000106225
Mm.8681	1	0.000502008	2	0.00021245
Mm.5236	0	0	1	0.000106225
Mm.28262	4	0.002008032	1	0.000106225
Mm.252210	0	0	1	0.000106225
Mm.6710	4	0.002008032	1	0.000106225
Mm.247564	0	0	1	0.000106225
Mm.293120	0	0	1	0.000106225
Mm.249934	0	0	5	0.000531124
Mm.8055	1	0.000502008	0	0
Mm.203747	1	0.000502008	0	0
Mm.24430	0	0	2	0.00021245
Mm.217362	1	0.000502008	0	0
Mm.219648	0	0	3	0.000318674
Mm.329277	0	0	3	0.000318674
Mm.328846	0	0	1	0.000106225
Mm.233813	0	0	3	0.000318674
Mm.289630	1	0.000502008	1	0.000106225
Mm.350516	0	0	20	0.002124495
Mm.277217	1	0.000502008	0	0
Mm.222810	0	0	1	0.000106225
Mm.373622	1	0.000502008	0	0
Mm.23978	0	0	2	0.00021245
Mm.137134	2	0.001004016	0	0
Mm.196581	0	0	2	0.00021245
Mm.8385	0	0	3	0.000318674
Mm.21495	3	0.001506024	0	0

Mm.240396	0	0	1	0.000106225
Mm.259626	0	0	1	0.000106225
Mm.286753	0	0	2	0.00021245
Mm.332231	1	0.000502008	0	0
Mm.286600	0	0	2	0.00021245
Mm.258739	0	0	1	0.000106225
Mm.19169	0	0	3	0.000318674
Mm.91920	1	0.000502008	0	0
Mm.103728	0	0	2	0.00021245
Mm.248478	1	0.000502008	0	0
Mm.44606	0	0	1	0.000106225
Mm.334321	0	0	1	0.000106225
Mm.234472	0	0	1	0.000106225
Mm.103439	2	0.001004016	0	0
Mm.209349	0	0	1	0.000106225
Mm.23957	0	0	1	0.000106225
Mm.354761	0	0	2	0.00021245
Mm.273251	0	0	1	0.000106225
Mm.18635	0	0	1	0.000106225
Mm.242413	0	0	1	0.000106225
Mm.21876	1	0.000502008	0	0
Mm.272974	0	0	1	0.000106225
Mm.196208	0	0	1	0.000106225
Mm.264743	0	0	1	0.000106225
Mm.87046	0	0	1	0.000106225
Mm.370272	0	0	2	0.00021245
Mm.249586	1	0.000502008	0	0
Mm.162811	1	0.000502008	1	0.000106225
Mm.271944	0	0	5	0.000531124
Mm.132802	0	0	1	0.000106225
Mm.260376	1	0.000502008	0	0
Mm.1387	0	0	2	0.00021245
Mm.28158	0	0	1	0.000106225
Mm.24997	2	0.001004016	4	0.000424899
Mm.35691	0	0	1	0.000106225
Mm.266871	1	0.000502008	0	0
Mm.150	0	0	1	0.000106225
Mm.22119	0	0	1	0.000106225
Mm.233802	0	0	1	0.000106225
Mm.41417	0	0	1	0.000106225
Mm.309954	0	0	1	0.000106225
Mm.271976	0	0	2	0.00021245
Mm.88367	0	0	1	0.000106225
Mm.275742	2	0.001004016	0	0
Mm.247073	0	0	2	0.00021245
Mm.3401	1	0.000502008	0	0
Mm.151940	0	0	1	0.000106225
Mm.5090	0	0	1	0.000106225
Mm.224246	0	0	2	0.00021245
Mm.29891	4	0.002008032	2	0.00021245
Mm.221688	2	0.001004016	0	0
Mm.4509	1	0.000502008	1	0.000106225
Mm.154660	0	0	1	0.000106225
Mm.268521	0	0	1	0.000106225
Mm.288726	0	0	2	0.00021245
Mm.348266	0	0	1	0.000106225

Mm.39089	0	0	2	0.00021245
Mm.172346	1	0.000502008	1	0.000106225
Mm.29790	2	0.001004016	0	0
Mm.118034	1	0.000502008	0	0
Mm.237825	0	0	2	0.00021245
Mm.13806	0	0	2	0.00021245
Mm.7320	1	0.000502008	1	0.000106225
Mm.28733	0	0	4	0.000424899
Mm.223717	0	0	1	0.000106225
Mm.100399	1	0.000502008	1	0.000106225
Mm.22584	0	0	2	0.00021245
Mm.4913	1	0.000502008	0	0
Mm.945	0	0	1	0.000106225
Mm.287425	0	0	1	0.000106225
Mm.332303	0	0	2	0.00021245
Mm.332936	0	0	1	0.000106225
Mm.277351	2	0.001004016	0	0
Mm.235018	0	0	1	0.000106225
Mm.192026	0	0	1	0.000106225
Mm.285993	3	0.001506024	9	0.000956023
Mm.329243	0	0	3	0.000318674
Mm.298775	0	0	3	0.000318674
Mm.28265	0	0	1	0.000106225
Mm.254629	0	0	1	0.000106225
Mm.103354	2	0.001004016	0	0
Mm.320183	0	0	5	0.000531124
Mm.349120	0	0	1	0.000106225
Mm.83689	1	0.000502008	0	0
Mm.264860	0	0	1	0.000106225
Mm.29279	0	0	2	0.00021245
Mm.373635	1	0.000502008	5	0.000531124
Mm.70979	1	0.000502008	0	0
Mm.39863	0	0	1	0.000106225
Mm.298256	0	0	2	0.00021245
Mm.273142	0	0	2	0.00021245
Mm.159019	1	0.000502008	0	0
Mm.310036	0	0	2	0.00021245
Mm.325086	1	0.000502008	0	0
Mm.222685	1	0.000502008	0	0
Mm.44241	0	0	1	0.000106225
Mm.8004	1	0.000502008	0	0
Mm.228	0	0	1	0.000106225
Mm.201322	1	0.000502008	0	0
Mm.26150	2	0.001004016	2	0.00021245
Mm.248291	1	0.000502008	1	0.000106225
Mm.12091	1	0.000502008	2	0.00021245
Mm.21739	0	0	2	0.00021245
Mm.159724	0	0	1	0.000106225
Mm.293761	0	0	2	0.00021245
Mm.275574	1	0.000502008	0	0
Mm.228798	0	0	1	0.000106225
Mm.275895	0	0	1	0.000106225
Mm.254494	0	0	1	0.000106225
Mm.271674	0	0	3	0.000318674
Mm.224825	1	0.000502008	0	0
Mm.208855	0	0	2	0.00021245

Mm.28854	1	0.000502008	0	0
Mm.272616	0	0	1	0.000106225
Mm.206764	1	0.000502008	4	0.000424899
Mm.7883	0	0	2	0.00021245
Mm.23684	0	0	1	0.000106225
Mm.298893	1	0.000502008	0	0
Mm.51136	0	0	5	0.000531124
Mm.327442	0	0	1	0.000106225
Mm.321990	0	0	1	0.000106225
Mm.222723	1	0.000502008	0	0
Mm.103638	0	0	1	0.000106225
Mm.20593	2	0.001004016	2	0.00021245
Mm.55143	1	0.000502008	0	0
Mm.200770	0	0	1	0.000106225
Mm.87600	1	0.000502008	0	0
Mm.209989	0	0	1	0.000106225
Mm.26908	0	0	8	0.000849798
Mm.348326	0	0	1	0.000106225
Mm.275839	2	0.001004016	0	0
Mm.27804	0	0	2	0.00021245
Mm.336104	0	0	2	0.00021245
Mm.374904	0	0	1	0.000106225
Mm.257073	1	0.000502008	0	0
Mm.252718	0	0	1	0.000106225
Mm.124502	9	0.004518072	0	0
Mm.309296	1	0.000502008	0	0
Mm.5159	0	0	5	0.000531124
Mm.90218	0	0	2	0.00021245
Mm.280125	0	0	1	0.000106225
Mm.21974	0	0	1	0.000106225
Mm.272203	0	0	2	0.00021245
Mm.6900	0	0	1	0.000106225
Mm.289657	2	0.001004016	1	0.000106225
Mm.271947	0	0	2	0.00021245
Mm.265350	2	0.001004016	0	0
Mm.29872	0	0	1	0.000106225
Mm.3810	0	0	2	0.00021245
Mm.333471	0	0	1	0.000106225
Mm.259333	3	0.001506024	0	0
Mm.38370	0	0	1	0.000106225
Mm.291554	1	0.000502008	0	0
Mm.329993	0	0	1	0.000106225
Mm.212039	1	0.000502008	1	0.000106225
Mm.86595	0	0	1	0.000106225
Mm.20755	1	0.000502008	0	0
Mm.229287	0	0	1	0.000106225
Mm.262067	0	0	1	0.000106225
Mm.227642	3	0.001506024	1	0.000106225
Mm.29515	0	0	2	0.00021245
Mm.234700	0	0	6	0.000637349
Mm.275127	0	0	1	0.000106225
Mm.274346	0	0	1	0.000106225
Mm.279603	0	0	1	0.000106225
Mm.316592	0	0	4	0.000424899
Mm.273804	0	0	2	0.00021245
Mm.46014	0	0	2	0.00021245

Mm.202606	0	0	2	0.00021245
Mm.244068	0	0	1	0.000106225
Mm.28015	1	0.000502008	0	0
Mm.28489	0	0	1	0.000106225
Mm.277354	0	0	1	0.000106225
Mm.89515	1	0.000502008	2	0.00021245
Mm.252171	1	0.000502008	2	0.00021245
Mm.28251	0	0	4	0.000424899
Mm.28196	0	0	1	0.000106225
Mm.273379	0	0	5	0.000531124
Mm.275266	0	0	3	0.000318674
Mm.44490	0	0	3	0.000318674
Mm.272078	0	0	2	0.00021245
Mm.196692	1	0.000502008	0	0
Mm.28376	0	0	2	0.00021245
Mm.307022	0	0	5	0.000531124
Mm.227583	2	0.001004016	1	0.000106225
Mm.293628	1	0.000502008	0	0
Mm.330160	0	0	13	0.001380922
Mm.214593	0	0	1	0.000106225
Mm.281298	0	0	1	0.000106225
Mm.330501	1	0.000502008	0	0
Mm.28278	0	0	2	0.00021245
Mm.292040	0	0	3	0.000318674
Mm.286006	1	0.000502008	0	0
Mm.308180	0	0	3	0.000318674
Mm.173337	0	0	1	0.000106225
Mm.203952	0	0	1	0.000106225
Mm.244393	0	0	1	0.000106225
Mm.22548	0	0	1	0.000106225
Mm.3996	0	0	3	0.000318674
Mm.290924	1	0.000502008	0	0
Mm.318430	0	0	1	0.000106225
Mm.212525	1	0.000502008	0	0
Mm.147226	0	0	1	0.000106225
Mm.1457	0	0	2	0.00021245
Mm.826	3	0.001506024	0	0
Mm.291247	0	0	2	0.00021245
Mm.333868	3	0.001506024	0	0
Mm.371546	1	0.000502008	8	0.000849798
Mm.27701	0	0	1	0.000106225
Mm.35059	0	0	1	0.000106225
Mm.12723	0	0	1	0.000106225
Mm.34867	0	0	1	0.000106225
Mm.1022	0	0	10	0.001062248
Mm.268397	0	0	1	0.000106225
Mm.153226	0	0	7	0.000743573
Mm.334313	0	0	1	0.000106225
Mm.256975	0	0	2	0.00021245
Mm.35727	0	0	1	0.000106225
Mm.28650	2	0.001004016	0	0
Mm.333233	0	0	1	0.000106225
Mm.3903	0	0	2	0.00021245
Mm.322186	0	0	1	0.000106225
Mm.233009	1	0.000502008	1	0.000106225
Mm.21936	0	0	1	0.000106225

Mm.22413	0	0	1	0.000106225					
Mm.290530	9	0.004518072	11	0.001168472					
Mm.329123	0	0	1	0.000106225					
Mm.207619	0	0	1	0.000106225					
Mm.27968	0	0	1	0.000106225					
Mm.22085	0	0	1	0.000106225					
Mm.25306	0	0	3	0.000318674					
Mm.27832	0	0	1	0.000106225					
Mm.276572	1	0.000502008	0	0					
Mm.31486	3	0.001506024	0	0					
Mm.198264	2	0.001004016	2	0.00021245					
Mm.276669	0	0	2	0.00021245					
Mm.179267	0	0	3	0.000318674					
Mm.245675	0	0	1	0.000106225					
Mm.41637	0	0	1	0.000106225					
Mm.325524	0	0	1	0.000106225					
Mm.271898	0	0	5	0.000531124					
Mm.247473	1	0.000502008	3	0.000318674					
Mm.197534	0	0	1	0.000106225					
Mm.221440	1	0.000502008	1	0.000106225					
Mm.315593	2	0.001004016	0	0					
Mm.331970	2	0.001004016	1	0.000106225					
Mm.187079	0	0	2	0.00021245					
Mm.236009	1	0.000502008	0	0					
Mm.7454	0	0	5	0.000531124					
Mm.42038	0	0	2	0.00021245					
Mm.321227	0	0	1	0.000106225					
Mm.41035	1	0.000502008	2	0.00021245					
Mm.3555	0	0	1	0.000106225					
Mm.227704	0	0	1	0.000106225					
Mm.113877	1	0.000502008	0	0					
Mm.257035	1	0.000502008	0	0					
Mm.255607	0	0	3	0.000318674					
Mm.331182	0	0	1	0.000106225					
Mm.93636	0	0	1	0.000106225					
Mm.7060	0	0	2	0.00021245					
Mm.31597	0	0	3	0.000318674					
>GO:0043228	141	0.098463687	1161	0.160359116	1.628611732	2.67E-09	5.81E-07	non-membrane-bound orga	C
Mm.288460	0	0	2	0.00021245					
Mm.150231	0	0	1	0.000106225					
Mm.290563	0	0	3	0.000318674					
Mm.24350	0	0	1	0.000106225					
Mm.352429	1	0.000502008	0	0					
Mm.371563	5	0.00251004	11	0.001168472					
Mm.313345	0	0	1	0.000106225					
Mm.10027	0	0	1	0.000106225					
Mm.274770	0	0	1	0.000106225					
Mm.159684	1	0.000502008	2	0.00021245					
Mm.245739	0	0	1	0.000106225					
Mm.26412	5	0.00251004	2	0.00021245					
Mm.133101	1	0.000502008	1	0.000106225					
Mm.283802	0	0	1	0.000106225					
Mm.280717	1	0.000502008	0	0					
Mm.117541	0	0	5	0.000531124					
Mm.281005	0	0	1	0.000106225					
Mm.206841	1	0.000502008	3	0.000318674					

Mm.288015	2	0.001004016	0	0
Mm.89568	0	0	3	0.000318674
Mm.182628	0	0	3	0.000318674
Mm.279751	0	0	3	0.000318674
Mm.10141	2	0.001004016	4	0.000424899
Mm.328945	0	0	5	0.000531124
Mm.254784	0	0	1	0.000106225
Mm.333357	0	0	2	0.00021245
Mm.4071	0	0	11	0.001168472
Mm.29055	0	0	11	0.001168472
Mm.262059	0	0	1	0.000106225
Mm.5001	0	0	1	0.000106225
Mm.311655	1	0.000502008	1	0.000106225
Mm.9244	0	0	1	0.000106225
Mm.32019	0	0	3	0.000318674
Mm.305561	0	0	3	0.000318674
Mm.13433	0	0	2	0.00021245
Mm.128273	2	0.001004016	0	0
Mm.259893	0	0	3	0.000318674
Mm.278578	0	0	1	0.000106225
Mm.247844	0	0	2	0.00021245
Mm.29755	0	0	2	0.00021245
Mm.272226	1	0.000502008	0	0
Mm.16711	0	0	6	0.000637349
Mm.3411	0	0	3	0.000318674
Mm.29709	3	0.001506024	0	0
Mm.129746	1	0.000502008	0	0
Mm.8552	0	0	4	0.000424899
Mm.161470	1	0.000502008	1	0.000106225
Mm.29133	2	0.001004016	3	0.000318674
Mm.15755	0	0	2	0.00021245
Mm.273502	0	0	1	0.000106225
Mm.215389	0	0	2	0.00021245
Mm.24250	0	0	1	0.000106225
Mm.151315	0	0	1	0.000106225
Mm.195770	0	0	1	0.000106225
Mm.132238	0	0	2	0.00021245
Mm.333388	0	0	1	0.000106225
Mm.370289	0	0	1	0.000106225
Mm.8137	0	0	2	0.00021245
Mm.2756	0	0	1	0.000106225
Mm.319660	0	0	1	0.000106225
Mm.22522	0	0	2	0.00021245
Mm.7142	0	0	2	0.00021245
Mm.24248	0	0	1	0.000106225
Mm.15701	0	0	7	0.000743573
Mm.246237	0	0	1	0.000106225
Mm.298443	2	0.001004016	0	0
Mm.29680	1	0.000502008	1	0.000106225
Mm.152466	0	0	1	0.000106225
Mm.21899	0	0	5	0.000531124
Mm.182776	0	0	1	0.000106225
Mm.260893	0	0	2	0.00021245
Mm.128580	0	0	1	0.000106225
Mm.19806	0	0	3	0.000318674
Mm.348392	0	0	2	0.00021245

Mm.160061	0	0	2	0.00021245
Mm.4237	0	0	2	0.00021245
Mm.28659	0	0	1	0.000106225
Mm.180734	1	0.000502008	4	0.000424899
Mm.257590	0	0	1	0.000106225
Mm.7141	0	0	2	0.00021245
Mm.2903	0	0	1	0.000106225
Mm.27705	1	0.000502008	0	0
Mm.16549	0	0	2	0.00021245
Mm.370283	0	0	3	0.000318674
Mm.148877	0	0	1	0.000106225
Mm.332739	0	0	1	0.000106225
Mm.12553	1	0.000502008	0	0
Mm.12864	0	0	1	0.000106225
Mm.290015	0	0	1	0.000106225
Mm.4283	0	0	1	0.000106225
Mm.282719	0	0	3	0.000318674
Mm.22670	1	0.000502008	0	0
Mm.154378	0	0	6	0.000637349
Mm.338720	0	0	2	0.00021245
Mm.6343	3	0.001506024	17	0.001805821
Mm.28764	5	0.00251004	3	0.000318674
Mm.2845	0	0	3	0.000318674
Mm.2215	0	0	2	0.00021245
Mm.297196	1	0.000502008	0	0
Mm.250030	0	0	11	0.001168472
Mm.188413	0	0	2	0.00021245
Mm.88512	0	0	2	0.00021245
Mm.29810	0	0	2	0.00021245
Mm.220367	0	0	1	0.000106225
Mm.25264	0	0	1	0.000106225
Mm.257482	0	0	3	0.000318674
Mm.28630	0	0	2	0.00021245
Mm.140380	0	0	5	0.000531124
Mm.227258	0	0	4	0.000424899
Mm.280311	0	0	1	0.000106225
Mm.271715	0	0	1	0.000106225
Mm.29906	0	0	2	0.00021245
Mm.78861	0	0	4	0.000424899
Mm.33437	0	0	2	0.00021245
Mm.210845	0	0	7	0.000743573
Mm.284592	0	0	2	0.00021245
Mm.358640	0	0	1	0.000106225
Mm.244820	0	0	4	0.000424899
Mm.100113	0	0	16	0.001699596
Mm.371669	0	0	2	0.00021245
Mm.88216	0	0	2	0.00021245
Mm.274995	0	0	3	0.000318674
Mm.306162	0	0	1	0.000106225
Mm.246693	0	0	3	0.000318674
Mm.275158	0	0	1	0.000106225
Mm.3458	0	0	1	0.000106225
Mm.317557	0	0	2	0.00021245
Mm.288730	0	0	1	0.000106225
Mm.279861	0	0	2	0.00021245
Mm.38344	0	0	2	0.00021245

Mm.56337	2	0.001004016	1	0.000106225
Mm.251794	0	0	5	0.000531124
Mm.359653	1	0.000502008	3	0.000318674
Mm.89845	0	0	1	0.000106225
Mm.212525	1	0.000502008	0	0
Mm.24643	0	0	1	0.000106225
Mm.24105	0	0	1	0.000106225
Mm.31979	0	0	1	0.000106225
Mm.12481	0	0	1	0.000106225
Mm.358671	0	0	1	0.000106225
Mm.2444	0	0	1	0.000106225
Mm.273862	0	0	1	0.000106225
Mm.301827	0	0	1	0.000106225
Mm.188432	6	0.003012048	12	0.001274697
Mm.227274	0	0	2	0.00021245
Mm.311337	0	0	3	0.000318674
Mm.361980	0	0	14	0.001487147
Mm.298467	0	0	1	0.000106225
Mm.371545	0	0	7	0.000743573
Mm.5286	0	0	23	0.00244317
Mm.217354	0	0	1	0.000106225
Mm.329631	0	0	1	0.000106225
Mm.328846	0	0	1	0.000106225
Mm.336955	0	0	1	0.000106225
Mm.347060	1	0.000502008	4	0.000424899
Mm.349277	0	0	8	0.000849798
Mm.372072	0	0	3	0.000318674
Mm.309019	0	0	1	0.000106225
Mm.29046	0	0	2	0.00021245
Mm.316592	0	0	4	0.000424899
Mm.307846	0	0	4	0.000424899
Mm.306548	0	0	1	0.000106225
Mm.300263	0	0	3	0.000318674
Mm.325521	0	0	5	0.000531124
Mm.247113	0	0	3	0.000318674
Mm.358632	0	0	1	0.000106225
Mm.104368	0	0	6	0.000637349
Mm.21529	1	0.000502008	1	0.000106225
Mm.371576	0	0	1	0.000106225
Mm.323357	0	0	4	0.000424899
Mm.354330	0	0	10	0.001062248
Mm.4419	0	0	1	0.000106225
Mm.262021	0	0	18	0.001912046
Mm.282053	0	0	14	0.001487147
Mm.290772	0	0	1	0.000106225
Mm.289868	0	0	9	0.000956023
Mm.300271	0	0	1	0.000106225
Mm.353923	0	0	6	0.000637349
Mm.43778	0	0	7	0.000743573
Mm.643	0	0	7	0.000743573
Mm.702	0	0	1	0.000106225
Mm.371577	0	0	2	0.00021245
Mm.371578	0	0	2	0.00021245
Mm.300281	0	0	1	0.000106225
Mm.35583	0	0	1	0.000106225
Mm.16775	3	0.001506024	11	0.001168472

Mm.154915	0	0	2	0.00021245
Mm.331113	0	0	11	0.001168472
Mm.6957	0	0	6	0.000637349
Mm.66	0	0	21	0.00223072
Mm.5291	2	0.001004016	17	0.001805821
Mm.325584	0	0	3	0.000318674
Mm.371579	0	0	5	0.000531124
Mm.260904	0	0	4	0.000424899
Mm.299312	0	0	1	0.000106225
Mm.262067	0	0	1	0.000106225
Mm.328378	0	0	1	0.000106225
Mm.180458	2	0.001004016	14	0.001487147
Mm.297372	0	0	4	0.000424899
Mm.275195	0	0	2	0.00021245
Mm.312227	0	0	1	0.000106225
Mm.288212	1	0.000502008	2	0.00021245
Mm.22723	1	0.000502008	2	0.00021245
Mm.30066	1	0.000502008	14	0.001487147
Mm.319719	0	0	2	0.00021245
Mm.236868	0	0	1	0.000106225
Mm.330075	1	0.000502008	11	0.001168472
Mm.196538	0	0	6	0.000637349
Mm.316362	0	0	1	0.000106225
Mm.290899	0	0	22	0.002336945
Mm.261679	0	0	6	0.000637349
Mm.29183	1	0.000502008	0	0
Mm.276337	0	0	5	0.000531124
Mm.11376	0	0	2	0.00021245
Mm.277891	0	0	1	0.000106225
Mm.3158	0	0	5	0.000531124
Mm.133851	0	0	1	0.000106225
Mm.19355	0	0	1	0.000106225
Mm.27796	0	0	1	0.000106225
Mm.313236	0	0	1	0.000106225
Mm.155033	0	0	1	0.000106225
Mm.218533	0	0	4	0.000424899
Mm.249809	0	0	2	0.00021245
Mm.195628	0	0	1	0.000106225
Mm.28291	2	0.001004016	1	0.000106225
Mm.295618	0	0	4	0.000424899
Mm.2050	0	0	5	0.000531124
Mm.350080	0	0	9	0.000956023
Mm.289669	0	0	1	0.000106225
Mm.345333	0	0	1	0.000106225
Mm.371622	1	0.000502008	1	0.000106225
Mm.289810	0	0	12	0.001274697
Mm.341719	1	0.000502008	7	0.000743573
Mm.21938	0	0	4	0.000424899
Mm.238817	0	0	2	0.00021245
Mm.280083	1	0.000502008	15	0.001593372
Mm.290786	0	0	11	0.001168472
Mm.339491	0	0	8	0.000849798
Mm.21724	0	0	2	0.00021245
Mm.107869	0	0	3	0.000318674
Mm.140568	0	0	5	0.000531124
Mm.236795	0	0	1	0.000106225

Mm.287443	0	0	1	0.000106225
Mm.27871	0	0	2	0.00021245
Mm.288960	0	0	1	0.000106225
Mm.180873	0	0	6	0.000637349
Mm.371658	0	0	11	0.001168472
Mm.13944	0	0	19	0.002018271
Mm.199698	1	0.000502008	2	0.00021245
Mm.218515	1	0.000502008	0	0
Mm.34951	0	0	1	0.000106225
Mm.193040	0	0	1	0.000106225
Mm.168680	0	0	4	0.000424899
Mm.12144	1	0.000502008	0	0
Mm.23825	0	0	2	0.00021245
Mm.260737	0	0	5	0.000531124
Mm.262707	0	0	1	0.000106225
Mm.33360	0	0	1	0.000106225
Mm.355327	3	0.001506024	3	0.000318674
Mm.297	0	0	2	0.00021245
Mm.300639	3	0.001506024	13	0.001380922
Mm.213025	0	0	6	0.000637349
Mm.289106	2	0.001004016	1	0.000106225
Mm.757	2	0.001004016	2	0.00021245
Mm.2945	0	0	1	0.000106225
Mm.18962	0	0	1	0.000106225
Mm.291928	1	0.000502008	0	0
Mm.35738	0	0	2	0.00021245
Mm.205601	0	0	2	0.00021245
Mm.7524	0	0	3	0.000318674
Mm.275608	0	0	1	0.000106225
Mm.336625	0	0	2	0.00021245
Mm.293683	0	0	1	0.000106225
Mm.254494	0	0	1	0.000106225
Mm.289657	2	0.001004016	1	0.000106225
Mm.275839	2	0.001004016	0	0
Mm.12508	0	0	7	0.000743573
Mm.312059	0	0	6	0.000637349
Mm.271967	0	0	1	0.000106225
Mm.247775	0	0	2	0.00021245
Mm.138876	1	0.000502008	0	0
Mm.337074	0	0	5	0.000531124
Mm.14526	0	0	1	0.000106225
Mm.60590	0	0	2	0.00021245
Mm.260098	1	0.000502008	4	0.000424899
Mm.1403	0	0	1	0.000106225
Mm.33779	0	0	1	0.000106225
Mm.297109	0	0	2	0.00021245
Mm.234912	0	0	2	0.00021245
Mm.295533	0	0	1	0.000106225
Mm.3532	0	0	3	0.000318674
Mm.142729	0	0	7	0.000743573
Mm.245746	2	0.001004016	3	0.000318674
Mm.6710	4	0.002008032	1	0.000106225
Mm.18709	0	0	1	0.000106225
Mm.204969	0	0	1	0.000106225
Mm.210018	0	0	2	0.00021245
Mm.121878	0	0	1	0.000106225

Mm.277812	1	0.000502008	1	0.000106225
Mm.136791	2	0.001004016	0	0
Mm.217161	0	0	1	0.000106225
Mm.253156	0	0	1	0.000106225
Mm.21109	0	0	3	0.000318674
Mm.93636	0	0	1	0.000106225
Mm.4420	2	0.001004016	0	0
Mm.338890	1	0.000502008	0	0
Mm.341742	0	0	1	0.000106225
Mm.288195	0	0	1	0.000106225
Mm.44106	0	0	1	0.000106225
Mm.46014	0	0	2	0.00021245
Mm.28095	0	0	2	0.00021245
Mm.295124	0	0	1	0.000106225
Mm.151948	1	0.000502008	0	0
Mm.38445	0	0	1	0.000106225
Mm.3118	0	0	1	0.000106225
Mm.5567	1	0.000502008	3	0.000318674
Mm.28217	0	0	2	0.00021245
Mm.218891	1	0.000502008	0	0
Mm.178947	1	0.000502008	0	0
Mm.215110	0	0	1	0.000106225
Mm.143763	0	0	1	0.000106225
Mm.240839	0	0	4	0.000424899
Mm.33207	0	0	1	0.000106225
Mm.27947	0	0	3	0.000318674
Mm.4113	1	0.000502008	0	0
Mm.259045	0	0	2	0.00021245
Mm.283045	1	0.000502008	4	0.000424899
Mm.335292	0	0	2	0.00021245
Mm.202092	0	0	2	0.00021245
Mm.306770	0	0	6	0.000637349
Mm.183102	0	0	5	0.000531124
Mm.234823	1	0.000502008	0	0
Mm.350347	1	0.000502008	1	0.000106225
Mm.13705	0	0	2	0.00021245
Mm.273768	0	0	4	0.000424899
Mm.371666	0	0	1	0.000106225
Mm.329515	1	0.000502008	0	0
Mm.374780	0	0	1	0.000106225
Mm.87759	0	0	1	0.000106225
Mm.105208	0	0	1	0.000106225
Mm.223504	0	0	1	0.000106225
Mm.6510	1	0.000502008	8	0.000849798
Mm.224189	0	0	1	0.000106225
Mm.264215	1	0.000502008	0	0
Mm.371610	0	0	1	0.000106225
Mm.41077	1	0.000502008	1	0.000106225
Mm.288974	0	0	1	0.000106225
Mm.282751	1	0.000502008	2	0.00021245
Mm.238343	0	0	1	0.000106225
Mm.8681	1	0.000502008	2	0.00021245
Mm.210815	2	0.001004016	0	0
Mm.248464	1	0.000502008	0	0
Mm.1948	1	0.000502008	0	0
Mm.181430	0	0	1	0.000106225

Mm.249479	0	0	1	0.000106225				
Mm.128627	0	0	1	0.000106225				
Mm.150373	0	0	4	0.000424899				
Mm.153911	0	0	1	0.000106225				
Mm.30010	0	0	2	0.00021245				
Mm.259380	0	0	1	0.000106225				
Mm.262039	0	0	3	0.000318674				
Mm.117238	1	0.000502008	1	0.000106225				
Mm.17917	0	0	1	0.000106225				
Mm.288689	0	0	5	0.000531124				
Mm.26834	0	0	1	0.000106225				
Mm.28623	0	0	3	0.000318674				
Mm.271770	0	0	1	0.000106225				
Mm.318430	0	0	1	0.000106225				
Mm.143877	4	0.002008032	1	0.000106225				
Mm.329287	1	0.000502008	0	0				
Mm.324696	1	0.000502008	4	0.000424899				
Mm.287784	0	0	3	0.000318674				
Mm.270295	0	0	1	0.000106225				
Mm.273538	0	0	5	0.000531124				
Mm.227260	0	0	10	0.001062248				
Mm.23895	0	0	1	0.000106225				
Mm.266767	0	0	4	0.000424899				
Mm.240189	0	0	1	0.000106225				
Mm.222272	1	0.000502008	1	0.000106225				
Mm.355686	0	0	1	0.000106225				
Mm.88694	0	0	1	0.000106225				
Mm.223744	0	0	1	0.000106225				
Mm.42170	0	0	1	0.000106225				
Mm.217318	1	0.000502008	3	0.000318674				
Mm.20928	0	0	1	0.000106225				
Mm.258846	1	0.000502008	0	0				
Mm.259374	4	0.002008032	1	0.000106225				
Mm.154121	0	0	1	0.000106225				
Mm.23114	0	0	1	0.000106225				
Mm.276042	0	0	8	0.000849798				
Mm.271661	2	0.001004016	7	0.000743573				
Mm.271674	0	0	3	0.000318674				
Mm.22479	0	0	9	0.000956023				
Mm.273177	0	0	1	0.000106225				
Mm.358618	0	0	6	0.000637349				
Mm.243014	0	0	2	0.00021245				
Mm.1956	0	0	2	0.00021245				
Mm.268000	0	0	1	0.000106225				
>GO:0043232	141	0.098463687	1161	0.160359116	1.628611732	2.67E-09	5.81E-07	intracellular non-membrane· C
Mm.288460	0	0	2	0.00021245				
Mm.150231	0	0	1	0.000106225				
Mm.290563	0	0	3	0.000318674				
Mm.24350	0	0	1	0.000106225				
Mm.352429	1	0.000502008	0	0				
Mm.371563	5	0.00251004	11	0.001168472				
Mm.313345	0	0	1	0.000106225				
Mm.10027	0	0	1	0.000106225				
Mm.274770	0	0	1	0.000106225				
Mm.159684	1	0.000502008	2	0.00021245				
Mm.245739	0	0	1	0.000106225				

Mm.26412	5	0.00251004	2	0.00021245
Mm.133101	1	0.000502008	1	0.000106225
Mm.283802	0	0	1	0.000106225
Mm.280717	1	0.000502008	0	0
Mm.117541	0	0	5	0.000531124
Mm.281005	0	0	1	0.000106225
Mm.206841	1	0.000502008	3	0.000318674
Mm.288015	2	0.001004016	0	0
Mm.89568	0	0	3	0.000318674
Mm.182628	0	0	3	0.000318674
Mm.279751	0	0	3	0.000318674
Mm.10141	2	0.001004016	4	0.000424899
Mm.328945	0	0	5	0.000531124
Mm.254784	0	0	1	0.000106225
Mm.333357	0	0	2	0.00021245
Mm.4071	0	0	11	0.001168472
Mm.29055	0	0	11	0.001168472
Mm.262059	0	0	1	0.000106225
Mm.5001	0	0	1	0.000106225
Mm.311655	1	0.000502008	1	0.000106225
Mm.9244	0	0	1	0.000106225
Mm.32019	0	0	3	0.000318674
Mm.305561	0	0	3	0.000318674
Mm.13433	0	0	2	0.00021245
Mm.128273	2	0.001004016	0	0
Mm.259893	0	0	3	0.000318674
Mm.278578	0	0	1	0.000106225
Mm.247844	0	0	2	0.00021245
Mm.29755	0	0	2	0.00021245
Mm.272226	1	0.000502008	0	0
Mm.16711	0	0	6	0.000637349
Mm.3411	0	0	3	0.000318674
Mm.29709	3	0.001506024	0	0
Mm.129746	1	0.000502008	0	0
Mm.8552	0	0	4	0.000424899
Mm.161470	1	0.000502008	1	0.000106225
Mm.29133	2	0.001004016	3	0.000318674
Mm.15755	0	0	2	0.00021245
Mm.273502	0	0	1	0.000106225
Mm.215389	0	0	2	0.00021245
Mm.24250	0	0	1	0.000106225
Mm.151315	0	0	1	0.000106225
Mm.195770	0	0	1	0.000106225
Mm.132238	0	0	2	0.00021245
Mm.333388	0	0	1	0.000106225
Mm.370289	0	0	1	0.000106225
Mm.8137	0	0	2	0.00021245
Mm.2756	0	0	1	0.000106225
Mm.319660	0	0	1	0.000106225
Mm.22522	0	0	2	0.00021245
Mm.7142	0	0	2	0.00021245
Mm.24248	0	0	1	0.000106225
Mm.15701	0	0	7	0.000743573
Mm.246237	0	0	1	0.000106225
Mm.298443	2	0.001004016	0	0
Mm.29680	1	0.000502008	1	0.000106225

Mm.152466	0	0	1	0.000106225
Mm.21899	0	0	5	0.000531124
Mm.182776	0	0	1	0.000106225
Mm.260893	0	0	2	0.00021245
Mm.128580	0	0	1	0.000106225
Mm.19806	0	0	3	0.000318674
Mm.348392	0	0	2	0.00021245
Mm.160061	0	0	2	0.00021245
Mm.4237	0	0	2	0.00021245
Mm.28659	0	0	1	0.000106225
Mm.180734	1	0.000502008	4	0.000424899
Mm.257590	0	0	1	0.000106225
Mm.7141	0	0	2	0.00021245
Mm.2903	0	0	1	0.000106225
Mm.27705	1	0.000502008	0	0
Mm.16549	0	0	2	0.00021245
Mm.370283	0	0	3	0.000318674
Mm.148877	0	0	1	0.000106225
Mm.332739	0	0	1	0.000106225
Mm.12553	1	0.000502008	0	0
Mm.12864	0	0	1	0.000106225
Mm.290015	0	0	1	0.000106225
Mm.4283	0	0	1	0.000106225
Mm.282719	0	0	3	0.000318674
Mm.22670	1	0.000502008	0	0
Mm.154378	0	0	6	0.000637349
Mm.338720	0	0	2	0.00021245
Mm.6343	3	0.001506024	17	0.001805821
Mm.28764	5	0.00251004	3	0.000318674
Mm.2845	0	0	3	0.000318674
Mm.2215	0	0	2	0.00021245
Mm.297196	1	0.000502008	0	0
Mm.250030	0	0	11	0.001168472
Mm.188413	0	0	2	0.00021245
Mm.88512	0	0	2	0.00021245
Mm.29810	0	0	2	0.00021245
Mm.220367	0	0	1	0.000106225
Mm.25264	0	0	1	0.000106225
Mm.257482	0	0	3	0.000318674
Mm.28630	0	0	2	0.00021245
Mm.140380	0	0	5	0.000531124
Mm.227258	0	0	4	0.000424899
Mm.280311	0	0	1	0.000106225
Mm.271715	0	0	1	0.000106225
Mm.29906	0	0	2	0.00021245
Mm.78861	0	0	4	0.000424899
Mm.33437	0	0	2	0.00021245
Mm.210845	0	0	7	0.000743573
Mm.284592	0	0	2	0.00021245
Mm.358640	0	0	1	0.000106225
Mm.244820	0	0	4	0.000424899
Mm.100113	0	0	16	0.001699596
Mm.371669	0	0	2	0.00021245
Mm.88216	0	0	2	0.00021245
Mm.274995	0	0	3	0.000318674
Mm.306162	0	0	1	0.000106225

Mm.246693	0	0	3	0.000318674
Mm.275158	0	0	1	0.000106225
Mm.3458	0	0	1	0.000106225
Mm.317557	0	0	2	0.00021245
Mm.288730	0	0	1	0.000106225
Mm.279861	0	0	2	0.00021245
Mm.38344	0	0	2	0.00021245
Mm.56337	2	0.001004016	1	0.000106225
Mm.251794	0	0	5	0.000531124
Mm.359653	1	0.000502008	3	0.000318674
Mm.89845	0	0	1	0.000106225
Mm.212525	1	0.000502008	0	0
Mm.24643	0	0	1	0.000106225
Mm.24105	0	0	1	0.000106225
Mm.31979	0	0	1	0.000106225
Mm.12481	0	0	1	0.000106225
Mm.358671	0	0	1	0.000106225
Mm.2444	0	0	1	0.000106225
Mm.273862	0	0	1	0.000106225
Mm.301827	0	0	1	0.000106225
Mm.188432	6	0.003012048	12	0.001274697
Mm.227274	0	0	2	0.00021245
Mm.311337	0	0	3	0.000318674
Mm.361980	0	0	14	0.001487147
Mm.298467	0	0	1	0.000106225
Mm.371545	0	0	7	0.000743573
Mm.5286	0	0	23	0.00244317
Mm.217354	0	0	1	0.000106225
Mm.329631	0	0	1	0.000106225
Mm.328846	0	0	1	0.000106225
Mm.336955	0	0	1	0.000106225
Mm.347060	1	0.000502008	4	0.000424899
Mm.349277	0	0	8	0.000849798
Mm.372072	0	0	3	0.000318674
Mm.309019	0	0	1	0.000106225
Mm.29046	0	0	2	0.00021245
Mm.316592	0	0	4	0.000424899
Mm.307846	0	0	4	0.000424899
Mm.306548	0	0	1	0.000106225
Mm.300263	0	0	3	0.000318674
Mm.325521	0	0	5	0.000531124
Mm.247113	0	0	3	0.000318674
Mm.358632	0	0	1	0.000106225
Mm.104368	0	0	6	0.000637349
Mm.21529	1	0.000502008	1	0.000106225
Mm.371576	0	0	1	0.000106225
Mm.323357	0	0	4	0.000424899
Mm.354330	0	0	10	0.001062248
Mm.4419	0	0	1	0.000106225
Mm.262021	0	0	18	0.001912046
Mm.282053	0	0	14	0.001487147
Mm.290772	0	0	1	0.000106225
Mm.289868	0	0	9	0.000956023
Mm.300271	0	0	1	0.000106225
Mm.353923	0	0	6	0.000637349
Mm.43778	0	0	7	0.000743573

Mm.643	0	0	7	0.000743573
Mm.702	0	0	1	0.000106225
Mm.371577	0	0	2	0.00021245
Mm.371578	0	0	2	0.00021245
Mm.300281	0	0	1	0.000106225
Mm.35583	0	0	1	0.000106225
Mm.16775	3	0.001506024	11	0.001168472
Mm.154915	0	0	2	0.00021245
Mm.331113	0	0	11	0.001168472
Mm.6957	0	0	6	0.000637349
Mm.66	0	0	21	0.00223072
Mm.5291	2	0.001004016	17	0.001805821
Mm.325584	0	0	3	0.000318674
Mm.371579	0	0	5	0.000531124
Mm.260904	0	0	4	0.000424899
Mm.299312	0	0	1	0.000106225
Mm.262067	0	0	1	0.000106225
Mm.328378	0	0	1	0.000106225
Mm.180458	2	0.001004016	14	0.001487147
Mm.297372	0	0	4	0.000424899
Mm.275195	0	0	2	0.00021245
Mm.312227	0	0	1	0.000106225
Mm.288212	1	0.000502008	2	0.00021245
Mm.22723	1	0.000502008	2	0.00021245
Mm.30066	1	0.000502008	14	0.001487147
Mm.319719	0	0	2	0.00021245
Mm.236868	0	0	1	0.000106225
Mm.330075	1	0.000502008	11	0.001168472
Mm.196538	0	0	6	0.000637349
Mm.316362	0	0	1	0.000106225
Mm.290899	0	0	22	0.002336945
Mm.261679	0	0	6	0.000637349
Mm.29183	1	0.000502008	0	0
Mm.276337	0	0	5	0.000531124
Mm.11376	0	0	2	0.00021245
Mm.277891	0	0	1	0.000106225
Mm.3158	0	0	5	0.000531124
Mm.133851	0	0	1	0.000106225
Mm.19355	0	0	1	0.000106225
Mm.27796	0	0	1	0.000106225
Mm.313236	0	0	1	0.000106225
Mm.155033	0	0	1	0.000106225
Mm.218533	0	0	4	0.000424899
Mm.249809	0	0	2	0.00021245
Mm.195628	0	0	1	0.000106225
Mm.28291	2	0.001004016	1	0.000106225
Mm.295618	0	0	4	0.000424899
Mm.2050	0	0	5	0.000531124
Mm.350080	0	0	9	0.000956023
Mm.289669	0	0	1	0.000106225
Mm.345333	0	0	1	0.000106225
Mm.371622	1	0.000502008	1	0.000106225
Mm.289810	0	0	12	0.001274697
Mm.341719	1	0.000502008	7	0.000743573
Mm.21938	0	0	4	0.000424899
Mm.238817	0	0	2	0.00021245

Mm.280083	1	0.000502008	15	0.001593372
Mm.290786	0	0	11	0.001168472
Mm.339491	0	0	8	0.000849798
Mm.21724	0	0	2	0.00021245
Mm.107869	0	0	3	0.000318674
Mm.140568	0	0	5	0.000531124
Mm.236795	0	0	1	0.000106225
Mm.287443	0	0	1	0.000106225
Mm.27871	0	0	2	0.00021245
Mm.288960	0	0	1	0.000106225
Mm.180873	0	0	6	0.000637349
Mm.371658	0	0	11	0.001168472
Mm.13944	0	0	19	0.002018271
Mm.199698	1	0.000502008	2	0.00021245
Mm.218515	1	0.000502008	0	0
Mm.34951	0	0	1	0.000106225
Mm.193040	0	0	1	0.000106225
Mm.168680	0	0	4	0.000424899
Mm.12144	1	0.000502008	0	0
Mm.23825	0	0	2	0.00021245
Mm.260737	0	0	5	0.000531124
Mm.262707	0	0	1	0.000106225
Mm.33360	0	0	1	0.000106225
Mm.355327	3	0.001506024	3	0.000318674
Mm.297	0	0	2	0.00021245
Mm.300639	3	0.001506024	13	0.001380922
Mm.213025	0	0	6	0.000637349
Mm.289106	2	0.001004016	1	0.000106225
Mm.757	2	0.001004016	2	0.00021245
Mm.2945	0	0	1	0.000106225
Mm.18962	0	0	1	0.000106225
Mm.291928	1	0.000502008	0	0
Mm.35738	0	0	2	0.00021245
Mm.205601	0	0	2	0.00021245
Mm.7524	0	0	3	0.000318674
Mm.275608	0	0	1	0.000106225
Mm.336625	0	0	2	0.00021245
Mm.293683	0	0	1	0.000106225
Mm.254494	0	0	1	0.000106225
Mm.289657	2	0.001004016	1	0.000106225
Mm.275839	2	0.001004016	0	0
Mm.12508	0	0	7	0.000743573
Mm.312059	0	0	6	0.000637349
Mm.271967	0	0	1	0.000106225
Mm.247775	0	0	2	0.00021245
Mm.138876	1	0.000502008	0	0
Mm.337074	0	0	5	0.000531124
Mm.14526	0	0	1	0.000106225
Mm.60590	0	0	2	0.00021245
Mm.260098	1	0.000502008	4	0.000424899
Mm.1403	0	0	1	0.000106225
Mm.33779	0	0	1	0.000106225
Mm.297109	0	0	2	0.00021245
Mm.234912	0	0	2	0.00021245
Mm.295533	0	0	1	0.000106225
Mm.3532	0	0	3	0.000318674

Mm.142729	0	0	7	0.000743573
Mm.245746	2	0.001004016	3	0.000318674
Mm.6710	4	0.002008032	1	0.000106225
Mm.18709	0	0	1	0.000106225
Mm.204969	0	0	1	0.000106225
Mm.210018	0	0	2	0.00021245
Mm.121878	0	0	1	0.000106225
Mm.277812	1	0.000502008	1	0.000106225
Mm.136791	2	0.001004016	0	0
Mm.217161	0	0	1	0.000106225
Mm.253156	0	0	1	0.000106225
Mm.21109	0	0	3	0.000318674
Mm.93636	0	0	1	0.000106225
Mm.4420	2	0.001004016	0	0
Mm.338890	1	0.000502008	0	0
Mm.341742	0	0	1	0.000106225
Mm.288195	0	0	1	0.000106225
Mm.44106	0	0	1	0.000106225
Mm.46014	0	0	2	0.00021245
Mm.28095	0	0	2	0.00021245
Mm.295124	0	0	1	0.000106225
Mm.151948	1	0.000502008	0	0
Mm.38445	0	0	1	0.000106225
Mm.3118	0	0	1	0.000106225
Mm.5567	1	0.000502008	3	0.000318674
Mm.28217	0	0	2	0.00021245
Mm.218891	1	0.000502008	0	0
Mm.178947	1	0.000502008	0	0
Mm.215110	0	0	1	0.000106225
Mm.143763	0	0	1	0.000106225
Mm.240839	0	0	4	0.000424899
Mm.33207	0	0	1	0.000106225
Mm.27947	0	0	3	0.000318674
Mm.4113	1	0.000502008	0	0
Mm.259045	0	0	2	0.00021245
Mm.283045	1	0.000502008	4	0.000424899
Mm.335292	0	0	2	0.00021245
Mm.202092	0	0	2	0.00021245
Mm.306770	0	0	6	0.000637349
Mm.183102	0	0	5	0.000531124
Mm.234823	1	0.000502008	0	0
Mm.350347	1	0.000502008	1	0.000106225
Mm.13705	0	0	2	0.00021245
Mm.273768	0	0	4	0.000424899
Mm.371666	0	0	1	0.000106225
Mm.329515	1	0.000502008	0	0
Mm.374780	0	0	1	0.000106225
Mm.87759	0	0	1	0.000106225
Mm.105208	0	0	1	0.000106225
Mm.223504	0	0	1	0.000106225
Mm.6510	1	0.000502008	8	0.000849798
Mm.224189	0	0	1	0.000106225
Mm.264215	1	0.000502008	0	0
Mm.371610	0	0	1	0.000106225
Mm.41077	1	0.000502008	1	0.000106225
Mm.288974	0	0	1	0.000106225

Mm.282751	1	0.000502008	2	0.00021245				
Mm.238343	0	0	1	0.000106225				
Mm.8681	1	0.000502008	2	0.00021245				
Mm.210815	2	0.001004016	0	0				
Mm.248464	1	0.000502008	0	0				
Mm.1948	1	0.000502008	0	0				
Mm.181430	0	0	1	0.000106225				
Mm.249479	0	0	1	0.000106225				
Mm.128627	0	0	1	0.000106225				
Mm.150373	0	0	4	0.000424899				
Mm.153911	0	0	1	0.000106225				
Mm.30010	0	0	2	0.00021245				
Mm.259380	0	0	1	0.000106225				
Mm.262039	0	0	3	0.000318674				
Mm.117238	1	0.000502008	1	0.000106225				
Mm.17917	0	0	1	0.000106225				
Mm.288689	0	0	5	0.000531124				
Mm.26834	0	0	1	0.000106225				
Mm.28623	0	0	3	0.000318674				
Mm.271770	0	0	1	0.000106225				
Mm.318430	0	0	1	0.000106225				
Mm.143877	4	0.002008032	1	0.000106225				
Mm.329287	1	0.000502008	0	0				
Mm.324696	1	0.000502008	4	0.000424899				
Mm.287784	0	0	3	0.000318674				
Mm.270295	0	0	1	0.000106225				
Mm.273538	0	0	5	0.000531124				
Mm.227260	0	0	10	0.001062248				
Mm.23895	0	0	1	0.000106225				
Mm.266767	0	0	4	0.000424899				
Mm.240189	0	0	1	0.000106225				
Mm.222272	1	0.000502008	1	0.000106225				
Mm.355686	0	0	1	0.000106225				
Mm.88694	0	0	1	0.000106225				
Mm.223744	0	0	1	0.000106225				
Mm.42170	0	0	1	0.000106225				
Mm.217318	1	0.000502008	3	0.000318674				
Mm.20928	0	0	1	0.000106225				
Mm.258846	1	0.000502008	0	0				
Mm.259374	4	0.002008032	1	0.000106225				
Mm.154121	0	0	1	0.000106225				
Mm.23114	0	0	1	0.000106225				
Mm.276042	0	0	8	0.000849798				
Mm.271661	2	0.001004016	7	0.000743573				
Mm.271674	0	0	3	0.000318674				
Mm.22479	0	0	9	0.000956023				
Mm.273177	0	0	1	0.000106225				
Mm.358618	0	0	6	0.000637349				
Mm.243014	0	0	2	0.00021245				
Mm.1956	0	0	2	0.00021245				
Mm.268000	0	0	1	0.000106225				
>GO:0005830	9	0.006284916	242	0.033425414	5.318354819	3.56E-08	7.30E-06	cytosolic ribosome (sensu E C
Mm.29046	0	0	2	0.00021245				
Mm.316592	0	0	4	0.000424899				
Mm.307846	0	0	4	0.000424899				
Mm.306548	0	0	1	0.000106225				

Mm.260737	0	0	5	0.000531124					
Mm.300263	0	0	3	0.000318674					
Mm.325521	0	0	5	0.000531124					
Mm.21529	1	0.000502008	1	0.000106225					
Mm.371576	0	0	1	0.000106225					
Mm.323357	0	0	4	0.000424899					
Mm.354330	0	0	10	0.001062248					
Mm.4419	0	0	1	0.000106225					
Mm.262021	0	0	18	0.001912046					
Mm.282053	0	0	14	0.001487147					
Mm.290772	0	0	1	0.000106225					
Mm.289868	0	0	9	0.000956023					
Mm.300271	0	0	1	0.000106225					
Mm.353923	0	0	6	0.000637349					
Mm.43778	0	0	7	0.000743573					
Mm.643	0	0	7	0.000743573					
Mm.702	0	0	1	0.000106225					
Mm.371578	0	0	2	0.00021245					
Mm.300281	0	0	1	0.000106225					
Mm.35583	0	0	1	0.000106225					
Mm.331113	0	0	11	0.001168472					
Mm.6957	0	0	6	0.000637349					
Mm.66	0	0	21	0.00223072					
Mm.5291	2	0.001004016	17	0.001805821					
Mm.325584	0	0	3	0.000318674					
Mm.371579	0	0	5	0.000531124					
Mm.260904	0	0	4	0.000424899					
Mm.250030	0	0	11	0.001168472					
Mm.319719	0	0	2	0.00021245					
Mm.140380	0	0	5	0.000531124					
Mm.262707	0	0	1	0.000106225					
Mm.309019	0	0	1	0.000106225					
Mm.104368	0	0	6	0.000637349					
Mm.297196	1	0.000502008	0	0					
Mm.30066	1	0.000502008	14	0.001487147					
Mm.371622	1	0.000502008	1	0.000106225					
Mm.238817	0	0	2	0.00021245					
Mm.371577	0	0	2	0.00021245					
Mm.16775	3	0.001506024	11	0.001168472					
Mm.154915	0	0	2	0.00021245					
Mm.295618	0	0	4	0.000424899					
Mm.21938	0	0	4	0.000424899					
>GO:0000279	55	0.038407821	120	0.016574586	0.431541939	1.40E-07	2.60E-05	M phase	P
Mm.307103	2	0.001004016	0	0					
Mm.286602	0	0	1	0.000106225					
Mm.181836	8	0.004016064	0	0					
Mm.290015	0	0	1	0.000106225					
Mm.289747	3	0.001506024	1	0.000106225					
Mm.29133	2	0.001004016	3	0.000318674					
Mm.168523	1	0.000502008	0	0					
Mm.4189	0	0	2	0.00021245					
Mm.260114	0	0	3	0.000318674					
Mm.22592	0	0	1	0.000106225					
Mm.2103	0	0	1	0.000106225					
Mm.281367	0	0	2	0.00021245					
Mm.12481	0	0	1	0.000106225					

Mm.143877	4	0.002008032	1	0.000106225				
Mm.29755	0	0	2	0.00021245				
Mm.116649	0	0	1	0.000106225				
Mm.56337	2	0.001004016	1	0.000106225				
Mm.182628	0	0	3	0.000318674				
Mm.42135	0	0	3	0.000318674				
Mm.290422	0	0	1	0.000106225				
Mm.332684	0	0	2	0.00021245				
Mm.315959	1	0.000502008	0	0				
Mm.37825	0	0	2	0.00021245				
Mm.29071	1	0.000502008	1	0.000106225				
Mm.26412	5	0.00251004	2	0.00021245				
Mm.24643	0	0	1	0.000106225				
Mm.318364	0	0	1	0.000106225				
Mm.273502	0	0	1	0.000106225				
Mm.172411	0	0	1	0.000106225				
Mm.6856	11	0.005522088	4	0.000424899				
Mm.272568	0	0	3	0.000318674				
Mm.24250	0	0	1	0.000106225				
Mm.24202	0	0	2	0.00021245				
Mm.257590	0	0	1	0.000106225				
Mm.89830	0	0	3	0.000318674				
Mm.257445	0	0	3	0.000318674				
Mm.206841	1	0.000502008	3	0.000318674				
Mm.291624	0	0	2	0.00021245				
Mm.328945	0	0	5	0.000531124				
Mm.28659	0	0	1	0.000106225				
Mm.271947	0	0	2	0.00021245				
Mm.273538	0	0	5	0.000531124				
Mm.24105	0	0	1	0.000106225				
Mm.193924	0	0	1	0.000106225				
Mm.273570	0	0	1	0.000106225				
Mm.272024	0	0	1	0.000106225				
Mm.89845	0	0	1	0.000106225				
Mm.2852	0	0	2	0.00021245				
Mm.12743	0	0	2	0.00021245				
Mm.272226	1	0.000502008	0	0				
Mm.76911	0	0	1	0.000106225				
Mm.180734	1	0.000502008	4	0.000424899				
Mm.31598	0	0	1	0.000106225				
Mm.245522	5	0.00251004	3	0.000318674				
Mm.288324	0	0	4	0.000424899				
Mm.29760	0	0	5	0.000531124				
Mm.273122	1	0.000502008	0	0				
Mm.259893	0	0	3	0.000318674				
Mm.324553	0	0	2	0.00021245				
Mm.188432	6	0.003012048	12	0.001274697				
Mm.1971	0	0	2	0.00021245				
>GO:0000087	41	0.028631285	79	0.010911602	0.381107667	3.04E-07	4.60E-05	M phase of mitotic cell cycle P
Mm.307103	2	0.001004016	0	0				
Mm.286602	0	0	1	0.000106225				
Mm.181836	8	0.004016064	0	0				
Mm.290015	0	0	1	0.000106225				
Mm.289747	3	0.001506024	1	0.000106225				
Mm.29133	2	0.001004016	3	0.000318674				
Mm.168523	1	0.000502008	0	0				

Mm.4189	0	0	2	0.00021245					
Mm.260114	0	0	3	0.000318674					
Mm.22592	0	0	1	0.000106225					
Mm.2103	0	0	1	0.000106225					
Mm.281367	0	0	2	0.00021245					
Mm.12481	0	0	1	0.000106225					
Mm.143877	4	0.002008032	1	0.000106225					
Mm.29755	0	0	2	0.00021245					
Mm.116649	0	0	1	0.000106225					
Mm.56337	2	0.001004016	1	0.000106225					
Mm.182628	0	0	3	0.000318674					
Mm.42135	0	0	3	0.000318674					
Mm.290422	0	0	1	0.000106225					
Mm.332684	0	0	2	0.00021245					
Mm.315959	1	0.000502008	0	0					
Mm.37825	0	0	2	0.00021245					
Mm.29071	1	0.000502008	1	0.000106225					
Mm.26412	5	0.00251004	2	0.00021245					
Mm.24643	0	0	1	0.000106225					
Mm.318364	0	0	1	0.000106225					
Mm.273502	0	0	1	0.000106225					
Mm.172411	0	0	1	0.000106225					
Mm.6856	11	0.005522088	4	0.000424899					
Mm.272568	0	0	3	0.000318674					
Mm.24250	0	0	1	0.000106225					
Mm.24202	0	0	2	0.00021245					
Mm.257590	0	0	1	0.000106225					
Mm.89830	0	0	3	0.000318674					
Mm.257445	0	0	3	0.000318674					
Mm.206841	1	0.000502008	3	0.000318674					
Mm.291624	0	0	2	0.00021245					
Mm.328945	0	0	5	0.000531124					
Mm.28659	0	0	1	0.000106225					
Mm.271947	0	0	2	0.00021245					
Mm.273538	0	0	5	0.000531124					
Mm.24105	0	0	1	0.000106225					
Mm.193924	0	0	1	0.000106225					
Mm.273570	0	0	1	0.000106225					
Mm.272024	0	0	1	0.000106225					
Mm.89845	0	0	1	0.000106225					
>GO:0051327	30	0.020949721	48	0.006629834	0.316464088	3.56E-07	4.77E-05	M phase of meiotic cell cycl	P
Mm.2852	0	0	2	0.00021245					
Mm.290422	0	0	1	0.000106225					
Mm.12743	0	0	2	0.00021245					
Mm.26412	5	0.00251004	2	0.00021245					
Mm.272226	1	0.000502008	0	0					
Mm.76911	0	0	1	0.000106225					
Mm.180734	1	0.000502008	4	0.000424899					
Mm.31598	0	0	1	0.000106225					
Mm.245522	5	0.00251004	3	0.000318674					
Mm.288324	0	0	4	0.000424899					
Mm.29760	0	0	5	0.000531124					
Mm.273122	1	0.000502008	0	0					
Mm.259893	0	0	3	0.000318674					
Mm.6856	11	0.005522088	4	0.000424899					
Mm.324553	0	0	2	0.00021245					

Mm.188432	6	0.003012048	12	0.001274697					
Mm.1971	0	0	2	0.00021245					
>GO:0051321	30	0.020949721	48	0.006629834	0.316464088	3.56E-07	4.77E-05	meiotic cell cycle	P
Mm.2852	0	0	2	0.00021245					
Mm.290422	0	0	1	0.000106225					
Mm.12743	0	0	2	0.00021245					
Mm.26412	5	0.00251004	2	0.00021245					
Mm.272226	1	0.000502008	0	0					
Mm.76911	0	0	1	0.000106225					
Mm.180734	1	0.000502008	4	0.000424899					
Mm.31598	0	0	1	0.000106225					
Mm.245522	5	0.00251004	3	0.000318674					
Mm.288324	0	0	4	0.000424899					
Mm.29760	0	0	5	0.000531124					
Mm.273122	1	0.000502008	0	0					
Mm.259893	0	0	3	0.000318674					
Mm.6856	11	0.005522088	4	0.000424899					
Mm.324553	0	0	2	0.00021245					
Mm.188432	6	0.003012048	12	0.001274697					
Mm.1971	0	0	2	0.00021245					
>GO:0007126	30	0.020949721	48	0.006629834	0.316464088	3.56E-07	4.77E-05	meiosis	P
Mm.2852	0	0	2	0.00021245					
Mm.290422	0	0	1	0.000106225					
Mm.12743	0	0	2	0.00021245					
Mm.26412	5	0.00251004	2	0.00021245					
Mm.272226	1	0.000502008	0	0					
Mm.76911	0	0	1	0.000106225					
Mm.180734	1	0.000502008	4	0.000424899					
Mm.31598	0	0	1	0.000106225					
Mm.245522	5	0.00251004	3	0.000318674					
Mm.288324	0	0	4	0.000424899					
Mm.29760	0	0	5	0.000531124					
Mm.273122	1	0.000502008	0	0					
Mm.259893	0	0	3	0.000318674					
Mm.6856	11	0.005522088	4	0.000424899					
Mm.324553	0	0	2	0.00021245					
Mm.188432	6	0.003012048	12	0.001274697					
Mm.1971	0	0	2	0.00021245					
>GO:0006414	5	0.00349162	176	0.024309392	6.962209945	8.05E-07	0.000103966	translational elongation	P
Mm.371545	0	0	7	0.000743573					
Mm.5286	0	0	23	0.00244317					
Mm.138471	0	0	1	0.000106225					
Mm.360075	4	0.002008032	85	0.009029106					
Mm.289431	0	0	17	0.001805821					
Mm.280768	0	0	1	0.000106225					
Mm.219675	0	0	1	0.000106225					
Mm.27816	0	0	3	0.000318674					
Mm.2718	0	0	11	0.001168472					
Mm.3158	0	0	5	0.000531124					
Mm.22147	0	0	2	0.00021245					
Mm.29900	0	0	2	0.00021245					
Mm.371625	0	0	6	0.000637349					
Mm.341719	1	0.000502008	7	0.000743573					
Mm.274904	0	0	1	0.000106225					
Mm.303071	0	0	4	0.000424899					
>GO:0000067	11	0.007681564	4	0.000552486	0.071923656	1.72E-06	0.000213634	DNA replication and chromo	P

Mm.6856	11	0.005522088	4	0.000424899					
>GO:0048513	88	0.061452514	251	0.034668508	0.56415118	2.55E-06	0.000307011	organ development	P
Mm.249342	0	0	2	0.00021245					
Mm.254494	0	0	1	0.000106225					
Mm.24615	0	0	4	0.000424899					
Mm.29891	4	0.002008032	2	0.00021245					
Mm.348326	0	0	1	0.000106225					
Mm.238343	0	0	1	0.000106225					
Mm.4352	0	0	1	0.000106225					
Mm.293761	0	0	2	0.00021245					
Mm.241282	0	0	2	0.00021245					
Mm.193925	0	0	4	0.000424899					
Mm.137222	0	0	1	0.000106225					
Mm.344820	0	0	1	0.000106225					
Mm.28052	0	0	2	0.00021245					
Mm.311337	0	0	3	0.000318674					
Mm.192580	0	0	3	0.000318674					
Mm.291928	1	0.000502008	0	0					
Mm.2390	0	0	1	0.000106225					
Mm.262294	1	0.000502008	0	0					
Mm.29279	0	0	2	0.00021245					
Mm.34554	1	0.000502008	0	0					
Mm.1399	0	0	1	0.000106225					
Mm.153415	1	0.000502008	1	0.000106225					
Mm.209813	0	0	1	0.000106225					
Mm.181862	1	0.000502008	0	0					
Mm.268521	0	0	1	0.000106225					
Mm.275742	2	0.001004016	0	0					
Mm.22398	2	0.001004016	0	0					
Mm.223717	0	0	1	0.000106225					
Mm.7320	1	0.000502008	1	0.000106225					
Mm.100399	1	0.000502008	1	0.000106225					
Mm.43358	0	0	4	0.000424899					
Mm.30039	1	0.000502008	0	0					
Mm.25594	0	0	1	0.000106225					
Mm.228798	0	0	1	0.000106225					
Mm.196581	0	0	2	0.00021245					
Mm.8385	0	0	3	0.000318674					
Mm.87611	5	0.00251004	6	0.000637349					
Mm.200770	0	0	1	0.000106225					
Mm.12239	0	0	4	0.000424899					
Mm.214514	0	0	1	0.000106225					
Mm.289584	2	0.001004016	3	0.000318674					
Mm.118034	1	0.000502008	0	0					
Mm.4509	1	0.000502008	1	0.000106225					
Mm.28265	0	0	1	0.000106225					
Mm.273292	1	0.000502008	0	0					
Mm.195898	0	0	2	0.00021245					
Mm.18213	2	0.001004016	3	0.000318674					
Mm.2018	0	0	1	0.000106225					
Mm.288474	3	0.001506024	1	0.000106225					
Mm.10214	0	0	2	0.00021245					
Mm.5356	0	0	2	0.00021245					
Mm.18509	0	0	1	0.000106225					
Mm.16340	0	0	2	0.00021245					
Mm.39089	0	0	2	0.00021245					

Mm.247566	2	0.001004016	4	0.000424899
Mm.249934	0	0	5	0.000531124
Mm.31927	1	0.000502008	1	0.000106225
Mm.287100	0	0	1	0.000106225
Mm.290924	1	0.000502008	0	0
Mm.57223	0	0	1	0.000106225
Mm.259702	0	0	1	0.000106225
Mm.88694	0	0	1	0.000106225
Mm.259318	0	0	1	0.000106225
Mm.330428	2	0.001004016	0	0
Mm.7978	0	0	1	0.000106225
Mm.87759	0	0	1	0.000106225
Mm.267514	1	0.000502008	1	0.000106225
Mm.7996	2	0.001004016	0	0
Mm.246513	0	0	1	0.000106225
Mm.88367	0	0	1	0.000106225
Mm.238213	0	0	1	0.000106225
Mm.4974	0	0	1	0.000106225
Mm.56337	2	0.001004016	1	0.000106225
Mm.287178	0	0	5	0.000531124
Mm.134093	1	0.000502008	0	0
Mm.260374	1	0.000502008	0	0
Mm.288726	0	0	2	0.00021245
Mm.14297	0	0	1	0.000106225
Mm.3057	1	0.000502008	0	0
Mm.24105	0	0	1	0.000106225
Mm.3451	1	0.000502008	0	0
Mm.5098	3	0.001506024	0	0
Mm.245395	0	0	1	0.000106225
Mm.258939	0	0	1	0.000106225
Mm.22699	0	0	2	0.00021245
Mm.24430	0	0	2	0.00021245
Mm.19133	0	0	1	0.000106225
Mm.229532	1	0.000502008	0	0
Mm.298798	0	0	1	0.000106225
Mm.271947	0	0	2	0.00021245
Mm.8681	1	0.000502008	2	0.00021245
Mm.332936	0	0	1	0.000106225
Mm.271898	0	0	5	0.000531124
Mm.9684	0	0	1	0.000106225
Mm.217161	0	0	1	0.000106225
Mm.292510	1	0.000502008	1	0.000106225
Mm.23636	0	0	2	0.00021245
Mm.34871	0	0	5	0.000531124
Mm.4071	0	0	11	0.001168472
Mm.274432	1	0.000502008	0	0
Mm.338720	0	0	2	0.00021245
Mm.3122	0	0	1	0.000106225
Mm.256765	3	0.001506024	0	0
Mm.350712	0	0	1	0.000106225
Mm.213025	0	0	6	0.000637349
Mm.275608	0	0	1	0.000106225
Mm.259021	0	0	3	0.000318674
Mm.337074	0	0	5	0.000531124
Mm.14526	0	0	1	0.000106225
Mm.297109	0	0	2	0.00021245

Mm.271711	0	0	1	0.000106225					
Mm.121878	0	0	1	0.000106225					
Mm.17917	0	0	1	0.000106225					
Mm.295124	0	0	1	0.000106225					
Mm.240839	0	0	4	0.000424899					
Mm.26834	0	0	1	0.000106225					
Mm.28623	0	0	3	0.000318674					
Mm.13705	0	0	2	0.00021245					
Mm.271770	0	0	1	0.000106225					
Mm.757	2	0.001004016	2	0.00021245					
Mm.28095	0	0	2	0.00021245					
Mm.351459	1	0.000502008	2	0.00021245					
Mm.341742	0	0	1	0.000106225					
Mm.129746	1	0.000502008	0	0					
Mm.321828	0	0	4	0.000424899					
Mm.168	1	0.000502008	0	0					
Mm.4913	1	0.000502008	0	0					
Mm.17715	0	0	1	0.000106225					
Mm.272226	1	0.000502008	0	0					
Mm.23122	0	0	1	0.000106225					
Mm.237825	0	0	2	0.00021245					
Mm.925	1	0.000502008	0	0					
Mm.198803	1	0.000502008	4	0.000424899					
Mm.309395	1	0.000502008	0	0					
Mm.348392	0	0	2	0.00021245					
Mm.245522	5	0.00251004	3	0.000318674					
Mm.260288	0	0	2	0.00021245					
Mm.29790	2	0.001004016	0	0					
Mm.18503	1	0.000502008	0	0					
Mm.103668	1	0.000502008	0	0					
Mm.27582	1	0.000502008	0	0					
Mm.288567	0	0	6	0.000637349					
Mm.247073	0	0	2	0.00021245					
Mm.359653	1	0.000502008	3	0.000318674					
Mm.27792	0	0	2	0.00021245					
Mm.133825	2	0.001004016	5	0.000531124					
Mm.124328	0	0	1	0.000106225					
Mm.259333	3	0.001506024	0	0					
Mm.309954	0	0	1	0.000106225					
Mm.21185	0	0	2	0.00021245					
Mm.1894	0	0	1	0.000106225					
Mm.281298	0	0	1	0.000106225					
Mm.16373	0	0	1	0.000106225					
Mm.3825	0	0	1	0.000106225					
Mm.181959	0	0	1	0.000106225					
Mm.275839	2	0.001004016	0	0					
Mm.134191	0	0	1	0.000106225					
Mm.28969	0	0	1	0.000106225					
Mm.249142	1	0.000502008	0	0					
Mm.317947	2	0.001004016	0	0					
Mm.347883	1	0.000502008	0	0					
Mm.22680	0	0	1	0.000106225					
Mm.3996	0	0	3	0.000318674					
>GO:0003917	7	0.004888268	0	0	0	3.31E-06	0.000384168	DNA topoisomerase type I a	F
Mm.217233	7	0.003514056	0	0					
>GO:0006512	122	0.085195531	389	0.053729282	0.630658455	5.14E-06	0.000560238	ubiquitin cycle	P

Mm.6478	1	0.000502008	5	0.000531124
Mm.21295	2	0.001004016	1	0.000106225
Mm.78312	0	0	1	0.000106225
Mm.289747	3	0.001506024	1	0.000106225
Mm.258977	0	0	1	0.000106225
Mm.362087	0	0	1	0.000106225
Mm.360473	0	0	2	0.00021245
Mm.28792	0	0	2	0.00021245
Mm.290908	0	0	1	0.000106225
Mm.119717	1	0.000502008	1	0.000106225
Mm.10702	0	0	2	0.00021245
Mm.266871	1	0.000502008	0	0
Mm.371673	0	0	4	0.000424899
Mm.209265	5	0.00251004	0	0
Mm.218478	1	0.000502008	1	0.000106225
Mm.301373	0	0	4	0.000424899
Mm.291326	0	0	1	0.000106225
Mm.22670	1	0.000502008	0	0
Mm.6370	0	0	1	0.000106225
Mm.279923	0	0	3	0.000318674
Mm.296566	0	0	3	0.000318674
Mm.8211	1	0.000502008	0	0
Mm.22030	0	0	3	0.000318674
Mm.28235	0	0	3	0.000318674
Mm.324553	0	0	2	0.00021245
Mm.2847	2	0.001004016	1	0.000106225
Mm.342009	1	0.000502008	0	0
Mm.235683	0	0	5	0.000531124
Mm.258476	0	0	2	0.00021245
Mm.219581	0	0	2	0.00021245
Mm.42944	2	0.001004016	7	0.000743573
Mm.30602	0	0	1	0.000106225
Mm.23551	0	0	1	0.000106225
Mm.1485	0	0	3	0.000318674
Mm.3074	0	0	2	0.00021245
Mm.240044	2	0.001004016	0	0
Mm.1104	2	0.001004016	2	0.00021245
Mm.172835	2	0.001004016	0	0
Mm.9002	2	0.001004016	2	0.00021245
Mm.259278	1	0.000502008	2	0.00021245
Mm.275195	0	0	2	0.00021245
Mm.29807	6	0.003012048	8	0.000849798
Mm.3571	0	0	1	0.000106225
Mm.237594	0	0	1	0.000106225
Mm.3974	0	0	3	0.000318674
Mm.242646	2	0.001004016	1	0.000106225
Mm.130235	1	0.000502008	0	0
Mm.275138	0	0	1	0.000106225
Mm.18210	0	0	6	0.000637349
Mm.250262	1	0.000502008	0	0
Mm.80484	0	0	1	0.000106225
Mm.371692	1	0.000502008	2	0.00021245
Mm.34012	1	0.000502008	0	0
Mm.21912	0	0	1	0.000106225
Mm.221769	0	0	1	0.000106225
Mm.237670	0	0	2	0.00021245

Mm.21630	1	0.000502008	0	0
Mm.38976	0	0	3	0.000318674
Mm.133921	2	0.001004016	1	0.000106225
Mm.178524	0	0	1	0.000106225
Mm.305925	4	0.002008032	1	0.000106225
Mm.290447	0	0	3	0.000318674
Mm.21182	8	0.004016064	3	0.000318674
Mm.295330	0	0	6	0.000637349
Mm.28010	6	0.003012048	7	0.000743573
Mm.328135	2	0.001004016	4	0.000424899
Mm.12665	0	0	2	0.00021245
Mm.371711	0	0	2	0.00021245
Mm.87611	5	0.00251004	6	0.000637349
Mm.282122	0	0	1	0.000106225
Mm.275279	1	0.000502008	1	0.000106225
Mm.86406	0	0	2	0.00021245
Mm.24608	0	0	3	0.000318674
Mm.188028	0	0	1	0.000106225
Mm.335468	4	0.002008032	1	0.000106225
Mm.286149	0	0	6	0.000637349
Mm.251174	0	0	3	0.000318674
Mm.24088	0	0	1	0.000106225
Mm.296059	1	0.000502008	2	0.00021245
Mm.28369	0	0	18	0.001912046
Mm.214746	0	0	1	0.000106225
Mm.311110	0	0	3	0.000318674
Mm.275970	0	0	1	0.000106225
Mm.27560	0	0	2	0.00021245
Mm.101264	1	0.000502008	0	0
Mm.151640	1	0.000502008	0	0
Mm.272568	0	0	3	0.000318674
Mm.319512	0	0	4	0.000424899
Mm.272770	0	0	2	0.00021245
Mm.261004	0	0	1	0.000106225
Mm.24202	0	0	2	0.00021245
Mm.29405	0	0	4	0.000424899
Mm.258530	0	0	3	0.000318674
Mm.180052	1	0.000502008	9	0.000956023
Mm.228903	0	0	6	0.000637349
Mm.143768	0	0	4	0.000424899
Mm.25298	1	0.000502008	0	0
Mm.329277	0	0	3	0.000318674
Mm.27372	0	0	1	0.000106225
Mm.288924	2	0.001004016	2	0.00021245
Mm.256765	3	0.001506024	0	0
Mm.49884	14	0.007028112	6	0.000637349
Mm.27888	0	0	2	0.00021245
Mm.333157	0	0	6	0.000637349
Mm.371619	0	0	3	0.000318674
Mm.122430	0	0	1	0.000106225
Mm.294777	1	0.000502008	10	0.001062248
Mm.340315	0	0	4	0.000424899
Mm.284587	0	0	4	0.000424899
Mm.28357	0	0	9	0.000956023
Mm.9852	2	0.001004016	0	0
Mm.207678	0	0	2	0.00021245

Mm.337238	1	0.000502008	0	0				
Mm.289248	0	0	2	0.00021245				
Mm.30737	1	0.000502008	1	0.000106225				
Mm.18972	0	0	11	0.001168472				
Mm.159453	1	0.000502008	1	0.000106225				
Mm.89830	0	0	3	0.000318674				
Mm.257445	0	0	3	0.000318674				
Mm.44236	1	0.000502008	0	0				
Mm.309193	0	0	2	0.00021245				
Mm.275426	0	0	3	0.000318674				
Mm.279070	0	0	1	0.000106225				
Mm.276229	0	0	2	0.00021245				
Mm.218350	0	0	4	0.000424899				
Mm.291811	0	0	1	0.000106225				
Mm.152941	0	0	3	0.000318674				
Mm.26696	0	0	1	0.000106225				
Mm.13437	2	0.001004016	2	0.00021245				
Mm.16974	0	0	5	0.000531124				
Mm.101141	0	0	3	0.000318674				
Mm.218910	0	0	1	0.000106225				
Mm.4537	0	0	1	0.000106225				
Mm.154306	0	0	1	0.000106225				
Mm.22491	0	0	2	0.00021245				
Mm.11935	2	0.001004016	3	0.000318674				
Mm.284592	0	0	2	0.00021245				
Mm.98668	0	0	1	0.000106225				
Mm.371667	0	0	3	0.000318674				
Mm.291624	0	0	2	0.00021245				
Mm.219684	3	0.001506024	0	0				
Mm.2026	0	0	4	0.000424899				
Mm.259879	1	0.000502008	0	0				
Mm.289584	2	0.001004016	3	0.000318674				
Mm.272203	0	0	2	0.00021245				
Mm.18706	1	0.000502008	2	0.00021245				
Mm.246750	0	0	1	0.000106225				
Mm.314056	0	0	2	0.00021245				
Mm.21281	1	0.000502008	2	0.00021245				
Mm.321227	0	0	1	0.000106225				
Mm.41063	0	0	1	0.000106225				
Mm.15701	0	0	7	0.000743573				
Mm.29407	0	0	2	0.00021245				
Mm.310	0	0	1	0.000106225				
Mm.332268	0	0	1	0.000106225				
Mm.274360	0	0	2	0.00021245				
Mm.172605	0	0	2	0.00021245				
Mm.287783	1	0.000502008	0	0				
Mm.270484	0	0	3	0.000318674				
Mm.166372	0	0	2	0.00021245				
Mm.26194	0	0	3	0.000318674				
Mm.28930	1	0.000502008	1	0.000106225				
Mm.262859	1	0.000502008	0	0				
Mm.275574	1	0.000502008	0	0				
Mm.22225	0	0	1	0.000106225				
Mm.274843	0	0	2	0.00021245				
Mm.326945	0	0	1	0.000106225				
>GO:0008639	32	0.022346369	62	0.008563536	0.383218232	8.09E-06	0.000828555	small protein conjugating er F

Mm.360473	0	0	2	0.00021245					
Mm.290908	0	0	1	0.000106225					
Mm.119717	1	0.000502008	1	0.000106225					
Mm.371673	0	0	4	0.000424899					
Mm.23551	0	0	1	0.000106225					
Mm.1485	0	0	3	0.000318674					
Mm.3074	0	0	2	0.00021245					
Mm.240044	2	0.001004016	0	0					
Mm.172835	2	0.001004016	0	0					
Mm.275195	0	0	2	0.00021245					
Mm.21182	8	0.004016064	3	0.000318674					
Mm.319512	0	0	4	0.000424899					
Mm.180052	1	0.000502008	9	0.000956023					
Mm.256765	3	0.001506024	0	0					
Mm.49884	14	0.007028112	6	0.000637349					
Mm.371619	0	0	3	0.000318674					
Mm.122430	0	0	1	0.000106225					
Mm.340315	0	0	4	0.000424899					
Mm.284587	0	0	4	0.000424899					
Mm.207678	0	0	2	0.00021245					
Mm.337238	1	0.000502008	0	0					
Mm.89830	0	0	3	0.000318674					
Mm.309193	0	0	2	0.00021245					
Mm.22491	0	0	2	0.00021245					
Mm.371667	0	0	3	0.000318674					
>GO:0009887	84	0.058659218	244	0.033701657	0.574533018	8.68E-06	0.000828555	organogenesis	P
Mm.34554	1	0.000502008	0	0					
Mm.1399	0	0	1	0.000106225					
Mm.153415	1	0.000502008	1	0.000106225					
Mm.209813	0	0	1	0.000106225					
Mm.181862	1	0.000502008	0	0					
Mm.268521	0	0	1	0.000106225					
Mm.275742	2	0.001004016	0	0					
Mm.22398	2	0.001004016	0	0					
Mm.223717	0	0	1	0.000106225					
Mm.7320	1	0.000502008	1	0.000106225					
Mm.100399	1	0.000502008	1	0.000106225					
Mm.43358	0	0	4	0.000424899					
Mm.30039	1	0.000502008	0	0					
Mm.25594	0	0	1	0.000106225					
Mm.228798	0	0	1	0.000106225					
Mm.196581	0	0	2	0.00021245					
Mm.8385	0	0	3	0.000318674					
Mm.87611	5	0.00251004	6	0.000637349					
Mm.200770	0	0	1	0.000106225					
Mm.12239	0	0	4	0.000424899					
Mm.214514	0	0	1	0.000106225					
Mm.289584	2	0.001004016	3	0.000318674					
Mm.118034	1	0.000502008	0	0					
Mm.4509	1	0.000502008	1	0.000106225					
Mm.28265	0	0	1	0.000106225					
Mm.273292	1	0.000502008	0	0					
Mm.195898	0	0	2	0.00021245					
Mm.18213	2	0.001004016	3	0.000318674					
Mm.2018	0	0	1	0.000106225					
Mm.288474	3	0.001506024	1	0.000106225					

Mm.10214	0	0	2	0.00021245
Mm.29279	0	0	2	0.00021245
Mm.291928	1	0.000502008	0	0
Mm.5356	0	0	2	0.00021245
Mm.18509	0	0	1	0.000106225
Mm.16340	0	0	2	0.00021245
Mm.39089	0	0	2	0.00021245
Mm.247566	2	0.001004016	4	0.000424899
Mm.249934	0	0	5	0.000531124
Mm.31927	1	0.000502008	1	0.000106225
Mm.287100	0	0	1	0.000106225
Mm.290924	1	0.000502008	0	0
Mm.57223	0	0	1	0.000106225
Mm.259702	0	0	1	0.000106225
Mm.88694	0	0	1	0.000106225
Mm.259318	0	0	1	0.000106225
Mm.330428	2	0.001004016	0	0
Mm.2390	0	0	1	0.000106225
Mm.7978	0	0	1	0.000106225
Mm.87759	0	0	1	0.000106225
Mm.293761	0	0	2	0.00021245
Mm.267514	1	0.000502008	1	0.000106225
Mm.7996	2	0.001004016	0	0
Mm.246513	0	0	1	0.000106225
Mm.88367	0	0	1	0.000106225
Mm.238213	0	0	1	0.000106225
Mm.4974	0	0	1	0.000106225
Mm.56337	2	0.001004016	1	0.000106225
Mm.287178	0	0	5	0.000531124
Mm.134093	1	0.000502008	0	0
Mm.260374	1	0.000502008	0	0
Mm.288726	0	0	2	0.00021245
Mm.192580	0	0	3	0.000318674
Mm.14297	0	0	1	0.000106225
Mm.254494	0	0	1	0.000106225
Mm.3057	1	0.000502008	0	0
Mm.24105	0	0	1	0.000106225
Mm.3451	1	0.000502008	0	0
Mm.5098	3	0.001506024	0	0
Mm.245395	0	0	1	0.000106225
Mm.258939	0	0	1	0.000106225
Mm.22699	0	0	2	0.00021245
Mm.24430	0	0	2	0.00021245
Mm.19133	0	0	1	0.000106225
Mm.229532	1	0.000502008	0	0
Mm.298798	0	0	1	0.000106225
Mm.271947	0	0	2	0.00021245
Mm.8681	1	0.000502008	2	0.00021245
Mm.332936	0	0	1	0.000106225
Mm.271898	0	0	5	0.000531124
Mm.9684	0	0	1	0.000106225
Mm.217161	0	0	1	0.000106225
Mm.292510	1	0.000502008	1	0.000106225
Mm.23636	0	0	2	0.00021245
Mm.262294	1	0.000502008	0	0
Mm.34871	0	0	5	0.000531124

Mm.4071	0	0	11	0.001168472
Mm.274432	1	0.000502008	0	0
Mm.338720	0	0	2	0.00021245
Mm.3122	0	0	1	0.000106225
Mm.256765	3	0.001506024	0	0
Mm.350712	0	0	1	0.000106225
Mm.213025	0	0	6	0.000637349
Mm.275608	0	0	1	0.000106225
Mm.259021	0	0	3	0.000318674
Mm.337074	0	0	5	0.000531124
Mm.14526	0	0	1	0.000106225
Mm.297109	0	0	2	0.00021245
Mm.271711	0	0	1	0.000106225
Mm.121878	0	0	1	0.000106225
Mm.17917	0	0	1	0.000106225
Mm.295124	0	0	1	0.000106225
Mm.240839	0	0	4	0.000424899
Mm.26834	0	0	1	0.000106225
Mm.28623	0	0	3	0.000318674
Mm.13705	0	0	2	0.00021245
Mm.271770	0	0	1	0.000106225
Mm.757	2	0.001004016	2	0.00021245
Mm.28095	0	0	2	0.00021245
Mm.351459	1	0.000502008	2	0.00021245
Mm.341742	0	0	1	0.000106225
Mm.129746	1	0.000502008	0	0
Mm.321828	0	0	4	0.000424899
Mm.168	1	0.000502008	0	0
Mm.4913	1	0.000502008	0	0
Mm.17715	0	0	1	0.000106225
Mm.272226	1	0.000502008	0	0
Mm.23122	0	0	1	0.000106225
Mm.237825	0	0	2	0.00021245
Mm.925	1	0.000502008	0	0
Mm.198803	1	0.000502008	4	0.000424899
Mm.309395	1	0.000502008	0	0
Mm.348392	0	0	2	0.00021245
Mm.245522	5	0.00251004	3	0.000318674
Mm.260288	0	0	2	0.00021245
Mm.29790	2	0.001004016	0	0
Mm.18503	1	0.000502008	0	0
Mm.103668	1	0.000502008	0	0
Mm.241282	0	0	2	0.00021245
Mm.27582	1	0.000502008	0	0
Mm.288567	0	0	6	0.000637349
Mm.247073	0	0	2	0.00021245
Mm.359653	1	0.000502008	3	0.000318674
Mm.27792	0	0	2	0.00021245
Mm.133825	2	0.001004016	5	0.000531124
Mm.124328	0	0	1	0.000106225
Mm.259333	3	0.001506024	0	0
Mm.309954	0	0	1	0.000106225
Mm.21185	0	0	2	0.00021245
Mm.1894	0	0	1	0.000106225
Mm.281298	0	0	1	0.000106225
Mm.16373	0	0	1	0.000106225

Mm.3825	0	0	1	0.000106225						
Mm.181959	0	0	1	0.000106225						
Mm.275839	2	0.001004016	0	0						
Mm.134191	0	0	1	0.000106225						
Mm.28969	0	0	1	0.000106225						
Mm.249142	1	0.000502008	0	0						
Mm.249342	0	0	2	0.00021245						
Mm.317947	2	0.001004016	0	0						
Mm.347883	1	0.000502008	0	0						
Mm.22680	0	0	1	0.000106225						
Mm.3996	0	0	3	0.000318674						
Mm.238343	0	0	1	0.000106225						
Mm.4352	0	0	1	0.000106225						
Mm.193925	0	0	4	0.000424899						
Mm.137222	0	0	1	0.000106225						
Mm.344820	0	0	1	0.000106225						
Mm.28052	0	0	2	0.00021245						
Mm.311337	0	0	3	0.000318674						
>GO:0004840	32	0.022346369	62	0.008563536	0.383218232	8.09E-06	0.000828555	ubiquitin conjugating enzym	F	
Mm.360473	0	0	2	0.00021245						
Mm.290908	0	0	1	0.000106225						
Mm.119717	1	0.000502008	1	0.000106225						
Mm.371673	0	0	4	0.000424899						
Mm.23551	0	0	1	0.000106225						
Mm.1485	0	0	3	0.000318674						
Mm.3074	0	0	2	0.00021245						
Mm.240044	2	0.001004016	0	0						
Mm.172835	2	0.001004016	0	0						
Mm.275195	0	0	2	0.00021245						
Mm.21182	8	0.004016064	3	0.000318674						
Mm.319512	0	0	4	0.000424899						
Mm.180052	1	0.000502008	9	0.000956023						
Mm.256765	3	0.001506024	0	0						
Mm.49884	14	0.007028112	6	0.000637349						
Mm.371619	0	0	3	0.000318674						
Mm.122430	0	0	1	0.000106225						
Mm.340315	0	0	4	0.000424899						
Mm.284587	0	0	4	0.000424899						
Mm.207678	0	0	2	0.00021245						
Mm.337238	1	0.000502008	0	0						
Mm.89830	0	0	3	0.000318674						
Mm.309193	0	0	2	0.00021245						
Mm.22491	0	0	2	0.00021245						
Mm.371667	0	0	3	0.000318674						
>GO:0006259	99	0.069134078	302	0.041712707	0.603359562	8.75E-06	0.000828555	DNA metabolism	P	
Mm.277779	0	0	1	0.000106225						
Mm.36241	0	0	3	0.000318674						
Mm.12932	1	0.000502008	0	0						
Mm.4619	0	0	4	0.000424899						
Mm.18210	0	0	6	0.000637349						
Mm.21469	1	0.000502008	0	0						
Mm.315959	1	0.000502008	0	0						
Mm.37825	0	0	2	0.00021245						
Mm.4237	0	0	2	0.00021245						
Mm.6856	11	0.005522088	4	0.000424899						
Mm.287837	0	0	1	0.000106225						

Mm.272226	1	0.000502008	0	0
Mm.259294	1	0.000502008	0	0
Mm.183110	1	0.000502008	0	0
Mm.370283	0	0	3	0.000318674
Mm.282499	0	0	1	0.000106225
Mm.2952	0	0	3	0.000318674
Mm.288179	0	0	1	0.000106225
Mm.277136	0	0	1	0.000106225
Mm.4502	0	0	2	0.00021245
Mm.16711	0	0	6	0.000637349
Mm.1500	0	0	1	0.000106225
Mm.5048	0	0	1	0.000106225
Mm.4933	0	0	2	0.00021245
Mm.31274	1	0.000502008	5	0.000531124
Mm.317947	2	0.001004016	0	0
Mm.3411	0	0	3	0.000318674
Mm.7141	0	0	2	0.00021245
Mm.16549	0	0	2	0.00021245
Mm.35061	0	0	1	0.000106225
Mm.9199	0	0	5	0.000531124
Mm.2903	0	0	1	0.000106225
Mm.27705	1	0.000502008	0	0
Mm.12145	0	0	4	0.000424899
Mm.148877	0	0	1	0.000106225
Mm.332739	0	0	1	0.000106225
Mm.2870	0	0	3	0.000318674
Mm.197486	0	0	2	0.00021245
Mm.99	0	0	1	0.000106225
Mm.217233	7	0.003514056	0	0
Mm.291059	1	0.000502008	0	0
Mm.21724	0	0	2	0.00021245
Mm.358656	0	0	1	0.000106225
Mm.334856	0	0	1	0.000106225
Mm.276356	0	0	1	0.000106225
Mm.24738	1	0.000502008	2	0.00021245
Mm.335942	0	0	5	0.000531124
Mm.29709	3	0.001506024	0	0
Mm.21873	0	0	3	0.000318674
Mm.37562	1	0.000502008	7	0.000743573
Mm.29073	0	0	1	0.000106225
Mm.180734	1	0.000502008	4	0.000424899
Mm.12553	1	0.000502008	0	0
Mm.214703	0	0	1	0.000106225
Mm.105585	0	0	2	0.00021245
Mm.16110	0	0	1	0.000106225
Mm.12239	0	0	4	0.000424899
Mm.206921	0	0	2	0.00021245
Mm.216227	0	0	1	0.000106225
Mm.312323	0	0	1	0.000106225
Mm.281482	0	0	1	0.000106225
Mm.203	0	0	4	0.000424899
Mm.236256	1	0.000502008	0	0
Mm.289915	2	0.001004016	2	0.00021245
Mm.36524	0	0	2	0.00021245
Mm.282335	0	0	4	0.000424899
Mm.288809	0	0	1	0.000106225

Mm.22700	1	0.000502008	1	0.000106225
Mm.71	0	0	1	0.000106225
Mm.182628	0	0	3	0.000318674
Mm.196846	0	0	1	0.000106225
Mm.185467	0	0	1	0.000106225
Mm.298456	0	0	2	0.00021245
Mm.262117	0	0	1	0.000106225
Mm.172835	2	0.001004016	0	0
Mm.259278	1	0.000502008	2	0.00021245
Mm.1393	0	0	1	0.000106225
Mm.10141	2	0.001004016	4	0.000424899
Mm.4347	0	0	3	0.000318674
Mm.23267	0	0	1	0.000106225
Mm.246010	0	0	3	0.000318674
Mm.26412	5	0.00251004	2	0.00021245
Mm.23122	0	0	1	0.000106225
Mm.272989	1	0.000502008	0	0
Mm.173953	2	0.001004016	2	0.00021245
Mm.23739	0	0	2	0.00021245
Mm.41447	1	0.000502008	0	0
Mm.323072	0	0	1	0.000106225
Mm.2805	1	0.000502008	0	0
Mm.22117	0	0	3	0.000318674
Mm.128580	0	0	1	0.000106225
Mm.5001	0	0	1	0.000106225
Mm.57223	0	0	1	0.000106225
Mm.22522	0	0	2	0.00021245
Mm.7142	0	0	2	0.00021245
Mm.268657	0	0	1	0.000106225
Mm.2444	0	0	1	0.000106225
Mm.240434	0	0	1	0.000106225
Mm.34410	1	0.000502008	0	0
Mm.259893	0	0	3	0.000318674
Mm.272472	0	0	1	0.000106225
Mm.313345	0	0	1	0.000106225
Mm.2756	0	0	1	0.000106225
Mm.319660	0	0	1	0.000106225
Mm.9244	0	0	1	0.000106225
Mm.25339	0	0	1	0.000106225
Mm.311655	1	0.000502008	1	0.000106225
Mm.313303	0	0	2	0.00021245
Mm.22478	4	0.002008032	0	0
Mm.333388	0	0	1	0.000106225
Mm.29055	0	0	11	0.001168472
Mm.262059	0	0	1	0.000106225
Mm.8137	0	0	2	0.00021245
Mm.24248	0	0	1	0.000106225
Mm.128273	2	0.001004016	0	0
Mm.195770	0	0	1	0.000106225
Mm.152466	0	0	1	0.000106225
Mm.246803	2	0.001004016	1	0.000106225
Mm.290563	0	0	3	0.000318674
Mm.24350	0	0	1	0.000106225
Mm.352429	1	0.000502008	0	0
Mm.371563	5	0.00251004	11	0.001168472
Mm.21899	0	0	5	0.000531124

Mm.294625	0	0	5	0.000531124					
Mm.182776	0	0	1	0.000106225					
Mm.133101	1	0.000502008	1	0.000106225					
Mm.283802	0	0	1	0.000106225					
Mm.117541	0	0	5	0.000531124					
Mm.290407	1	0.000502008	3	0.000318674					
Mm.337558	0	0	3	0.000318674					
Mm.29680	1	0.000502008	1	0.000106225					
Mm.273570	0	0	1	0.000106225					
Mm.35345	0	0	1	0.000106225					
Mm.289584	2	0.001004016	3	0.000318674					
Mm.19806	0	0	3	0.000318674					
Mm.344671	1	0.000502008	0	0					
Mm.280768	0	0	1	0.000106225					
Mm.196508	1	0.000502008	1	0.000106225					
Mm.209385	3	0.001506024	2	0.00021245					
Mm.332268	0	0	1	0.000106225					
Mm.126976	0	0	2	0.00021245					
Mm.348392	0	0	2	0.00021245					
Mm.283410	1	0.000502008	8	0.000849798					
Mm.41077	1	0.000502008	1	0.000106225					
Mm.29142	0	0	2	0.00021245					
Mm.270487	1	0.000502008	0	0					
Mm.24176	0	0	1	0.000106225					
Mm.181661	1	0.000502008	0	0					
Mm.182836	1	0.000502008	1	0.000106225					
Mm.252213	0	0	1	0.000106225					
Mm.89568	0	0	3	0.000318674					
Mm.279751	0	0	3	0.000318674					
Mm.622	0	0	1	0.000106225					
Mm.270186	9	0.004518072	8	0.000849798					
Mm.371732	0	0	1	0.000106225					
Mm.291274	0	0	1	0.000106225					
Mm.351459	1	0.000502008	2	0.00021245					
Mm.246688	3	0.001506024	2	0.00021245					
Mm.278578	0	0	1	0.000106225					
Mm.247844	0	0	2	0.00021245					
Mm.248967	0	0	1	0.000106225					
Mm.2817	0	0	1	0.000106225					
Mm.318430	0	0	1	0.000106225					
>GO:0005615	96	0.067039106	767	0.105939227	1.580260129	8.80E-06	0.000828555	extracellular space	C
Mm.287187	0	0	6	0.000637349					
Mm.188544	0	0	1	0.000106225					
Mm.151819	0	0	1	0.000106225					
Mm.335520	1	0.000502008	0	0					
Mm.292803	0	0	1	0.000106225					
Mm.275054	0	0	1	0.000106225					
Mm.244319	0	0	1	0.000106225					
Mm.17807	0	0	2	0.00021245					
Mm.145488	0	0	3	0.000318674					
Mm.246440	0	0	1	0.000106225					
Mm.250392	0	0	8	0.000849798					
Mm.28853	1	0.000502008	7	0.000743573					
Mm.255607	0	0	3	0.000318674					
Mm.260144	0	0	1	0.000106225					
Mm.2445	0	0	5	0.000531124					

Mm.28908	0	0	2	0.00021245
Mm.1408	0	0	2	0.00021245
Mm.1114	0	0	2	0.00021245
Mm.195224	0	0	7	0.000743573
Mm.302724	0	0	1	0.000106225
Mm.35691	0	0	1	0.000106225
Mm.214514	0	0	1	0.000106225
Mm.29821	0	0	3	0.000318674
Mm.19133	0	0	1	0.000106225
Mm.276137	1	0.000502008	9	0.000956023
Mm.163	0	0	1	0.000106225
Mm.290516	0	0	2	0.00021245
Mm.2608	0	0	1	0.000106225
Mm.42160	2	0.001004016	9	0.000956023
Mm.118034	1	0.000502008	0	0
Mm.237825	0	0	2	0.00021245
Mm.13806	0	0	2	0.00021245
Mm.29095	0	0	1	0.000106225
Mm.370172	1	0.000502008	0	0
Mm.1971	0	0	2	0.00021245
Mm.248827	0	0	6	0.000637349
Mm.1641	0	0	1	0.000106225
Mm.4880	0	0	1	0.000106225
Mm.297964	0	0	1	0.000106225
Mm.298775	0	0	3	0.000318674
Mm.35605	1	0.000502008	2	0.00021245
Mm.257437	1	0.000502008	1	0.000106225
Mm.22680	0	0	1	0.000106225
Mm.117180	0	0	1	0.000106225
Mm.298812	0	0	73	0.007754408
Mm.252145	0	0	1	0.000106225
Mm.20837	0	0	1	0.000106225
Mm.200608	1	0.000502008	10	0.001062248
Mm.290924	1	0.000502008	0	0
Mm.272210	0	0	1	0.000106225
Mm.21325	0	0	1	0.000106225
Mm.3819	0	0	1	0.000106225
Mm.4352	0	0	1	0.000106225
Mm.181021	0	0	4	0.000424899
Mm.2509	1	0.000502008	2	0.00021245
Mm.277792	0	0	1	0.000106225
Mm.30041	0	0	2	0.00021245
Mm.180182	0	0	1	0.000106225
Mm.548	0	0	4	0.000424899
Mm.13787	1	0.000502008	0	0
Mm.4263	0	0	1	0.000106225
Mm.236553	0	0	11	0.001168472
Mm.322945	0	0	5	0.000531124
Mm.930	0	0	20	0.002124495
Mm.66222	0	0	1	0.000106225
Mm.316000	1	0.000502008	0	0
Mm.101591	4	0.002008032	3	0.000318674
Mm.167882	0	0	1	0.000106225
Mm.371556	0	0	1	0.000106225
Mm.7236	0	0	3	0.000318674
Mm.18353	0	0	1	0.000106225

Mm.3433	0	0	1	0.000106225
Mm.125580	0	0	1	0.000106225
Mm.229532	1	0.000502008	0	0
Mm.7978	0	0	1	0.000106225
Mm.209813	0	0	1	0.000106225
Mm.271976	0	0	2	0.00021245
Mm.9075	0	0	1	0.000106225
Mm.15295	1	0.000502008	0	0
Mm.1650	0	0	1	0.000106225
Mm.309395	1	0.000502008	0	0
Mm.293761	0	0	2	0.00021245
Mm.271717	0	0	1	0.000106225
Mm.150	0	0	1	0.000106225
Mm.22119	0	0	1	0.000106225
Mm.241282	0	0	2	0.00021245
Mm.7996	2	0.001004016	0	0
Mm.16340	0	0	2	0.00021245
Mm.3894	0	0	1	0.000106225
Mm.287146	0	0	1	0.000106225
Mm.193099	0	0	1	0.000106225
Mm.4913	1	0.000502008	0	0
Mm.243722	0	0	4	0.000424899
Mm.3196	0	0	2	0.00021245
Mm.3982	0	0	1	0.000106225
Mm.12468	1	0.000502008	0	0
Mm.45054	2	0.001004016	1	0.000106225
Mm.9714	4	0.002008032	4	0.000424899
Mm.3711	0	0	4	0.000424899
Mm.88367	0	0	1	0.000106225
Mm.26859	0	0	3	0.000318674
Mm.287807	0	0	1	0.000106225
Mm.230169	0	0	2	0.00021245
Mm.291831	1	0.000502008	0	0
Mm.290834	0	0	1	0.000106225
Mm.1568	0	0	3	0.000318674
Mm.263177	0	0	1	0.000106225
Mm.18556	0	0	1	0.000106225
Mm.292208	0	0	9	0.000956023
Mm.27816	0	0	3	0.000318674
Mm.293529	0	0	2	0.00021245
Mm.313345	0	0	1	0.000106225
Mm.22668	0	0	3	0.000318674
Mm.12559	0	0	1	0.000106225
Mm.275742	2	0.001004016	0	0
Mm.233799	0	0	6	0.000637349
Mm.233802	0	0	1	0.000106225
Mm.4364	0	0	3	0.000318674
Mm.245210	0	0	1	0.000106225
Mm.6424	0	0	2	0.00021245
Mm.2863	0	0	1	0.000106225
Mm.22398	2	0.001004016	0	0
Mm.142722	0	0	1	0.000106225
Mm.16716	0	0	3	0.000318674
Mm.218846	0	0	1	0.000106225
Mm.9537	0	0	5	0.000531124
Mm.43831	0	0	1	0.000106225

Mm.21981	0	0	6	0.000637349
Mm.1514	1	0.000502008	0	0
Mm.271854	0	0	4	0.000424899
Mm.277661	0	0	5	0.000531124
Mm.3122	0	0	1	0.000106225
Mm.282359	0	0	7	0.000743573
Mm.18888	0	0	2	0.00021245
Mm.788	0	0	2	0.00021245
Mm.29590	0	0	1	0.000106225
Mm.4259	0	0	5	0.000531124
Mm.24754	4	0.002008032	6	0.000637349
Mm.249225	0	0	6	0.000637349
Mm.17771	0	0	1	0.000106225
Mm.234654	0	0	18	0.001912046
Mm.263847	0	0	1	0.000106225
Mm.286488	0	0	2	0.00021245
Mm.30045	0	0	2	0.00021245
Mm.291799	0	0	4	0.000424899
Mm.1451	0	0	4	0.000424899
Mm.255116	0	0	1	0.000106225
Mm.373531	0	0	2	0.00021245
Mm.4825	0	0	2	0.00021245
Mm.21535	0	0	1	0.000106225
Mm.269657	0	0	3	0.000318674
Mm.443	1	0.000502008	0	0
Mm.4974	0	0	1	0.000106225
Mm.258759	1	0.000502008	0	0
Mm.29842	0	0	2	0.00021245
Mm.93335	0	0	4	0.000424899
Mm.16660	0	0	6	0.000637349
Mm.5121	0	0	4	0.000424899
Mm.3401	1	0.000502008	0	0
Mm.296814	0	0	1	0.000106225
Mm.34775	1	0.000502008	4	0.000424899
Mm.5092	0	0	1	0.000106225
Mm.154660	0	0	1	0.000106225
Mm.214593	0	0	1	0.000106225
Mm.12616	1	0.000502008	1	0.000106225
Mm.277498	1	0.000502008	9	0.000956023
Mm.316080	0	0	1	0.000106225
Mm.220821	0	0	1	0.000106225
Mm.211494	0	0	1	0.000106225
Mm.330764	0	0	1	0.000106225
Mm.24807	0	0	1	0.000106225
Mm.2339	0	0	1	0.000106225
Mm.945	0	0	1	0.000106225
Mm.29855	0	0	1	0.000106225
Mm.287425	0	0	1	0.000106225
Mm.332303	0	0	2	0.00021245
Mm.332936	0	0	1	0.000106225
Mm.276776	0	0	1	0.000106225
Mm.194462	0	0	3	0.000318674
Mm.3057	1	0.000502008	0	0
Mm.10152	0	0	1	0.000106225
Mm.293517	0	0	3	0.000318674
Mm.15125	0	0	1	0.000106225

Mm.332590	0	0	2	0.00021245
Mm.134093	1	0.000502008	0	0
Mm.260374	1	0.000502008	0	0
Mm.22699	0	0	2	0.00021245
Mm.248334	0	0	1	0.000106225
Mm.4954	0	0	2	0.00021245
Mm.272675	0	0	1	0.000106225
Mm.269857	0	0	1	0.000106225
Mm.291442	0	0	2	0.00021245
Mm.295230	0	0	4	0.000424899
Mm.288474	3	0.001506024	1	0.000106225
Mm.296169	0	0	1	0.000106225
Mm.14814	0	0	1	0.000106225
Mm.229107	0	0	2	0.00021245
Mm.5090	0	0	1	0.000106225
Mm.137222	0	0	1	0.000106225
Mm.18213	2	0.001004016	3	0.000318674
Mm.14455	0	0	1	0.000106225
Mm.200775	0	0	1	0.000106225
Mm.159651	0	0	1	0.000106225
Mm.3509	0	0	1	0.000106225
Mm.790	0	0	2	0.00021245
Mm.87773	0	0	8	0.000849798
Mm.198803	1	0.000502008	4	0.000424899
Mm.24430	0	0	2	0.00021245
Mm.3555	0	0	1	0.000106225
Mm.4141	0	0	1	0.000106225
Mm.22718	1	0.000502008	1	0.000106225
Mm.1249	0	0	1	0.000106225
Mm.262184	0	0	1	0.000106225
Mm.21109	0	0	3	0.000318674
Mm.6510	1	0.000502008	8	0.000849798
Mm.1381	0	0	9	0.000956023
Mm.283682	1	0.000502008	0	0
Mm.7244	0	0	1	0.000106225
Mm.24044	0	0	2	0.00021245
Mm.227549	0	0	1	0.000106225
Mm.234129	2	0.001004016	0	0
Mm.12863	1	0.000502008	1	0.000106225
Mm.334648	0	0	2	0.00021245
Mm.103648	0	0	1	0.000106225
Mm.29183	1	0.000502008	0	0
Mm.95452	0	0	1	0.000106225
Mm.258484	0	0	2	0.00021245
Mm.281356	0	0	2	0.00021245
Mm.154994	0	0	1	0.000106225
Mm.249164	0	0	1	0.000106225
Mm.371723	0	0	2	0.00021245
Mm.221440	1	0.000502008	1	0.000106225
Mm.315593	2	0.001004016	0	0
Mm.17819	0	0	1	0.000106225
Mm.55143	1	0.000502008	0	0
Mm.269363	0	0	1	0.000106225
Mm.219527	0	0	1	0.000106225
Mm.199964	0	0	3	0.000318674
Mm.254114	0	0	3	0.000318674

Mm.28597	0	0	2	0.00021245
Mm.327932	0	0	1	0.000106225
Mm.9901	0	0	2	0.00021245
Mm.269815	4	0.002008032	3	0.000318674
Mm.35628	2	0.001004016	0	0
Mm.281896	0	0	1	0.000106225
Mm.34693	0	0	3	0.000318674
Mm.357762	1	0.000502008	0	0
Mm.11132	0	0	1	0.000106225
Mm.13430	0	0	2	0.00021245
Mm.298256	0	0	2	0.00021245
Mm.25836	1	0.000502008	0	0
Mm.17510	0	0	2	0.00021245
Mm.273142	0	0	2	0.00021245
Mm.182377	0	0	1	0.000106225
Mm.129136	2	0.001004016	1	0.000106225
Mm.28484	0	0	3	0.000318674
Mm.44220	0	0	2	0.00021245
Mm.248788	0	0	1	0.000106225
Mm.328072	1	0.000502008	1	0.000106225
Mm.166372	0	0	2	0.00021245
Mm.27435	0	0	1	0.000106225
Mm.251638	0	0	1	0.000106225
Mm.328321	0	0	3	0.000318674
Mm.156919	0	0	1	0.000106225
Mm.103439	2	0.001004016	0	0
Mm.27606	0	0	1	0.000106225
Mm.7091	0	0	8	0.000849798
Mm.281887	1	0.000502008	0	0
Mm.239196	0	0	2	0.00021245
Mm.101368	1	0.000502008	0	0
Mm.70980	0	0	1	0.000106225
Mm.269881	0	0	1	0.000106225
Mm.27764	0	0	3	0.000318674
Mm.38868	0	0	2	0.00021245
Mm.335385	1	0.000502008	0	0
Mm.275943	0	0	1	0.000106225
Mm.30181	0	0	4	0.000424899
Mm.260782	0	0	1	0.000106225
Mm.177724	0	0	1	0.000106225
Mm.248778	1	0.000502008	0	0
Mm.41758	0	0	4	0.000424899
Mm.313938	1	0.000502008	0	0
Mm.260060	0	0	3	0.000318674
Mm.30239	0	0	1	0.000106225
Mm.60758	0	0	1	0.000106225
Mm.290166	1	0.000502008	4	0.000424899
Mm.272705	0	0	1	0.000106225
Mm.27289	0	0	4	0.000424899
Mm.282556	0	0	7	0.000743573
Mm.245355	0	0	1	0.000106225
Mm.275191	1	0.000502008	2	0.00021245
Mm.27107	0	0	1	0.000106225
Mm.340095	0	0	1	0.000106225
Mm.215096	0	0	1	0.000106225
Mm.287443	0	0	1	0.000106225

Mm.278878	0	0	1	0.000106225					
Mm.274180	0	0	1	0.000106225					
Mm.29431	0	0	2	0.00021245					
Mm.11827	1	0.000502008	0	0					
Mm.280779	0	0	1	0.000106225					
Mm.232525	0	0	2	0.00021245					
Mm.28231	0	0	2	0.00021245					
Mm.211131	0	0	1	0.000106225					
Mm.100065	0	0	1	0.000106225					
Mm.222825	1	0.000502008	2	0.00021245					
Mm.283293	0	0	3	0.000318674					
Mm.317515	0	0	1	0.000106225					
Mm.280459	0	0	1	0.000106225					
Mm.119714	0	0	1	0.000106225					
Mm.277527	0	0	2	0.00021245					
Mm.291811	0	0	1	0.000106225					
Mm.237103	1	0.000502008	0	0					
Mm.331583	0	0	1	0.000106225					
Mm.313558	0	0	1	0.000106225					
Mm.268027	0	0	1	0.000106225					
Mm.29128	0	0	1	0.000106225					
Mm.173119	0	0	1	0.000106225					
Mm.28037	0	0	1	0.000106225					
Mm.2180	1	0.000502008	21	0.00223072					
Mm.319123	1	0.000502008	1	0.000106225					
Mm.210305	0	0	2	0.00021245					
Mm.29778	0	0	6	0.000637349					
Mm.28712	0	0	2	0.00021245					
Mm.321068	0	0	1	0.000106225					
Mm.272278	0	0	3	0.000318674					
Mm.276721	0	0	2	0.00021245					
Mm.46016	0	0	2	0.00021245					
Mm.292567	0	0	1	0.000106225					
Mm.31283	2	0.001004016	3	0.000318674					
Mm.50109	1	0.000502008	3	0.000318674					
Mm.340943	0	0	2	0.00021245					
Mm.272722	1	0.000502008	1	0.000106225					
Mm.209989	0	0	1	0.000106225					
Mm.154783	0	0	1	0.000106225					
Mm.291624	0	0	2	0.00021245					
Mm.374904	0	0	1	0.000106225					
>GO:0005576	104	0.072625698	811	0.112016575	1.542382065	1.15E-05	0.00105715	extracellular region	C
Mm.275054	0	0	1	0.000106225					
Mm.260144	0	0	1	0.000106225					
Mm.1408	0	0	2	0.00021245					
Mm.42160	2	0.001004016	9	0.000956023					
Mm.290583	0	0	1	0.000106225					
Mm.3433	0	0	1	0.000106225					
Mm.193099	0	0	1	0.000106225					
Mm.3982	0	0	1	0.000106225					
Mm.9714	4	0.002008032	4	0.000424899					
Mm.291831	1	0.000502008	0	0					
Mm.268521	0	0	1	0.000106225					
Mm.233799	0	0	6	0.000637349					
Mm.3825	0	0	1	0.000106225					
Mm.245297	0	0	1	0.000106225					

Mm.282359	0	0	7	0.000743573
Mm.24754	4	0.002008032	6	0.000637349
Mm.154660	0	0	1	0.000106225
Mm.22699	0	0	2	0.00021245
Mm.5090	0	0	1	0.000106225
Mm.344820	0	0	1	0.000106225
Mm.221440	1	0.000502008	1	0.000106225
Mm.315593	2	0.001004016	0	0
Mm.17819	0	0	1	0.000106225
Mm.311912	0	0	1	0.000106225
Mm.55143	1	0.000502008	0	0
Mm.24163	0	0	3	0.000318674
Mm.270393	0	0	1	0.000106225
Mm.205169	0	0	3	0.000318674
Mm.317073	1	0.000502008	0	0
Mm.31283	2	0.001004016	3	0.000318674
Mm.255607	0	0	3	0.000318674
Mm.355306	0	0	1	0.000106225
Mm.214514	0	0	1	0.000106225
Mm.2608	0	0	1	0.000106225
Mm.3819	0	0	1	0.000106225
Mm.4352	0	0	1	0.000106225
Mm.249555	0	0	4	0.000424899
Mm.181021	0	0	4	0.000424899
Mm.2509	1	0.000502008	2	0.00021245
Mm.277792	0	0	1	0.000106225
Mm.287146	0	0	1	0.000106225
Mm.42012	0	0	1	0.000106225
Mm.172674	1	0.000502008	4	0.000424899
Mm.18888	0	0	2	0.00021245
Mm.373531	0	0	2	0.00021245
Mm.4825	0	0	2	0.00021245
Mm.332936	0	0	1	0.000106225
Mm.3057	1	0.000502008	0	0
Mm.291442	0	0	2	0.00021245
Mm.14455	0	0	1	0.000106225
Mm.4871	0	0	1	0.000106225
Mm.1249	0	0	1	0.000106225
Mm.6510	1	0.000502008	8	0.000849798
Mm.1381	0	0	9	0.000956023
Mm.291120	0	0	2	0.00021245
Mm.234129	2	0.001004016	0	0
Mm.224825	1	0.000502008	0	0
Mm.71963	0	0	1	0.000106225
Mm.276367	0	0	1	0.000106225
Mm.272278	0	0	3	0.000318674
Mm.29095	0	0	1	0.000106225
Mm.279437	0	0	3	0.000318674
Mm.298775	0	0	3	0.000318674
Mm.7524	0	0	3	0.000318674
Mm.18709	0	0	1	0.000106225
Mm.338890	1	0.000502008	0	0
Mm.309296	1	0.000502008	0	0
Mm.327591	0	0	1	0.000106225
Mm.257437	1	0.000502008	1	0.000106225
Mm.275608	0	0	1	0.000106225

Mm.316080	0	0	1	0.000106225
Mm.181166	1	0.000502008	0	0
Mm.331182	0	0	1	0.000106225
Mm.230301	0	0	1	0.000106225
Mm.370315	0	0	4	0.000424899
Mm.317515	0	0	1	0.000106225
Mm.8004	1	0.000502008	0	0
Mm.257035	1	0.000502008	0	0
Mm.38993	0	0	1	0.000106225
Mm.287187	0	0	6	0.000637349
Mm.188544	0	0	1	0.000106225
Mm.151819	0	0	1	0.000106225
Mm.335520	1	0.000502008	0	0
Mm.292803	0	0	1	0.000106225
Mm.244319	0	0	1	0.000106225
Mm.17807	0	0	2	0.00021245
Mm.145488	0	0	3	0.000318674
Mm.246440	0	0	1	0.000106225
Mm.250392	0	0	8	0.000849798
Mm.28853	1	0.000502008	7	0.000743573
Mm.2445	0	0	5	0.000531124
Mm.28908	0	0	2	0.00021245
Mm.1114	0	0	2	0.00021245
Mm.195224	0	0	7	0.000743573
Mm.302724	0	0	1	0.000106225
Mm.35691	0	0	1	0.000106225
Mm.29821	0	0	3	0.000318674
Mm.19133	0	0	1	0.000106225
Mm.276137	1	0.000502008	9	0.000956023
Mm.163	0	0	1	0.000106225
Mm.290516	0	0	2	0.00021245
Mm.118034	1	0.000502008	0	0
Mm.237825	0	0	2	0.00021245
Mm.13806	0	0	2	0.00021245
Mm.370172	1	0.000502008	0	0
Mm.1971	0	0	2	0.00021245
Mm.248827	0	0	6	0.000637349
Mm.1641	0	0	1	0.000106225
Mm.4880	0	0	1	0.000106225
Mm.297964	0	0	1	0.000106225
Mm.35605	1	0.000502008	2	0.00021245
Mm.22680	0	0	1	0.000106225
Mm.117180	0	0	1	0.000106225
Mm.298812	0	0	73	0.007754408
Mm.252145	0	0	1	0.000106225
Mm.20837	0	0	1	0.000106225
Mm.200608	1	0.000502008	10	0.001062248
Mm.290924	1	0.000502008	0	0
Mm.272210	0	0	1	0.000106225
Mm.21325	0	0	1	0.000106225
Mm.30041	0	0	2	0.00021245
Mm.180182	0	0	1	0.000106225
Mm.548	0	0	4	0.000424899
Mm.13787	1	0.000502008	0	0
Mm.4263	0	0	1	0.000106225
Mm.236553	0	0	11	0.001168472

Mm.322945	0	0	5	0.000531124
Mm.930	0	0	20	0.002124495
Mm.66222	0	0	1	0.000106225
Mm.316000	1	0.000502008	0	0
Mm.101591	4	0.002008032	3	0.000318674
Mm.167882	0	0	1	0.000106225
Mm.371556	0	0	1	0.000106225
Mm.7236	0	0	3	0.000318674
Mm.18353	0	0	1	0.000106225
Mm.125580	0	0	1	0.000106225
Mm.229532	1	0.000502008	0	0
Mm.7978	0	0	1	0.000106225
Mm.209813	0	0	1	0.000106225
Mm.271976	0	0	2	0.00021245
Mm.9075	0	0	1	0.000106225
Mm.15295	1	0.000502008	0	0
Mm.1650	0	0	1	0.000106225
Mm.309395	1	0.000502008	0	0
Mm.293761	0	0	2	0.00021245
Mm.271717	0	0	1	0.000106225
Mm.150	0	0	1	0.000106225
Mm.22119	0	0	1	0.000106225
Mm.241282	0	0	2	0.00021245
Mm.7996	2	0.001004016	0	0
Mm.16340	0	0	2	0.00021245
Mm.3894	0	0	1	0.000106225
Mm.4913	1	0.000502008	0	0
Mm.243722	0	0	4	0.000424899
Mm.3196	0	0	2	0.00021245
Mm.12468	1	0.000502008	0	0
Mm.45054	2	0.001004016	1	0.000106225
Mm.3711	0	0	4	0.000424899
Mm.88367	0	0	1	0.000106225
Mm.26859	0	0	3	0.000318674
Mm.287807	0	0	1	0.000106225
Mm.230169	0	0	2	0.00021245
Mm.290834	0	0	1	0.000106225
Mm.1568	0	0	3	0.000318674
Mm.263177	0	0	1	0.000106225
Mm.18556	0	0	1	0.000106225
Mm.292208	0	0	9	0.000956023
Mm.27816	0	0	3	0.000318674
Mm.293529	0	0	2	0.00021245
Mm.313345	0	0	1	0.000106225
Mm.22668	0	0	3	0.000318674
Mm.12559	0	0	1	0.000106225
Mm.275742	2	0.001004016	0	0
Mm.233802	0	0	1	0.000106225
Mm.4364	0	0	3	0.000318674
Mm.245210	0	0	1	0.000106225
Mm.6424	0	0	2	0.00021245
Mm.2863	0	0	1	0.000106225
Mm.22398	2	0.001004016	0	0
Mm.142722	0	0	1	0.000106225
Mm.16716	0	0	3	0.000318674
Mm.218846	0	0	1	0.000106225

Mm.9537	0	0	5	0.000531124
Mm.43831	0	0	1	0.000106225
Mm.21981	0	0	6	0.000637349
Mm.1514	1	0.000502008	0	0
Mm.271854	0	0	4	0.000424899
Mm.277661	0	0	5	0.000531124
Mm.3122	0	0	1	0.000106225
Mm.788	0	0	2	0.00021245
Mm.29590	0	0	1	0.000106225
Mm.4259	0	0	5	0.000531124
Mm.249225	0	0	6	0.000637349
Mm.17771	0	0	1	0.000106225
Mm.234654	0	0	18	0.001912046
Mm.263847	0	0	1	0.000106225
Mm.286488	0	0	2	0.00021245
Mm.30045	0	0	2	0.00021245
Mm.291799	0	0	4	0.000424899
Mm.1451	0	0	4	0.000424899
Mm.255116	0	0	1	0.000106225
Mm.21535	0	0	1	0.000106225
Mm.269657	0	0	3	0.000318674
Mm.443	1	0.000502008	0	0
Mm.4974	0	0	1	0.000106225
Mm.258759	1	0.000502008	0	0
Mm.29842	0	0	2	0.00021245
Mm.93335	0	0	4	0.000424899
Mm.16660	0	0	6	0.000637349
Mm.5121	0	0	4	0.000424899
Mm.3401	1	0.000502008	0	0
Mm.296814	0	0	1	0.000106225
Mm.34775	1	0.000502008	4	0.000424899
Mm.5092	0	0	1	0.000106225
Mm.214593	0	0	1	0.000106225
Mm.12616	1	0.000502008	1	0.000106225
Mm.277498	1	0.000502008	9	0.000956023
Mm.220821	0	0	1	0.000106225
Mm.211494	0	0	1	0.000106225
Mm.330764	0	0	1	0.000106225
Mm.24807	0	0	1	0.000106225
Mm.2339	0	0	1	0.000106225
Mm.945	0	0	1	0.000106225
Mm.29855	0	0	1	0.000106225
Mm.287425	0	0	1	0.000106225
Mm.332303	0	0	2	0.00021245
Mm.276776	0	0	1	0.000106225
Mm.194462	0	0	3	0.000318674
Mm.10152	0	0	1	0.000106225
Mm.293517	0	0	3	0.000318674
Mm.15125	0	0	1	0.000106225
Mm.332590	0	0	2	0.00021245
Mm.134093	1	0.000502008	0	0
Mm.260374	1	0.000502008	0	0
Mm.248334	0	0	1	0.000106225
Mm.4954	0	0	2	0.00021245
Mm.272675	0	0	1	0.000106225
Mm.269857	0	0	1	0.000106225

Mm.295230	0	0	4	0.000424899
Mm.288474	3	0.001506024	1	0.000106225
Mm.296169	0	0	1	0.000106225
Mm.14814	0	0	1	0.000106225
Mm.229107	0	0	2	0.00021245
Mm.137222	0	0	1	0.000106225
Mm.18213	2	0.001004016	3	0.000318674
Mm.200775	0	0	1	0.000106225
Mm.159651	0	0	1	0.000106225
Mm.3509	0	0	1	0.000106225
Mm.790	0	0	2	0.00021245
Mm.87773	0	0	8	0.000849798
Mm.198803	1	0.000502008	4	0.000424899
Mm.24430	0	0	2	0.00021245
Mm.3555	0	0	1	0.000106225
Mm.4141	0	0	1	0.000106225
Mm.22718	1	0.000502008	1	0.000106225
Mm.262184	0	0	1	0.000106225
Mm.21109	0	0	3	0.000318674
Mm.283682	1	0.000502008	0	0
Mm.7244	0	0	1	0.000106225
Mm.24044	0	0	2	0.00021245
Mm.227549	0	0	1	0.000106225
Mm.12863	1	0.000502008	1	0.000106225
Mm.334648	0	0	2	0.00021245
Mm.103648	0	0	1	0.000106225
Mm.29183	1	0.000502008	0	0
Mm.95452	0	0	1	0.000106225
Mm.258484	0	0	2	0.00021245
Mm.281356	0	0	2	0.00021245
Mm.154994	0	0	1	0.000106225
Mm.249164	0	0	1	0.000106225
Mm.371723	0	0	2	0.00021245
Mm.269363	0	0	1	0.000106225
Mm.219527	0	0	1	0.000106225
Mm.199964	0	0	3	0.000318674
Mm.254114	0	0	3	0.000318674
Mm.28597	0	0	2	0.00021245
Mm.327932	0	0	1	0.000106225
Mm.9901	0	0	2	0.00021245
Mm.269815	4	0.002008032	3	0.000318674
Mm.35628	2	0.001004016	0	0
Mm.281896	0	0	1	0.000106225
Mm.34693	0	0	3	0.000318674
Mm.357762	1	0.000502008	0	0
Mm.11132	0	0	1	0.000106225
Mm.13430	0	0	2	0.00021245
Mm.298256	0	0	2	0.00021245
Mm.25836	1	0.000502008	0	0
Mm.17510	0	0	2	0.00021245
Mm.273142	0	0	2	0.00021245
Mm.182377	0	0	1	0.000106225
Mm.129136	2	0.001004016	1	0.000106225
Mm.28484	0	0	3	0.000318674
Mm.44220	0	0	2	0.00021245
Mm.248788	0	0	1	0.000106225

Mm.328072	1	0.000502008	1	0.000106225
Mm.166372	0	0	2	0.00021245
Mm.27435	0	0	1	0.000106225
Mm.251638	0	0	1	0.000106225
Mm.328321	0	0	3	0.000318674
Mm.156919	0	0	1	0.000106225
Mm.103439	2	0.001004016	0	0
Mm.27606	0	0	1	0.000106225
Mm.7091	0	0	8	0.000849798
Mm.281887	1	0.000502008	0	0
Mm.239196	0	0	2	0.00021245
Mm.101368	1	0.000502008	0	0
Mm.70980	0	0	1	0.000106225
Mm.269881	0	0	1	0.000106225
Mm.27764	0	0	3	0.000318674
Mm.38868	0	0	2	0.00021245
Mm.335385	1	0.000502008	0	0
Mm.275943	0	0	1	0.000106225
Mm.30181	0	0	4	0.000424899
Mm.260782	0	0	1	0.000106225
Mm.177724	0	0	1	0.000106225
Mm.248778	1	0.000502008	0	0
Mm.41758	0	0	4	0.000424899
Mm.313938	1	0.000502008	0	0
Mm.260060	0	0	3	0.000318674
Mm.30239	0	0	1	0.000106225
Mm.60758	0	0	1	0.000106225
Mm.290166	1	0.000502008	4	0.000424899
Mm.272705	0	0	1	0.000106225
Mm.27289	0	0	4	0.000424899
Mm.282556	0	0	7	0.000743573
Mm.245355	0	0	1	0.000106225
Mm.275191	1	0.000502008	2	0.00021245
Mm.27107	0	0	1	0.000106225
Mm.340095	0	0	1	0.000106225
Mm.215096	0	0	1	0.000106225
Mm.287443	0	0	1	0.000106225
Mm.278878	0	0	1	0.000106225
Mm.274180	0	0	1	0.000106225
Mm.29431	0	0	2	0.00021245
Mm.11827	1	0.000502008	0	0
Mm.280779	0	0	1	0.000106225
Mm.232525	0	0	2	0.00021245
Mm.28231	0	0	2	0.00021245
Mm.211131	0	0	1	0.000106225
Mm.100065	0	0	1	0.000106225
Mm.222825	1	0.000502008	2	0.00021245
Mm.283293	0	0	3	0.000318674
Mm.280459	0	0	1	0.000106225
Mm.119714	0	0	1	0.000106225
Mm.277527	0	0	2	0.00021245
Mm.291811	0	0	1	0.000106225
Mm.237103	1	0.000502008	0	0
Mm.331583	0	0	1	0.000106225
Mm.313558	0	0	1	0.000106225
Mm.268027	0	0	1	0.000106225

Mm.29128	0	0	1	0.000106225					
Mm.173119	0	0	1	0.000106225					
Mm.28037	0	0	1	0.000106225					
Mm.2180	1	0.000502008	21	0.00223072					
Mm.319123	1	0.000502008	1	0.000106225					
Mm.210305	0	0	2	0.00021245					
Mm.29778	0	0	6	0.000637349					
Mm.28712	0	0	2	0.00021245					
Mm.321068	0	0	1	0.000106225					
Mm.276721	0	0	2	0.00021245					
Mm.46016	0	0	2	0.00021245					
Mm.292567	0	0	1	0.000106225					
Mm.50109	1	0.000502008	3	0.000318674					
Mm.340943	0	0	2	0.00021245					
Mm.272722	1	0.000502008	1	0.000106225					
Mm.209989	0	0	1	0.000106225					
Mm.154783	0	0	1	0.000106225					
Mm.291624	0	0	2	0.00021245					
Mm.374904	0	0	1	0.000106225					
>GO:0007166	76	0.053072626	216	0.029834254	0.562140157	1.22E-05	0.001088752	cell surface receptor linked s	P
Mm.24997	2	0.001004016	4	0.000424899					
Mm.35691	0	0	1	0.000106225					
Mm.266871	1	0.000502008	0	0					
Mm.22673	0	0	1	0.000106225					
Mm.150	0	0	1	0.000106225					
Mm.22119	0	0	1	0.000106225					
Mm.243722	0	0	4	0.000424899					
Mm.233802	0	0	1	0.000106225					
Mm.41417	0	0	1	0.000106225					
Mm.213003	0	0	1	0.000106225					
Mm.309954	0	0	1	0.000106225					
Mm.271976	0	0	2	0.00021245					
Mm.88367	0	0	1	0.000106225					
Mm.275742	2	0.001004016	0	0					
Mm.247073	0	0	2	0.00021245					
Mm.3401	1	0.000502008	0	0					
Mm.151940	0	0	1	0.000106225					
Mm.5090	0	0	1	0.000106225					
Mm.224246	0	0	2	0.00021245					
Mm.29891	4	0.002008032	2	0.00021245					
Mm.221688	2	0.001004016	0	0					
Mm.4509	1	0.000502008	1	0.000106225					
Mm.154660	0	0	1	0.000106225					
Mm.268521	0	0	1	0.000106225					
Mm.8681	1	0.000502008	2	0.00021245					
Mm.288726	0	0	2	0.00021245					
Mm.348266	0	0	1	0.000106225					
Mm.39089	0	0	2	0.00021245					
Mm.172346	1	0.000502008	1	0.000106225					
Mm.29790	2	0.001004016	0	0					
Mm.118034	1	0.000502008	0	0					
Mm.237825	0	0	2	0.00021245					
Mm.13806	0	0	2	0.00021245					
Mm.7320	1	0.000502008	1	0.000106225					
Mm.28733	0	0	4	0.000424899					
Mm.223717	0	0	1	0.000106225					

Mm.100399	1	0.000502008	1	0.000106225
Mm.22584	0	0	2	0.00021245
Mm.4913	1	0.000502008	0	0
Mm.945	0	0	1	0.000106225
Mm.29855	0	0	1	0.000106225
Mm.287425	0	0	1	0.000106225
Mm.332303	0	0	2	0.00021245
Mm.332936	0	0	1	0.000106225
Mm.277351	2	0.001004016	0	0
Mm.235018	0	0	1	0.000106225
Mm.192026	0	0	1	0.000106225
Mm.285993	3	0.001506024	9	0.000956023
Mm.329243	0	0	3	0.000318674
Mm.298775	0	0	3	0.000318674
Mm.22680	0	0	1	0.000106225
Mm.342315	0	0	3	0.000318674
Mm.327835	0	0	1	0.000106225
Mm.229532	1	0.000502008	0	0
Mm.28265	0	0	1	0.000106225
Mm.193925	0	0	4	0.000424899
Mm.313181	2	0.001004016	0	0
Mm.254629	0	0	1	0.000106225
Mm.196464	1	0.000502008	1	0.000106225
Mm.195898	0	0	2	0.00021245
Mm.125770	4	0.002008032	4	0.000424899
Mm.2344	0	0	1	0.000106225
Mm.17604	0	0	1	0.000106225
Mm.234342	0	0	1	0.000106225
Mm.329700	0	0	1	0.000106225
Mm.140804	0	0	1	0.000106225
Mm.290834	0	0	1	0.000106225
Mm.279400	0	0	2	0.00021245
Mm.93335	0	0	4	0.000424899
Mm.18509	0	0	1	0.000106225
Mm.28262	4	0.002008032	1	0.000106225
Mm.8055	1	0.000502008	0	0
Mm.103354	2	0.001004016	0	0
Mm.320183	0	0	5	0.000531124
Mm.349120	0	0	1	0.000106225
Mm.83689	1	0.000502008	0	0
Mm.264860	0	0	1	0.000106225
Mm.334648	0	0	2	0.00021245
Mm.29279	0	0	2	0.00021245
Mm.373635	1	0.000502008	5	0.000531124
Mm.70979	1	0.000502008	0	0
Mm.286753	0	0	2	0.00021245
Mm.39863	0	0	1	0.000106225
Mm.298256	0	0	2	0.00021245
Mm.273142	0	0	2	0.00021245
Mm.44606	0	0	1	0.000106225
Mm.103439	2	0.001004016	0	0
Mm.159019	1	0.000502008	0	0
Mm.242413	0	0	1	0.000106225
Mm.272974	0	0	1	0.000106225
Mm.87046	0	0	1	0.000106225
Mm.310036	0	0	2	0.00021245

Mm.325086	1	0.000502008	0	0					
Mm.5305	3	0.001506024	17	0.001805821					
Mm.222685	1	0.000502008	0	0					
Mm.44241	0	0	1	0.000106225					
Mm.8004	1	0.000502008	0	0					
Mm.228	0	0	1	0.000106225					
Mm.201322	1	0.000502008	0	0					
Mm.26150	2	0.001004016	2	0.00021245					
Mm.248291	1	0.000502008	1	0.000106225					
Mm.12091	1	0.000502008	2	0.00021245					
Mm.21739	0	0	2	0.00021245					
Mm.203747	1	0.000502008	0	0					
Mm.159724	0	0	1	0.000106225					
Mm.293761	0	0	2	0.00021245					
Mm.275574	1	0.000502008	0	0					
Mm.4364	0	0	3	0.000318674					
Mm.228798	0	0	1	0.000106225					
Mm.335520	1	0.000502008	0	0					
Mm.275895	0	0	1	0.000106225					
Mm.15969	0	0	2	0.00021245					
Mm.757	2	0.001004016	2	0.00021245					
Mm.254494	0	0	1	0.000106225					
Mm.271674	0	0	3	0.000318674					
Mm.6424	0	0	2	0.00021245					
Mm.224825	1	0.000502008	0	0					
Mm.208855	0	0	2	0.00021245					
Mm.28854	1	0.000502008	0	0					
Mm.272616	0	0	1	0.000106225					
Mm.216227	0	0	1	0.000106225					
Mm.206764	1	0.000502008	4	0.000424899					
Mm.7883	0	0	2	0.00021245					
Mm.23684	0	0	1	0.000106225					
Mm.291928	1	0.000502008	0	0					
Mm.298893	1	0.000502008	0	0					
Mm.51136	0	0	5	0.000531124					
Mm.7405	1	0.000502008	0	0					
Mm.327442	0	0	1	0.000106225					
Mm.321990	0	0	1	0.000106225					
Mm.222723	1	0.000502008	0	0					
Mm.103638	0	0	1	0.000106225					
Mm.20593	2	0.001004016	2	0.00021245					
Mm.55143	1	0.000502008	0	0					
Mm.200770	0	0	1	0.000106225					
Mm.87600	1	0.000502008	0	0					
Mm.209989	0	0	1	0.000106225					
Mm.26908	0	0	8	0.000849798					
Mm.348326	0	0	1	0.000106225					
Mm.275839	2	0.001004016	0	0					
Mm.27804	0	0	2	0.00021245					
Mm.336104	0	0	2	0.00021245					
Mm.374904	0	0	1	0.000106225					
>GO:0009653	101	0.070530726	317	0.04378453	0.620786609	2.14E-05	0.00186178	morphogenesis	P
Mm.3963	0	0	3	0.000318674					
Mm.88367	0	0	1	0.000106225					
Mm.223504	0	0	1	0.000106225					
Mm.210018	0	0	2	0.00021245					

Mm.277812	1	0.000502008	1	0.000106225
Mm.286006	1	0.000502008	0	0
Mm.371610	0	0	1	0.000106225
Mm.21876	1	0.000502008	0	0
Mm.240830	0	0	1	0.000106225
Mm.3057	1	0.000502008	0	0
Mm.4352	0	0	1	0.000106225
Mm.27365	2	0.001004016	0	0
Mm.260076	0	0	4	0.000424899
Mm.285453	0	0	1	0.000106225
Mm.826	3	0.001506024	0	0
Mm.193925	0	0	4	0.000424899
Mm.154915	0	0	2	0.00021245
Mm.279437	0	0	3	0.000318674
Mm.205045	0	0	1	0.000106225
Mm.41637	0	0	1	0.000106225
Mm.34554	1	0.000502008	0	0
Mm.35059	0	0	1	0.000106225
Mm.18213	2	0.001004016	3	0.000318674
Mm.220367	0	0	1	0.000106225
Mm.3982	0	0	1	0.000106225
Mm.233799	0	0	6	0.000637349
Mm.86595	0	0	1	0.000106225
Mm.196508	1	0.000502008	1	0.000106225
Mm.209385	3	0.001506024	2	0.00021245
Mm.311912	0	0	1	0.000106225
Mm.91920	1	0.000502008	0	0
Mm.41077	1	0.000502008	1	0.000106225
Mm.29142	0	0	2	0.00021245
Mm.23957	0	0	1	0.000106225
Mm.354761	0	0	2	0.00021245
Mm.2945	0	0	1	0.000106225
Mm.18626	0	0	1	0.000106225
Mm.1574	0	0	2	0.00021245
Mm.292107	0	0	1	0.000106225
Mm.292510	1	0.000502008	1	0.000106225
Mm.37249	1	0.000502008	0	0
Mm.25203	0	0	1	0.000106225
Mm.245746	2	0.001004016	3	0.000318674
Mm.1022	0	0	10	0.001062248
Mm.251794	0	0	5	0.000531124
Mm.23636	0	0	2	0.00021245
Mm.22680	0	0	1	0.000106225
Mm.3996	0	0	3	0.000318674
Mm.7524	0	0	3	0.000318674
Mm.22398	2	0.001004016	0	0
Mm.22847	0	0	2	0.00021245
Mm.87759	0	0	1	0.000106225
Mm.9684	0	0	1	0.000106225
Mm.294882	0	0	1	0.000106225
Mm.10516	0	0	1	0.000106225
Mm.347883	1	0.000502008	0	0
Mm.5098	3	0.001506024	0	0
Mm.228798	0	0	1	0.000106225
Mm.291928	1	0.000502008	0	0
Mm.321990	0	0	1	0.000106225

Mm.287100	0	0	1	0.000106225
Mm.1399	0	0	1	0.000106225
Mm.153415	1	0.000502008	1	0.000106225
Mm.209813	0	0	1	0.000106225
Mm.181862	1	0.000502008	0	0
Mm.268521	0	0	1	0.000106225
Mm.275742	2	0.001004016	0	0
Mm.223717	0	0	1	0.000106225
Mm.7320	1	0.000502008	1	0.000106225
Mm.100399	1	0.000502008	1	0.000106225
Mm.43358	0	0	4	0.000424899
Mm.30039	1	0.000502008	0	0
Mm.25594	0	0	1	0.000106225
Mm.196581	0	0	2	0.00021245
Mm.8385	0	0	3	0.000318674
Mm.87611	5	0.00251004	6	0.000637349
Mm.200770	0	0	1	0.000106225
Mm.12239	0	0	4	0.000424899
Mm.214514	0	0	1	0.000106225
Mm.289584	2	0.001004016	3	0.000318674
Mm.118034	1	0.000502008	0	0
Mm.4509	1	0.000502008	1	0.000106225
Mm.28265	0	0	1	0.000106225
Mm.273292	1	0.000502008	0	0
Mm.195898	0	0	2	0.00021245
Mm.2018	0	0	1	0.000106225
Mm.288474	3	0.001506024	1	0.000106225
Mm.10214	0	0	2	0.00021245
Mm.29279	0	0	2	0.00021245
Mm.5356	0	0	2	0.00021245
Mm.18509	0	0	1	0.000106225
Mm.16340	0	0	2	0.00021245
Mm.39089	0	0	2	0.00021245
Mm.247566	2	0.001004016	4	0.000424899
Mm.249934	0	0	5	0.000531124
Mm.31927	1	0.000502008	1	0.000106225
Mm.290924	1	0.000502008	0	0
Mm.57223	0	0	1	0.000106225
Mm.259702	0	0	1	0.000106225
Mm.88694	0	0	1	0.000106225
Mm.259318	0	0	1	0.000106225
Mm.330428	2	0.001004016	0	0
Mm.2390	0	0	1	0.000106225
Mm.7978	0	0	1	0.000106225
Mm.293761	0	0	2	0.00021245
Mm.267514	1	0.000502008	1	0.000106225
Mm.7996	2	0.001004016	0	0
Mm.246513	0	0	1	0.000106225
Mm.238213	0	0	1	0.000106225
Mm.4974	0	0	1	0.000106225
Mm.56337	2	0.001004016	1	0.000106225
Mm.287178	0	0	5	0.000531124
Mm.134093	1	0.000502008	0	0
Mm.260374	1	0.000502008	0	0
Mm.288726	0	0	2	0.00021245
Mm.192580	0	0	3	0.000318674

Mm.14297	0	0	1	0.000106225
Mm.254494	0	0	1	0.000106225
Mm.24105	0	0	1	0.000106225
Mm.3451	1	0.000502008	0	0
Mm.245395	0	0	1	0.000106225
Mm.258939	0	0	1	0.000106225
Mm.22699	0	0	2	0.00021245
Mm.24430	0	0	2	0.00021245
Mm.19133	0	0	1	0.000106225
Mm.229532	1	0.000502008	0	0
Mm.298798	0	0	1	0.000106225
Mm.271947	0	0	2	0.00021245
Mm.8681	1	0.000502008	2	0.00021245
Mm.332936	0	0	1	0.000106225
Mm.271898	0	0	5	0.000531124
Mm.217161	0	0	1	0.000106225
Mm.262294	1	0.000502008	0	0
Mm.34871	0	0	5	0.000531124
Mm.4071	0	0	11	0.001168472
Mm.274432	1	0.000502008	0	0
Mm.338720	0	0	2	0.00021245
Mm.3122	0	0	1	0.000106225
Mm.256765	3	0.001506024	0	0
Mm.350712	0	0	1	0.000106225
Mm.213025	0	0	6	0.000637349
Mm.275608	0	0	1	0.000106225
Mm.259021	0	0	3	0.000318674
Mm.337074	0	0	5	0.000531124
Mm.14526	0	0	1	0.000106225
Mm.297109	0	0	2	0.00021245
Mm.271711	0	0	1	0.000106225
Mm.121878	0	0	1	0.000106225
Mm.17917	0	0	1	0.000106225
Mm.295124	0	0	1	0.000106225
Mm.240839	0	0	4	0.000424899
Mm.26834	0	0	1	0.000106225
Mm.28623	0	0	3	0.000318674
Mm.13705	0	0	2	0.00021245
Mm.271770	0	0	1	0.000106225
Mm.757	2	0.001004016	2	0.00021245
Mm.28095	0	0	2	0.00021245
Mm.351459	1	0.000502008	2	0.00021245
Mm.341742	0	0	1	0.000106225
Mm.129746	1	0.000502008	0	0
Mm.321828	0	0	4	0.000424899
Mm.168	1	0.000502008	0	0
Mm.4913	1	0.000502008	0	0
Mm.17715	0	0	1	0.000106225
Mm.272226	1	0.000502008	0	0
Mm.23122	0	0	1	0.000106225
Mm.237825	0	0	2	0.00021245
Mm.925	1	0.000502008	0	0
Mm.198803	1	0.000502008	4	0.000424899
Mm.309395	1	0.000502008	0	0
Mm.348392	0	0	2	0.00021245
Mm.245522	5	0.00251004	3	0.000318674

Mm.260288	0	0	2	0.00021245					
Mm.29790	2	0.001004016	0	0					
Mm.18503	1	0.000502008	0	0					
Mm.103668	1	0.000502008	0	0					
Mm.241282	0	0	2	0.00021245					
Mm.27582	1	0.000502008	0	0					
Mm.288567	0	0	6	0.000637349					
Mm.247073	0	0	2	0.00021245					
Mm.359653	1	0.000502008	3	0.000318674					
Mm.27792	0	0	2	0.00021245					
Mm.133825	2	0.001004016	5	0.000531124					
Mm.124328	0	0	1	0.000106225					
Mm.259333	3	0.001506024	0	0					
Mm.309954	0	0	1	0.000106225					
Mm.21185	0	0	2	0.00021245					
Mm.1894	0	0	1	0.000106225					
Mm.281298	0	0	1	0.000106225					
Mm.16373	0	0	1	0.000106225					
Mm.3825	0	0	1	0.000106225					
Mm.181959	0	0	1	0.000106225					
Mm.275839	2	0.001004016	0	0					
Mm.134191	0	0	1	0.000106225					
Mm.28969	0	0	1	0.000106225					
Mm.249142	1	0.000502008	0	0					
Mm.249342	0	0	2	0.00021245					
Mm.317947	2	0.001004016	0	0					
Mm.238343	0	0	1	0.000106225					
Mm.137222	0	0	1	0.000106225					
Mm.344820	0	0	1	0.000106225					
Mm.28052	0	0	2	0.00021245					
Mm.311337	0	0	3	0.000318674					
>GO:0009566	1	0.000698324	106	0.014640884	20.96574586	2.27E-05	0.001865272	fertilization	P
Mm.210676	0	0	5	0.000531124					
Mm.298812	0	0	73	0.007754408					
Mm.1451	0	0	4	0.000424899					
Mm.299312	0	0	1	0.000106225					
Mm.290692	0	0	5	0.000531124					
Mm.6510	1	0.000502008	8	0.000849798					
Mm.22374	0	0	1	0.000106225					
Mm.1381	0	0	9	0.000956023					
>GO:0007338	1	0.000698324	106	0.014640884	20.96574586	2.27E-05	0.001865272	fertilization (sensu Metazoa)	P
Mm.210676	0	0	5	0.000531124					
Mm.298812	0	0	73	0.007754408					
Mm.1451	0	0	4	0.000424899					
Mm.299312	0	0	1	0.000106225					
Mm.290692	0	0	5	0.000531124					
Mm.6510	1	0.000502008	8	0.000849798					
Mm.22374	0	0	1	0.000106225					
Mm.1381	0	0	9	0.000956023					
>GO:0007242	99	0.069134078	310	0.04281768	0.619342597	2.40E-05	0.001899697	intracellular signaling casca	P
Mm.257073	1	0.000502008	0	0					
Mm.245513	0	0	2	0.00021245					
Mm.184163	1	0.000502008	2	0.00021245					
Mm.252718	0	0	1	0.000106225					
Mm.124502	9	0.004518072	0	0					
Mm.310036	0	0	2	0.00021245					

Mm.309296	1	0.000502008	0	0
Mm.5159	0	0	5	0.000531124
Mm.90218	0	0	2	0.00021245
Mm.280125	0	0	1	0.000106225
Mm.21974	0	0	1	0.000106225
Mm.272203	0	0	2	0.00021245
Mm.5305	3	0.001506024	17	0.001805821
Mm.6900	0	0	1	0.000106225
Mm.289657	2	0.001004016	1	0.000106225
Mm.275839	2	0.001004016	0	0
Mm.271947	0	0	2	0.00021245
Mm.265350	2	0.001004016	0	0
Mm.29872	0	0	1	0.000106225
Mm.33779	0	0	1	0.000106225
Mm.3810	0	0	2	0.00021245
Mm.333471	0	0	1	0.000106225
Mm.259333	3	0.001506024	0	0
Mm.38370	0	0	1	0.000106225
Mm.291554	1	0.000502008	0	0
Mm.329993	0	0	1	0.000106225
Mm.38009	0	0	1	0.000106225
Mm.23963	0	0	4	0.000424899
Mm.212039	1	0.000502008	1	0.000106225
Mm.8681	1	0.000502008	2	0.00021245
Mm.292510	1	0.000502008	1	0.000106225
Mm.5236	0	0	1	0.000106225
Mm.6710	4	0.002008032	1	0.000106225
Mm.86595	0	0	1	0.000106225
Mm.293120	0	0	1	0.000106225
Mm.249934	0	0	5	0.000531124
Mm.20755	1	0.000502008	0	0
Mm.229287	0	0	1	0.000106225
Mm.262067	0	0	1	0.000106225
Mm.227642	3	0.001506024	1	0.000106225
Mm.29515	0	0	2	0.00021245
Mm.217362	1	0.000502008	0	0
Mm.234700	0	0	6	0.000637349
Mm.233813	0	0	3	0.000318674
Mm.275127	0	0	1	0.000106225
Mm.274346	0	0	1	0.000106225
Mm.137134	2	0.001004016	0	0
Mm.279603	0	0	1	0.000106225
Mm.316592	0	0	4	0.000424899
Mm.273804	0	0	2	0.00021245
Mm.46014	0	0	2	0.00021245
Mm.202606	0	0	2	0.00021245
Mm.332231	1	0.000502008	0	0
Mm.286600	0	0	2	0.00021245
Mm.244068	0	0	1	0.000106225
Mm.248291	1	0.000502008	1	0.000106225
Mm.91920	1	0.000502008	0	0
Mm.309954	0	0	1	0.000106225
Mm.28015	1	0.000502008	0	0
Mm.28489	0	0	1	0.000106225
Mm.277354	0	0	1	0.000106225
Mm.89515	1	0.000502008	2	0.00021245

Mm.23957	0	0	1	0.000106225
Mm.354761	0	0	2	0.00021245
Mm.252171	1	0.000502008	2	0.00021245
Mm.28251	0	0	4	0.000424899
Mm.28196	0	0	1	0.000106225
Mm.273379	0	0	5	0.000531124
Mm.275266	0	0	3	0.000318674
Mm.44490	0	0	3	0.000318674
Mm.196208	0	0	1	0.000106225
Mm.264743	0	0	1	0.000106225
Mm.272078	0	0	2	0.00021245
Mm.196692	1	0.000502008	0	0
Mm.28376	0	0	2	0.00021245
Mm.307022	0	0	5	0.000531124
Mm.227583	2	0.001004016	1	0.000106225
Mm.293628	1	0.000502008	0	0
Mm.330160	0	0	13	0.001380922
Mm.193925	0	0	4	0.000424899
Mm.247073	0	0	2	0.00021245
Mm.214593	0	0	1	0.000106225
Mm.223717	0	0	1	0.000106225
Mm.196581	0	0	2	0.00021245
Mm.281298	0	0	1	0.000106225
Mm.330501	1	0.000502008	0	0
Mm.329700	0	0	1	0.000106225
Mm.374904	0	0	1	0.000106225
Mm.5090	0	0	1	0.000106225
Mm.28278	0	0	2	0.00021245
Mm.292040	0	0	3	0.000318674
Mm.286006	1	0.000502008	0	0
Mm.308180	0	0	3	0.000318674
Mm.173337	0	0	1	0.000106225
Mm.203952	0	0	1	0.000106225
Mm.213003	0	0	1	0.000106225
Mm.244393	0	0	1	0.000106225
Mm.22548	0	0	1	0.000106225
Mm.3996	0	0	3	0.000318674
Mm.290924	1	0.000502008	0	0
Mm.318430	0	0	1	0.000106225
Mm.212525	1	0.000502008	0	0
Mm.147226	0	0	1	0.000106225
Mm.1457	0	0	2	0.00021245
Mm.826	3	0.001506024	0	0
Mm.291247	0	0	2	0.00021245
Mm.333868	3	0.001506024	0	0
Mm.371546	1	0.000502008	8	0.000849798
Mm.757	2	0.001004016	2	0.00021245
Mm.27701	0	0	1	0.000106225
Mm.35059	0	0	1	0.000106225
Mm.12723	0	0	1	0.000106225
Mm.34867	0	0	1	0.000106225
Mm.1022	0	0	10	0.001062248
Mm.268397	0	0	1	0.000106225
Mm.153226	0	0	7	0.000743573
Mm.334313	0	0	1	0.000106225
Mm.162811	1	0.000502008	1	0.000106225

Mm.256975	0	0	2	0.00021245				
Mm.271944	0	0	5	0.000531124				
Mm.35727	0	0	1	0.000106225				
Mm.132802	0	0	1	0.000106225				
Mm.28650	2	0.001004016	0	0				
Mm.333233	0	0	1	0.000106225				
Mm.3903	0	0	2	0.00021245				
Mm.322186	0	0	1	0.000106225				
Mm.233009	1	0.000502008	1	0.000106225				
Mm.21936	0	0	1	0.000106225				
Mm.289630	1	0.000502008	1	0.000106225				
Mm.350516	0	0	20	0.002124495				
Mm.22413	0	0	1	0.000106225				
Mm.260376	1	0.000502008	0	0				
Mm.290530	9	0.004518072	11	0.001168472				
Mm.329123	0	0	1	0.000106225				
Mm.207619	0	0	1	0.000106225				
Mm.27968	0	0	1	0.000106225				
Mm.1387	0	0	2	0.00021245				
Mm.22085	0	0	1	0.000106225				
Mm.25306	0	0	3	0.000318674				
Mm.27832	0	0	1	0.000106225				
Mm.276572	1	0.000502008	0	0				
Mm.31486	3	0.001506024	0	0				
Mm.198264	2	0.001004016	2	0.00021245				
Mm.276669	0	0	2	0.00021245				
Mm.179267	0	0	3	0.000318674				
Mm.245675	0	0	1	0.000106225				
Mm.41637	0	0	1	0.000106225				
Mm.325524	0	0	1	0.000106225				
Mm.2344	0	0	1	0.000106225				
Mm.271898	0	0	5	0.000531124				
Mm.247473	1	0.000502008	3	0.000318674				
Mm.197534	0	0	1	0.000106225				
Mm.343951	0	0	1	0.000106225				
Mm.218891	1	0.000502008	0	0				
Mm.3057	1	0.000502008	0	0				
Mm.221440	1	0.000502008	1	0.000106225				
Mm.315593	2	0.001004016	0	0				
Mm.331970	2	0.001004016	1	0.000106225				
Mm.195898	0	0	2	0.00021245				
Mm.125770	4	0.002008032	4	0.000424899				
Mm.18509	0	0	1	0.000106225				
Mm.196464	1	0.000502008	1	0.000106225				
Mm.187079	0	0	2	0.00021245				
Mm.325086	1	0.000502008	0	0				
Mm.222685	1	0.000502008	0	0				
Mm.342315	0	0	3	0.000318674				
Mm.236009	1	0.000502008	0	0				
>GO:0007028	19	0.013268156	251	0.034668508	2.61291073	2.95E-05	0.002286359	cytoplasm organization and P
Mm.223744	0	0	1	0.000106225				
Mm.279603	0	0	1	0.000106225				
Mm.193099	0	0	1	0.000106225				
Mm.257482	0	0	3	0.000318674				
Mm.29061	0	0	1	0.000106225				
Mm.27546	0	0	4	0.000424899				

Mm.251255	0	0	3	0.000318674
Mm.4283	0	0	1	0.000106225
Mm.29046	0	0	2	0.00021245
Mm.316592	0	0	4	0.000424899
Mm.307846	0	0	4	0.000424899
Mm.12144	1	0.000502008	0	0
Mm.306548	0	0	1	0.000106225
Mm.260737	0	0	5	0.000531124
Mm.300263	0	0	3	0.000318674
Mm.325521	0	0	5	0.000531124
Mm.21529	1	0.000502008	1	0.000106225
Mm.371576	0	0	1	0.000106225
Mm.323357	0	0	4	0.000424899
Mm.354330	0	0	10	0.001062248
Mm.4419	0	0	1	0.000106225
Mm.262021	0	0	18	0.001912046
Mm.282053	0	0	14	0.001487147
Mm.290772	0	0	1	0.000106225
Mm.289868	0	0	9	0.000956023
Mm.300271	0	0	1	0.000106225
Mm.353923	0	0	6	0.000637349
Mm.43778	0	0	7	0.000743573
Mm.643	0	0	7	0.000743573
Mm.702	0	0	1	0.000106225
Mm.371578	0	0	2	0.00021245
Mm.300281	0	0	1	0.000106225
Mm.35583	0	0	1	0.000106225
Mm.16775	3	0.001506024	11	0.001168472
Mm.154915	0	0	2	0.00021245
Mm.331113	0	0	11	0.001168472
Mm.6957	0	0	6	0.000637349
Mm.66	0	0	21	0.00223072
Mm.5291	2	0.001004016	17	0.001805821
Mm.325584	0	0	3	0.000318674
Mm.301827	0	0	1	0.000106225
Mm.371579	0	0	5	0.000531124
Mm.260904	0	0	4	0.000424899
Mm.312227	0	0	1	0.000106225
Mm.250030	0	0	11	0.001168472
Mm.319719	0	0	2	0.00021245
Mm.29810	0	0	2	0.00021245
Mm.220367	0	0	1	0.000106225
Mm.28659	0	0	1	0.000106225
Mm.29363	0	0	3	0.000318674
Mm.28057	5	0.00251004	2	0.00021245
Mm.29906	0	0	2	0.00021245
Mm.41800	0	0	1	0.000106225
Mm.27871	0	0	2	0.00021245
Mm.262707	0	0	1	0.000106225
Mm.21062	1	0.000502008	1	0.000106225
Mm.12864	0	0	1	0.000106225
Mm.38344	0	0	2	0.00021245
Mm.197555	1	0.000502008	2	0.00021245
Mm.239997	1	0.000502008	0	0
Mm.275788	1	0.000502008	0	0
Mm.259121	0	0	2	0.00021245

Mm.29253	3	0.001506024	1	0.000106225					
Mm.28630	0	0	2	0.00021245					
Mm.297109	0	0	2	0.00021245					
Mm.260076	0	0	4	0.000424899					
>GO:0045143	11	0.007681564	8	0.001104972	0.143847313	4.98E-05	0.003409477	homologous chromosome s	P
Mm.288324	0	0	4	0.000424899					
Mm.6856	11	0.005522088	4	0.000424899					
>GO:0045132	11	0.007681564	8	0.001104972	0.143847313	4.98E-05	0.003409477	meiotic chromosome segreg	P
Mm.288324	0	0	4	0.000424899					
Mm.6856	11	0.005522088	4	0.000424899					
>GO:0009888	18	0.012569832	27	0.003729282	0.296685083	5.05E-05	0.003409477	histogenesis	P
Mm.237825	0	0	2	0.00021245					
Mm.291928	1	0.000502008	0	0					
Mm.925	1	0.000502008	0	0					
Mm.198803	1	0.000502008	4	0.000424899					
Mm.309395	1	0.000502008	0	0					
Mm.348392	0	0	2	0.00021245					
Mm.245522	5	0.00251004	3	0.000318674					
Mm.260288	0	0	2	0.00021245					
Mm.7320	1	0.000502008	1	0.000106225					
Mm.30039	1	0.000502008	0	0					
Mm.29790	2	0.001004016	0	0					
Mm.2018	0	0	1	0.000106225					
Mm.288474	3	0.001506024	1	0.000106225					
Mm.10214	0	0	2	0.00021245					
Mm.29279	0	0	2	0.00021245					
Mm.5356	0	0	2	0.00021245					
Mm.18509	0	0	1	0.000106225					
Mm.16340	0	0	2	0.00021245					
Mm.18503	1	0.000502008	0	0					
Mm.103668	1	0.000502008	0	0					
Mm.39089	0	0	2	0.00021245					
>GO:0016881	55	0.038407821	147	0.020303867	0.528638875	5.03E-05	0.003409477	acid-amino acid ligase activ	F
Mm.273270	1	0.000502008	0	0					
Mm.78312	0	0	1	0.000106225					
Mm.258977	0	0	1	0.000106225					
Mm.362087	0	0	1	0.000106225					
Mm.28792	0	0	2	0.00021245					
Mm.2026	0	0	4	0.000424899					
Mm.259879	1	0.000502008	0	0					
Mm.289584	2	0.001004016	3	0.000318674					
Mm.266871	1	0.000502008	0	0					
Mm.272203	0	0	2	0.00021245					
Mm.209265	5	0.00251004	0	0					
Mm.291326	0	0	1	0.000106225					
Mm.22670	1	0.000502008	0	0					
Mm.18706	1	0.000502008	2	0.00021245					
Mm.246750	0	0	1	0.000106225					
Mm.279923	0	0	3	0.000318674					
Mm.8211	1	0.000502008	0	0					
Mm.314056	0	0	2	0.00021245					
Mm.22030	0	0	3	0.000318674					
Mm.21281	1	0.000502008	2	0.00021245					
Mm.28235	0	0	3	0.000318674					
Mm.324553	0	0	2	0.00021245					
Mm.2847	2	0.001004016	1	0.000106225					

Mm.321227	0	0	1	0.000106225					
Mm.41063	0	0	1	0.000106225					
Mm.15701	0	0	7	0.000743573					
Mm.1485	0	0	3	0.000318674					
Mm.3074	0	0	2	0.00021245					
Mm.240044	2	0.001004016	0	0					
Mm.172835	2	0.001004016	0	0					
Mm.9002	2	0.001004016	2	0.00021245					
Mm.275195	0	0	2	0.00021245					
Mm.18210	0	0	6	0.000637349					
Mm.250262	1	0.000502008	0	0					
Mm.310	0	0	1	0.000106225					
Mm.332268	0	0	1	0.000106225					
Mm.305925	4	0.002008032	1	0.000106225					
Mm.290447	0	0	3	0.000318674					
Mm.274360	0	0	2	0.00021245					
Mm.328135	2	0.001004016	4	0.000424899					
Mm.172605	0	0	2	0.00021245					
Mm.287783	1	0.000502008	0	0					
Mm.311110	0	0	3	0.000318674					
Mm.319512	0	0	4	0.000424899					
Mm.270484	0	0	3	0.000318674					
Mm.29405	0	0	4	0.000424899					
Mm.180052	1	0.000502008	9	0.000956023					
Mm.228903	0	0	6	0.000637349					
Mm.27372	0	0	1	0.000106225					
Mm.166372	0	0	2	0.00021245					
Mm.288924	2	0.001004016	2	0.00021245					
Mm.256765	3	0.001506024	0	0					
Mm.49884	14	0.007028112	6	0.000637349					
Mm.27764	0	0	3	0.000318674					
Mm.294777	1	0.000502008	10	0.001062248					
Mm.340315	0	0	4	0.000424899					
Mm.207678	0	0	2	0.00021245					
Mm.26194	0	0	3	0.000318674					
Mm.28930	1	0.000502008	1	0.000106225					
Mm.159453	1	0.000502008	1	0.000106225					
Mm.89830	0	0	3	0.000318674					
Mm.275426	0	0	3	0.000318674					
Mm.262859	1	0.000502008	0	0					
Mm.275574	1	0.000502008	0	0					
Mm.22225	0	0	1	0.000106225					
Mm.98668	0	0	1	0.000106225					
Mm.274843	0	0	2	0.00021245					
Mm.326945	0	0	1	0.000106225					
>GO:0016879	58	0.040502793	158	0.021823204	0.538807392	5.09E-05	0.003409477	ligase activity, forming carb(	F
Mm.1815	0	0	1	0.000106225					
Mm.338021	1	0.000502008	0	0					
Mm.3217	0	0	3	0.000318674					
Mm.335385	1	0.000502008	0	0					
Mm.29584	0	0	1	0.000106225					
Mm.4505	0	0	1	0.000106225					
Mm.273270	1	0.000502008	0	0					
Mm.78312	0	0	1	0.000106225					
Mm.258977	0	0	1	0.000106225					
Mm.362087	0	0	1	0.000106225					

Mm.28792	0	0	2	0.00021245
Mm.2026	0	0	4	0.000424899
Mm.259879	1	0.000502008	0	0
Mm.289584	2	0.001004016	3	0.000318674
Mm.266871	1	0.000502008	0	0
Mm.272203	0	0	2	0.00021245
Mm.209265	5	0.00251004	0	0
Mm.291326	0	0	1	0.000106225
Mm.22670	1	0.000502008	0	0
Mm.18706	1	0.000502008	2	0.00021245
Mm.246750	0	0	1	0.000106225
Mm.279923	0	0	3	0.000318674
Mm.8211	1	0.000502008	0	0
Mm.314056	0	0	2	0.00021245
Mm.22030	0	0	3	0.000318674
Mm.21281	1	0.000502008	2	0.00021245
Mm.28235	0	0	3	0.000318674
Mm.324553	0	0	2	0.00021245
Mm.2847	2	0.001004016	1	0.000106225
Mm.321227	0	0	1	0.000106225
Mm.41063	0	0	1	0.000106225
Mm.15701	0	0	7	0.000743573
Mm.1485	0	0	3	0.000318674
Mm.3074	0	0	2	0.00021245
Mm.240044	2	0.001004016	0	0
Mm.172835	2	0.001004016	0	0
Mm.9002	2	0.001004016	2	0.00021245
Mm.275195	0	0	2	0.00021245
Mm.18210	0	0	6	0.000637349
Mm.250262	1	0.000502008	0	0
Mm.310	0	0	1	0.000106225
Mm.332268	0	0	1	0.000106225
Mm.305925	4	0.002008032	1	0.000106225
Mm.290447	0	0	3	0.000318674
Mm.274360	0	0	2	0.00021245
Mm.328135	2	0.001004016	4	0.000424899
Mm.172605	0	0	2	0.00021245
Mm.287783	1	0.000502008	0	0
Mm.311110	0	0	3	0.000318674
Mm.319512	0	0	4	0.000424899
Mm.270484	0	0	3	0.000318674
Mm.29405	0	0	4	0.000424899
Mm.180052	1	0.000502008	9	0.000956023
Mm.228903	0	0	6	0.000637349
Mm.27372	0	0	1	0.000106225
Mm.166372	0	0	2	0.00021245
Mm.288924	2	0.001004016	2	0.00021245
Mm.256765	3	0.001506024	0	0
Mm.49884	14	0.007028112	6	0.000637349
Mm.27764	0	0	3	0.000318674
Mm.294777	1	0.000502008	10	0.001062248
Mm.340315	0	0	4	0.000424899
Mm.207678	0	0	2	0.00021245
Mm.26194	0	0	3	0.000318674
Mm.28930	1	0.000502008	1	0.000106225
Mm.159453	1	0.000502008	1	0.000106225

Mm.89830	0	0	3	0.000318674				
Mm.275426	0	0	3	0.000318674				
Mm.262859	1	0.000502008	0	0				
Mm.275574	1	0.000502008	0	0				
Mm.22225	0	0	1	0.000106225				
Mm.98668	0	0	1	0.000106225				
Mm.274843	0	0	2	0.00021245				
Mm.326945	0	0	1	0.000106225				
Mm.52275	1	0.000502008	0	0				
Mm.287178	0	0	5	0.000531124				
>GO:0004842	55	0.038407821	147	0.020303867	0.528638875	5.03E-05	0.003409477	ubiquitin-protein ligase activ F
Mm.273270	1	0.000502008	0	0				
Mm.78312	0	0	1	0.000106225				
Mm.258977	0	0	1	0.000106225				
Mm.362087	0	0	1	0.000106225				
Mm.28792	0	0	2	0.00021245				
Mm.2026	0	0	4	0.000424899				
Mm.259879	1	0.000502008	0	0				
Mm.289584	2	0.001004016	3	0.000318674				
Mm.266871	1	0.000502008	0	0				
Mm.272203	0	0	2	0.00021245				
Mm.209265	5	0.00251004	0	0				
Mm.291326	0	0	1	0.000106225				
Mm.22670	1	0.000502008	0	0				
Mm.18706	1	0.000502008	2	0.00021245				
Mm.246750	0	0	1	0.000106225				
Mm.279923	0	0	3	0.000318674				
Mm.8211	1	0.000502008	0	0				
Mm.314056	0	0	2	0.00021245				
Mm.22030	0	0	3	0.000318674				
Mm.21281	1	0.000502008	2	0.00021245				
Mm.28235	0	0	3	0.000318674				
Mm.324553	0	0	2	0.00021245				
Mm.2847	2	0.001004016	1	0.000106225				
Mm.321227	0	0	1	0.000106225				
Mm.41063	0	0	1	0.000106225				
Mm.15701	0	0	7	0.000743573				
Mm.1485	0	0	3	0.000318674				
Mm.3074	0	0	2	0.00021245				
Mm.240044	2	0.001004016	0	0				
Mm.172835	2	0.001004016	0	0				
Mm.9002	2	0.001004016	2	0.00021245				
Mm.275195	0	0	2	0.00021245				
Mm.18210	0	0	6	0.000637349				
Mm.250262	1	0.000502008	0	0				
Mm.310	0	0	1	0.000106225				
Mm.332268	0	0	1	0.000106225				
Mm.305925	4	0.002008032	1	0.000106225				
Mm.290447	0	0	3	0.000318674				
Mm.274360	0	0	2	0.00021245				
Mm.328135	2	0.001004016	4	0.000424899				
Mm.172605	0	0	2	0.00021245				
Mm.287783	1	0.000502008	0	0				
Mm.311110	0	0	3	0.000318674				
Mm.319512	0	0	4	0.000424899				
Mm.270484	0	0	3	0.000318674				

Mm.29405	0	0	4	0.000424899					
Mm.180052	1	0.000502008	9	0.000956023					
Mm.228903	0	0	6	0.000637349					
Mm.27372	0	0	1	0.000106225					
Mm.166372	0	0	2	0.00021245					
Mm.288924	2	0.001004016	2	0.00021245					
Mm.256765	3	0.001506024	0	0					
Mm.49884	14	0.007028112	6	0.000637349					
Mm.27764	0	0	3	0.000318674					
Mm.294777	1	0.000502008	10	0.001062248					
Mm.340315	0	0	4	0.000424899					
Mm.207678	0	0	2	0.00021245					
Mm.26194	0	0	3	0.000318674					
Mm.28930	1	0.000502008	1	0.000106225					
Mm.159453	1	0.000502008	1	0.000106225					
Mm.89830	0	0	3	0.000318674					
Mm.275426	0	0	3	0.000318674					
Mm.262859	1	0.000502008	0	0					
Mm.275574	1	0.000502008	0	0					
Mm.22225	0	0	1	0.000106225					
Mm.98668	0	0	1	0.000106225					
Mm.274843	0	0	2	0.00021245					
Mm.326945	0	0	1	0.000106225					
>GO:0009719	41	0.028631285	98	0.013535912	0.472766474	5.32E-05	0.0035004	response to endogenous sti	P
Mm.312323	0	0	1	0.000106225					
Mm.277779	0	0	1	0.000106225					
Mm.203	0	0	4	0.000424899					
Mm.236256	1	0.000502008	0	0					
Mm.16753	4	0.002008032	1	0.000106225					
Mm.36524	0	0	2	0.00021245					
Mm.282335	0	0	4	0.000424899					
Mm.288809	0	0	1	0.000106225					
Mm.22700	1	0.000502008	1	0.000106225					
Mm.42201	1	0.000502008	0	0					
Mm.288179	0	0	1	0.000106225					
Mm.277136	0	0	1	0.000106225					
Mm.4619	0	0	4	0.000424899					
Mm.18210	0	0	6	0.000637349					
Mm.35061	0	0	1	0.000106225					
Mm.71	0	0	1	0.000106225					
Mm.182628	0	0	3	0.000318674					
Mm.196846	0	0	1	0.000106225					
Mm.12145	0	0	4	0.000424899					
Mm.185467	0	0	1	0.000106225					
Mm.298456	0	0	2	0.00021245					
Mm.262117	0	0	1	0.000106225					
Mm.172835	2	0.001004016	0	0					
Mm.1393	0	0	1	0.000106225					
Mm.10141	2	0.001004016	4	0.000424899					
Mm.4347	0	0	3	0.000318674					
Mm.246010	0	0	3	0.000318674					
Mm.26412	5	0.00251004	2	0.00021245					
Mm.126976	0	0	2	0.00021245					
Mm.358656	0	0	1	0.000106225					
Mm.6856	11	0.005522088	4	0.000424899					
Mm.287837	0	0	1	0.000106225					

Mm.259294	1	0.000502008	0	0					
Mm.23122	0	0	1	0.000106225					
Mm.23739	0	0	2	0.00021245					
Mm.206921	0	0	2	0.00021245					
Mm.216227	0	0	1	0.000106225					
Mm.281482	0	0	1	0.000106225					
Mm.289915	2	0.001004016	2	0.00021245					
Mm.2952	0	0	3	0.000318674					
Mm.259278	1	0.000502008	2	0.00021245					
Mm.23267	0	0	1	0.000106225					
Mm.272226	1	0.000502008	0	0					
Mm.272989	1	0.000502008	0	0					
Mm.173953	2	0.001004016	2	0.00021245					
Mm.41447	1	0.000502008	0	0					
Mm.323072	0	0	1	0.000106225					
Mm.7141	0	0	2	0.00021245					
Mm.16549	0	0	2	0.00021245					
Mm.2805	1	0.000502008	0	0					
Mm.22117	0	0	3	0.000318674					
Mm.8681	1	0.000502008	2	0.00021245					
Mm.212462	0	0	1	0.000106225					
Mm.311337	0	0	3	0.000318674					
Mm.233734	0	0	1	0.000106225					
Mm.341972	0	0	3	0.000318674					
Mm.221440	1	0.000502008	1	0.000106225					
Mm.315593	2	0.001004016	0	0					
>GO:0042254	19	0.013268156	242	0.033425414	2.519220704	6.48E-05	0.004180773	ribosome biogenesis and as	P
Mm.257482	0	0	3	0.000318674					
Mm.29061	0	0	1	0.000106225					
Mm.27546	0	0	4	0.000424899					
Mm.251255	0	0	3	0.000318674					
Mm.4283	0	0	1	0.000106225					
Mm.29046	0	0	2	0.00021245					
Mm.316592	0	0	4	0.000424899					
Mm.307846	0	0	4	0.000424899					
Mm.12144	1	0.000502008	0	0					
Mm.306548	0	0	1	0.000106225					
Mm.260737	0	0	5	0.000531124					
Mm.300263	0	0	3	0.000318674					
Mm.325521	0	0	5	0.000531124					
Mm.21529	1	0.000502008	1	0.000106225					
Mm.371576	0	0	1	0.000106225					
Mm.323357	0	0	4	0.000424899					
Mm.354330	0	0	10	0.001062248					
Mm.4419	0	0	1	0.000106225					
Mm.262021	0	0	18	0.001912046					
Mm.282053	0	0	14	0.001487147					
Mm.290772	0	0	1	0.000106225					
Mm.289868	0	0	9	0.000956023					
Mm.300271	0	0	1	0.000106225					
Mm.353923	0	0	6	0.000637349					
Mm.43778	0	0	7	0.000743573					
Mm.643	0	0	7	0.000743573					
Mm.702	0	0	1	0.000106225					
Mm.371578	0	0	2	0.00021245					
Mm.300281	0	0	1	0.000106225					

Mm.35583	0	0	1	0.000106225					
Mm.16775	3	0.001506024	11	0.001168472					
Mm.154915	0	0	2	0.00021245					
Mm.331113	0	0	11	0.001168472					
Mm.6957	0	0	6	0.000637349					
Mm.66	0	0	21	0.00223072					
Mm.5291	2	0.001004016	17	0.001805821					
Mm.325584	0	0	3	0.000318674					
Mm.301827	0	0	1	0.000106225					
Mm.371579	0	0	5	0.000531124					
Mm.260904	0	0	4	0.000424899					
Mm.312227	0	0	1	0.000106225					
Mm.250030	0	0	11	0.001168472					
Mm.319719	0	0	2	0.00021245					
Mm.29810	0	0	2	0.00021245					
Mm.220367	0	0	1	0.000106225					
Mm.28659	0	0	1	0.000106225					
Mm.29363	0	0	3	0.000318674					
Mm.28057	5	0.00251004	2	0.00021245					
Mm.29906	0	0	2	0.00021245					
Mm.41800	0	0	1	0.000106225					
Mm.27871	0	0	2	0.00021245					
Mm.262707	0	0	1	0.000106225					
Mm.21062	1	0.000502008	1	0.000106225					
Mm.12864	0	0	1	0.000106225					
Mm.38344	0	0	2	0.00021245					
Mm.197555	1	0.000502008	2	0.00021245					
Mm.239997	1	0.000502008	0	0					
Mm.275788	1	0.000502008	0	0					
Mm.259121	0	0	2	0.00021245					
Mm.29253	3	0.001506024	1	0.000106225					
Mm.28630	0	0	2	0.00021245					
>GO:0000080	7	0.004888268	2	0.000276243	0.056511444	8.73E-05	0.005156221	G1 phase of mitotic cell cycl	P
Mm.22670	1	0.000502008	0	0					
Mm.292470	6	0.003012048	2	0.00021245					
>GO:0007089	7	0.004888268	2	0.000276243	0.056511444	8.73E-05	0.005156221	traversing start control point	P
Mm.22670	1	0.000502008	0	0					
Mm.292470	6	0.003012048	2	0.00021245					
>GO:0006265	7	0.004888268	2	0.000276243	0.056511444	8.73E-05	0.005156221	DNA topological change	P
Mm.217233	7	0.003514056	0	0					
Mm.4237	0	0	2	0.00021245					
>GO:0051318	7	0.004888268	2	0.000276243	0.056511444	8.73E-05	0.005156221	G1 phase	P
Mm.22670	1	0.000502008	0	0					
Mm.292470	6	0.003012048	2	0.00021245					
>GO:0003916	7	0.004888268	2	0.000276243	0.056511444	8.73E-05	0.005156221	DNA topoisomerase activity	F
Mm.217233	7	0.003514056	0	0					
Mm.4237	0	0	2	0.00021245					
>GO:0016829	1	0.000698324	91	0.012569061	17.99889503	0.000111032	0.006343368	lyase activity	F
Mm.28301	0	0	2	0.00021245					
Mm.23869	0	0	1	0.000106225					
Mm.154581	0	0	3	0.000318674					
Mm.310036	0	0	2	0.00021245					
Mm.38151	0	0	2	0.00021245					
Mm.275831	0	0	1	0.000106225					
Mm.7729	0	0	1	0.000106225					
Mm.203	0	0	4	0.000424899					

Mm.1641	0	0	1	0.000106225					
Mm.206417	0	0	3	0.000318674					
Mm.26237	0	0	5	0.000531124					
Mm.22668	0	0	3	0.000318674					
Mm.277857	0	0	6	0.000637349					
Mm.6988	0	0	1	0.000106225					
Mm.34102	0	0	19	0.002018271					
Mm.61526	0	0	5	0.000531124					
Mm.283682	1	0.000502008	0	0					
Mm.296382	0	0	4	0.000424899					
Mm.216089	0	0	2	0.00021245					
Mm.107869	0	0	3	0.000318674					
Mm.24887	0	0	2	0.00021245					
Mm.29815	0	0	12	0.001274697					
Mm.287425	0	0	1	0.000106225					
Mm.332936	0	0	1	0.000106225					
Mm.24603	0	0	5	0.000531124					
Mm.282499	0	0	1	0.000106225					
Mm.245739	0	0	1	0.000106225					
>GO:0007046	19	0.013268156	235	0.032458564	2.446350683	0.000118404	0.006655437	ribosome biogenesis	P
Mm.251255	0	0	3	0.000318674					
Mm.4283	0	0	1	0.000106225					
Mm.29046	0	0	2	0.00021245					
Mm.316592	0	0	4	0.000424899					
Mm.307846	0	0	4	0.000424899					
Mm.12144	1	0.000502008	0	0					
Mm.306548	0	0	1	0.000106225					
Mm.260737	0	0	5	0.000531124					
Mm.300263	0	0	3	0.000318674					
Mm.325521	0	0	5	0.000531124					
Mm.21529	1	0.000502008	1	0.000106225					
Mm.371576	0	0	1	0.000106225					
Mm.323357	0	0	4	0.000424899					
Mm.354330	0	0	10	0.001062248					
Mm.4419	0	0	1	0.000106225					
Mm.262021	0	0	18	0.001912046					
Mm.282053	0	0	14	0.001487147					
Mm.290772	0	0	1	0.000106225					
Mm.289868	0	0	9	0.000956023					
Mm.300271	0	0	1	0.000106225					
Mm.353923	0	0	6	0.000637349					
Mm.43778	0	0	7	0.000743573					
Mm.643	0	0	7	0.000743573					
Mm.702	0	0	1	0.000106225					
Mm.371578	0	0	2	0.00021245					
Mm.300281	0	0	1	0.000106225					
Mm.35583	0	0	1	0.000106225					
Mm.16775	3	0.001506024	11	0.001168472					
Mm.154915	0	0	2	0.00021245					
Mm.331113	0	0	11	0.001168472					
Mm.6957	0	0	6	0.000637349					
Mm.66	0	0	21	0.00223072					
Mm.5291	2	0.001004016	17	0.001805821					
Mm.325584	0	0	3	0.000318674					
Mm.301827	0	0	1	0.000106225					
Mm.371579	0	0	5	0.000531124					

Mm.260904	0	0	4	0.000424899				
Mm.312227	0	0	1	0.000106225				
Mm.250030	0	0	11	0.001168472				
Mm.319719	0	0	2	0.00021245				
Mm.29810	0	0	2	0.00021245				
Mm.220367	0	0	1	0.000106225				
Mm.29061	0	0	1	0.000106225				
Mm.28659	0	0	1	0.000106225				
Mm.29363	0	0	3	0.000318674				
Mm.28057	5	0.00251004	2	0.00021245				
Mm.29906	0	0	2	0.00021245				
Mm.41800	0	0	1	0.000106225				
Mm.27871	0	0	2	0.00021245				
Mm.262707	0	0	1	0.000106225				
Mm.21062	1	0.000502008	1	0.000106225				
Mm.12864	0	0	1	0.000106225				
Mm.38344	0	0	2	0.00021245				
Mm.197555	1	0.000502008	2	0.00021245				
Mm.239997	1	0.000502008	0	0				
Mm.275788	1	0.000502008	0	0				
Mm.259121	0	0	2	0.00021245				
Mm.29253	3	0.001506024	1	0.000106225				
Mm.28630	0	0	2	0.00021245				
>GO:0006511	47	0.032821229	123	0.01698895	0.517620783	0.000120831	0.006684079	ubiquitin-dependent protein P
Mm.119717	1	0.000502008	1	0.000106225				
Mm.252255	1	0.000502008	0	0				
Mm.32912	0	0	3	0.000318674				
Mm.368	0	0	3	0.000318674				
Mm.8911	0	0	1	0.000106225				
Mm.98	0	0	1	0.000106225				
Mm.143818	0	0	4	0.000424899				
Mm.324553	0	0	2	0.00021245				
Mm.2847	2	0.001004016	1	0.000106225				
Mm.1485	0	0	3	0.000318674				
Mm.3074	0	0	2	0.00021245				
Mm.240044	2	0.001004016	0	0				
Mm.1104	2	0.001004016	2	0.00021245				
Mm.172835	2	0.001004016	0	0				
Mm.9002	2	0.001004016	2	0.00021245				
Mm.275195	0	0	2	0.00021245				
Mm.29807	6	0.003012048	8	0.000849798				
Mm.3571	0	0	1	0.000106225				
Mm.237594	0	0	1	0.000106225				
Mm.3974	0	0	3	0.000318674				
Mm.242646	2	0.001004016	1	0.000106225				
Mm.80484	0	0	1	0.000106225				
Mm.21630	1	0.000502008	0	0				
Mm.38976	0	0	3	0.000318674				
Mm.305925	4	0.002008032	1	0.000106225				
Mm.290447	0	0	3	0.000318674				
Mm.121265	0	0	6	0.000637349				
Mm.30270	0	0	2	0.00021245				
Mm.208883	0	0	2	0.00021245				
Mm.30210	0	0	6	0.000637349				
Mm.21728	0	0	3	0.000318674				
Mm.22233	0	0	1	0.000106225				

Mm.172605	0	0	2	0.00021245					
Mm.24608	0	0	3	0.000318674					
Mm.251174	0	0	3	0.000318674					
Mm.275970	0	0	1	0.000106225					
Mm.319512	0	0	4	0.000424899					
Mm.272770	0	0	2	0.00021245					
Mm.261004	0	0	1	0.000106225					
Mm.180052	1	0.000502008	9	0.000956023					
Mm.329277	0	0	3	0.000318674					
Mm.288924	2	0.001004016	2	0.00021245					
Mm.256765	3	0.001506024	0	0					
Mm.49884	14	0.007028112	6	0.000637349					
Mm.340315	0	0	4	0.000424899					
Mm.89830	0	0	3	0.000318674					
Mm.13437	2	0.001004016	2	0.00021245					
Mm.16974	0	0	5	0.000531124					
Mm.371667	0	0	3	0.000318674					
Mm.21596	0	0	1	0.000106225					
>GO:0005554	105	0.073324022	349	0.04820442	0.657416469	0.000125811	0.006850823	molecular_function unknowı	F
Mm.28722	4	0.002008032	7	0.000743573					
Mm.318846	1	0.000502008	0	0					
Mm.11285	0	0	1	0.000106225					
Mm.327439	1	0.000502008	4	0.000424899					
Mm.58882	0	0	1	0.000106225					
Mm.265874	0	0	1	0.000106225					
Mm.172176	0	0	1	0.000106225					
Mm.129746	1	0.000502008	0	0					
Mm.277939	0	0	1	0.000106225					
Mm.318248	2	0.001004016	1	0.000106225					
Mm.174256	0	0	2	0.00021245					
Mm.27747	0	0	1	0.000106225					
Mm.275309	0	0	2	0.00021245					
Mm.34046	0	0	1	0.000106225					
Mm.28131	0	0	1	0.000106225					
Mm.27155	0	0	2	0.00021245					
Mm.373524	0	0	1	0.000106225					
Mm.236139	0	0	1	0.000106225					
Mm.233082	1	0.000502008	0	0					
Mm.5045	0	0	1	0.000106225					
Mm.12468	1	0.000502008	0	0					
Mm.285723	0	0	1	0.000106225					
Mm.1775	0	0	1	0.000106225					
Mm.262294	1	0.000502008	0	0					
Mm.210787	0	0	3	0.000318674					
Mm.301373	0	0	4	0.000424899					
Mm.250060	0	0	1	0.000106225					
Mm.69	1	0.000502008	1	0.000106225					
Mm.2261	1	0.000502008	2	0.00021245					
Mm.208554	15	0.00753012	15	0.001593372					
Mm.22130	0	0	3	0.000318674					
Mm.246990	0	0	2	0.00021245					
Mm.344671	1	0.000502008	0	0					
Mm.188432	6	0.003012048	12	0.001274697					
Mm.355701	0	0	2	0.00021245					
Mm.229141	0	0	2	0.00021245					
Mm.260512	0	0	1	0.000106225					

Mm.244403	3	0.001506024	0	0
Mm.371682	0	0	5	0.000531124
Mm.289630	1	0.000502008	1	0.000106225
Mm.27539	0	0	1	0.000106225
Mm.35828	0	0	3	0.000318674
Mm.1290	0	0	1	0.000106225
Mm.339640	0	0	2	0.00021245
Mm.82680	0	0	1	0.000106225
Mm.101274	0	0	1	0.000106225
Mm.89845	0	0	1	0.000106225
Mm.247143	0	0	3	0.000318674
Mm.790	0	0	2	0.00021245
Mm.254438	0	0	2	0.00021245
Mm.352946	0	0	7	0.000743573
Mm.8924	1	0.000502008	0	0
Mm.21795	0	0	1	0.000106225
Mm.24551	0	0	4	0.000424899
Mm.221992	0	0	1	0.000106225
Mm.292751	0	0	1	0.000106225
Mm.196604	0	0	3	0.000318674
Mm.36834	0	0	1	0.000106225
Mm.245948	0	0	1	0.000106225
Mm.259876	0	0	2	0.00021245
Mm.227925	3	0.001506024	2	0.00021245
Mm.103262	1	0.000502008	2	0.00021245
Mm.373615	0	0	1	0.000106225
Mm.229444	0	0	1	0.000106225
Mm.358876	0	0	1	0.000106225
Mm.34609	2	0.001004016	2	0.00021245
Mm.20488	0	0	1	0.000106225
Mm.223639	0	0	3	0.000318674
Mm.333597	0	0	4	0.000424899
Mm.231450	0	0	2	0.00021245
Mm.259220	0	0	1	0.000106225
Mm.173068	1	0.000502008	0	0
Mm.275127	0	0	1	0.000106225
Mm.21846	0	0	1	0.000106225
Mm.148643	0	0	1	0.000106225
Mm.358931	0	0	1	0.000106225
Mm.170103	0	0	2	0.00021245
Mm.322502	0	0	1	0.000106225
Mm.24216	0	0	1	0.000106225
Mm.206206	0	0	2	0.00021245
Mm.220885	0	0	2	0.00021245
Mm.1444	0	0	1	0.000106225
Mm.30042	0	0	1	0.000106225
Mm.2454	0	0	1	0.000106225
Mm.2310	0	0	1	0.000106225
Mm.24608	0	0	3	0.000318674
Mm.26652	0	0	2	0.00021245
Mm.25549	1	0.000502008	3	0.000318674
Mm.217323	1	0.000502008	0	0
Mm.348795	1	0.000502008	1	0.000106225
Mm.138792	0	0	5	0.000531124
Mm.333357	0	0	2	0.00021245
Mm.193086	0	0	1	0.000106225

Mm.291754	0	0	1	0.000106225
Mm.154427	0	0	1	0.000106225
Mm.246240	1	0.000502008	2	0.00021245
Mm.327220	1	0.000502008	3	0.000318674
Mm.277533	1	0.000502008	2	0.00021245
Mm.341686	1	0.000502008	0	0
Mm.286149	0	0	6	0.000637349
Mm.214746	0	0	1	0.000106225
Mm.101264	1	0.000502008	0	0
Mm.151640	1	0.000502008	0	0
Mm.25923	2	0.001004016	1	0.000106225
Mm.23636	0	0	2	0.00021245
Mm.21901	1	0.000502008	0	0
Mm.28038	0	0	1	0.000106225
Mm.282851	1	0.000502008	1	0.000106225
Mm.27652	0	0	4	0.000424899
Mm.248906	0	0	1	0.000106225
Mm.11175	0	0	2	0.00021245
Mm.350855	0	0	1	0.000106225
Mm.103199	0	0	1	0.000106225
Mm.288510	0	0	1	0.000106225
Mm.104932	4	0.002008032	2	0.00021245
Mm.305816	0	0	1	0.000106225
Mm.44220	0	0	2	0.00021245
Mm.175612	0	0	1	0.000106225
Mm.260760	0	0	1	0.000106225
Mm.305921	0	0	3	0.000318674
Mm.87352	0	0	1	0.000106225
Mm.27478	0	0	1	0.000106225
Mm.260635	0	0	3	0.000318674
Mm.12239	0	0	4	0.000424899
Mm.374808	1	0.000502008	0	0
Mm.247837	0	0	2	0.00021245
Mm.29122	1	0.000502008	3	0.000318674
Mm.133202	0	0	1	0.000106225
Mm.275608	0	0	1	0.000106225
Mm.157700	0	0	1	0.000106225
Mm.28659	0	0	1	0.000106225
Mm.27792	0	0	2	0.00021245
Mm.245938	0	0	2	0.00021245
Mm.291716	0	0	2	0.00021245
Mm.248637	0	0	2	0.00021245
Mm.277864	1	0.000502008	4	0.000424899
Mm.268018	0	0	2	0.00021245
Mm.296976	0	0	2	0.00021245
Mm.103986	0	0	2	0.00021245
Mm.178818	0	0	2	0.00021245
Mm.290991	1	0.000502008	1	0.000106225
Mm.248755	4	0.002008032	1	0.000106225
Mm.270676	0	0	4	0.000424899
Mm.151315	0	0	1	0.000106225
Mm.29150	0	0	2	0.00021245
Mm.30181	0	0	4	0.000424899
Mm.292107	0	0	1	0.000106225
Mm.272772	0	0	1	0.000106225
Mm.196371	1	0.000502008	0	0

Mm.263913	0	0	3	0.000318674					
Mm.240619	0	0	1	0.000106225					
Mm.220710	2	0.001004016	1	0.000106225					
Mm.25939	0	0	1	0.000106225					
Mm.126534	0	0	1	0.000106225					
Mm.104919	0	0	1	0.000106225					
Mm.316025	0	0	2	0.00021245					
Mm.28251	0	0	4	0.000424899					
Mm.212227	1	0.000502008	2	0.00021245					
Mm.27804	0	0	2	0.00021245					
Mm.332684	0	0	2	0.00021245					
Mm.18889	0	0	2	0.00021245					
Mm.210734	0	0	2	0.00021245					
Mm.261182	2	0.001004016	0	0					
Mm.288898	0	0	4	0.000424899					
Mm.28050	0	0	3	0.000318674					
Mm.18574	0	0	2	0.00021245					
Mm.17665	0	0	1	0.000106225					
Mm.235570	1	0.000502008	0	0					
Mm.28507	0	0	1	0.000106225					
Mm.313185	2	0.001004016	1	0.000106225					
Mm.3774	5	0.00251004	2	0.00021245					
Mm.41540	0	0	3	0.000318674					
Mm.268475	1	0.000502008	0	0					
Mm.288249	0	0	1	0.000106225					
Mm.276650	0	0	2	0.00021245					
Mm.258250	1	0.000502008	1	0.000106225					
Mm.181973	0	0	1	0.000106225					
Mm.274715	0	0	1	0.000106225					
Mm.275121	1	0.000502008	0	0					
Mm.283636	0	0	1	0.000106225					
Mm.325435	0	0	1	0.000106225					
Mm.296049	0	0	4	0.000424899					
Mm.212763	2	0.001004016	0	0					
Mm.17166	0	0	1	0.000106225					
Mm.295565	0	0	1	0.000106225					
Mm.283708	0	0	1	0.000106225					
Mm.11935	2	0.001004016	3	0.000318674					
Mm.21739	0	0	2	0.00021245					
Mm.245522	5	0.00251004	3	0.000318674					
Mm.27932	0	0	1	0.000106225					
Mm.209989	0	0	1	0.000106225					
Mm.306903	0	0	2	0.00021245					
Mm.22314	6	0.003012048	4	0.000424899					
Mm.353975	0	0	2	0.00021245					
Mm.316536	0	0	2	0.00021245					
Mm.246398	0	0	3	0.000318674					
>GO:0043037	31	0.021648045	317	0.04378453	2.022562823	0.000130126	0.006976776	translation	P
Mm.272427	0	0	2	0.00021245					
Mm.29394	0	0	1	0.000106225					
Mm.3941	1	0.000502008	9	0.000956023					
Mm.271674	0	0	3	0.000318674					
Mm.13886	0	0	1	0.000106225					
Mm.28839	3	0.001506024	3	0.000318674					
Mm.29041	0	0	1	0.000106225					
Mm.271222	2	0.001004016	10	0.001062248					

Mm.227183	0	0	1	0.000106225
Mm.196607	0	0	8	0.000849798
Mm.21671	0	0	4	0.000424899
Mm.3955	1	0.000502008	5	0.000531124
Mm.22776	1	0.000502008	4	0.000424899
Mm.294623	0	0	1	0.000106225
Mm.32889	0	0	1	0.000106225
Mm.373568	2	0.001004016	11	0.001168472
Mm.28549	0	0	1	0.000106225
Mm.28753	3	0.001506024	0	0
Mm.29714	0	0	1	0.000106225
Mm.251255	0	0	3	0.000318674
Mm.293628	1	0.000502008	0	0
Mm.185453	2	0.001004016	14	0.001487147
Mm.289992	0	0	4	0.000424899
Mm.261831	1	0.000502008	4	0.000424899
Mm.288669	0	0	3	0.000318674
Mm.289800	1	0.000502008	2	0.00021245
Mm.371545	0	0	7	0.000743573
Mm.5286	0	0	23	0.00244317
Mm.138471	0	0	1	0.000106225
Mm.360075	4	0.002008032	85	0.009029106
Mm.289431	0	0	17	0.001805821
Mm.280768	0	0	1	0.000106225
Mm.219675	0	0	1	0.000106225
Mm.27816	0	0	3	0.000318674
Mm.2718	0	0	11	0.001168472
Mm.3158	0	0	5	0.000531124
Mm.22147	0	0	2	0.00021245
Mm.29900	0	0	2	0.00021245
Mm.371625	0	0	6	0.000637349
Mm.341719	1	0.000502008	7	0.000743573
Mm.274904	0	0	1	0.000106225
Mm.303071	0	0	4	0.000424899
Mm.329353	0	0	2	0.00021245
Mm.21118	0	0	3	0.000318674
Mm.371950	0	0	1	0.000106225
Mm.145488	0	0	3	0.000318674
Mm.154511	3	0.001506024	7	0.000743573
Mm.22363	0	0	1	0.000106225
Mm.286061	0	0	3	0.000318674
Mm.486	0	0	4	0.000424899
Mm.27435	0	0	1	0.000106225
Mm.292517	0	0	2	0.00021245
Mm.70690	0	0	6	0.000637349
Mm.316754	0	0	1	0.000106225
Mm.26379	2	0.001004016	0	0
Mm.196544	0	0	1	0.000106225
Mm.331142	0	0	3	0.000318674
Mm.18845	0	0	1	0.000106225
Mm.273122	1	0.000502008	0	0
Mm.196220	1	0.000502008	0	0
Mm.351579	1	0.000502008	2	0.00021245
Mm.126534	0	0	1	0.000106225
Mm.34701	0	0	1	0.000106225
Mm.341243	0	0	1	0.000106225

>GO:0016773	77	0.05377095	240	0.033149171	0.616488484	0.000197322	0.010419221	phosphotransferase activity F
Mm.273570	0	0	1	0.000106225				
Mm.333471	0	0	1	0.000106225				
Mm.260521	0	0	1	0.000106225				
Mm.71	0	0	1	0.000106225				
Mm.183110	1	0.000502008	0	0				
Mm.248778	1	0.000502008	0	0				
Mm.225505	0	0	3	0.000318674				
Mm.222685	1	0.000502008	0	0				
Mm.277217	1	0.000502008	0	0				
Mm.3810	0	0	2	0.00021245				
Mm.212462	0	0	1	0.000106225				
Mm.276155	1	0.000502008	1	0.000106225				
Mm.245867	1	0.000502008	0	0				
Mm.22682	0	0	2	0.00021245				
Mm.38370	0	0	1	0.000106225				
Mm.259333	3	0.001506024	0	0				
Mm.255044	0	0	1	0.000106225				
Mm.151640	1	0.000502008	0	0				
Mm.25720	1	0.000502008	0	0				
Mm.216227	0	0	1	0.000106225				
Mm.74982	0	0	1	0.000106225				
Mm.255075	0	0	1	0.000106225				
Mm.27557	0	0	2	0.00021245				
Mm.244236	1	0.000502008	0	0				
Mm.184163	1	0.000502008	2	0.00021245				
Mm.254144	0	0	3	0.000318674				
Mm.329515	1	0.000502008	0	0				
Mm.237825	0	0	2	0.00021245				
Mm.39089	0	0	2	0.00021245				
Mm.13806	0	0	2	0.00021245				
Mm.29133	2	0.001004016	3	0.000318674				
Mm.235182	0	0	1	0.000106225				
Mm.327591	0	0	1	0.000106225				
Mm.273049	0	0	2	0.00021245				
Mm.247788	0	0	5	0.000531124				
Mm.281367	0	0	2	0.00021245				
Mm.6839	0	0	3	0.000318674				
Mm.298798	0	0	1	0.000106225				
Mm.16753	4	0.002008032	1	0.000106225				
Mm.3996	0	0	3	0.000318674				
Mm.1761	1	0.000502008	2	0.00021245				
Mm.21974	0	0	1	0.000106225				
Mm.298893	1	0.000502008	0	0				
Mm.51136	0	0	5	0.000531124				
Mm.258986	1	0.000502008	1	0.000106225				
Mm.271976	0	0	2	0.00021245				
Mm.254494	0	0	1	0.000106225				
Mm.23788	0	0	1	0.000106225				
Mm.16340	0	0	2	0.00021245				
Mm.279400	0	0	2	0.00021245				
Mm.20827	0	0	3	0.000318674				
Mm.23790	0	0	1	0.000106225				
Mm.257925	0	0	1	0.000106225				
Mm.275742	2	0.001004016	0	0				
Mm.289657	2	0.001004016	1	0.000106225				

Mm.275839	2	0.001004016	0	0
Mm.209150	1	0.000502008	0	0
Mm.247073	0	0	2	0.00021245
Mm.358618	0	0	6	0.000637349
Mm.124176	0	0	2	0.00021245
Mm.8149	0	0	2	0.00021245
Mm.272206	0	0	2	0.00021245
Mm.28678	0	0	1	0.000106225
Mm.268668	0	0	2	0.00021245
Mm.317339	0	0	2	0.00021245
Mm.116649	0	0	1	0.000106225
Mm.9001	0	0	1	0.000106225
Mm.10504	0	0	1	0.000106225
Mm.291554	1	0.000502008	0	0
Mm.329993	0	0	1	0.000106225
Mm.370198	0	0	1	0.000106225
Mm.311918	0	0	1	0.000106225
Mm.10027	0	0	1	0.000106225
Mm.252210	0	0	1	0.000106225
Mm.6710	4	0.002008032	1	0.000106225
Mm.301827	0	0	1	0.000106225
Mm.28405	0	0	2	0.00021245
Mm.257989	0	0	2	0.00021245
Mm.15252	1	0.000502008	1	0.000106225
Mm.288728	1	0.000502008	0	0
Mm.249363	0	0	5	0.000531124
Mm.257120	1	0.000502008	0	0
Mm.29071	1	0.000502008	1	0.000106225
Mm.172346	1	0.000502008	1	0.000106225
Mm.29515	0	0	2	0.00021245
Mm.271898	0	0	5	0.000531124
Mm.374864	0	0	6	0.000637349
Mm.311948	0	0	1	0.000106225
Mm.286006	1	0.000502008	0	0
Mm.191949	0	0	3	0.000318674
Mm.333349	0	0	1	0.000106225
Mm.235194	0	0	1	0.000106225
Mm.126976	0	0	2	0.00021245
Mm.248907	0	0	2	0.00021245
Mm.172897	0	0	3	0.000318674
Mm.196581	0	0	2	0.00021245
Mm.39253	2	0.001004016	0	0
Mm.27970	0	0	1	0.000106225
Mm.311337	0	0	3	0.000318674
Mm.8385	0	0	3	0.000318674
Mm.21495	3	0.001506024	0	0
Mm.68933	1	0.000502008	0	0
Mm.140948	3	0.001506024	1	0.000106225
Mm.19073	0	0	5	0.000531124
Mm.269211	0	0	1	0.000106225
Mm.202606	0	0	2	0.00021245
Mm.291936	1	0.000502008	1	0.000106225
Mm.18856	0	0	5	0.000531124
Mm.279308	0	0	1	0.000106225
Mm.24337	0	0	2	0.00021245
Mm.34580	0	0	1	0.000106225

Mm.200770	0	0	1	0.000106225					
Mm.234472	0	0	1	0.000106225					
Mm.28761	3	0.001506024	2	0.00021245					
Mm.143817	0	0	1	0.000106225					
Mm.37617	1	0.000502008	0	0					
Mm.253721	1	0.000502008	1	0.000106225					
Mm.31486	3	0.001506024	0	0					
Mm.18539	0	0	1	0.000106225					
Mm.260516	0	0	2	0.00021245					
Mm.193924	0	0	1	0.000106225					
Mm.21876	1	0.000502008	0	0					
Mm.44490	0	0	3	0.000318674					
Mm.227202	0	0	2	0.00021245					
Mm.35290	0	0	1	0.000106225					
Mm.276669	0	0	2	0.00021245					
Mm.262707	0	0	1	0.000106225					
Mm.276063	0	0	1	0.000106225					
Mm.340943	0	0	2	0.00021245					
Mm.26908	0	0	8	0.000849798					
Mm.25559	0	0	1	0.000106225					
Mm.249586	1	0.000502008	0	0					
Mm.4489	1	0.000502008	0	0					
Mm.374793	0	0	1	0.000106225					
Mm.33360	0	0	1	0.000106225					
Mm.7405	1	0.000502008	0	0					
Mm.292040	0	0	3	0.000318674					
Mm.288726	0	0	2	0.00021245					
Mm.292470	6	0.003012048	2	0.00021245					
Mm.162025	0	0	1	0.000106225					
Mm.20593	2	0.001004016	2	0.00021245					
Mm.44963	0	0	2	0.00021245					
Mm.24125	0	0	3	0.000318674					
Mm.101990	1	0.000502008	0	0					
Mm.260802	0	0	1	0.000106225					
Mm.347843	0	0	1	0.000106225					
Mm.200912	0	0	2	0.00021245					
Mm.350712	0	0	1	0.000106225					
Mm.336205	0	0	4	0.000424899					
Mm.260576	0	0	1	0.000106225					
Mm.272221	3	0.001506024	0	0					
Mm.3049	0	0	1	0.000106225					
Mm.222228	0	0	1	0.000106225					
Mm.3401	1	0.000502008	0	0					
Mm.216135	1	0.000502008	0	0					
Mm.196067	0	0	2	0.00021245					
Mm.206159	0	0	2	0.00021245					
Mm.358727	0	0	3	0.000318674					
Mm.7013	0	0	2	0.00021245					
>GO:0015935	5	0.00349162	120	0.016574586	4.746961326	0.000238816	0.012239322	small ribosomal subunit	C
Mm.4071	0	0	11	0.001168472					
Mm.328846	0	0	1	0.000106225					
Mm.643	0	0	7	0.000743573					
Mm.5291	2	0.001004016	17	0.001805821					
Mm.236868	0	0	1	0.000106225					
Mm.295618	0	0	4	0.000424899					
Mm.21938	0	0	4	0.000424899					

Mm.13944	0	0	19	0.002018271					
Mm.193040	0	0	1	0.000106225					
Mm.168680	0	0	4	0.000424899					
Mm.43778	0	0	7	0.000743573					
Mm.371577	0	0	2	0.00021245					
Mm.16775	3	0.001506024	11	0.001168472					
Mm.154915	0	0	2	0.00021245					
Mm.66	0	0	21	0.00223072					
Mm.325584	0	0	3	0.000318674					
Mm.371579	0	0	5	0.000531124					
>GO:0005096	28	0.019553073	61	0.008425414	0.430899763	0.000238678	0.012239322	GTPase activator activity	F
Mm.347359	0	0	3	0.000318674					
Mm.257073	1	0.000502008	0	0					
Mm.291372	0	0	3	0.000318674					
Mm.258939	0	0	1	0.000106225					
Mm.124502	9	0.004518072	0	0					
Mm.35059	0	0	1	0.000106225					
Mm.374780	0	0	1	0.000106225					
Mm.268397	0	0	1	0.000106225					
Mm.267514	1	0.000502008	1	0.000106225					
Mm.153226	0	0	7	0.000743573					
Mm.33779	0	0	1	0.000106225					
Mm.254898	0	0	1	0.000106225					
Mm.30016	0	0	3	0.000318674					
Mm.28262	4	0.002008032	1	0.000106225					
Mm.17009	0	0	4	0.000424899					
Mm.229287	0	0	1	0.000106225					
Mm.227198	0	0	2	0.00021245					
Mm.134338	0	0	2	0.00021245					
Mm.28904	0	0	1	0.000106225					
Mm.22413	0	0	1	0.000106225					
Mm.28646	0	0	1	0.000106225					
Mm.137134	2	0.001004016	0	0					
Mm.286753	0	0	2	0.00021245					
Mm.272230	0	0	1	0.000106225					
Mm.288962	0	0	3	0.000318674					
Mm.44606	0	0	1	0.000106225					
Mm.292180	0	0	1	0.000106225					
Mm.286353	1	0.000502008	0	0					
Mm.22252	10	0.00502008	5	0.000531124					
Mm.156452	0	0	1	0.000106225					
Mm.275266	0	0	3	0.000318674					
Mm.19172	0	0	1	0.000106225					
Mm.193021	0	0	2	0.00021245					
Mm.21646	0	0	1	0.000106225					
Mm.329396	0	0	1	0.000106225					
Mm.356496	0	0	1	0.000106225					
Mm.40673	0	0	1	0.000106225					
Mm.207619	0	0	1	0.000106225					
>GO:0019941	47	0.032821229	127	0.017541436	0.534453979	0.00024768	0.012509642	modification-dependent prot	P
Mm.119717	1	0.000502008	1	0.000106225					
Mm.252255	1	0.000502008	0	0					
Mm.32912	0	0	3	0.000318674					
Mm.368	0	0	3	0.000318674					
Mm.8911	0	0	1	0.000106225					
Mm.98	0	0	1	0.000106225					

Mm.143818	0	0	4	0.000424899					
Mm.324553	0	0	2	0.00021245					
Mm.2847	2	0.001004016	1	0.000106225					
Mm.1485	0	0	3	0.000318674					
Mm.3074	0	0	2	0.00021245					
Mm.240044	2	0.001004016	0	0					
Mm.1104	2	0.001004016	2	0.00021245					
Mm.172835	2	0.001004016	0	0					
Mm.9002	2	0.001004016	2	0.00021245					
Mm.275195	0	0	2	0.00021245					
Mm.29807	6	0.003012048	8	0.000849798					
Mm.3571	0	0	1	0.000106225					
Mm.237594	0	0	1	0.000106225					
Mm.3974	0	0	3	0.000318674					
Mm.242646	2	0.001004016	1	0.000106225					
Mm.80484	0	0	1	0.000106225					
Mm.21630	1	0.000502008	0	0					
Mm.38976	0	0	3	0.000318674					
Mm.305925	4	0.002008032	1	0.000106225					
Mm.290447	0	0	3	0.000318674					
Mm.121265	0	0	6	0.000637349					
Mm.30270	0	0	2	0.00021245					
Mm.208883	0	0	2	0.00021245					
Mm.30210	0	0	6	0.000637349					
Mm.21728	0	0	3	0.000318674					
Mm.22233	0	0	1	0.000106225					
Mm.172605	0	0	2	0.00021245					
Mm.24608	0	0	3	0.000318674					
Mm.251174	0	0	3	0.000318674					
Mm.275970	0	0	1	0.000106225					
Mm.319512	0	0	4	0.000424899					
Mm.272770	0	0	2	0.00021245					
Mm.261004	0	0	1	0.000106225					
Mm.180052	1	0.000502008	9	0.000956023					
Mm.329277	0	0	3	0.000318674					
Mm.288924	2	0.001004016	2	0.00021245					
Mm.256765	3	0.001506024	0	0					
Mm.49884	14	0.007028112	6	0.000637349					
Mm.340315	0	0	4	0.000424899					
Mm.89830	0	0	3	0.000318674					
Mm.13437	2	0.001004016	2	0.00021245					
Mm.16974	0	0	5	0.000531124					
Mm.371667	0	0	3	0.000318674					
Mm.21596	0	0	1	0.000106225					
Mm.296566	0	0	3	0.000318674					
Mm.279070	0	0	1	0.000106225					
>GO:0007127	13	0.009078212	16	0.002209945	0.243433914	0.000319887	0.0159258	meiosis I	P
Mm.288324	0	0	4	0.000424899					
Mm.29760	0	0	5	0.000531124					
Mm.272226	1	0.000502008	0	0					
Mm.273122	1	0.000502008	0	0					
Mm.259893	0	0	3	0.000318674					
Mm.6856	11	0.005522088	4	0.000424899					
>GO:0007067	33	0.023044693	79	0.010911602	0.473497405	0.000333864	0.01638753	mitosis	P
Mm.289747	3	0.001506024	1	0.000106225					
Mm.29133	2	0.001004016	3	0.000318674					

Mm.168523	1	0.000502008	0	0				
Mm.4189	0	0	2	0.00021245				
Mm.260114	0	0	3	0.000318674				
Mm.22592	0	0	1	0.000106225				
Mm.2103	0	0	1	0.000106225				
Mm.307103	2	0.001004016	0	0				
Mm.286602	0	0	1	0.000106225				
Mm.281367	0	0	2	0.00021245				
Mm.12481	0	0	1	0.000106225				
Mm.143877	4	0.002008032	1	0.000106225				
Mm.29755	0	0	2	0.00021245				
Mm.116649	0	0	1	0.000106225				
Mm.56337	2	0.001004016	1	0.000106225				
Mm.182628	0	0	3	0.000318674				
Mm.42135	0	0	3	0.000318674				
Mm.290422	0	0	1	0.000106225				
Mm.332684	0	0	2	0.00021245				
Mm.315959	1	0.000502008	0	0				
Mm.37825	0	0	2	0.00021245				
Mm.29071	1	0.000502008	1	0.000106225				
Mm.26412	5	0.00251004	2	0.00021245				
Mm.24643	0	0	1	0.000106225				
Mm.318364	0	0	1	0.000106225				
Mm.273502	0	0	1	0.000106225				
Mm.172411	0	0	1	0.000106225				
Mm.6856	11	0.005522088	4	0.000424899				
Mm.272568	0	0	3	0.000318674				
Mm.24250	0	0	1	0.000106225				
Mm.24202	0	0	2	0.00021245				
Mm.257590	0	0	1	0.000106225				
Mm.89830	0	0	3	0.000318674				
Mm.257445	0	0	3	0.000318674				
Mm.206841	1	0.000502008	3	0.000318674				
Mm.291624	0	0	2	0.00021245				
Mm.290015	0	0	1	0.000106225				
Mm.328945	0	0	5	0.000531124				
Mm.28659	0	0	1	0.000106225				
Mm.271947	0	0	2	0.00021245				
Mm.273538	0	0	5	0.000531124				
Mm.24105	0	0	1	0.000106225				
Mm.193924	0	0	1	0.000106225				
Mm.273570	0	0	1	0.000106225				
Mm.272024	0	0	1	0.000106225				
Mm.89845	0	0	1	0.000106225				
>GO:0006974	38	0.026536313	97	0.01339779	0.504885141	0.000381065	0.018444619	response to DNA damage s P
Mm.312323	0	0	1	0.000106225				
Mm.277779	0	0	1	0.000106225				
Mm.203	0	0	4	0.000424899				
Mm.236256	1	0.000502008	0	0				
Mm.16753	4	0.002008032	1	0.000106225				
Mm.36524	0	0	2	0.00021245				
Mm.282335	0	0	4	0.000424899				
Mm.288809	0	0	1	0.000106225				
Mm.22700	1	0.000502008	1	0.000106225				
Mm.42201	1	0.000502008	0	0				
Mm.288179	0	0	1	0.000106225				

Mm.277136	0	0	1	0.000106225					
Mm.4619	0	0	4	0.000424899					
Mm.18210	0	0	6	0.000637349					
Mm.35061	0	0	1	0.000106225					
Mm.71	0	0	1	0.000106225					
Mm.182628	0	0	3	0.000318674					
Mm.196846	0	0	1	0.000106225					
Mm.12145	0	0	4	0.000424899					
Mm.185467	0	0	1	0.000106225					
Mm.298456	0	0	2	0.00021245					
Mm.262117	0	0	1	0.000106225					
Mm.172835	2	0.001004016	0	0					
Mm.1393	0	0	1	0.000106225					
Mm.10141	2	0.001004016	4	0.000424899					
Mm.4347	0	0	3	0.000318674					
Mm.246010	0	0	3	0.000318674					
Mm.26412	5	0.00251004	2	0.00021245					
Mm.126976	0	0	2	0.00021245					
Mm.358656	0	0	1	0.000106225					
Mm.6856	11	0.005522088	4	0.000424899					
Mm.287837	0	0	1	0.000106225					
Mm.259294	1	0.000502008	0	0					
Mm.23122	0	0	1	0.000106225					
Mm.23739	0	0	2	0.00021245					
Mm.206921	0	0	2	0.00021245					
Mm.216227	0	0	1	0.000106225					
Mm.281482	0	0	1	0.000106225					
Mm.289915	2	0.001004016	2	0.00021245					
Mm.2952	0	0	3	0.000318674					
Mm.259278	1	0.000502008	2	0.00021245					
Mm.23267	0	0	1	0.000106225					
Mm.272226	1	0.000502008	0	0					
Mm.272989	1	0.000502008	0	0					
Mm.173953	2	0.001004016	2	0.00021245					
Mm.41447	1	0.000502008	0	0					
Mm.323072	0	0	1	0.000106225					
Mm.7141	0	0	2	0.00021245					
Mm.16549	0	0	2	0.00021245					
Mm.2805	1	0.000502008	0	0					
Mm.22117	0	0	3	0.000318674					
Mm.8681	1	0.000502008	2	0.00021245					
Mm.212462	0	0	1	0.000106225					
Mm.311337	0	0	3	0.000318674					
Mm.233734	0	0	1	0.000106225					
Mm.341972	0	0	3	0.000318674					
>GO:0006338	18	0.012569832	32	0.00441989	0.351626765	0.000414121	0.019770037	chromatin remodeling	P
Mm.182836	1	0.000502008	1	0.000106225					
Mm.252213	0	0	1	0.000106225					
Mm.89568	0	0	3	0.000318674					
Mm.12145	0	0	4	0.000424899					
Mm.279751	0	0	3	0.000318674					
Mm.622	0	0	1	0.000106225					
Mm.196508	1	0.000502008	1	0.000106225					
Mm.209385	3	0.001506024	2	0.00021245					
Mm.25339	0	0	1	0.000106225					
Mm.270186	9	0.004518072	8	0.000849798					

Mm.371732	0	0	1	0.000106225					
Mm.41077	1	0.000502008	1	0.000106225					
Mm.291274	0	0	1	0.000106225					
Mm.246803	2	0.001004016	1	0.000106225					
Mm.57223	0	0	1	0.000106225					
Mm.351459	1	0.000502008	2	0.00021245					
>GO:0007346	10	0.00698324	10	0.001381215	0.197790055	0.000545093	0.025670946	regulation of mitotic cell cyc	P
Mm.257482	0	0	3	0.000318674					
Mm.292470	6	0.003012048	2	0.00021245					
Mm.21873	0	0	3	0.000318674					
Mm.104932	4	0.002008032	2	0.00021245					
>GO:0051325	13	0.009078212	19	0.002624309	0.289077773	0.000577702	0.026490697	interphase	P
Mm.22670	1	0.000502008	0	0					
Mm.292470	6	0.003012048	2	0.00021245					
Mm.233734	0	0	1	0.000106225					
Mm.341972	0	0	3	0.000318674					
Mm.925	1	0.000502008	0	0					
Mm.263913	0	0	3	0.000318674					
Mm.235182	0	0	1	0.000106225					
Mm.153415	1	0.000502008	1	0.000106225					
Mm.331389	0	0	2	0.00021245					
Mm.285771	0	0	2	0.00021245					
Mm.44482	0	0	1	0.000106225					
Mm.150686	0	0	1	0.000106225					
Mm.34405	0	0	1	0.000106225					
Mm.16753	4	0.002008032	1	0.000106225					
>GO:0051329	13	0.009078212	19	0.002624309	0.289077773	0.000577702	0.026490697	interphase of mitotic cell cyc	P
Mm.22670	1	0.000502008	0	0					
Mm.292470	6	0.003012048	2	0.00021245					
Mm.235182	0	0	1	0.000106225					
Mm.153415	1	0.000502008	1	0.000106225					
Mm.331389	0	0	2	0.00021245					
Mm.285771	0	0	2	0.00021245					
Mm.44482	0	0	1	0.000106225					
Mm.150686	0	0	1	0.000106225					
Mm.34405	0	0	1	0.000106225					
Mm.925	1	0.000502008	0	0					
Mm.263913	0	0	3	0.000318674					
Mm.233734	0	0	1	0.000106225					
Mm.341972	0	0	3	0.000318674					
Mm.16753	4	0.002008032	1	0.000106225					
>GO:0030004	9	0.006284916	8	0.001104972	0.175813382	0.000609429	0.027228984	monovalent inorganic catior	P
Mm.324393	1	0.000502008	0	0					
Mm.273285	4	0.002008032	2	0.00021245					
Mm.229532	1	0.000502008	0	0					
Mm.17815	0	0	3	0.000318674					
Mm.41044	2	0.001004016	0	0					
Mm.30155	1	0.000502008	3	0.000318674					
>GO:0009309	1	0.000698324	75	0.010359116	14.83425414	0.000606327	0.027228984	amine biosynthesis	P
Mm.289936	0	0	14	0.001487147					
Mm.28301	0	0	2	0.00021245					
Mm.29584	0	0	1	0.000106225					
Mm.23869	0	0	1	0.000106225					
Mm.3217	0	0	3	0.000318674					
Mm.4606	0	0	2	0.00021245					
Mm.206417	0	0	3	0.000318674					

Mm.16898	0	0	3	0.000318674					
Mm.258142	0	0	1	0.000106225					
Mm.196067	0	0	2	0.00021245					
Mm.274180	0	0	1	0.000106225					
Mm.29815	0	0	12	0.001274697					
Mm.24887	0	0	2	0.00021245					
Mm.7286	0	0	1	0.000106225					
Mm.246240	1	0.000502008	2	0.00021245					
Mm.196574	0	0	1	0.000106225					
Mm.34102	0	0	19	0.002018271					
Mm.250214	0	0	2	0.00021245					
Mm.31597	0	0	3	0.000318674					
>GO:0000045	4	0.002793296	0	0	0	0.000740925	0.032276554	autophagic vacuole formatic	P
Mm.22264	2	0.001004016	0	0					
Mm.9852	2	0.001004016	0	0					
>GO:0016236	4	0.002793296	0	0	0	0.000740925	0.032276554	macroautophagy	P
Mm.22264	2	0.001004016	0	0					
Mm.9852	2	0.001004016	0	0					
>GO:0007059	18	0.012569832	34	0.004696133	0.373603438	0.000840709	0.03617126	chromosome segregation	P
Mm.288324	0	0	4	0.000424899					
Mm.182628	0	0	3	0.000318674					
Mm.42135	0	0	3	0.000318674					
Mm.290422	0	0	1	0.000106225					
Mm.4237	0	0	2	0.00021245					
Mm.26412	5	0.00251004	2	0.00021245					
Mm.273502	0	0	1	0.000106225					
Mm.6856	11	0.005522088	4	0.000424899					
Mm.29906	0	0	2	0.00021245					
Mm.206841	1	0.000502008	3	0.000318674					
Mm.290015	0	0	1	0.000106225					
Mm.328945	0	0	5	0.000531124					
Mm.257590	0	0	1	0.000106225					
Mm.161470	1	0.000502008	1	0.000106225					
Mm.151315	0	0	1	0.000106225					
>GO:0017053	6	0.004189944	3	0.000414365	0.098895028	0.00107164	0.044995974	transcriptional repressor cor	C
Mm.5098	3	0.001506024	0	0					
Mm.28840	2	0.001004016	2	0.00021245					
Mm.272826	0	0	1	0.000106225					
Mm.287982	1	0.000502008	0	0					
>GO:0016585	11	0.007681564	14	0.001933702	0.251732798	0.001119413	0.046442297	chromatin remodeling comp	C
Mm.196508	1	0.000502008	1	0.000106225					
Mm.209385	3	0.001506024	2	0.00021245					
Mm.25339	0	0	1	0.000106225					
Mm.41077	1	0.000502008	1	0.000106225					
Mm.272989	1	0.000502008	0	0					
Mm.351459	1	0.000502008	2	0.00021245					
Mm.294625	0	0	5	0.000531124					
Mm.313303	0	0	2	0.00021245					
Mm.22478	4	0.002008032	0	0					
>GO:0019992	8	0.005586592	7	0.000966851	0.173066298	0.001167422	0.047307743	diacylglycerol binding	F
Mm.257073	1	0.000502008	0	0					
Mm.184163	1	0.000502008	2	0.00021245					
Mm.33779	0	0	1	0.000106225					
Mm.291554	1	0.000502008	0	0					
Mm.329993	0	0	1	0.000106225					
Mm.6710	4	0.002008032	1	0.000106225					

Mm.229287	0	0	1	0.000106225					
Mm.248291	1	0.000502008	1	0.000106225					
>GO:0003700	66	0.046089385	212	0.029281768	0.635325632	0.001295205	0.051741238	transcription factor activity	F
Mm.187453	3	0.001506024	0	0					
Mm.12459	0	0	3	0.000318674					
Mm.34554	1	0.000502008	0	0					
Mm.201536	1	0.000502008	3	0.000318674					
Mm.11434	0	0	1	0.000106225					
Mm.252750	0	0	1	0.000106225					
Mm.1399	0	0	1	0.000106225					
Mm.24790	1	0.000502008	1	0.000106225					
Mm.12607	0	0	1	0.000106225					
Mm.324393	1	0.000502008	0	0					
Mm.102136	1	0.000502008	1	0.000106225					
Mm.332919	1	0.000502008	0	0					
Mm.201322	1	0.000502008	0	0					
Mm.17709	0	0	1	0.000106225					
Mm.277668	0	0	2	0.00021245					
Mm.250265	0	0	1	0.000106225					
Mm.12177	1	0.000502008	2	0.00021245					
Mm.676	0	0	2	0.00021245					
Mm.209903	0	0	1	0.000106225					
Mm.291928	1	0.000502008	0	0					
Mm.4509	1	0.000502008	1	0.000106225					
Mm.4909	1	0.000502008	1	0.000106225					
Mm.29914	0	0	1	0.000106225					
Mm.273090	1	0.000502008	0	0					
Mm.290251	3	0.001506024	2	0.00021245					
Mm.153415	1	0.000502008	1	0.000106225					
Mm.181959	0	0	1	0.000106225					
Mm.10724	0	0	3	0.000318674					
Mm.3963	0	0	3	0.000318674					
Mm.14297	0	0	1	0.000106225					
Mm.9213	0	0	5	0.000531124					
Mm.269995	1	0.000502008	0	0					
Mm.213206	0	0	1	0.000106225					
Mm.24069	2	0.001004016	1	0.000106225					
Mm.112824	2	0.001004016	0	0					
Mm.246513	0	0	1	0.000106225					
Mm.293266	1	0.000502008	0	0					
Mm.329287	1	0.000502008	0	0					
Mm.182746	0	0	2	0.00021245					
Mm.374790	0	0	1	0.000106225					
Mm.273292	1	0.000502008	0	0					
Mm.22700	1	0.000502008	1	0.000106225					
Mm.261570	0	0	1	0.000106225					
Mm.19806	0	0	3	0.000318674					
Mm.105218	0	0	1	0.000106225					
Mm.374794	0	0	1	0.000106225					
Mm.223717	0	0	1	0.000106225					
Mm.7320	1	0.000502008	1	0.000106225					
Mm.100399	1	0.000502008	1	0.000106225					
Mm.286488	0	0	2	0.00021245					
Mm.22522	0	0	2	0.00021245					
Mm.132788	0	0	3	0.000318674					
Mm.356578	0	0	1	0.000106225					

Mm.247566	2	0.001004016	4	0.000424899
Mm.373588	0	0	2	0.00021245
Mm.2444	0	0	1	0.000106225
Mm.1025	0	0	1	0.000106225
Mm.31274	1	0.000502008	5	0.000531124
Mm.317947	2	0.001004016	0	0
Mm.256765	3	0.001506024	0	0
Mm.318389	1	0.000502008	0	0
Mm.2380	0	0	2	0.00021245
Mm.259258	0	0	1	0.000106225
Mm.17715	0	0	1	0.000106225
Mm.43358	0	0	4	0.000424899
Mm.7103	0	0	1	0.000106225
Mm.239941	1	0.000502008	0	0
Mm.6923	0	0	1	0.000106225
Mm.292049	0	0	3	0.000318674
Mm.4742	1	0.000502008	4	0.000424899
Mm.17031	0	0	2	0.00021245
Mm.351523	0	0	1	0.000106225
Mm.1243	0	0	1	0.000106225
Mm.311655	1	0.000502008	1	0.000106225
Mm.347499	0	0	1	0.000106225
Mm.4618	1	0.000502008	6	0.000637349
Mm.124328	0	0	1	0.000106225
Mm.21642	0	0	1	0.000106225
Mm.278701	0	0	2	0.00021245
Mm.293120	0	0	1	0.000106225
Mm.249934	0	0	5	0.000531124
Mm.61526	0	0	5	0.000531124
Mm.622	0	0	1	0.000106225
Mm.185467	0	0	1	0.000106225
Mm.249142	1	0.000502008	0	0
Mm.244820	0	0	4	0.000424899
Mm.207263	2	0.001004016	1	0.000106225
Mm.112	0	0	1	0.000106225
Mm.3629	0	0	1	0.000106225
Mm.133919	0	0	1	0.000106225
Mm.628	0	0	2	0.00021245
Mm.2845	0	0	3	0.000318674
Mm.28081	0	0	1	0.000106225
Mm.229292	1	0.000502008	0	0
Mm.925	1	0.000502008	0	0
Mm.153272	3	0.001506024	5	0.000531124
Mm.371688	0	0	1	0.000106225
Mm.5121	0	0	4	0.000424899
Mm.259318	0	0	1	0.000106225
Mm.287100	0	0	1	0.000106225
Mm.253065	0	0	3	0.000318674
Mm.328378	0	0	1	0.000106225
Mm.325086	1	0.000502008	0	0
Mm.22690	0	0	1	0.000106225
Mm.70950	0	0	3	0.000318674
Mm.375273	0	0	1	0.000106225
Mm.3868	0	0	1	0.000106225
Mm.102253	0	0	3	0.000318674
Mm.290596	0	0	2	0.00021245

Mm.24124	0	0	1	0.000106225					
Mm.259227	0	0	1	0.000106225					
Mm.294173	0	0	3	0.000318674					
Mm.257371	0	0	1	0.000106225					
Mm.22501	1	0.000502008	0	0					
Mm.227549	0	0	1	0.000106225					
Mm.290207	1	0.000502008	0	0					
Mm.25339	0	0	1	0.000106225					
Mm.236009	1	0.000502008	0	0					
Mm.285771	0	0	2	0.00021245					
Mm.222222	0	0	1	0.000106225					
Mm.279116	1	0.000502008	0	0					
Mm.26852	0	0	1	0.000106225					
Mm.16794	0	0	2	0.00021245					
Mm.29790	2	0.001004016	0	0					
Mm.26768	0	0	1	0.000106225					
Mm.272414	2	0.001004016	0	0					
Mm.278922	0	0	1	0.000106225					
Mm.183030	0	0	1	0.000106225					
Mm.259326	0	0	1	0.000106225					
Mm.11747	0	0	2	0.00021245					
Mm.340052	0	0	2	0.00021245					
Mm.17640	0	0	1	0.000106225					
Mm.28392	0	0	1	0.000106225					
Mm.3960	0	0	1	0.000106225					
Mm.305674	0	0	1	0.000106225					
Mm.29891	4	0.002008032	2	0.00021245					
Mm.31452	0	0	2	0.00021245					
Mm.87142	1	0.000502008	0	0					
Mm.306482	0	0	1	0.000106225					
Mm.30184	0	0	2	0.00021245					
Mm.15351	0	0	1	0.000106225					
Mm.233734	0	0	1	0.000106225					
Mm.341972	0	0	3	0.000318674					
Mm.9550	0	0	2	0.00021245					
Mm.35290	0	0	1	0.000106225					
Mm.29081	0	0	1	0.000106225					
Mm.152466	0	0	1	0.000106225					
Mm.181009	0	0	1	0.000106225					
Mm.24176	0	0	1	0.000106225					
Mm.30466	1	0.000502008	1	0.000106225					
Mm.190649	0	0	2	0.00021245					
>GO:0008625	13	0.009078212	21	0.002900552	0.319507012	0.001439307	0.056109151	induction of apoptosis via de	P
Mm.25988	13	0.006526104	15	0.001593372					
Mm.34405	0	0	1	0.000106225					
Mm.22279	0	0	5	0.000531124					
>GO:0000910	43	0.030027933	123	0.01698895	0.565771553	0.001449017	0.056109151	cytokinesis	P
Mm.289747	3	0.001506024	1	0.000106225					
Mm.29133	2	0.001004016	3	0.000318674					
Mm.168523	1	0.000502008	0	0					
Mm.4189	0	0	2	0.00021245					
Mm.260114	0	0	3	0.000318674					
Mm.22592	0	0	1	0.000106225					
Mm.273049	0	0	2	0.00021245					
Mm.16110	0	0	1	0.000106225					
Mm.2103	0	0	1	0.000106225					

Mm.250419	0	0	1	0.000106225
Mm.29941	0	0	1	0.000106225
Mm.307103	2	0.001004016	0	0
Mm.286602	0	0	1	0.000106225
Mm.281367	0	0	2	0.00021245
Mm.1022	0	0	10	0.001062248
Mm.6839	0	0	3	0.000318674
Mm.298798	0	0	1	0.000106225
Mm.12481	0	0	1	0.000106225
Mm.143877	4	0.002008032	1	0.000106225
Mm.285723	0	0	1	0.000106225
Mm.29755	0	0	2	0.00021245
Mm.288179	0	0	1	0.000106225
Mm.277136	0	0	1	0.000106225
Mm.116649	0	0	1	0.000106225
Mm.56337	2	0.001004016	1	0.000106225
Mm.241931	0	0	2	0.00021245
Mm.280784	2	0.001004016	7	0.000743573
Mm.182628	0	0	3	0.000318674
Mm.42135	0	0	3	0.000318674
Mm.290422	0	0	1	0.000106225
Mm.332684	0	0	2	0.00021245
Mm.315959	1	0.000502008	0	0
Mm.37825	0	0	2	0.00021245
Mm.89845	0	0	1	0.000106225
Mm.29071	1	0.000502008	1	0.000106225
Mm.23928	2	0.001004016	0	0
Mm.17647	0	0	3	0.000318674
Mm.227274	0	0	2	0.00021245
Mm.270259	0	0	3	0.000318674
Mm.26412	5	0.00251004	2	0.00021245
Mm.24643	0	0	1	0.000106225
Mm.318364	0	0	1	0.000106225
Mm.172411	0	0	1	0.000106225
Mm.6856	11	0.005522088	4	0.000424899
Mm.278584	0	0	1	0.000106225
Mm.272568	0	0	3	0.000318674
Mm.236587	0	0	1	0.000106225
Mm.272221	3	0.001506024	0	0
Mm.3049	0	0	1	0.000106225
Mm.24250	0	0	1	0.000106225
Mm.24202	0	0	2	0.00021245
Mm.175612	0	0	1	0.000106225
Mm.222228	0	0	1	0.000106225
Mm.248637	0	0	2	0.00021245
Mm.270676	0	0	4	0.000424899
Mm.23526	1	0.000502008	1	0.000106225
Mm.86523	0	0	3	0.000318674
Mm.257590	0	0	1	0.000106225
Mm.89830	0	0	3	0.000318674
Mm.282751	1	0.000502008	2	0.00021245
Mm.257445	0	0	3	0.000318674
Mm.260516	0	0	2	0.00021245
Mm.193924	0	0	1	0.000106225
Mm.206841	1	0.000502008	3	0.000318674
Mm.28270	0	0	2	0.00021245

Mm.299254	1	0.000502008	1	0.000106225					
Mm.291624	0	0	2	0.00021245					
Mm.290015	0	0	1	0.000106225					
>GO:0003746	10	0.00698324	140	0.019337017	2.769060773	0.001548776	0.059313032	translation elongation factor	F
Mm.238020	0	0	3	0.000318674					
Mm.138471	0	0	1	0.000106225					
Mm.360075	4	0.002008032	85	0.009029106					
Mm.289431	0	0	17	0.001805821					
Mm.21288	0	0	1	0.000106225					
Mm.194486	3	0.001506024	3	0.000318674					
Mm.207263	2	0.001004016	1	0.000106225					
Mm.280768	0	0	1	0.000106225					
Mm.21105	0	0	1	0.000106225					
Mm.219675	0	0	1	0.000106225					
Mm.27816	0	0	3	0.000318674					
Mm.2718	0	0	11	0.001168472					
Mm.22147	0	0	2	0.00021245					
Mm.29900	0	0	2	0.00021245					
Mm.1116	1	0.000502008	0	0					
Mm.371625	0	0	6	0.000637349					
Mm.274904	0	0	1	0.000106225					
Mm.2152	0	0	1	0.000106225					
>GO:0043296	11	0.007681564	15	0.002071823	0.269713712	0.001653979	0.062653452	apical junction complex	C
Mm.18962	0	0	1	0.000106225					
Mm.158662	0	0	3	0.000318674					
Mm.294882	0	0	1	0.000106225					
Mm.104744	0	0	1	0.000106225					
Mm.235300	3	0.001506024	1	0.000106225					
Mm.281896	0	0	1	0.000106225					
Mm.86421	0	0	1	0.000106225					
Mm.25836	1	0.000502008	0	0					
Mm.370228	0	0	1	0.000106225					
Mm.119714	0	0	1	0.000106225					
Mm.235074	3	0.001506024	0	0					
Mm.299254	1	0.000502008	1	0.000106225					
Mm.355327	3	0.001506024	3	0.000318674					
>GO:0000323	6	0.004189944	108	0.014917127	3.560220994	0.001750965	0.064916093	lytic vacuole	C
Mm.249225	0	0	6	0.000637349					
Mm.45570	2	0.001004016	0	0					
Mm.1114	0	0	2	0.00021245					
Mm.186185	0	0	1	0.000106225					
Mm.290516	0	0	2	0.00021245					
Mm.1894	0	0	1	0.000106225					
Mm.297964	0	0	1	0.000106225					
Mm.371552	0	0	2	0.00021245					
Mm.20837	0	0	1	0.000106225					
Mm.236553	0	0	11	0.001168472					
Mm.322945	0	0	5	0.000531124					
Mm.231395	0	0	15	0.001593372					
Mm.930	0	0	20	0.002124495					
Mm.4793	0	0	5	0.000531124					
Mm.287807	0	0	1	0.000106225					
Mm.16373	0	0	1	0.000106225					
Mm.27816	0	0	3	0.000318674					
Mm.16716	0	0	3	0.000318674					
Mm.486	0	0	4	0.000424899					

Mm.271868	0	0	1	0.000106225					
Mm.4219	0	0	1	0.000106225					
Mm.3484	0	0	1	0.000106225					
Mm.7046	0	0	2	0.00021245					
Mm.17185	1	0.000502008	0	0					
Mm.277498	1	0.000502008	9	0.000956023					
Mm.4628	0	0	8	0.000849798					
Mm.362839	1	0.000502008	0	0					
Mm.156919	0	0	1	0.000106225					
Mm.291443	1	0.000502008	0	0					
Mm.207683	0	0	1	0.000106225					
>GO:0005764	6	0.004189944	108	0.014917127	3.560220994	0.001750965	0.064916093	lysosome	C
Mm.45570	2	0.001004016	0	0					
Mm.1114	0	0	2	0.00021245					
Mm.186185	0	0	1	0.000106225					
Mm.290516	0	0	2	0.00021245					
Mm.1894	0	0	1	0.000106225					
Mm.297964	0	0	1	0.000106225					
Mm.371552	0	0	2	0.00021245					
Mm.20837	0	0	1	0.000106225					
Mm.236553	0	0	11	0.001168472					
Mm.322945	0	0	5	0.000531124					
Mm.231395	0	0	15	0.001593372					
Mm.930	0	0	20	0.002124495					
Mm.4793	0	0	5	0.000531124					
Mm.287807	0	0	1	0.000106225					
Mm.16373	0	0	1	0.000106225					
Mm.27816	0	0	3	0.000318674					
Mm.16716	0	0	3	0.000318674					
Mm.486	0	0	4	0.000424899					
Mm.271868	0	0	1	0.000106225					
Mm.249225	0	0	6	0.000637349					
Mm.4219	0	0	1	0.000106225					
Mm.3484	0	0	1	0.000106225					
Mm.7046	0	0	2	0.00021245					
Mm.17185	1	0.000502008	0	0					
Mm.277498	1	0.000502008	9	0.000956023					
Mm.4628	0	0	8	0.000849798					
Mm.362839	1	0.000502008	0	0					
Mm.156919	0	0	1	0.000106225					
Mm.291443	1	0.000502008	0	0					
Mm.207683	0	0	1	0.000106225					
>GO:0048519	67	0.046787709	219	0.030248619	0.646507793	0.001800858	0.065374883	negative regulation of biolog	P
Mm.297109	0	0	2	0.00021245					
Mm.3193	0	0	3	0.000318674					
Mm.12616	1	0.000502008	1	0.000106225					
Mm.281298	0	0	1	0.000106225					
Mm.34405	0	0	1	0.000106225					
Mm.27764	0	0	3	0.000318674					
Mm.5356	0	0	2	0.00021245					
Mm.256765	3	0.001506024	0	0					
Mm.28278	0	0	2	0.00021245					
Mm.295330	0	0	6	0.000637349					
Mm.332919	1	0.000502008	0	0					
Mm.3596	1	0.000502008	1	0.000106225					
Mm.5098	3	0.001506024	0	0					

Mm.25903	0	0	1	0.000106225
Mm.147946	0	0	2	0.00021245
Mm.272551	2	0.001004016	7	0.000743573
Mm.272361	2	0.001004016	4	0.000424899
Mm.271814	0	0	1	0.000106225
Mm.8256	0	0	1	0.000106225
Mm.103638	0	0	1	0.000106225
Mm.22690	0	0	1	0.000106225
Mm.269211	0	0	1	0.000106225
Mm.271709	0	0	4	0.000424899
Mm.287982	1	0.000502008	0	0
Mm.29142	0	0	2	0.00021245
Mm.28910	0	0	3	0.000318674
Mm.25059	1	0.000502008	8	0.000849798
Mm.4742	1	0.000502008	4	0.000424899
Mm.28840	2	0.001004016	2	0.00021245
Mm.374790	0	0	1	0.000106225
Mm.23790	0	0	1	0.000106225
Mm.7320	1	0.000502008	1	0.000106225
Mm.2537	1	0.000502008	0	0
Mm.168965	0	0	1	0.000106225
Mm.273862	0	0	1	0.000106225
Mm.15755	0	0	2	0.00021245
Mm.22584	0	0	2	0.00021245
Mm.15701	0	0	7	0.000743573
Mm.305561	0	0	3	0.000318674
Mm.39496	1	0.000502008	0	0
Mm.25339	0	0	1	0.000106225
Mm.270186	9	0.004518072	8	0.000849798
Mm.371732	0	0	1	0.000106225
Mm.272826	0	0	1	0.000106225
Mm.173271	0	0	1	0.000106225
Mm.321633	0	0	5	0.000531124
Mm.358714	1	0.000502008	0	0
Mm.318430	0	0	1	0.000106225
Mm.24621	0	0	1	0.000106225
Mm.57223	0	0	1	0.000106225
Mm.351459	1	0.000502008	2	0.00021245
Mm.2171	0	0	1	0.000106225
Mm.12239	0	0	4	0.000424899
Mm.4619	0	0	4	0.000424899
Mm.18210	0	0	6	0.000637349
Mm.21109	0	0	3	0.000318674
Mm.38445	0	0	1	0.000106225
Mm.245297	0	0	1	0.000106225
Mm.238343	0	0	1	0.000106225
Mm.336104	0	0	2	0.00021245
Mm.35691	0	0	1	0.000106225
Mm.272183	0	0	2	0.00021245
Mm.275839	2	0.001004016	0	0
Mm.41417	0	0	1	0.000106225
Mm.30136	0	0	1	0.000106225
Mm.11747	0	0	2	0.00021245
Mm.141021	0	0	1	0.000106225
Mm.313303	0	0	2	0.00021245
Mm.371656	0	0	1	0.000106225

Mm.259879	1	0.000502008	0	0				
Mm.238213	0	0	1	0.000106225				
Mm.3825	0	0	1	0.000106225				
Mm.260521	0	0	1	0.000106225				
Mm.259333	3	0.001506024	0	0				
Mm.84073	0	0	2	0.00021245				
Mm.27582	1	0.000502008	0	0				
Mm.2026	0	0	4	0.000424899				
Mm.8552	0	0	4	0.000424899				
Mm.181824	0	0	2	0.00021245				
Mm.257460	0	0	1	0.000106225				
Mm.25988	13	0.006526104	15	0.001593372				
Mm.290908	0	0	1	0.000106225				
Mm.319038	0	0	2	0.00021245				
Mm.268521	0	0	1	0.000106225				
Mm.28235	0	0	3	0.000318674				
Mm.347009	3	0.001506024	7	0.000743573				
Mm.296922	0	0	6	0.000637349				
Mm.286006	1	0.000502008	0	0				
Mm.29780	0	0	4	0.000424899				
Mm.279437	0	0	3	0.000318674				
Mm.200770	0	0	1	0.000106225				
Mm.272427	0	0	2	0.00021245				
Mm.2444	0	0	1	0.000106225				
Mm.28835	0	0	1	0.000106225				
Mm.23978	0	0	2	0.00021245				
Mm.359653	1	0.000502008	3	0.000318674				
Mm.217318	1	0.000502008	3	0.000318674				
Mm.222272	1	0.000502008	1	0.000106225				
Mm.45372	0	0	1	0.000106225				
Mm.273270	1	0.000502008	0	0				
Mm.23684	0	0	1	0.000106225				
Mm.55143	1	0.000502008	0	0				
Mm.159724	0	0	1	0.000106225				
Mm.4913	1	0.000502008	0	0				
Mm.28969	0	0	1	0.000106225				
Mm.290924	1	0.000502008	0	0				
Mm.246688	3	0.001506024	2	0.00021245				
Mm.17185	1	0.000502008	0	0				
Mm.228798	0	0	1	0.000106225				
Mm.292107	0	0	1	0.000106225				
>GO:0051276	53	0.037011173	163	0.022513812	0.608297717	0.00178602	0.065374883	chromosome organization a P
Mm.26412	5	0.00251004	2	0.00021245				
Mm.206841	1	0.000502008	3	0.000318674				
Mm.290563	0	0	3	0.000318674				
Mm.24350	0	0	1	0.000106225				
Mm.352429	1	0.000502008	0	0				
Mm.371563	5	0.00251004	11	0.001168472				
Mm.182628	0	0	3	0.000318674				
Mm.182776	0	0	1	0.000106225				
Mm.133101	1	0.000502008	1	0.000106225				
Mm.283802	0	0	1	0.000106225				
Mm.117541	0	0	5	0.000531124				
Mm.277779	0	0	1	0.000106225				
Mm.71	0	0	1	0.000106225				
Mm.11333	0	0	1	0.000106225				

Mm.280717	1	0.000502008	0	0
Mm.288460	0	0	2	0.00021245
Mm.150231	0	0	1	0.000106225
Mm.245739	0	0	1	0.000106225
Mm.288015	2	0.001004016	0	0
Mm.311655	1	0.000502008	1	0.000106225
Mm.313303	0	0	2	0.00021245
Mm.22478	4	0.002008032	0	0
Mm.333388	0	0	1	0.000106225
Mm.29055	0	0	11	0.001168472
Mm.262059	0	0	1	0.000106225
Mm.8137	0	0	2	0.00021245
Mm.24248	0	0	1	0.000106225
Mm.9244	0	0	1	0.000106225
Mm.128273	2	0.001004016	0	0
Mm.195770	0	0	1	0.000106225
Mm.152466	0	0	1	0.000106225
Mm.246803	2	0.001004016	1	0.000106225
Mm.21899	0	0	5	0.000531124
Mm.16711	0	0	6	0.000637349
Mm.294625	0	0	5	0.000531124
Mm.290407	1	0.000502008	3	0.000318674
Mm.337558	0	0	3	0.000318674
Mm.335942	0	0	5	0.000531124
Mm.272989	1	0.000502008	0	0
Mm.29680	1	0.000502008	1	0.000106225
Mm.273570	0	0	1	0.000106225
Mm.35345	0	0	1	0.000106225
Mm.289584	2	0.001004016	3	0.000318674
Mm.19806	0	0	3	0.000318674
Mm.344671	1	0.000502008	0	0
Mm.280768	0	0	1	0.000106225
Mm.196508	1	0.000502008	1	0.000106225
Mm.209385	3	0.001506024	2	0.00021245
Mm.332268	0	0	1	0.000106225
Mm.246010	0	0	3	0.000318674
Mm.126976	0	0	2	0.00021245
Mm.348392	0	0	2	0.00021245
Mm.283410	1	0.000502008	8	0.000849798
Mm.41077	1	0.000502008	1	0.000106225
Mm.29142	0	0	2	0.00021245
Mm.270487	1	0.000502008	0	0
Mm.24176	0	0	1	0.000106225
Mm.181661	1	0.000502008	0	0
Mm.182836	1	0.000502008	1	0.000106225
Mm.252213	0	0	1	0.000106225
Mm.89568	0	0	3	0.000318674
Mm.12145	0	0	4	0.000424899
Mm.279751	0	0	3	0.000318674
Mm.622	0	0	1	0.000106225
Mm.25339	0	0	1	0.000106225
Mm.270186	9	0.004518072	8	0.000849798
Mm.371732	0	0	1	0.000106225
Mm.291274	0	0	1	0.000106225
Mm.57223	0	0	1	0.000106225
Mm.351459	1	0.000502008	2	0.00021245

Mm.246688	3	0.001506024	2	0.00021245					
Mm.278578	0	0	1	0.000106225					
Mm.247844	0	0	2	0.00021245					
Mm.248967	0	0	1	0.000106225					
Mm.4237	0	0	2	0.00021245					
Mm.257590	0	0	1	0.000106225					
Mm.290015	0	0	1	0.000106225					
Mm.328945	0	0	5	0.000531124					
>GO:0006281	32	0.022346369	84	0.01160221	0.519198895	0.001884289	0.067698441	DNA repair	P
Mm.206921	0	0	2	0.00021245					
Mm.216227	0	0	1	0.000106225					
Mm.312323	0	0	1	0.000106225					
Mm.277779	0	0	1	0.000106225					
Mm.281482	0	0	1	0.000106225					
Mm.203	0	0	4	0.000424899					
Mm.236256	1	0.000502008	0	0					
Mm.289915	2	0.001004016	2	0.00021245					
Mm.36524	0	0	2	0.00021245					
Mm.282335	0	0	4	0.000424899					
Mm.2952	0	0	3	0.000318674					
Mm.288809	0	0	1	0.000106225					
Mm.22700	1	0.000502008	1	0.000106225					
Mm.288179	0	0	1	0.000106225					
Mm.277136	0	0	1	0.000106225					
Mm.4619	0	0	4	0.000424899					
Mm.18210	0	0	6	0.000637349					
Mm.35061	0	0	1	0.000106225					
Mm.71	0	0	1	0.000106225					
Mm.182628	0	0	3	0.000318674					
Mm.196846	0	0	1	0.000106225					
Mm.12145	0	0	4	0.000424899					
Mm.185467	0	0	1	0.000106225					
Mm.298456	0	0	2	0.00021245					
Mm.262117	0	0	1	0.000106225					
Mm.172835	2	0.001004016	0	0					
Mm.259278	1	0.000502008	2	0.00021245					
Mm.1393	0	0	1	0.000106225					
Mm.10141	2	0.001004016	4	0.000424899					
Mm.4347	0	0	3	0.000318674					
Mm.23267	0	0	1	0.000106225					
Mm.246010	0	0	3	0.000318674					
Mm.26412	5	0.00251004	2	0.00021245					
Mm.358656	0	0	1	0.000106225					
Mm.6856	11	0.005522088	4	0.000424899					
Mm.287837	0	0	1	0.000106225					
Mm.272226	1	0.000502008	0	0					
Mm.259294	1	0.000502008	0	0					
Mm.23122	0	0	1	0.000106225					
Mm.272989	1	0.000502008	0	0					
Mm.173953	2	0.001004016	2	0.00021245					
Mm.23739	0	0	2	0.00021245					
Mm.41447	1	0.000502008	0	0					
Mm.323072	0	0	1	0.000106225					
Mm.7141	0	0	2	0.00021245					
Mm.16549	0	0	2	0.00021245					
Mm.2805	1	0.000502008	0	0					

Mm.22117	0	0	3	0.000318674					
>GO:0006883	5	0.00349162	2	0.000276243	0.079116022	0.001909519	0.067904837	sodium ion homeostasis	P
Mm.324393	1	0.000502008	0	0					
Mm.273285	4	0.002008032	2	0.00021245					
>GO:0051301	45	0.031424581	133	0.018370166	0.584579497	0.002060176	0.072522369	cell division	P
Mm.289747	3	0.001506024	1	0.000106225					
Mm.29133	2	0.001004016	3	0.000318674					
Mm.168523	1	0.000502008	0	0					
Mm.4189	0	0	2	0.00021245					
Mm.260114	0	0	3	0.000318674					
Mm.22592	0	0	1	0.000106225					
Mm.273049	0	0	2	0.00021245					
Mm.16110	0	0	1	0.000106225					
Mm.2103	0	0	1	0.000106225					
Mm.250419	0	0	1	0.000106225					
Mm.29941	0	0	1	0.000106225					
Mm.307103	2	0.001004016	0	0					
Mm.286602	0	0	1	0.000106225					
Mm.281367	0	0	2	0.00021245					
Mm.1022	0	0	10	0.001062248					
Mm.6839	0	0	3	0.000318674					
Mm.298798	0	0	1	0.000106225					
Mm.12481	0	0	1	0.000106225					
Mm.143877	4	0.002008032	1	0.000106225					
Mm.285723	0	0	1	0.000106225					
Mm.29755	0	0	2	0.00021245					
Mm.288179	0	0	1	0.000106225					
Mm.277136	0	0	1	0.000106225					
Mm.116649	0	0	1	0.000106225					
Mm.56337	2	0.001004016	1	0.000106225					
Mm.241931	0	0	2	0.00021245					
Mm.280784	2	0.001004016	7	0.000743573					
Mm.182628	0	0	3	0.000318674					
Mm.42135	0	0	3	0.000318674					
Mm.290422	0	0	1	0.000106225					
Mm.332684	0	0	2	0.00021245					
Mm.315959	1	0.000502008	0	0					
Mm.37825	0	0	2	0.00021245					
Mm.89845	0	0	1	0.000106225					
Mm.29071	1	0.000502008	1	0.000106225					
Mm.23928	2	0.001004016	0	0					
Mm.17647	0	0	3	0.000318674					
Mm.227274	0	0	2	0.00021245					
Mm.270259	0	0	3	0.000318674					
Mm.26412	5	0.00251004	2	0.00021245					
Mm.24643	0	0	1	0.000106225					
Mm.318364	0	0	1	0.000106225					
Mm.172411	0	0	1	0.000106225					
Mm.6856	11	0.005522088	4	0.000424899					
Mm.278584	0	0	1	0.000106225					
Mm.272568	0	0	3	0.000318674					
Mm.236587	0	0	1	0.000106225					
Mm.272221	3	0.001506024	0	0					
Mm.3049	0	0	1	0.000106225					
Mm.24250	0	0	1	0.000106225					
Mm.24202	0	0	2	0.00021245					

Mm.175612	0	0	1	0.000106225						
Mm.222228	0	0	1	0.000106225						
Mm.248637	0	0	2	0.00021245						
Mm.270676	0	0	4	0.000424899						
Mm.23526	1	0.000502008	1	0.000106225						
Mm.86523	0	0	3	0.000318674						
Mm.257590	0	0	1	0.000106225						
Mm.89830	0	0	3	0.000318674						
Mm.282751	1	0.000502008	2	0.00021245						
Mm.257445	0	0	3	0.000318674						
Mm.260516	0	0	2	0.00021245						
Mm.193924	0	0	1	0.000106225						
Mm.206841	1	0.000502008	3	0.000318674						
Mm.28270	0	0	2	0.00021245						
Mm.299254	1	0.000502008	1	0.000106225						
Mm.291624	0	0	2	0.00021245						
Mm.290015	0	0	1	0.000106225						
Mm.289584	2	0.001004016	3	0.000318674						
Mm.4237	0	0	2	0.00021245						
Mm.257482	0	0	3	0.000318674						
Mm.27982	0	0	2	0.00021245						
>GO:0030323	6	0.004189944	4	0.000552486	0.131860037	0.002305113	0.075786033	respiratory tube developmen	P
Mm.291928	1	0.000502008	0	0						
Mm.249342	0	0	2	0.00021245						
Mm.241282	0	0	2	0.00021245						
Mm.5098	3	0.001506024	0	0						
Mm.317947	2	0.001004016	0	0						
>GO:0004672	66	0.046089385	217	0.029972376	0.650309727	0.002250236	0.075786033	protein kinase activity	F
Mm.25720	1	0.000502008	0	0						
Mm.216227	0	0	1	0.000106225						
Mm.74982	0	0	1	0.000106225						
Mm.255075	0	0	1	0.000106225						
Mm.27557	0	0	2	0.00021245						
Mm.244236	1	0.000502008	0	0						
Mm.184163	1	0.000502008	2	0.00021245						
Mm.254144	0	0	3	0.000318674						
Mm.329515	1	0.000502008	0	0						
Mm.237825	0	0	2	0.00021245						
Mm.39089	0	0	2	0.00021245						
Mm.13806	0	0	2	0.00021245						
Mm.29133	2	0.001004016	3	0.000318674						
Mm.235182	0	0	1	0.000106225						
Mm.327591	0	0	1	0.000106225						
Mm.273049	0	0	2	0.00021245						
Mm.247788	0	0	5	0.000531124						
Mm.281367	0	0	2	0.00021245						
Mm.6839	0	0	3	0.000318674						
Mm.298798	0	0	1	0.000106225						
Mm.16753	4	0.002008032	1	0.000106225						
Mm.3996	0	0	3	0.000318674						
Mm.1761	1	0.000502008	2	0.00021245						
Mm.21974	0	0	1	0.000106225						
Mm.298893	1	0.000502008	0	0						
Mm.51136	0	0	5	0.000531124						
Mm.258986	1	0.000502008	1	0.000106225						
Mm.271976	0	0	2	0.00021245						

Mm.254494	0	0	1	0.000106225
Mm.23788	0	0	1	0.000106225
Mm.16340	0	0	2	0.00021245
Mm.279400	0	0	2	0.00021245
Mm.20827	0	0	3	0.000318674
Mm.23790	0	0	1	0.000106225
Mm.257925	0	0	1	0.000106225
Mm.275742	2	0.001004016	0	0
Mm.289657	2	0.001004016	1	0.000106225
Mm.275839	2	0.001004016	0	0
Mm.209150	1	0.000502008	0	0
Mm.247073	0	0	2	0.00021245
Mm.358618	0	0	6	0.000637349
Mm.124176	0	0	2	0.00021245
Mm.8149	0	0	2	0.00021245
Mm.272206	0	0	2	0.00021245
Mm.28678	0	0	1	0.000106225
Mm.268668	0	0	2	0.00021245
Mm.317339	0	0	2	0.00021245
Mm.116649	0	0	1	0.000106225
Mm.9001	0	0	1	0.000106225
Mm.10504	0	0	1	0.000106225
Mm.291554	1	0.000502008	0	0
Mm.329993	0	0	1	0.000106225
Mm.370198	0	0	1	0.000106225
Mm.311918	0	0	1	0.000106225
Mm.10027	0	0	1	0.000106225
Mm.252210	0	0	1	0.000106225
Mm.6710	4	0.002008032	1	0.000106225
Mm.301827	0	0	1	0.000106225
Mm.28405	0	0	2	0.00021245
Mm.257989	0	0	2	0.00021245
Mm.15252	1	0.000502008	1	0.000106225
Mm.288728	1	0.000502008	0	0
Mm.249363	0	0	5	0.000531124
Mm.257120	1	0.000502008	0	0
Mm.29071	1	0.000502008	1	0.000106225
Mm.172346	1	0.000502008	1	0.000106225
Mm.29515	0	0	2	0.00021245
Mm.271898	0	0	5	0.000531124
Mm.374864	0	0	6	0.000637349
Mm.311948	0	0	1	0.000106225
Mm.286006	1	0.000502008	0	0
Mm.191949	0	0	3	0.000318674
Mm.333349	0	0	1	0.000106225
Mm.235194	0	0	1	0.000106225
Mm.126976	0	0	2	0.00021245
Mm.248907	0	0	2	0.00021245
Mm.172897	0	0	3	0.000318674
Mm.196581	0	0	2	0.00021245
Mm.39253	2	0.001004016	0	0
Mm.27970	0	0	1	0.000106225
Mm.311337	0	0	3	0.000318674
Mm.8385	0	0	3	0.000318674
Mm.21495	3	0.001506024	0	0
Mm.68933	1	0.000502008	0	0

Mm.140948	3	0.001506024	1	0.000106225					
Mm.19073	0	0	5	0.000531124					
Mm.269211	0	0	1	0.000106225					
Mm.202606	0	0	2	0.00021245					
Mm.291936	1	0.000502008	1	0.000106225					
Mm.18856	0	0	5	0.000531124					
Mm.279308	0	0	1	0.000106225					
Mm.24337	0	0	2	0.00021245					
Mm.34580	0	0	1	0.000106225					
Mm.200770	0	0	1	0.000106225					
Mm.234472	0	0	1	0.000106225					
Mm.28761	3	0.001506024	2	0.00021245					
Mm.143817	0	0	1	0.000106225					
Mm.37617	1	0.000502008	0	0					
Mm.253721	1	0.000502008	1	0.000106225					
Mm.31486	3	0.001506024	0	0					
Mm.18539	0	0	1	0.000106225					
Mm.260516	0	0	2	0.00021245					
Mm.193924	0	0	1	0.000106225					
Mm.21876	1	0.000502008	0	0					
Mm.44490	0	0	3	0.000318674					
Mm.227202	0	0	2	0.00021245					
Mm.35290	0	0	1	0.000106225					
Mm.276669	0	0	2	0.00021245					
Mm.262707	0	0	1	0.000106225					
Mm.276063	0	0	1	0.000106225					
Mm.340943	0	0	2	0.00021245					
Mm.26908	0	0	8	0.000849798					
Mm.25559	0	0	1	0.000106225					
Mm.249586	1	0.000502008	0	0					
Mm.4489	1	0.000502008	0	0					
Mm.374793	0	0	1	0.000106225					
Mm.33360	0	0	1	0.000106225					
Mm.7405	1	0.000502008	0	0					
Mm.71	0	0	1	0.000106225					
Mm.292040	0	0	3	0.000318674					
Mm.288726	0	0	2	0.00021245					
Mm.292470	6	0.003012048	2	0.00021245					
Mm.162025	0	0	1	0.000106225					
Mm.20593	2	0.001004016	2	0.00021245					
Mm.44963	0	0	2	0.00021245					
Mm.24125	0	0	3	0.000318674					
Mm.101990	1	0.000502008	0	0					
Mm.260802	0	0	1	0.000106225					
Mm.347843	0	0	1	0.000106225					
Mm.200912	0	0	2	0.00021245					
Mm.350712	0	0	1	0.000106225					
Mm.336205	0	0	4	0.000424899					
Mm.260576	0	0	1	0.000106225					
Mm.272221	3	0.001506024	0	0					
Mm.3049	0	0	1	0.000106225					
Mm.222228	0	0	1	0.000106225					
Mm.3401	1	0.000502008	0	0					
>GO:0008286	6	0.004189944	4	0.000552486	0.131860037	0.002305113	0.075786033	insulin receptor signaling pa	P
Mm.224246	0	0	2	0.00021245					
Mm.29891	4	0.002008032	2	0.00021245					

Mm.221688	2	0.001004016	0	0					
>GO:0030324	6	0.004189944	4	0.000552486	0.131860037	0.002305113	0.075786033	lung development	P
Mm.291928	1	0.000502008	0	0					
Mm.249342	0	0	2	0.00021245					
Mm.241282	0	0	2	0.00021245					
Mm.5098	3	0.001506024	0	0					
Mm.317947	2	0.001004016	0	0					
>GO:0004459	7	0.004888268	6	0.000828729	0.169534333	0.002249226	0.075786033	L-lactate dehydrogenase ac	F
Mm.29324	0	0	1	0.000106225					
Mm.9745	7	0.003514056	5	0.000531124					
>GO:0004457	7	0.004888268	6	0.000828729	0.169534333	0.002249226	0.075786033	lactate dehydrogenase activ	F
Mm.29324	0	0	1	0.000106225					
Mm.9745	7	0.003514056	5	0.000531124					
>GO:0031090	19	0.013268156	198	0.027348066	2.061180576	0.002492481	0.081180336	organelle membrane	C
Mm.2433	0	0	1	0.000106225					
Mm.209207	0	0	2	0.00021245					
Mm.9392	0	0	1	0.000106225					
Mm.17	0	0	1	0.000106225					
Mm.155696	0	0	2	0.00021245					
Mm.153061	0	0	2	0.00021245					
Mm.196269	0	0	4	0.000424899					
Mm.330070	0	0	1	0.000106225					
Mm.273768	0	0	4	0.000424899					
Mm.371666	0	0	1	0.000106225					
Mm.316536	0	0	2	0.00021245					
Mm.241604	0	0	2	0.00021245					
Mm.279713	0	0	1	0.000106225					
Mm.18892	1	0.000502008	0	0					
Mm.4538	0	0	1	0.000106225					
Mm.271674	0	0	3	0.000318674					
Mm.243014	0	0	2	0.00021245					
Mm.9901	0	0	2	0.00021245					
Mm.4113	1	0.000502008	0	0					
Mm.267006	0	0	1	0.000106225					
Mm.2159	1	0.000502008	1	0.000106225					
Mm.29820	0	0	2	0.00021245					
Mm.180182	0	0	1	0.000106225					
Mm.43415	0	0	3	0.000318674					
Mm.2731	0	0	2	0.00021245					
Mm.271775	1	0.000502008	1	0.000106225					
Mm.278560	0	0	1	0.000106225					
Mm.28663	0	0	2	0.00021245					
Mm.280784	2	0.001004016	7	0.000743573					
Mm.3555	0	0	1	0.000106225					
Mm.262327	0	0	9	0.000956023					
Mm.227704	0	0	1	0.000106225					
Mm.279603	0	0	1	0.000106225					
Mm.240434	0	0	1	0.000106225					
Mm.284503	0	0	1	0.000106225					
Mm.16228	0	0	3	0.000318674					
Mm.371544	0	0	2	0.00021245					
Mm.2136	0	0	10	0.001062248					
Mm.42012	0	0	1	0.000106225					
Mm.228067	0	0	2	0.00021245					
Mm.2368	0	0	9	0.000956023					
Mm.27393	0	0	2	0.00021245					

Mm.171378	0	0	3	0.000318674					
Mm.247093	1	0.000502008	1	0.000106225					
Mm.274243	0	0	1	0.000106225					
Mm.18803	0	0	2	0.00021245					
Mm.285322	0	0	1	0.000106225					
Mm.168680	0	0	4	0.000424899					
Mm.269881	0	0	1	0.000106225					
Mm.28336	0	0	1	0.000106225					
Mm.296082	0	0	1	0.000106225					
Mm.334031	0	0	1	0.000106225					
Mm.17875	0	0	1	0.000106225					
Mm.195249	2	0.001004016	0	0					
Mm.5381	0	0	2	0.00021245					
Mm.335460	0	0	3	0.000318674					
Mm.197728	0	0	3	0.000318674					
Mm.29196	0	0	1	0.000106225					
Mm.334206	2	0.001004016	1	0.000106225					
Mm.24805	0	0	3	0.000318674					
Mm.29842	0	0	2	0.00021245					
Mm.290791	0	0	1	0.000106225					
Mm.340095	0	0	1	0.000106225					
Mm.400	0	0	1	0.000106225					
Mm.548	0	0	4	0.000424899					
Mm.43786	0	0	2	0.00021245					
Mm.14022	0	0	1	0.000106225					
Mm.276137	1	0.000502008	9	0.000956023					
Mm.12677	0	0	2	0.00021245					
Mm.217787	1	0.000502008	2	0.00021245					
Mm.29045	0	0	1	0.000106225					
Mm.30155	1	0.000502008	3	0.000318674					
Mm.214504	0	0	1	0.000106225					
Mm.286702	0	0	2	0.00021245					
Mm.7236	0	0	3	0.000318674					
Mm.18353	0	0	1	0.000106225					
Mm.277327	0	0	1	0.000106225					
Mm.288741	0	0	1	0.000106225					
Mm.340943	0	0	2	0.00021245					
Mm.14816	2	0.001004016	1	0.000106225					
Mm.28852	0	0	1	0.000106225					
Mm.319038	0	0	2	0.00021245					
Mm.29924	1	0.000502008	7	0.000743573					
Mm.284688	0	0	2	0.00021245					
Mm.22362	0	0	3	0.000318674					
Mm.314113	0	0	2	0.00021245					
Mm.21596	0	0	1	0.000106225					
Mm.13705	0	0	2	0.00021245					
Mm.16373	0	0	1	0.000106225					
Mm.271898	0	0	5	0.000531124					
Mm.486	0	0	4	0.000424899					
Mm.3337	1	0.000502008	0	0					
Mm.201455	1	0.000502008	1	0.000106225					
>GO:0007420	9	0.006284916	11	0.001519337	0.241743401	0.002628211	0.084808465	brain development	P
Mm.258939	0	0	1	0.000106225					
Mm.289584	2	0.001004016	3	0.000318674					
Mm.5098	3	0.001506024	0	0					
Mm.275742	2	0.001004016	0	0					

Mm.3057	1	0.000502008	0	0					
Mm.22699	0	0	2	0.00021245					
Mm.24430	0	0	2	0.00021245					
Mm.19133	0	0	1	0.000106225					
Mm.14297	0	0	1	0.000106225					
Mm.24105	0	0	1	0.000106225					
Mm.273292	1	0.000502008	0	0					
>GO:0001707	4	0.002793296	1	0.000138122	0.049447514	0.003216374	0.084917157	mesoderm formation	P
Mm.7320	1	0.000502008	1	0.000106225					
Mm.30039	1	0.000502008	0	0					
Mm.29790	2	0.001004016	0	0					
>GO:0008135	32	0.022346369	282	0.038950276	1.743024862	0.002737093	0.084917157	translation factor activity, nu	F
Mm.262037	0	0	1	0.000106225					
Mm.185467	0	0	1	0.000106225					
Mm.52275	1	0.000502008	0	0					
Mm.294623	0	0	1	0.000106225					
Mm.289800	1	0.000502008	2	0.00021245					
Mm.196220	1	0.000502008	0	0					
Mm.29394	0	0	1	0.000106225					
Mm.2238	0	0	2	0.00021245					
Mm.371557	0	0	10	0.001062248					
Mm.260084	0	0	5	0.000531124					
Mm.3941	1	0.000502008	9	0.000956023					
Mm.185453	2	0.001004016	14	0.001487147					
Mm.289992	0	0	4	0.000424899					
Mm.271674	0	0	3	0.000318674					
Mm.197555	1	0.000502008	2	0.00021245					
Mm.260256	0	0	1	0.000106225					
Mm.13886	0	0	1	0.000106225					
Mm.28839	3	0.001506024	3	0.000318674					
Mm.29041	0	0	1	0.000106225					
Mm.271222	2	0.001004016	10	0.001062248					
Mm.259294	1	0.000502008	0	0					
Mm.260943	0	0	3	0.000318674					
Mm.21617	0	0	2	0.00021245					
Mm.274482	0	0	1	0.000106225					
Mm.218851	0	0	5	0.000531124					
Mm.250909	0	0	1	0.000106225					
Mm.227183	0	0	1	0.000106225					
Mm.196607	0	0	8	0.000849798					
Mm.21671	0	0	4	0.000424899					
Mm.260064	0	0	7	0.000743573					
Mm.250874	0	0	2	0.00021245					
Mm.3955	1	0.000502008	5	0.000531124					
Mm.22776	1	0.000502008	4	0.000424899					
Mm.334467	1	0.000502008	0	0					
Mm.87046	0	0	1	0.000106225					
Mm.261831	1	0.000502008	4	0.000424899					
Mm.32889	0	0	1	0.000106225					
Mm.288669	0	0	3	0.000318674					
Mm.373568	2	0.001004016	11	0.001168472					
Mm.28549	0	0	1	0.000106225					
Mm.267998	0	0	1	0.000106225					
Mm.28753	3	0.001506024	0	0					
Mm.29714	0	0	1	0.000106225					
Mm.255649	0	0	1	0.000106225					

Mm.27695	0	0	1	0.000106225					
Mm.238020	0	0	3	0.000318674					
Mm.138471	0	0	1	0.000106225					
Mm.360075	4	0.002008032	85	0.009029106					
Mm.289431	0	0	17	0.001805821					
Mm.21288	0	0	1	0.000106225					
Mm.194486	3	0.001506024	3	0.000318674					
Mm.207263	2	0.001004016	1	0.000106225					
Mm.280768	0	0	1	0.000106225					
Mm.21105	0	0	1	0.000106225					
Mm.219675	0	0	1	0.000106225					
Mm.27816	0	0	3	0.000318674					
Mm.2718	0	0	11	0.001168472					
Mm.22147	0	0	2	0.00021245					
Mm.29900	0	0	2	0.00021245					
Mm.1116	1	0.000502008	0	0					
Mm.371625	0	0	6	0.000637349					
Mm.274904	0	0	1	0.000106225					
Mm.2152	0	0	1	0.000106225					
Mm.329353	0	0	2	0.00021245					
Mm.274146	0	0	1	0.000106225					
>GO:0007417	10	0.00698324	14	0.001933702	0.276906077	0.003069791	0.084917157	central nervous system dev	P
Mm.3451	1	0.000502008	0	0					
Mm.5098	3	0.001506024	0	0					
Mm.245395	0	0	1	0.000106225					
Mm.3057	1	0.000502008	0	0					
Mm.29279	0	0	2	0.00021245					
Mm.258939	0	0	1	0.000106225					
Mm.289584	2	0.001004016	3	0.000318674					
Mm.275742	2	0.001004016	0	0					
Mm.22699	0	0	2	0.00021245					
Mm.24430	0	0	2	0.00021245					
Mm.19133	0	0	1	0.000106225					
Mm.14297	0	0	1	0.000106225					
Mm.24105	0	0	1	0.000106225					
Mm.273292	1	0.000502008	0	0					
>GO:0004718	4	0.002793296	1	0.000138122	0.049447514	0.003216374	0.084917157	Janus kinase activity	F
Mm.289657	2	0.001004016	1	0.000106225					
Mm.275839	2	0.001004016	0	0					
>GO:0001933	4	0.002793296	1	0.000138122	0.049447514	0.003216374	0.084917157	negative regulation of protei	P
Mm.22315	4	0.002008032	1	0.000106225					
>GO:0051209	4	0.002793296	1	0.000138122	0.049447514	0.003216374	0.084917157	release of sequestered calc	P
Mm.22315	4	0.002008032	1	0.000106225					
>GO:0051282	4	0.002793296	1	0.000138122	0.049447514	0.003216374	0.084917157	regulation of sequestering o	P
Mm.22315	4	0.002008032	1	0.000106225					
>GO:0051084	1	0.000698324	60	0.008287293	11.86740331	0.003010184	0.084917157	posttranslational protein fold	P
Mm.16373	0	0	1	0.000106225					
Mm.290774	1	0.000502008	17	0.001805821					
Mm.336743	0	0	36	0.003824092					
Mm.270681	0	0	4	0.000424899					
Mm.154994	0	0	1	0.000106225					
Mm.249164	0	0	1	0.000106225					
>GO:0007186	38	0.026536313	109	0.015055249	0.567345158	0.003044567	0.084917157	G-protein coupled receptor	P
Mm.277351	2	0.001004016	0	0					
Mm.235018	0	0	1	0.000106225					
Mm.192026	0	0	1	0.000106225					

Mm.285993	3	0.001506024	9	0.000956023
Mm.329243	0	0	3	0.000318674
Mm.298775	0	0	3	0.000318674
Mm.22680	0	0	1	0.000106225
Mm.342315	0	0	3	0.000318674
Mm.327835	0	0	1	0.000106225
Mm.229532	1	0.000502008	0	0
Mm.28265	0	0	1	0.000106225
Mm.243722	0	0	4	0.000424899
Mm.193925	0	0	4	0.000424899
Mm.313181	2	0.001004016	0	0
Mm.254629	0	0	1	0.000106225
Mm.196464	1	0.000502008	1	0.000106225
Mm.195898	0	0	2	0.00021245
Mm.125770	4	0.002008032	4	0.000424899
Mm.2344	0	0	1	0.000106225
Mm.17604	0	0	1	0.000106225
Mm.234342	0	0	1	0.000106225
Mm.329700	0	0	1	0.000106225
Mm.140804	0	0	1	0.000106225
Mm.290834	0	0	1	0.000106225
Mm.279400	0	0	2	0.00021245
Mm.93335	0	0	4	0.000424899
Mm.18509	0	0	1	0.000106225
Mm.28262	4	0.002008032	1	0.000106225
Mm.8055	1	0.000502008	0	0
Mm.103354	2	0.001004016	0	0
Mm.320183	0	0	5	0.000531124
Mm.349120	0	0	1	0.000106225
Mm.83689	1	0.000502008	0	0
Mm.264860	0	0	1	0.000106225
Mm.334648	0	0	2	0.00021245
Mm.29279	0	0	2	0.00021245
Mm.373635	1	0.000502008	5	0.000531124
Mm.70979	1	0.000502008	0	0
Mm.286753	0	0	2	0.00021245
Mm.39863	0	0	1	0.000106225
Mm.298256	0	0	2	0.00021245
Mm.273142	0	0	2	0.00021245
Mm.44606	0	0	1	0.000106225
Mm.103439	2	0.001004016	0	0
Mm.159019	1	0.000502008	0	0
Mm.242413	0	0	1	0.000106225
Mm.272974	0	0	1	0.000106225
Mm.87046	0	0	1	0.000106225
Mm.310036	0	0	2	0.00021245
Mm.325086	1	0.000502008	0	0
Mm.5305	3	0.001506024	17	0.001805821
Mm.222685	1	0.000502008	0	0
Mm.44241	0	0	1	0.000106225
Mm.8004	1	0.000502008	0	0
Mm.228	0	0	1	0.000106225
Mm.201322	1	0.000502008	0	0
Mm.26150	2	0.001004016	2	0.00021245
Mm.248291	1	0.000502008	1	0.000106225
Mm.12091	1	0.000502008	2	0.00021245

Mm.21739	0	0	2	0.00021245					
Mm.203747	1	0.000502008	0	0					
Mm.159724	0	0	1	0.000106225					
>GO:0000178	4	0.002793296	1	0.000138122	0.049447514	0.003216374	0.084917157	exosome (RNase complex)	C
Mm.275788	1	0.000502008	0	0					
Mm.29253	3	0.001506024	1	0.000106225					
>GO:0016765	2	0.001396648	70	0.009668508	6.922651934	0.002765675	0.084917157	transferase activity, transfer	F
Mm.2662	0	0	6	0.000637349					
Mm.37199	0	0	15	0.001593372					
Mm.272792	0	0	12	0.001274697					
Mm.282351	0	0	1	0.000106225					
Mm.299292	0	0	3	0.000318674					
Mm.29457	0	0	6	0.000637349					
Mm.14796	0	0	1	0.000106225					
Mm.29640	0	0	1	0.000106225					
Mm.29815	0	0	12	0.001274697					
Mm.277831	0	0	2	0.00021245					
Mm.262096	0	0	1	0.000106225					
Mm.371560	0	0	5	0.000531124					
Mm.371950	0	0	1	0.000106225					
Mm.11827	1	0.000502008	0	0					
Mm.210305	0	0	2	0.00021245					
Mm.18652	0	0	2	0.00021245					
Mm.331035	1	0.000502008	0	0					
>GO:0001704	4	0.002793296	1	0.000138122	0.049447514	0.003216374	0.084917157	formation of primary germ la	P
Mm.7320	1	0.000502008	1	0.000106225					
Mm.30039	1	0.000502008	0	0					
Mm.29790	2	0.001004016	0	0					
>GO:0051208	4	0.002793296	1	0.000138122	0.049447514	0.003216374	0.084917157	sequestering of calcium ion	P
Mm.22315	4	0.002008032	1	0.000106225					
>GO:0051238	4	0.002793296	1	0.000138122	0.049447514	0.003216374	0.084917157	sequestering of metal ion	P
Mm.22315	4	0.002008032	1	0.000106225					
>GO:0008624	13	0.009078212	23	0.003176796	0.349936252	0.003191425	0.084917157	induction of apoptosis by ex	P
Mm.222867	0	0	1	0.000106225					
Mm.280768	0	0	1	0.000106225					
Mm.25988	13	0.006526104	15	0.001593372					
Mm.34405	0	0	1	0.000106225					
Mm.22279	0	0	5	0.000531124					
>GO:0004716	4	0.002793296	1	0.000138122	0.049447514	0.003216374	0.084917157	receptor signaling protein ty	F
Mm.289657	2	0.001004016	1	0.000106225					
Mm.275839	2	0.001004016	0	0					
>GO:0006468	62	0.043296089	204	0.028176796	0.650793085	0.003199325	0.084917157	protein amino acid phospho	P
Mm.25720	1	0.000502008	0	0					
Mm.216227	0	0	1	0.000106225					
Mm.74982	0	0	1	0.000106225					
Mm.255075	0	0	1	0.000106225					
Mm.27557	0	0	2	0.00021245					
Mm.244236	1	0.000502008	0	0					
Mm.184163	1	0.000502008	2	0.00021245					
Mm.254144	0	0	3	0.000318674					
Mm.329515	1	0.000502008	0	0					
Mm.237825	0	0	2	0.00021245					
Mm.39089	0	0	2	0.00021245					
Mm.13806	0	0	2	0.00021245					
Mm.29133	2	0.001004016	3	0.000318674					
Mm.235182	0	0	1	0.000106225					

Mm.327591	0	0	1	0.000106225
Mm.273049	0	0	2	0.00021245
Mm.16110	0	0	1	0.000106225
Mm.247788	0	0	5	0.000531124
Mm.281367	0	0	2	0.00021245
Mm.6839	0	0	3	0.000318674
Mm.298798	0	0	1	0.000106225
Mm.16753	4	0.002008032	1	0.000106225
Mm.3996	0	0	3	0.000318674
Mm.1761	1	0.000502008	2	0.00021245
Mm.21974	0	0	1	0.000106225
Mm.298893	1	0.000502008	0	0
Mm.51136	0	0	5	0.000531124
Mm.258986	1	0.000502008	1	0.000106225
Mm.271976	0	0	2	0.00021245
Mm.254494	0	0	1	0.000106225
Mm.23788	0	0	1	0.000106225
Mm.16340	0	0	2	0.00021245
Mm.279400	0	0	2	0.00021245
Mm.20827	0	0	3	0.000318674
Mm.23790	0	0	1	0.000106225
Mm.257925	0	0	1	0.000106225
Mm.42201	1	0.000502008	0	0
Mm.275742	2	0.001004016	0	0
Mm.289657	2	0.001004016	1	0.000106225
Mm.275839	2	0.001004016	0	0
Mm.209150	1	0.000502008	0	0
Mm.247073	0	0	2	0.00021245
Mm.358618	0	0	6	0.000637349
Mm.124176	0	0	2	0.00021245
Mm.8149	0	0	2	0.00021245
Mm.272206	0	0	2	0.00021245
Mm.28678	0	0	1	0.000106225
Mm.268668	0	0	2	0.00021245
Mm.317339	0	0	2	0.00021245
Mm.116649	0	0	1	0.000106225
Mm.9001	0	0	1	0.000106225
Mm.3401	1	0.000502008	0	0
Mm.10504	0	0	1	0.000106225
Mm.260521	0	0	1	0.000106225
Mm.259333	3	0.001506024	0	0
Mm.291554	1	0.000502008	0	0
Mm.329993	0	0	1	0.000106225
Mm.30039	1	0.000502008	0	0
Mm.25594	0	0	1	0.000106225
Mm.370198	0	0	1	0.000106225
Mm.311918	0	0	1	0.000106225
Mm.10027	0	0	1	0.000106225
Mm.224246	0	0	2	0.00021245
Mm.252210	0	0	1	0.000106225
Mm.6710	4	0.002008032	1	0.000106225
Mm.301827	0	0	1	0.000106225
Mm.28405	0	0	2	0.00021245
Mm.257989	0	0	2	0.00021245
Mm.15252	1	0.000502008	1	0.000106225
Mm.288728	1	0.000502008	0	0

Mm.249363	0	0	5	0.000531124
Mm.257120	1	0.000502008	0	0
Mm.29071	1	0.000502008	1	0.000106225
Mm.172346	1	0.000502008	1	0.000106225
Mm.29515	0	0	2	0.00021245
Mm.271898	0	0	5	0.000531124
Mm.374864	0	0	6	0.000637349
Mm.311948	0	0	1	0.000106225
Mm.191949	0	0	3	0.000318674
Mm.333349	0	0	1	0.000106225
Mm.235194	0	0	1	0.000106225
Mm.126976	0	0	2	0.00021245
Mm.248907	0	0	2	0.00021245
Mm.172897	0	0	3	0.000318674
Mm.196581	0	0	2	0.00021245
Mm.39253	2	0.001004016	0	0
Mm.27970	0	0	1	0.000106225
Mm.311337	0	0	3	0.000318674
Mm.8385	0	0	3	0.000318674
Mm.21495	3	0.001506024	0	0
Mm.68933	1	0.000502008	0	0
Mm.140948	3	0.001506024	1	0.000106225
Mm.260114	0	0	3	0.000318674
Mm.318364	0	0	1	0.000106225
Mm.19073	0	0	5	0.000531124
Mm.269211	0	0	1	0.000106225
Mm.202606	0	0	2	0.00021245
Mm.291936	1	0.000502008	1	0.000106225
Mm.18856	0	0	5	0.000531124
Mm.279308	0	0	1	0.000106225
Mm.24337	0	0	2	0.00021245
Mm.34580	0	0	1	0.000106225
Mm.200770	0	0	1	0.000106225
Mm.234472	0	0	1	0.000106225
Mm.28761	3	0.001506024	2	0.00021245
Mm.143817	0	0	1	0.000106225
Mm.37617	1	0.000502008	0	0
Mm.253721	1	0.000502008	1	0.000106225
Mm.31486	3	0.001506024	0	0
Mm.18539	0	0	1	0.000106225
Mm.260516	0	0	2	0.00021245
Mm.193924	0	0	1	0.000106225
Mm.21876	1	0.000502008	0	0
Mm.44490	0	0	3	0.000318674
Mm.227202	0	0	2	0.00021245
Mm.35290	0	0	1	0.000106225
Mm.276669	0	0	2	0.00021245
Mm.262707	0	0	1	0.000106225
Mm.276063	0	0	1	0.000106225
Mm.340943	0	0	2	0.00021245
Mm.26908	0	0	8	0.000849798
Mm.25559	0	0	1	0.000106225
Mm.22315	4	0.002008032	1	0.000106225
Mm.290924	1	0.000502008	0	0
Mm.318430	0	0	1	0.000106225
Mm.374904	0	0	1	0.000106225

Mm.223717	0	0	1	0.000106225					
Mm.7320	1	0.000502008	1	0.000106225					
Mm.100399	1	0.000502008	1	0.000106225					
Mm.288726	0	0	2	0.00021245					
>GO:0030292	4	0.002793296	1	0.000138122	0.049447514	0.003216374	0.084917157	protein tyrosine kinase inhib	F
Mm.22315	4	0.002008032	1	0.000106225					
>GO:0051085	1	0.000698324	60	0.008287293	11.86740331	0.003010184	0.084917157	chaperone cofactor depend	P
Mm.16373	0	0	1	0.000106225					
Mm.290774	1	0.000502008	17	0.001805821					
Mm.336743	0	0	36	0.003824092					
Mm.270681	0	0	4	0.000424899					
Mm.154994	0	0	1	0.000106225					
Mm.249164	0	0	1	0.000106225					
>GO:0051283	4	0.002793296	1	0.000138122	0.049447514	0.003216374	0.084917157	negative regulation of seque	P
Mm.22315	4	0.002008032	1	0.000106225					
>GO:0005732	0	0	50	0.006906077	inf	0.003047858	0.084917157	small nucleolar ribonucleop	C
Mm.250030	0	0	11	0.001168472					
Mm.100113	0	0	16	0.001699596					
Mm.4071	0	0	11	0.001168472					
Mm.371669	0	0	2	0.00021245					
Mm.88216	0	0	2	0.00021245					
Mm.274995	0	0	3	0.000318674					
Mm.306162	0	0	1	0.000106225					
Mm.246693	0	0	3	0.000318674					
Mm.275158	0	0	1	0.000106225					
>GO:0048332	4	0.002793296	1	0.000138122	0.049447514	0.003216374	0.084917157	mesoderm morphogenesis	P
Mm.7320	1	0.000502008	1	0.000106225					
Mm.30039	1	0.000502008	0	0					
Mm.29790	2	0.001004016	0	0					
>GO:0005730	12	0.008379888	146	0.020165746	2.406445672	0.003294486	0.086325449	nucleolus	C
Mm.12864	0	0	1	0.000106225					
Mm.290015	0	0	1	0.000106225					
Mm.4283	0	0	1	0.000106225					
Mm.282719	0	0	3	0.000318674					
Mm.22670	1	0.000502008	0	0					
Mm.154378	0	0	6	0.000637349					
Mm.338720	0	0	2	0.00021245					
Mm.6343	3	0.001506024	17	0.001805821					
Mm.28764	5	0.00251004	3	0.000318674					
Mm.2845	0	0	3	0.000318674					
Mm.2215	0	0	2	0.00021245					
Mm.4237	0	0	2	0.00021245					
Mm.297196	1	0.000502008	0	0					
Mm.250030	0	0	11	0.001168472					
Mm.188413	0	0	2	0.00021245					
Mm.88512	0	0	2	0.00021245					
Mm.29810	0	0	2	0.00021245					
Mm.220367	0	0	1	0.000106225					
Mm.25264	0	0	1	0.000106225					
Mm.257482	0	0	3	0.000318674					
Mm.28630	0	0	2	0.00021245					
Mm.28659	0	0	1	0.000106225					
Mm.140380	0	0	5	0.000531124					
Mm.227258	0	0	4	0.000424899					
Mm.280311	0	0	1	0.000106225					
Mm.271715	0	0	1	0.000106225					

Mm.29906	0	0	2	0.00021245					
Mm.78861	0	0	4	0.000424899					
Mm.288015	2	0.001004016	0	0					
Mm.33437	0	0	2	0.00021245					
Mm.210845	0	0	7	0.000743573					
Mm.284592	0	0	2	0.00021245					
Mm.358640	0	0	1	0.000106225					
Mm.244820	0	0	4	0.000424899					
Mm.100113	0	0	16	0.001699596					
Mm.4071	0	0	11	0.001168472					
Mm.371669	0	0	2	0.00021245					
Mm.88216	0	0	2	0.00021245					
Mm.274995	0	0	3	0.000318674					
Mm.306162	0	0	1	0.000106225					
Mm.246693	0	0	3	0.000318674					
Mm.275158	0	0	1	0.000106225					
Mm.3458	0	0	1	0.000106225					
Mm.317557	0	0	2	0.00021245					
Mm.288730	0	0	1	0.000106225					
Mm.279861	0	0	2	0.00021245					
Mm.38344	0	0	2	0.00021245					
>GO:0004869	11	0.007681564	17	0.002348066	0.30567554	0.003343278	0.086950182	cysteine protease inhibitor a	F
Mm.2026	0	0	4	0.000424899					
Mm.8552	0	0	4	0.000424899					
Mm.676	0	0	2	0.00021245					
Mm.290908	0	0	1	0.000106225					
Mm.4263	0	0	1	0.000106225					
Mm.6856	11	0.005522088	4	0.000424899					
Mm.280779	0	0	1	0.000106225					
>GO:0042803	19	0.013268156	42	0.005801105	0.437220122	0.003542595	0.091451438	protein homodimerization ac	F
Mm.347407	1	0.000502008	0	0					
Mm.1843	14	0.007028112	5	0.000531124					
Mm.371565	2	0.001004016	20	0.002124495					
Mm.21535	0	0	1	0.000106225					
Mm.269657	0	0	3	0.000318674					
Mm.945	0	0	1	0.000106225					
Mm.277217	1	0.000502008	0	0					
Mm.29098	0	0	8	0.000849798					
Mm.331881	0	0	2	0.00021245					
Mm.3960	0	0	1	0.000106225					
Mm.350347	1	0.000502008	1	0.000106225					
>GO:0016481	31	0.021648045	84	0.01160221	0.535947246	0.003613022	0.0925837	negative regulation of trans	P
Mm.332919	1	0.000502008	0	0					
Mm.3596	1	0.000502008	1	0.000106225					
Mm.5098	3	0.001506024	0	0					
Mm.25903	0	0	1	0.000106225					
Mm.147946	0	0	2	0.00021245					
Mm.272551	2	0.001004016	7	0.000743573					
Mm.272361	2	0.001004016	4	0.000424899					
Mm.271814	0	0	1	0.000106225					
Mm.8256	0	0	1	0.000106225					
Mm.103638	0	0	1	0.000106225					
Mm.22690	0	0	1	0.000106225					
Mm.269211	0	0	1	0.000106225					
Mm.271709	0	0	4	0.000424899					
Mm.287982	1	0.000502008	0	0					

Mm.29142	0	0	2	0.00021245					
Mm.28910	0	0	3	0.000318674					
Mm.25059	1	0.000502008	8	0.000849798					
Mm.256765	3	0.001506024	0	0					
Mm.4742	1	0.000502008	4	0.000424899					
Mm.28840	2	0.001004016	2	0.00021245					
Mm.374790	0	0	1	0.000106225					
Mm.23790	0	0	1	0.000106225					
Mm.7320	1	0.000502008	1	0.000106225					
Mm.2537	1	0.000502008	0	0					
Mm.168965	0	0	1	0.000106225					
Mm.273862	0	0	1	0.000106225					
Mm.15755	0	0	2	0.00021245					
Mm.22584	0	0	2	0.00021245					
Mm.15701	0	0	7	0.000743573					
Mm.305561	0	0	3	0.000318674					
Mm.39496	1	0.000502008	0	0					
Mm.25339	0	0	1	0.000106225					
Mm.270186	9	0.004518072	8	0.000849798					
Mm.371732	0	0	1	0.000106225					
Mm.272826	0	0	1	0.000106225					
Mm.173271	0	0	1	0.000106225					
Mm.321633	0	0	5	0.000531124					
Mm.358714	1	0.000502008	0	0					
Mm.318430	0	0	1	0.000106225					
Mm.24621	0	0	1	0.000106225					
Mm.57223	0	0	1	0.000106225					
Mm.351459	1	0.000502008	2	0.00021245					
>GO:0018212	7	0.004888268	7	0.000966851	0.197790055	0.003861875	0.09752633	peptidyl-tyrosine modificatio	P
Mm.25720	1	0.000502008	0	0					
Mm.1761	1	0.000502008	2	0.00021245					
Mm.289657	2	0.001004016	1	0.000106225					
Mm.275839	2	0.001004016	0	0					
Mm.247073	0	0	2	0.00021245					
Mm.290924	1	0.000502008	0	0					
Mm.318430	0	0	1	0.000106225					
Mm.374904	0	0	1	0.000106225					
>GO:0018108	7	0.004888268	7	0.000966851	0.197790055	0.003861875	0.09752633	peptidyl-tyrosine phosphory	P
Mm.25720	1	0.000502008	0	0					
Mm.1761	1	0.000502008	2	0.00021245					
Mm.289657	2	0.001004016	1	0.000106225					
Mm.275839	2	0.001004016	0	0					
Mm.247073	0	0	2	0.00021245					
Mm.290924	1	0.000502008	0	0					
Mm.318430	0	0	1	0.000106225					
Mm.374904	0	0	1	0.000106225					
