# Supplementary material for: GO-Diff: Mining functional differentiation between EST-based transcriptomes
Source: BMC Bioinformatics. 2006 Feb 16;7:72. doi: 10.1186/1471-2105-7-72 (PMC1388240; doi:10.1186/1471-2105-7-72)
Supplement: Additional File 3 — List of Meta-analysis-supported GO terms identified by GO-Diff in the comparison between human and mouse liver Description: The number in the table is the EST coverage ratio (human/mouse) of the GO term. It is represented by "inf" when the EST coverage level in mouse is zero, and by "1" when no significant (ECRG >= 3, FDR <= 0.1) differences are found in the two sets of dbEST libraries. [file 1471-2105-7-72-S3.pdf]

Table 3. Meta-analysis-supported GO terms in the comparison between human and mouse liver.

ECRG is represented by "inf" when the RECLG of mouse is zero, and by "1" when no significant differences are found in the two sets of dbEST libraries. In the comparison and following meta-analysis, we used the following criteria to select differentially represented GO terms. First, the GO term should be differentially presented in at least half of the comparisons with ECRG above 3 and false discovery rate below 0.1. Second, no conflicts of up-regulation and down-regulation of RECLG were observed in all the comparisons. Third, the GO term should be supported by at least one comparison in both mode 1 and 3.

	ECRG (human/mouse)											
	mode 3					mode 1						
GO-ID	1883_13859	1883_1365	1883_13052	1778_252	1299_252	1883_13859	1883_1365	1883_13052	1778_252	1299_252	GO description	GO name space
GO:0000122	20	1	12	1	1	9.2	16	8.2	1	1	negative regulation of transcription from Pol II promoter	P
GO:0000267	3.3	1	5.2	1	1	5.6	5.4	8.6	4.7	1	cell fraction	C
GO:0000302	0.0079	0.024	0.0095	0.038	0.046	0.0058	0.062	0	0.21	0.047	response to reactive oxygen species	P
GO:0000313	0.064	1	0.23	1	0.32	0.027	1	0.14	1	1	organellar ribosome	C
GO:0000502	0.33	1	0.29	1	0.15	0.28	0.29	0.26	1	0.2	proteasome complex (sensu Eukarya)	C
GO:0000781	inf	1	inf	1	1	1	9.7	9.1	1	inf	chromosome, telomeric region	C
GO:0001540	0	1	0	1	0.056	0	1	0	1	0.038	beta-amyloid binding	F
GO:0001542	0	0.079	0.0079	1	1	0	0	0	1	1	ovulation (sensu Mammalia)	P
GO:0001871	1	5.2	3.2	1	1	1	3.6	2.3	5.3	1	pattern binding	F
GO:0003677	4.3	3.1	4.4	1	1	2.1	2	2.1	1	1	DNA binding	F
GO:0003690	inf	inf	inf	1	1	6.8	20	8.2	1	inf	double-stranded DNA binding	F
GO:0003697	17	21	21	1	1	inf	inf	inf	1	inf	single-stranded DNA binding	F
GO:0003712	3.6	1	4.9	1	1	4	4.7	4	1	1	transcription cofactor activity	F
GO:0003713	4.2	4	3.9	1	1	4	4.1	2.8	1	1	transcription coactivator activity	F
GO:0003779	78	16	38	1	1	4.6	1	2.1	1	1	actin binding	F
GO:0003812	1	inf	inf	1	7.1	1	inf	inf	inf	6.3	alternative-complement-pathway C3/C5 convertase activity	F
GO:0004252	5.1	4.9	7.1	6.8	5.2	45	25	34	2.3	5.2	serine-type endopeptidase activity	F
GO:0004263	inf	inf	inf	6.1	6.4	inf	inf	inf	2.6	5.7	chymotrypsin activity	F
GO:0004295	inf	inf	inf	6.1	6.3	inf	inf	inf	2.5	5.6	trypsin activity	F
GO:0004601	0.022	0.13	0.16	0.06	0.12	0.3	0.11	0.17	0.3	0.1	peroxidase activity	F
GO:0004602	0.0085	0	0.077	0.025	0.054	0.0059	0	0.075	0.21	0.081	glutathione peroxidase activity	F
GO:0004655	0.18	1	0	1	0	0.14	1	0	1	1	porphobilinogen synthase activity	F
GO:0004714	inf	1	1	1	inf	inf	inf	1	1	29	transmembrane receptor protein tyrosine kinase activity	F
GO:0004721	9.9	7.4	12	1	1	3.7	3.4	1	1	1	phosphoprotein phosphatase activity	F
GO:0004857	4.7	6	6.4	1	1	2.4	3.3	4	7.1	1	enzyme inhibitor activity	F
GO:0004866	5.1	7.5	7.3	1	1	2.7	4.2	4.9	6.9	1	endopeptidase inhibitor activity	F
GO:0004867	6.2	6.7	6.4	1	1	3.3	3.7	4.5	4.6	1	serine-type endopeptidase inhibitor activity	F
GO:0004871	5.7	3.6	5	1	1	3.2	2.8	2.3	1	1	signal transducer activity	F
GO:0005096	inf	inf	inf	1	1	14	17	6.9	1	1	GTPase activator activity	F
GO:0005102	7.7	9.5	9.5	inf	1	3.2	4.1	4	4.1	1	receptor binding	F
GO:0005386	3.3	1	1	1	1	2.4	2.4	2.3	4.3	1	carrier activity	F
GO:0005504	15	15	12	4.1	55	185	227	208	181	57	fatty acid binding	F
GO:0005507	16	16	13	4.2	109	34	41	38	60	48	copper ion binding	F
GO:0005518	7.8	1	1	1	5.7	inf	1	inf	1	inf	collagen binding	F
GO:0005576	5.8	6.7	6.7	1	1	1	2.5	3	3.2	1	extracellular	C
GO:0005577	25	33	77	inf	4.6	inf	inf	inf	inf	inf	fibrinogen complex	C
GO:0005578	4.2	4.5	6.1	1	1	4	2.6	2.3	1	1	extracellular matrix	C
GO:0005615	5.2	6	6.1	4.7	1	1	1	2.1	1	1	extracellular space	C
GO:0005625	11	9.2	21	14	5.2	16	16	38	40	42	soluble fraction	C
GO:0005732	0.071	1	1	1	1	0.012	0.086	0.041	1	0.1	small nucleolar ribonucleoprotein complex	C
GO:0005737	0.33	1	1	1	1	0.41	0.47	0.46	0.41	1	cytoplasm	C
GO:0005739	0.07	0.1	0.15	0.15	0.21	0.097	0.11	0.17	0.13	0.25	mitochondrion	C
GO:0005740	0.25	1	1	0.14	0.27	0.15	0.22	1	0.25	1	mitochondrial membrane	C
GO:0005743	0.2	1	1	0.1	0.3	0.13	0.22	0.43	0.23	1	mitochondrial inner membrane	C
GO:0005746	0.15	0	1	0.13	0.31	0.043	0.039	0.18	1	1	mitochondrial electron transport chain	C
GO:0005753	0.017	0.1	0.32	0.079	0.18	0.029	1	1	1	1	proton-transporting ATP synthase complex (sensu Eukarya)	C
GO:0005761	0.064	1	0.23	1	0.32	0.027	1	0.14	1	1	mitochondrial ribosome	C
GO:0005829	0.041	0.3	0.14	1	1	0.054	0.19	0.15	1	1	cytosol	C
GO:0005839	0.11	0.085	0.22	1	0.19	0.17	0.077	0.24	1	0.31	proteasome core complex (sensu Eukarya)	C
GO:0005886	3.7	3.2	5.1	1	1	2.3	2.1	1	1	1	plasma membrane	C
GO:0005887	3.7	1	3.7	1	1	6.5	5	4.6	1	1	integral to plasma membrane	C
GO:0006006	3.4	5.1	6	1	1	1	2.1	2.2	1	1	glucose metabolism	P
GO:0006007	22	45	50	1	1	1	2.9	3.6	1	1	glucose catabolism	P
GO:0006096	21	40	47	1	1	1	4.5	6.1	1	1	glycolysis	P
GO:0006119	0.048	0.23	1	0.087	0.18	0.16	0.23	0.49	1	1	oxidative phosphorylation	P
GO:0006163	0.19	0.2	1	0.062	0.15	0.19	0.27	0.39	1	1	purine nucleotide metabolism	P
GO:0006164	0.16	0.2	1	0.062	0.15	0.18	0.25	0.37	1	1	purine nucleotide biosynthesis	P
GO:0006260	8.4	6.8	6.4	1	1	1	2.2	2.4	1	1	DNA replication	P
GO:0006325	6.2	3.5	9.6	1	1	2.5	2.6	3.2	1	1	establishment and/or maintenance of chromatin architecture	P
GO:0006350	3.8	1	3.3	1	1	2.4	2.3	2	1	1	transcription	P
GO:0006351	4.1	1	3.5	1	1	2.7	2.5	2.2	1	1	transcription, DNA-dependent	P
GO:0006355	4.4	3.1	3.7	1	1	2.8	2.7	2.3	1	1	regulation of transcription, DNA-dependent	P
GO:0006357	6.9	3.8	6.7	1	1	5.3	7.6	5.3	1	1	regulation of transcription from Pol II promoter	P
GO:0006366	5.5	3.2	5.8	1	1	4.8	5.7	4.4	4.8	1	transcription from Pol II promoter	P
GO:0006412	0.15	1	0.3	1	1	0.19	0.45	0.3	1	1	protein biosynthesis	P
GO:0006461	9.2	7.4	13	1	1	28	23	24	1	6.4	protein complex assembly	P
GO:0006486	inf	1	inf	1	1	8	6.5	5.5	1	1	protein amino acid glycosylation	P
GO:0006720	0.12	1	1	1	0.089	0.11	0	1	1	0.15	isoprenoid metabolism	P
GO:0006732	1	1	1	1	0.29	0.43	0.47	1	0.18	0.3	coenzyme metabolism	P
GO:0006752	0.14	0.32	1	1	0.2	0.23	0.27	0.44	1	1	group transfer coenzyme metabolism	P
GO:0006753	0.047	0.24	1	0.1	0.13	0.11	0.18	0.33	1	1	nucleoside phosphate metabolism	P
GO:0006754	0.047	0.24	1	0.1	0.13	0.11	0.18	0.33	1	1	ATP biosynthesis	P
GO:0006800	0.22	0.22	1	0.13	0.15	0.36	0.15	0.27	1	0.2	oxygen and reactive oxygen species metabolism	P
GO:0006809	inf	inf	inf	1	1	1	3.9	1	1	8.6	nitric oxide biosynthesis	P
GO:0006818	1	1	1	0.12	0.16	0.32	0.33	0.37	1	1	hydrogen transport	P
GO:0006820	6.2	6.1	5.9	1	1	2.8	1	2.5	1	1	anion transport	P
GO:0006917	0.21	0.19	0.28	0.11	0.1	0.3	0.21	0.21	0.36	0.11	induction of apoptosis	P
GO:0006928	17	4.2	4.3	1	1	10	3	2.1	8.7	1	cell motility	P
GO:0006935	4.7	1	7.6	1	1	3.1	3.6	3.1	1	1	chemotaxis	P
GO:0006936	48	6.9	6	inf	18	94	15	11	23	30	muscle contraction	P
GO:0006937	inf	inf	inf	inf	70	inf	inf	inf	21	45	regulation of muscle contraction	P
GO:0006939	1	11	1	inf	30	1	inf	inf	1	1	smooth muscle contraction	P
GO:0006952	6.1	6.8	6.9	3.3	1	1	3.3	4.5	4.9	1	defense response	P
GO:0006953	18	14	20	1	1	1	3.1	4.6	8.4	1	acute-phase response	P
GO:0006954	24	29	26	17	1	1	3.6	5.3	4.9	1	inflammatory response	P
GO:0006955	6	6.8	6.6	1	1	1	3.3	4.4	4	1	immune response	P
GO:0006956	25	91	72	inf	4.9	8	24	22	19	5.3	complement activation	P
GO:0006957	11	49	27	1	3.4	8.7	43	28	inf	4	complement activation, alternative pathway	P
GO:0006958	20	79	58	inf	5.5	20	58	52	inf	6.6	complement activation, classical pathway	P
GO:0006959	5.5	15	12	inf	4.5	9.8	22	19	19	5.2	humoral immune response	P
GO:0006968	24	1	23	1	1	inf	inf	inf	1	1	cellular defense response	P

GO:0006979	0.17	0.18	0.29	0.15	0.14	0.35	0.15	0.24	1	0.19	response to oxidative stress	P
GO:0006997	6.1	4	8.7	1	1	2.4	2.7	2.9	1	1	nuclear organization and biogenesis	P
GO:0007001	5.5	4	8.4	1	1	2.6	3	3.1	1	1	chromosome organization and biogenesis (sensu Eukarya)	P
GO:0007046	1	1	1	1	0.24	0.027	0.027	0.044	1	0.064	ribosome biogenesis	P
GO:0007154	4.7	3.7	4.8	1	1	2.8	2.6	2.3	1	1	cell communication	P
GO:0007155	3.8	1	3.9	1	1	4	3.5	2.5	1	1	cell adhesion	P
GO:0007165	4.6	4.2	5.2	1	1	2.8	2.7	2.4	1	1	signal transduction	P
GO:0007166	5	5.9	8.2	1	1	2.5	2.9	3.2	1	1	cell surface receptor linked signal transduction	P
GO:0007186	6.5	11	9.1	3.5	3.8	1	1	1	1	4	G-protein coupled receptor protein signaling pathway	P
GO:0007270	1	1	0.14	1	0.054	0.23	1	0	1	0.037	nerve-nerve synaptic transmission	P
GO:0007292	0.1	0.2	0.22	1	1	0.02	0.11	0.017	1	1	female gamete generation	P
GO:0007399	6.3	7.1	3.1	12	1	2.2	2.6	1	1	1	neurogenesis	P
GO:0007416	0	1	1	1	0.095	0.24	1	0.25	1	0.037	synaptogenesis	P
GO:0007517	inf	inf	inf	1	1	46	8.2	4.6	1	1	muscle development	P
GO:0007565	31	11	8.9	1	1	inf	inf	inf	1	175	pregnancy	P
GO:0007596	14	19	35	11	1	6.2	8.6	20	4.4	3.2	blood coagulation	P
GO:0007599	14	19	36	11	1	6.3	8.7	21	4.4	3.2	hemostasis	P
GO:0008015	9.7	13	26	inf	3.4	9.4	13	29	36	5.9	circulation	P
GO:0008034	1	1	inf	inf	14	1	1	15	6.7	15	lipoprotein binding	F
GO:0008035	1	1	inf	inf	14	1	1	inf	inf	16	high-density lipoprotein binding	F
GO:0008043	1	inf	inf	1	inf	1	inf	inf	1	inf	ferritin complex	C
GO:0008047	12	7.8	11	1	1	7.3	9.3	5.1	1	1	enzyme activator activity	F
GO:0008121	0.059	0	1	0.056	0.15	0.24	1	1	1	1	ubiquinol-cytochrome-c reductase activity	F
GO:0008134	3.9	3.3	5.3	1	1	2.8	3.1	2.6	1	1	transcription factor binding	F
GO:0008144	16	16	13	4	153	inf	inf	inf	inf	1359	drug binding	F
GO:0008207	0.013	0.079	0.048	1	0	0.02	0	0.0083	1	0	C21-steroid hormone metabolism	P
GO:0008217	13	18	44	inf	3.3	31	46	138	88	13	regulation of blood pressure	P
GO:0008219	4.3	4.5	3.9	1	3.5	3.1	3.6	3.5	7.4	3.8	cell death	P
GO:0008236	4.8	4.6	6.8	6.9	5.2	46	27	36	2.3	5.3	serine-type peptidase activity	F
GO:0008283	3.8	3.2	5.5	3.3	1	1	1	2.8	2.7	4.3	cell proliferation	P
GO:0008284	31	38	84	inf	4	11	14	37	inf	26	positive regulation of cell proliferation	P
GO:0008289	7.2	7.8	6.6	3.3	1	4.6	5.6	5.3	5.1	1	lipid binding	F
GO:0008553	0.031	0.092	0.26	0.011	0	0.01	0	0.085	0	0	hydrogen-exporting ATPase activity, phosphorylative mechanis	F
GO:0008585	0	0.079	0.0079	1	1	0	0	0	1	1	female gonad development	P
GO:0008629	0.049	0.074	0.085	0.0042	0	0.036	0.065	0.071	0.023	0.01	induction of apoptosis by intracellular signals	P
GO:0008631	0	0	0	0.0042	0	0	0	0	0	0	induction of apoptosis by oxidative stress	P
GO:0009055	0.16	0	1	0.042	0.29	1	1	1	0.11	0.13	electron carrier activity	F
GO:0009100	inf	1	inf	1	1	11	7.7	6.9	1	1	glycoprotein metabolism	P
GO:0009101	inf	1	inf	1	1	9.2	7.7	5.5	1	1	glycoprotein biosynthesis	P
GO:0009108	0.15	0.32	1	1	0.16	0.3	0.22	1	1	1	coenzyme biosynthesis	P
GO:0009141	0.082	0.2	1	0.048	0.14	0.13	0.2	0.38	1	1	nucleoside triphosphate metabolism	P
GO:0009142	0.054	0.2	1	0.043	0.13	0.11	0.17	0.34	1	1	nucleoside triphosphate biosynthesis	P
GO:0009144	0.082	0.2	1	0.051	0.13	0.13	0.2	0.36	1	1	purine nucleoside triphosphate metabolism	P
GO:0009145	0.054	0.2	1	0.051	0.13	0.11	0.17	0.34	1	1	purine nucleoside triphosphate biosynthesis	P
GO:0009150	0.19	0.2	1	0.051	0.12	0.2	0.25	0.36	0.29	0.33	purine ribonucleotide metabolism	P
GO:0009152	0.16	0.2	1	0.051	0.12	0.18	0.23	0.34	0.29	1	purine ribonucleotide biosynthesis	P
GO:0009165	0.24	0.28	1	0.067	0.17	0.31	0.31	1	0.35	1	nucleotide biosynthesis	P
GO:0009199	0.082	0.2	1	0.051	0.13	0.13	0.2	0.36	1	1	ribonucleoside triphosphate metabolism	P
GO:0009201	0.054	0.2	1	0.051	0.13	0.11	0.17	0.34	1	1	ribonucleoside triphosphate biosynthesis	P
GO:0009205	0.082	0.2	1	0.051	0.13	0.13	0.2	0.36	1	1	purine ribonucleoside triphosphate metabolism	P
GO:0009206	0.054	0.2	1	0.051	0.13	0.11	0.17	0.34	1	1	purine ribonucleoside triphosphate biosynthesis	P
GO:0009259	0.2	0.2	1	0.056	0.13	0.22	0.25	0.4	1	1	ribonucleotide metabolism	P
GO:0009260	0.16	0.2	1	0.056	0.13	0.19	0.22	0.39	1	1	ribonucleotide biosynthesis	P
GO:0009605	13	15	14	4.4	1	1	3.1	3.1	2.1	1	response to external stimulus	P
GO:0009607	3.6	4	4.2	1	1	1	1	2.2	3.4	1	response to biotic stimulus	P
GO:0009611	21	25	23	17	1	1	3.7	5.8	3.5	1	response to wounding	P
GO:0009613	18	20	19	4.2	1	1	3.9	5.1	4.2	1	response to pest, pathogen or parasite	P
GO:0009618	1	1	9	1	1	inf	inf	inf	1	15	response to pathogenic bacteria	P
GO:0009653	0.29	0.19	0.22	1	1	0.47	0.28	0.31	1	1	morphogenesis	P
GO:0009791	0	0.079	0.016	1	1	0	0	0	0	0	post-embryonic development	P
GO:0009887	0.27	0.17	0.19	1	1	0.41	0.24	0.23	1	1	organogenesis	P
GO:0009894	1	7.2	3.8	1	38	1	1	1	10	12	regulation of catabolism	P
GO:0009966	1	3.3	5.5	1	1	3.9	9	12	2.9	1	regulation of signal transduction	P
GO:0012501	4.2	4.5	3.9	1	3.6	3.3	3.9	3.8	7.5	4.1	programmed cell death	P
GO:0012502	0.21	0.19	0.28	0.11	0.1	0.3	0.21	0.21	0.36	0.11	induction of programmed cell death	P
GO:0015077	0.051	1	1	0.17	1	0.2	0.29	0.35	1	1	monovalent inorganic cation transporter activity	F
GO:0015078	0.051	0.31	1	0.17	1	0.17	0.25	0.33	1	1	hydrogen ion transporter activity	F
GO:0015290	8.7	6.9	7.9	1	1	3	3	1	3.4	1	electrochemical potential-driven transporter activity	F
GO:0015291	8.7	6.9	7.9	1	1	3	3	1	3.4	1	porter activity	F
GO:0015399	0.22	1	1	0.19	1	0.37	0.36	0.41	1	1	primary active transporter activity	F
GO:0015457	1	6.4	1	1	1	inf	inf	1	inf	inf	auxiliary transport protein activity	F
GO:0015662	1	1	1	0.034	0.18	0.43	0.18	0.19	0.22	0.22	ATPase activity, coupled to transmembrane movement of ions	F
GO:0015875	1	1	4.1	inf	1	1	inf	inf	inf	1	vitamin or cofactor transport	P
GO:0015935	0.015	0.3	0.092	1	1	0.025	0.33	0.11	1	1	small ribosomal subunit	C
GO:0015985	0.049	0.2	1	0.1	0.14	0.12	0.16	0.34	1	1	energy coupled proton transport, down electrochemical gradier	P
GO:0015986	0.049	0.2	1	0.1	0.14	0.12	0.16	0.34	1	1	ATP synthesis coupled proton transport	P
GO:0015992	1	1	1	0.12	0.16	0.32	0.3	0.37	1	1	proton transport	P
GO:0016052	26	45	52	1	1	1	2.9	3.8	1	1	carbohydrate catabolism	P
GO:0016064	3.3	12	9.6	inf	5	15	34	28	inf	6.5	humoral defense mechanism (sensu Vertebrata)	P
GO:0016151	0.079	0.16	1	1	1	0.06	0.14	0.43	1	1	nickel ion binding	F
GO:0016209	3.6	3.9	3	1	4.3	3.4	4	3.7	18	4.1	antioxidant activity	F
GO:0016244	16	17	13	4	1530	inf	inf	inf	inf	inf	non-apoptotic programmed cell death	P
GO:0016247	1	6.4	1	1	1	inf	inf	1	inf	inf	channel regulator activity	F
GO:0016265	4.3	4.5	3.9	1	3.5	3.1	3.6	3.5	7.4	3.8	death	P
GO:0016469	0.049	0.2	1	0.1	0.14	0.12	0.16	0.34	1	1	proton-transporting two-sector ATPase complex	C
GO:0016564	5	1	4.7	1	1	11	17	19	1	3.4	transcriptional repressor activity	F
GO:0016568	5.1	1	6.1	1	1	4.9	4.8	6.1	1	1	chromatin modification	P
GO:0016638	1	1	1	1	0	0.17	1	0	0	0	oxidoreductase activity, acting on the CH-NH2 group of donors	F
GO:0016679	0.059	0	1	0.056	0.15	0.24	1	1	1	1	oxidoreductase activity, acting on diphenols and related substa	F
GO:0016681	0.059	0	1	0.056	0.15	0.24	1	1	1	1	oxidoreductase activity, acting on diphenols and related substa	F
GO:0016684	0.022	0.13	0.16	0.06	0.12	0.3	0.11	0.17	0.3	0.1	oxidoreductase activity, acting on peroxide as acceptor	F
GO:0017129	0	1	0.17	1	0.26	1	1	1	0	0	triglyceride binding	F
GO:0018987	16	17	13	4	765	inf	inf	inf	inf	1352	osmoregulation	P
GO:0019028	1	1	1	0.034	0.056	0.099	1	1	0	0	viral capsid	C
GO:0019199	inf	1	1	1	inf	inf	inf	1	1	29	transmembrane receptor protein kinase activity	F
GO:0019216	1	1	4.1	4.7	8.2	1	1	4.3	27	7.9	regulation of lipid metabolism	P
GO:0019219	4	1	3.4	1	1	2.5	2.5	2.1	1	1	regulation of nucleobase, nucleoside, nucleotide and nucleic ac	P
GO:0019222	3.2	1	3.3	1	1	2.5	2.6	2.4	1	1	regulation of metabolism	P
GO:0019320	22	45	50	1	1	1	2.9	3.6	1	1	hexose catabolism	P
GO:0019825	inf	1	inf	1	3.9	inf	1	inf	inf	inf	oxygen binding	F

GO:0019842	1	9.9	5.4	inf	1	1	4.5	3	1	1	vitamin binding	F
GO:0019865	1	5.7	1	1	1	1	inf	inf	inf	16	immunoglobulin binding	F
GO:0019866	0.22	0.31	1	0.089	1	0.19	0.32	0.46	0.28	1	inner membrane	C
GO:0019904	27	28	31	1	1	3.9	5.2	5.7	1	1	protein domain specific binding	F
GO:0030097	0.019	0.023	0.013	1	1	0.019	0.021	0.0086	1	1	hemopoiesis	P
GO:0030099	0.11	0	1	1	0.093	0.057	1	0	1	1	myeloid blood cell differentiation	P
GO:0030104	16	17	13	4	382	inf	inf	inf	inf	inf	water homeostasis	P
GO:0030162	inf	inf	inf	inf	474	1	1	1	1	inf	regulation of proteolysis and peptidolysis	P
GO:0030218	0	1	0.13	1	1	0	0	0	1	1	erythrocyte differentiation	P
GO:0030234	5.2	5.7	6.3	1	1	2.8	3.4	3.6	4.3	1	enzyme regulator activity	F
GO:0030235	inf	inf	inf	1	1	13	15	9.6	1	inf	nitric-oxide synthase regulator activity	F
GO:0030299	0.17	1	0.22	1	1	0.068	0	0	0.047	0.017	cholesterol absorption	P
GO:0030300	0.14	1	0.22	1	1	0	0	0	0	0	regulation of cholesterol absorption	P
GO:0030414	5.1	7.5	7.3	1	1	2.7	4.2	4.9	6.9	1	protease inhibitor activity	F
GO:0030484	inf	inf	inf	1	1	115	14	1	1	3.5	muscle fiber	C
GO:0030528	3.2	1	3	1	1	2.8	2.9	2.4	1	1	transcription regulator activity	F
GO:0030569	1	1	inf	1	inf	1	1	inf	inf	inf	chymotrypsin inhibitor activity	F
GO:0030728	0	0.079	0.0079	1	1	0	0	0	1	1	ovulation	P
GO:0030911	inf	inf	inf	1	1	inf	inf	inf	1	inf	TPR domain binding	F
GO:0042026	inf	inf	inf	1	inf	inf	inf	1	1	inf	protein refolding	P
GO:0042127	5.4	5.2	11	19	1	2.9	3.3	8	52	7.6	regulation of cell proliferation	P
GO:0042221	1	3.4	3.9	1	1	4.1	4.5	4.5	1	1	response to chemical substance	P
GO:0042254	1	1	1	1	0.19	0.027	0.035	0.05	1	0.064	ribosome biogenesis and assembly	P
GO:0042330	4.7	1	8.6	1	1	3.1	3.6	3.1	1	1	taxis	P
GO:0042448	0	0.079	0.016	1	1	0	0	0.0085	1	1	progesterone metabolism	P
GO:0042592	4.5	5.3	4.1	1	6.8	7.7	9.9	9.1	5.3	7.9	homeostasis	P
GO:0042625	1	1	1	0.12	0.15	0.47	0.31	0.41	1	1	ATPase activity, coupled to transmembrane movement of ions	F
GO:0042632	0	1	0	1	0.069	0	1	0	1	0.038	cholesterol homeostasis	P
GO:0042698	0	0.079	0.0079	1	1	0	0	0	1	1	menstrual cycle	P
GO:0042802	7.1	3.7	1	1	1	12	7.1	1	12	11	protein self binding	F
GO:0042803	1	3.5	1	1	1	4.7	15	5.5	20	18	protein homodimerization activity	F
GO:0042981	1	1	1	0.15	0.2	0.42	0.42	0.46	1	0.28	regulation of apoptosis	P
GO:0043065	0.21	0.23	1	0.12	0.11	0.31	0.3	0.32	0.42	0.13	positive regulation of apoptosis	P
GO:0043067	4	4.2	3.5	1	3.9	3.4	4	3.8	8.2	4.2	regulation of programmed cell death	P
GO:0043068	0.21	0.23	1	0.12	0.11	0.31	0.3	0.32	0.42	0.13	positive regulation of programmed cell death	P
GO:0043069	15	16	13	1	16	28	34	31	26	33	negative regulation of programmed cell death	P
GO:0043070	16	17	13	4	1530	inf	inf	inf	inf	inf	regulation of non-apoptotic programmed cell death	P
GO:0043072	16	17	13	4	1530	inf	inf	inf	inf	inf	negative regulation of non-apoptotic programmed cell death	P
GO:0043167	4.5	3.7	3.5	1	3	3.7	3.3	3.2	1	1	ion binding	F
GO:0043169	4.4	3.6	3.4	1	3.2	3.8	3.4	3.3	2.1	3.1	cation binding	F
GO:0045087	24	29	26	17	1	1	3.6	5.3	4.9	1	innate immune response	P
GO:0045255	0.017	0.1	0.32	0.079	0.18	0.014	0.097	0.35	1	1	hydrogen-translocating F-type ATPase complex	C
GO:0045259	0.017	0.1	0.32	0.079	0.18	0.027	1	1	1	1	proton-transporting ATP synthase complex	C
GO:0045428	inf	inf	inf	1	1	6	7.7	1	1	inf	regulation of nitric oxide biosynthesis	P
GO:0045429	inf	inf	inf	1	1	12	15	9.6	1	inf	positive regulation of nitric oxide biosynthesis	P
GO:0045449	4.1	1	3.5	1	1	2.6	2.4	2.2	1	1	regulation of transcription	P
GO:0046034	0.079	0.24	1	0.1	0.13	0.13	0.22	0.35	1	1	ATP metabolism	P
GO:0046138	0.25	1	1	0.13	1	0.3	0.35	1	0.27	1	coenzyme and prosthetic group biosynthesis	P
GO:0046164	22	45	51	1	1	1	2.9	3.7	1	1	alcohol catabolism	P
GO:0046209	inf	inf	inf	1	1	1	3.9	1	1	8.6	nitric oxide metabolism	P
GO:0046365	22	45	51	1	1	1	2.9	3.7	1	1	monosaccharide catabolism	P
GO:0046872	4.5	3.7	3.5	1	3	3.7	3.3	3.2	1	1	metal ion binding	F
GO:0046914	3.9	3.8	3.3	1	3.9	3.6	3.8	3.6	3	3.9	transition metal ion binding	F
GO:0046933	0.049	0.2	1	0.1	0.14	0.12	0.16	0.34	1	1	hydrogen-transporting ATP synthase activity, rotational mecha	F
GO:0046961	0.049	0.2	1	0.1	0.14	0.12	0.16	0.34	1	1	hydrogen-transporting ATPase activity, rotational mechanism	F
GO:0048156	0	1	0	1	0.054	0	1	0	1	0.038	tau protein binding	F
GO:0048168	0	1	0	1	0.054	0	1	0	1	0.038	regulation of neuronal synaptic plasticity	P
GO:0050750	0	1	0	1	0.056	0	1	0	1	0.038	low-density lipoprotein receptor binding	F
GO:0050776	1	14	21	1	1	1	7.7	13	inf	1	regulation of immune response	P
GO:0050789	3.8	3.9	4.3	1	1	2.9	3.3	3.8	2.8	1	regulation of biological process	P
GO:0050790	25	1	30	1	1	5.8	4.4	6.7	1	1	regulation of enzyme activity	P
GO:0050791	3.8	3.8	5.4	1	1	2.9	3.2	4.2	1	1	regulation of physiological process	P
GO:0050794	4.1	4.5	4.9	1	1	3.2	3.8	4.7	7.7	3.2	regulation of cellular process	P
GO:0050808	0	1	1	1	0.11	0.24	1	0.25	1	0.037	synapse organization and biogenesis	P
GO:0050817	14	19	35	11	1	6.2	8.7	20	4.4	3.2	coagulation	P
GO:0050824	16	17	13	4	1530	inf	inf	inf	inf	451	water binding	F
GO:0050874	5.1	5.8	6.1	1	1	3.7	4.8	5.6	6.3	3	organismal physiological process	P
GO:0050878	16	17	18	5.3	7.4	33	41	50	11	7.9	regulation of body fluids	P
GO:0050891	16	17	13	4	1530	inf	inf	inf	inf	inf	body fluid osmoregulation	P
GO:0050896	3.6	4.1	4.2	1	1	1	1	2.2	2.7	1	response to stimulus	P
