# Supplementary material for: GO-Diff: Mining functional differentiation between EST-based transcriptomes
Source: BMC Bioinformatics. 2006 Feb 16;7:72. doi: 10.1186/1471-2105-7-72 (PMC1388240; doi:10.1186/1471-2105-7-72)
Supplement: Additional File 4 — Procedures to evaluate GO-Diff consistency. [file 1471-2105-7-72-S4.pdf]

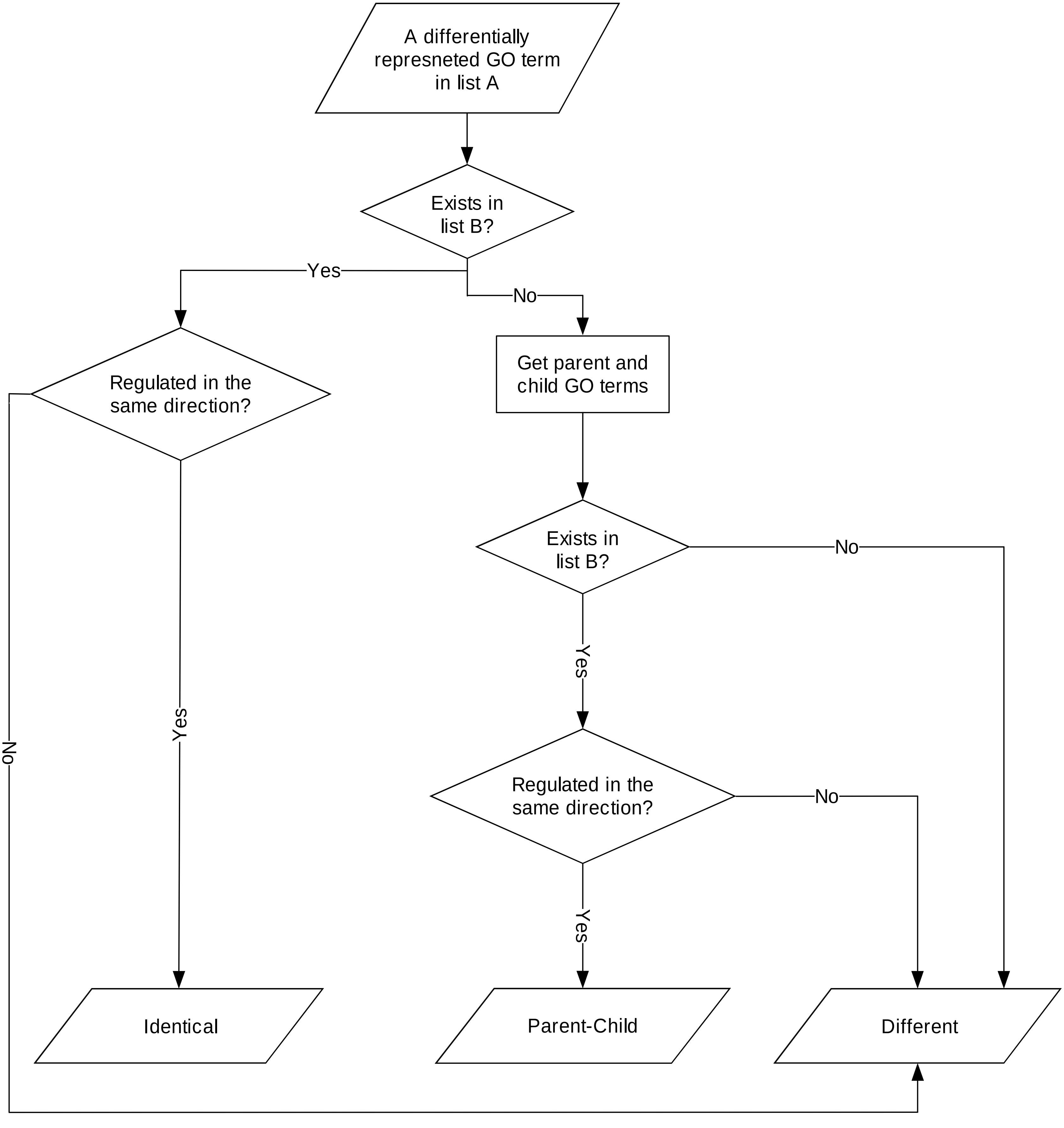

A differentially represneted GO term in list A
Exists in list B?
Yes
No
Regulated in the same direction?
Get parent and child GO terms
Exists in list B?
No
Yes
No
Regulated in the same direction?
No
Yes
Identical
Parent-Child
Different
